# Levy flight-assisted hybrid Sine-Cosine Aquila optimizer for solving chemical equilibrium problems through the Gibbs free energy minimization technique

**DOI:** 10.1038/s41598-025-22802-9

**Published:** 2025-11-14

**Authors:** Oguz Emrah Turgut, Hadi Genceli, Mustafa Asker, Ehsan Baniasadi, Mustafa Turhan Çoban

**Affiliations:** 1 Department of Industrial Engineering, İzmir Bakircay University, 35665 Izmir, Turkey; 2 Faculty of Mechanical Engineering, Yıldız Technical University, 34349 Istanbul, Turkey; 3 Department of Mechanical Engineering, Aydın Adnan Menderes University, 09010 Aydın, Turkey; 4 Energy and Bioproducts Research Institute (EBRI), College of Engineering and Physical Science, Aston University, Birmingham, B4 7ET UK; 5 Department of Mechanical Engineering, Piri Reis University, 34940 Istanbul, Turkey

**Keywords:** Aquila optimizer, Chemical equilibrium, Sine-Cosine algorithm, Gibbs free energy minimization method, Chemical engineering, Mechanical engineering

## Abstract

This research proposes a novel hybrid metaheuristic optimization framework that combines the Aquila Optimization algorithm with the Sine-Cosine Optimizer to find equilibrium points of reacting components under specified operational reaction conditions. The method aims to address the exploitative limitations of the standard Aquila algorithm by incorporating oscillatory sine-cosine movements into the hybrid optimizer, which is one of the significant drawbacks of the base Aquila algorithm that should be addressed. The effectiveness of the hybrid approach is thoroughly tested on a suite of 100 multidimensional unimodal and multimodal benchmark cases, with results compared to those from well-known literature optimizers. Additionally, twenty-eight 30-dimensional benchmark functions from the 2013 Congress on Evolutionary Computation competition are used to evaluate the prediction performance. Three multidimensional constrained engineering design problems are also solved, and their results are compared with those from other literature optimizers. The findings show that the hybrid algorithm produces the best estimates and ranks first among competing algorithms based on average ranking results. To further verify its robustness and accuracy, three more complex chemical equilibrium problems are solved using the Gibbs Free Energy minimization method. The predictions are benchmarked against recent metaheuristic algorithms for each case, demonstrating that the proposed hybrid effectively overcomes the challenges of highly nonlinear and non-convex free energy surfaces, achieving higher solution consistency while finding minimum objective function values across different chemical equilibrium scenarios.

## Introduction

 Various mathematical algorithms have been rapidly developed over the past two decades to overcome the algorithmic drawbacks of traditional optimization methods, leveraging their characteristics such as flexibility in solving multidimensional problems and notable ability to avoid local pitfalls in the search space. The advantages of stochastic-based metaheuristic optimizers make them better alternatives to contemporary heuristic search methods for solving problems with intricate and complex functional characteristics. The current literature categorizes the general concept of metaheuristic algorithms into four categories based on their primary inspiration, facilitating a more plausible and rational classification among them. The first commanding category involves swarm-based algorithms, which simulate the characteristic flocking and schooling behaviors of fish and birds during their extensive food search process. Swarm-based algorithms are activated by intelligently devised manipulation schemes, through which every candidate solution updates itself, relying on a simple yet effective search equation to explore the solution domain and achieve the optimal global answer to the problem with minimal computational effort. Particle Swarm Optimization (PSO)^1^, Artificial Bee Colony (ABC)^2^, and Ant Colony Optimization (ACO)^3^ methods are the predecessors of modern swarm intelligence optimizers, most of which are considerably influenced by these trailblazing methods, particularly in the development of search equations performed during the iterative process. Evolutionary algorithms belong to the second category, mainly inspired by the governing laws of evolution in nature. Randomly produced population individuals, occupying the trial solution matrix, evolve within subsequent generations as the iterations proceed, and predefined evolutionary search techniques are employed to reach the best solution. Developing population members are regrouped and updated using the governing search scheme in each generation, which combines the best individuals obtained so far into a single population over iterations to select the most feasible solution among the possible alternatives. The Genetic Algorithm (GA)^4^ is one of the first members of this category, representing the basic principles of the “survival of the fittest” concept. The Differential Evolution (DE) algorithm^5^ is another member of the class of evolutionary-based algorithms, simulating Darwin’s theory of evolution while probing the search domain to explore the best possible solutions. Evolution Strategy (ES)^6^ and Biogeography-based Optimization (BBO)^7^ can also be considered prominent members of the evolutionary algorithms. The second category deals with human-based stochastic algorithms, which mimic the individual efforts or intelligently devised strategies humans employ while solving a particular problem in daily life. Teaching-Learning-Based Optimization (TLBO)^8^ simulates the interaction of general teaching and learning activities in a classroom. Harmony Search (HS)^9^ is a population-based, human-inspired algorithm that simulates the music improvisation of a composer, focusing on finding the perfect harmony. The Imperialist Competitive Algorithm (ICA)^10^ influences imperialistic competition among conflicting countries, where the stronger country dominates the weaker one and takes possession of the colonies, thereby relinquishing its ruling power to enhance its sovereignty. Political Optimizer (PO)^11^ and Collective Decision Optimizer (CDO)^12^ can also be categorized into the concept of human-based algorithms. Physics-based optimizers are another distinct group, composed of algorithms that simulate the governing laws of physical and natural processes, which have been successfully implemented to find near-optimal solutions to related optimization problems. The Gravitational Search Algorithm (GSA)^13^ is inspired by the gravitational interactions between two or more neighboring masses and converts Newton’s laws of physics into a metaheuristic algorithm concept. The Simulated Annealing (SA)^14^ algorithm is one of the pioneers of physics-based methods. It simulates the annealing process in materials science as a set of mathematical equations that allow us to obtain the optimal solution to the problem. Kaveh et al.^15^ proposed Thermal Exchange Optimization (TEO), which numerically models Newton’s second law of cooling formulations during the iterative search process.

During the last two decades, metaheuristic optimizers have exerted their ruling authority over the conventional methods in computational sciences^16–27^; however, controversial confusion arises between the research community as to which existing algorithm performs when all available optimization problems are considered, why there is a need to develop a new algorithm rather than the application of the existing contemporary alternatives, and why researchers should employ ameliorative and innovative adjustments over the current stochastic optimizers. A satisfactory answer can be found in the implications of the No Free Lunch Theorem^28^, which states that no algorithm can accurately obtain the best global solution for all optimization problems, as it may work well for some problem instances. At the same time, they tend to collapse into the other class of issues. A fair conclusion can be drawn that the average prediction performance of all measurement methods is nearly equal. Researchers propose numerous innovative performance improvement strategies to overcome the algorithmic drawbacks of the developed optimizers, including poor convergence performance and a tendency to become trapped in a local minimum. Motivated by the outcomes of the No Free Lunch Theorem, they develop innovative enhancements over existing optimizers by devising an intelligently designed algorithmic structure. Hybridizing two or more metaheuristics in a single framework is the most employed solution improvement procedure, which finds its feasible applications in many research studies published up to now, relying on the complementary search characteristics of the used algorithms, which create a synergetic interaction between them and this interplay compensate for their search deficiencies to some extent, enabling to provide more promising predictions compared to their single applications^29–31^. Another effective way to enhance the overall solution quality in metaheuristic algorithms is to incorporate pseudo-random numbers generated from chaotic maps into the base algorithm, rather than using uniformly distributed random numbers, which facilitates the prevalent stochasticity in the algorithm. The current literature comprises numerous chaos-enhanced metaheuristic optimizers^32–34^, whose general prediction performance has been significantly improved by integrating chaotic numbers, thanks to their effectiveness in enhancing the exploration and exploitation mechanisms through the production of high-level randomness. Recently published studies deal with a novel solution quality improvement procedure based on the Q-learning concept, which has gained a great deal of interest among the members of the metaheuristic community as this innovative concept allows the algorithm to reach unexplored regions of the search space more effectively, thanks to the favorable advantages of using the recorded data points in Q-table, which successfully guides the responsible search agents during iterations to approach the best answer. Hsieh and Zu^35^ integrate the fundamentals of the Q-learning concepts into the PSO algorithm to direct the search process to the optimal solution. Zamli et al.^36^ benefit from the compiled data of the Q-table to identify the best search characteristics of the Sine Cosine Algorithm, which relates to the correct switching frequency between the sine and cosine function-based search equations during the ongoing iterations. The Q-learning mechanism picks the best possible option between two alternative functions, relying on the domain information collected in the Q-table. Liao and Li^37^ proposed a novel variant of the DE algorithm by integrating four different mutation strategies into the Q-learning concept and taking advantage of the Q-table through which the contestant mutation schemes communicate with each other based on their respective penalties or reward points and control the evolutionary search process by the recommendations extracted from the accumulated environmental data.

This study integrates various improved Sine Cosine algorithm (SCA) variants into a standard Aquila optimizer to enhance its overall solution accuracy and efficiency. It is observed that the Aquila Optimization algorithm (AQUILA) lacks a significant balance between exploration and exploitation mechanisms, resulting in slow convergence, premature entrapment in local solutions, and suboptimal solution quality in high-dimensional optimization problems. Previous research studies associated with the above-mentioned different SCA variants reveal that these optimizers are prolific metaheuristic methods that can reliably improve general solution diversity within the population, along with their ease of implementation and less computational cost burden on the processor. Therefore, this study aims to alleviate the premature solution convergence to local points inherent in the AQUILA by integrating enhancements based on sine-cosine oscillations. This is a significant research gap evident in the literature, which will be addressed by the intelligent integration proposed in this research study. To test the effectiveness of the proposed hybrid, 30D and 500D benchmark functions, comprising both unimodal and multimodal problems, were solved, and their respective predicted answers were compared against those of some reputable state-of-the-art optimizers. Moreover, a Wilcoxon rank sum test analysis was performed to test the statistical significance of the solutions obtained. The robustness and accuracy of the hybrid algorithm are then evaluated on 30D test problems employed in CEC-2013 competitions. Three real-world design problems will be solved, and the respective results will be compared to those of cutting-edge optimizers. Finally, the hybrid optimizer is utilized to solve Gibbs free energy minimization problems, which are highly nonlinear and require a sophisticated approach to attain the global optimal solution. To the best of the author’s knowledge, solving chemical equilibrium problems using the merits of the Gibbs Free Energy minimization method has not been comprehensively investigated in past literature studies associated with phase equilibrium problems. Furthermore, none of the completed literature works have accomplished a comparative study between the newly emerged metaheuristic algorithms and chemical equilibrium problems, another novelty proposed in this study. This research study makes the following contributions, which are explained in novel ways and highlighted in the following bullet points.

- After an exhaustive literature survey, it is understood that the original Aquila optimizer suffers from unexpected entrapment of local solutions resulting from the insufficient search capability of the algorithm.
- A novel Levy Flight-assisted dynamically varying weight parameter integrated Sine-Cosine optimizer is embedded into the base Aquila method to improve the inherent characteristic diversification and intensification mechanism of the algorithm through the favorable contributions of random numbers generated by Levy Flight, chaotic random numbers produced by Ikeda Map, and an iteratively adjusted weight parameter that is responsible for balancing exploration and exploitation of the hybridized method.
- AQSCA intelligently integrates the nature-inspired AQUILA, simulating the hunting behaviors of eagles, with the mathematical search equations of the SCA. This combination entails a favorable integration of AQUILA’s exploration and trigonometric-based exploitation of the SCA algorithm, producing a novel optimization framework that leverages the strengths of the advantageous search equations of each algorithm. This hybrid perturbation mechanism augments both global diversification and local precision, proposing a novel approach to escape local optimum points trapped in the search domain.
- AQSCA has been benchmarked against different types of test problems with varying functional characteristics and problem dimensionalities, and respective results have been compared to those obtained for the state-of-the-art cutting-edge metaheuristic optimizers.
- AQSCA is applied to solve Gibbs Free Energy Minimization problems, which is a critical challenge in the thermodynamic design process in chemical engineering. The goal is to determine the accurate equilibrium composition of a chemical system by minimizing the Gibbs Free Energy function under defined problem constraints. In this context, it is advantageous to employ AQSCA in this type of design problem, as AQSCA can efficiently explore intrinsic, complex, and high-dimensional energy surfaces, enabling the refinement of equilibrium compositions. Furthermore, this approach, which leverages the merits of metaheuristic algorithms over conventional analytical optimization methods, proposes an alternative solution strategy for solving chemical equilibrium problems.

## Literature survey on different applications of the Aquila algorithm

Despite its recent emergence, the AQUILA has been applied in various engineering disciplines in past studies. The main goal of using AQUILA is to examine its efficiency in terms of solution robustness and accuracy, as these aspects have not been well established in most previous research. This methodology was developed in 2021, so not much time has passed since its initial proposal. Researchers have addressed the inherent flaws of this algorithm, as noted in the literature, and have suggested alternative strategies to address two common issues: premature convergence and entrapment in a local minimum.

The Aquila algorithm has been applied in various engineering fields, ranging from PID controller design^38^ to image classification^39^. AlRassas et al. ^40^ developed an Adaptive Neuro-fuzzy Inference System (ANFIS) using the AQUILA algorithm to forecast oil production between different oil fields in Yemen and China. It is observed that reliable estimations have been obtained by utilizing the proposed AQUILA-supported ANFIS method, demonstrating its superiority over the state-of-the-art optimizers in terms of prediction accuracy. Another forecasting model was proposed by Ma et al.^41^, which is associated with the future prediction of China’s rural community population through a novel grey Bernoulli model whose model parameters are extracted using the AQUILA optimizer. Another novelty in this research is the integration of Quasi-opposition learning and wavelet mutation strategies into the base AQUILA algorithm, which enables the search procedures to meet very high standards. Wang et al.^42^ proposed using the AQUILA algorithm for the optimal techno-economic design of a hybrid energy system comprising a Solid Oxide Fuel Cell system-based integrated gas turbine and a Proton Exchange Electrolyzer. Bas^43^ developed a binary Aquila Optimizer for solving 0–1 knapsack problems and compared the respective prediction results with those obtained from other newly emerged metaheuristic optimizers. The AQUILA algorithm has enhanced the lifetime and energy efficiency of wireless sensor networks by employing an efficient search mechanism, which improves energy balancing within clusters across the entire sensor network during communication^44^. El-Ela et al.^45^ proposed using the AQUILA algorithm for accurate parameter estimation of the Weibull distribution of wind data. The accumulated error between the measured set of data and the extracted model parameters acquired by AQUILA, along with other analytical methods, has been comparatively analyzed, and the best prediction among them is selected. The AQUILA algorithm was implemented to minimize the thermal error of an electric spindle by determining the correct locations on the spindle that yield the most accurate measurements^46^. Mehmood et al.^47^ employed the AQUILA algorithm to estimate unknown parameters of the Control Autoregressive Model. They further considered different scenarios with various noise levels to assess this algorithm’s general optimization performance.

Various modifications have been made to this algorithm to eliminate its inherent algorithmic disadvantages. The AQUILA algorithm involves two explorative and two exploitative search mechanisms deemed sufficient for reaching the global best solution to the optimization problem within a defined number of maximum iterations. However, previous efforts on its successful application to real-world optimization problems from different domains reveal that it needs to be more balanced between the governing exploration and exploitation search mechanisms, which is the main flaw resulting in poor convergence and entrapment in local minima^48–51^. Zhang et al.^52^ hybridized the search equations of the AQUILA algorithm with those of the Arithmetic Optimization algorithm to reach better performance in solution accuracy. They also borrow the decisive “energy parameter (E)” from the Harris Hawks Optimization algorithm to achieve a more balanced approach between the exploration and exploitation phases within the hybrid method. They compared the prediction results obtained for multidimensional benchmark functions with nine literature optimizers, and promising outcomes were observed. Yu et al.^53^ attempted to overcome the algorithmic disadvantages of the AQUILA optimizer by incorporating three innovative search procedures, including a novel restart strategy, opposition-based learning, and chaotic local search, to exploit fertile regions encountered in successive iterations. The hybrid algorithm is evaluated on a test suite of CEC-2019 benchmark problems, demonstrating its effectiveness. Gao et al.^54^ introduced a Search Control Factor and random opposition learning-based method into the AQUILA algorithm to enhance its intelligently designed hunting strategies, resulting in a significant improvement in obtaining more accurate predictions. Ekinci et al.^55^ proposed a novel hybrid algorithm that integrates the AQUILA optimizer and the Nelder-Mead simplex search method to maintain efficient control of an air-fuel ratio system in a spark ignition engine, which is based on a proportional-integral controller. Nirmalapriya et al.^56^ hybridized the AQUILA algorithm with the Spider Monkey optimizer and fractional calculus to iteratively adjust the model parameters of the channel-wise feature pyramid network for brain tumor classification. It is observed that the proposed neural network model accurately predicts the actual data set with a total root mean square error of 0.089. Zhang et al.^57^ aimed to enhance the general search performance of the Hunger Search Algorithm by incorporating the search equations of the AQUILA optimizer and multiplicative map-based chaotic numbers into the base hybrid scheme. Liu et al.^58^ developed a reinforcement learning-based hybrid algorithm that combines the contributions of Aquila Optimization and an improved Arithmetic Optimization Algorithm. This approach provides an intelligently devised scheme that dynamically selects between these two competing algorithms based on their respective optimization success during iterations. A data clustering approach has been developed using a hybrid algorithm that combines an Aquila optimizer with integrated search operators from Arithmetic Optimization and Differential Evolution algorithms to address the shortcomings of the original Aquila algorithm, such as stagnation in local optimum points and premature convergence^59^.

In this study, a novel hybrid optimization algorithm is proposed to address the algorithmic drawbacks of the AQUILA algorithm. Different variants of SCA have been integrated into the original Aquila optimizer to enhance its overall solution accuracy and robustness^55–60^. A novel hybrid AQSCA is proposed, combining two variants of the SCA within the context of this research study. The following section explains the fundamentals of AQUILA and its basic mutation scheme.

### The proposed hybrid algorithm

#### Basics of the Aquila optimizer

The AQUILA algorithm is a nature-inspired metaheuristic optimizer inspired by the intrinsic hunting behaviors of eagles^61^. It is one of the prominent members of swarm-based metaheuristic optimizers, taking its main inspiration from the collective foraging behaviors of aquilas, similar to many available stochastic optimizers belonging to the categorical branch of swarm intelligence. Aquila is known for its bravery during attacks on prey individuals. Male aquilas can find more prey during solo attacks by utilizing their pace, agility, and sharp talons to hunt rabbits. They employ four different foraging strategies, each with many characteristic behaviors, to confuse and surprise the fleeing prey. The following expressions precisely explain the distinctive attacking methods of the Aquilas.

- The first method is related to a high soar with a vertical stoop in which the foraging aquila soars higher to the ground to explore the areas where the prey is possibly located more effectively. Once it detects the target, aquila performs a long-angled glide with an upward speed to grab the prey.
- The second strategy deals with a low flight accompanied by a short glide attack, the most utilized method for hunting aquilas. Then, the prey is followed closely by the predator.
- The third foraging strategy is a low flight with a descent attack. In this attacking movement, aquila flies down to the ground, selects the prey, and grabs the prey on its neck.
- The fourth and final hunting method is associated with walking on the ground and grabbing the fleeing prey.

The above-given hunting strategies of aquilas are the main inspirations for the general framework of the AQUILA optimization algorithm. The following subsections explain how these hunting methods are simulated into mathematical models that establish the governing manipulation schemes of the Aquila Optimizer.

## Generating the initial population

Since the Aquila optimization algorithm is a population-based algorithm, the initial population, composed of candidate solutions (X), should be stochastically generated between the predefined lower (low) and upper (upp) bounds of the search space. The solution with the lowest fitness value is considered the best solution obtained by the end of the successive iterations.

1 $$X = \left[ {\begin{array}{*{20}l} {\begin{array}{*{20}l} {x_{{1,1}} } & \cdots \\ \vdots & \vdots \\ \end{array} } & {\begin{array}{*{20}l} {x_{{1,j}} } & \cdots \\ \cdots & \cdots \\ \end{array} } & {\begin{array}{*{20}l} {x_{{1,D - 1}} } & {x_{{1,D}} } \\ \vdots & \vdots \\ \end{array} } \\ {\begin{array}{*{20}l} {x_{{i,1}} } & \vdots \\ \vdots & \vdots \\ \end{array} } & {\begin{array}{*{20}l} \ddots & \ddots \\ \ddots & \ddots \\ \end{array} } & {\begin{array}{*{20}l} \vdots & {x_{{i,D}} } \\ \vdots & \vdots \\ \end{array} } \\ {\begin{array}{*{20}l} {x_{{N - 1,1}} } & \vdots \\ {x_{{N,1}} } & \cdots \\ \end{array} } & {\begin{array}{*{20}l} \cdots & \cdots \\ {x_{{N,j}} } & \cdots \\ \end{array} } & {\begin{array}{*{20}l} \vdots & {x_{{N - 1,D}} } \\ {x_{{N,D - 1}} } & {x_{{N,D}} } \\ \end{array} } \\ \end{array} } \right]$$

Where *X* denotes the aquila population comprised of D-dimensional N solutions, which are randomly produced by the expression given below

2 $$\:{x}_{ij}={low}_{j}+\left({upp}_{j}-{low}_{j}\right)\times\:rnd\left(\text{0,1}\right),\:i=\text{1,2},3,\dots\:,N\:\:\:j=\text{1,2},3,\dots\:,D$$

Where *rnd*(0,1) is a uniformly distributed random number defined in the range [0,1]; *low*_j_ is the lower bound of the *j*th design variable; *up*p_j_ is the *j*th dimension of the upper bound of the defined optimization problem.

The AQUILA simulates the four distinct foraging behaviors of aquilas, which are briefly explained in the previous subsection. AQUILA can quickly shift from exploration to exploitation phases using different search schemes based on the condition that if *iter* < 0.66 *Maxiter*. Exploration is practiced if this algorithmic phase is satisfied; otherwise, the exploitation mechanism is activated. Intrinsic hunting skills of aquilas are converted into a dexterous optimization algorithm by the following mathematical models.

## Expanded exploration (X_1_)

In this phase, the aquila population detects fertile areas where prey is abundant and makes a dazzling attack by soaring high and then diving vertically. This attacking behavior enables aquila on the flight to effectively probe around the search domain and determine the most available prey formulated by the following.

3 $$\:{{{X}_{1}^{t+1}=X}_{best}^{t}\times\:\left(1-\frac{t}{T}\right)X}_{M}^{t}-{X}_{best}^{t}\times\:rnd\left(\text{0,1}\right)$$

$\:{X}_{1}^{t+1}$is the solution obtained for the next iteration t + 1, which is valid for the first hunting method (*X*_*1*_);

$\:{X}_{best}^{t+1}$ is the best solution obtained until the *t*th iteration, locating the approximate position of the prey. The iterative parameter $\:\left(1-\frac{t}{T}\right)\:$controls the transition between exploration and exploitation mechanisms; $\:{X}_{M}^{t}$ is the mean values of the foraging aquilas in the swarm at *t*th iteration are calculated by Eq. (4); *t* is the current iteration; *T* is the maximum number of iterations; and *rnd*(0,1) a random number between 0 and 1.

4 $$\:{X}_{M}^{t}=\frac{1}{N}{\sum\:}_{j=1}^{N}{X}_{j}^{t}\:\:\:\:\:\:\:\:\:\:\:\:\:\forall\:j=\text{1,2},3,\dots\:,D$$

## Narrowed exploration (X_2_)

The second foraging method (*X*_*2*_) aims to blitz the running prey individuals by efficiently performing circular movements above the ground after exploring the search space. The following expression can model this behavior

5 $$\:{X}_{2}^{t+1}={{X}_{best}^{t}\times\:Levy\left(D\right)+X}_{R}^{t}+(y-x)\times\:rnd\left(\text{0,1}\right)$$

Where $\:{X}_{2}^{t+1}$ is a candidate solution for the next iteration, produced by the second hunting method (*X*_*2*_), $X_{R}^{t}$is a random answer selected from the aquila swarm composed of *N* different individuals, and the *Levy(D)* function generates D-dimensional random numbers drawn from the Levy distribution, which is calculated by

6 $$\:Levy\left(D\right)=s\times\:\frac{u\times\:\sigma\:}{{\left|v\right|}^{1/\beta\:}}$$

Where *s* is a constant number fixed to 0.01; *u* and *v* are two different random numbers defined within [0,1]; and the parameter *σ* is calculated by

7 $$\:\sigma\:=\left(\frac{{\Gamma\:}(1+\beta\:)\times\:\text{s}\text{i}\text{n}\left(\frac{\pi\:\times\:\beta\:}{2}\right)}{{\Gamma\:}\left(\frac{1+\beta\:}{2}\right)\times\:{\upbeta\:}\times\:{2}^{\left(\frac{\beta\:-1}{2}\right)}}\right)$$

where *β* is a fixed number equal to 1.5, $\:{\Gamma\:}(.)$ represents the Gamma function; parameters *x* and *y* are used to form the spiral shape of the attacking movement employed by the responsible search mechanism, and computed by

8 $$\:y=r\times\:\text{c}\text{o}\text{s}\left(\theta\:\right)$$

9 $$\:x=r\times\:\text{s}\text{i}\text{n}\left(\theta\:\right)$$

Where.

10 $$r = rd_{{1 - 20}} + sv_{1} \times D_{{1 - dim}}$$

11 $$\theta = - \omega \times D_{{1 - dim}} + 1.5\Pi$$

*rd*_*1 − 20*_ takes a random value between 1 and 20 during iterations; *sv*_*1*_ is a small-valued number equal to 0.00565; *D*_*1 − dim*_ is an integer number defined between 1 and the dimensional length of the search space (*D*); and *ω* is fixed to 0.005.

### Expanded exploration

The third attacking strategy is devoted to an expanded exploration of the search space, in which fertile prey areas are accurately pinpointed and hunting aquilas are ready to attack. Aquilas make a vertical attacking move, descending vertically to the ground to comprehend the first reaction of the prey individuals. This hunting move is designed to exploit the selected fertile area, which is abundant in prey animals. This attacking behavior can be mathematically expressed by the following

12 $$\:{{{X}_{3}^{t+1}=0.1\times\:(X}_{best}^{t}-X}_{M}^{t})-rnd\left(\text{0,1}\right)+0.1\times\:\left(\left(upp-low\right)\times\:rnd\left(\text{0,1}\right)+low\right)\:$$

Where $\:{\text{X}}_{3}^{t+1}$ is a candidate solution obtained by the third hunting method (*X*_*3*_);$\:{\text{X}}_{best}^{t}$ is the approximate location of the prey individual retained within the iteration *t*; $\:{\text{X}}_{M}^{t}$ is the mean value of the aquila population, which is calculated by Eq. (4); *rnd*(0,1) is a random value between 0 and 1; and *low* and *upp* are respectively the lower and upper search limits of the problem.

## Narrowed exploitation

The fourth attacking method (*X*_*4*_) occurs when hunting aquilas get close to the prey by making stochastic movements to confuse it. It is simply walking towards the prey and grabbing it firmly to avoid running away from the blitz. This attacking behavior is associated with intensive exploitation of the promising regions over the search space discovered during the successive iterations and explicitly formulated by

13 $$\:{X}_{4}^{t+1}=Q{F}^{t}\times\:{X}_{best}^{t}-\left({G}_{1}\times\:{X}^{t}\times\:rnd\left(\text{0,1}\right)\right)-\left({G}_{2}\times\:Levy\left(D\right)\right)+rnd\left(\text{0,1}\right)\times\:{G}_{1}$$

Where $\:{\text{X}}_{4}^{t+1}$ is a trial solution obtained by the fourth attacking method (*X*_*4*_); *QF* is an iterative parameter employed to maintain a transition between search strategies and calculated by Eq. (14); *G*_*1*_ represents the various movements of preys performed to elope from the surrounding aquila attacks and computed by Eq. (15); *G*_*2*_ is another iterative parameter decreasing from 2 to 0 used for referencing the flight slope of the hunting aquilas and expressed by Eq. (16); and *X*^*t*^ stands for the current solution generated within the iteration *t*.

14 $$\:{QF}^{t}={t}^{\frac{2\times\:rnd\left(\text{0,1}\right)-1}{{(1-T)}^{2}}}$$

15 $$\:{G}_{1}=2\times\:rnd\left(\text{0,1}\right)-1$$

16 $$\:{G}_{2}=2\times\:(1-\frac{t}{T})$$

The pseudo-code of the Aquila optimizer is briefly described below in Table 1.

**Table 1:**
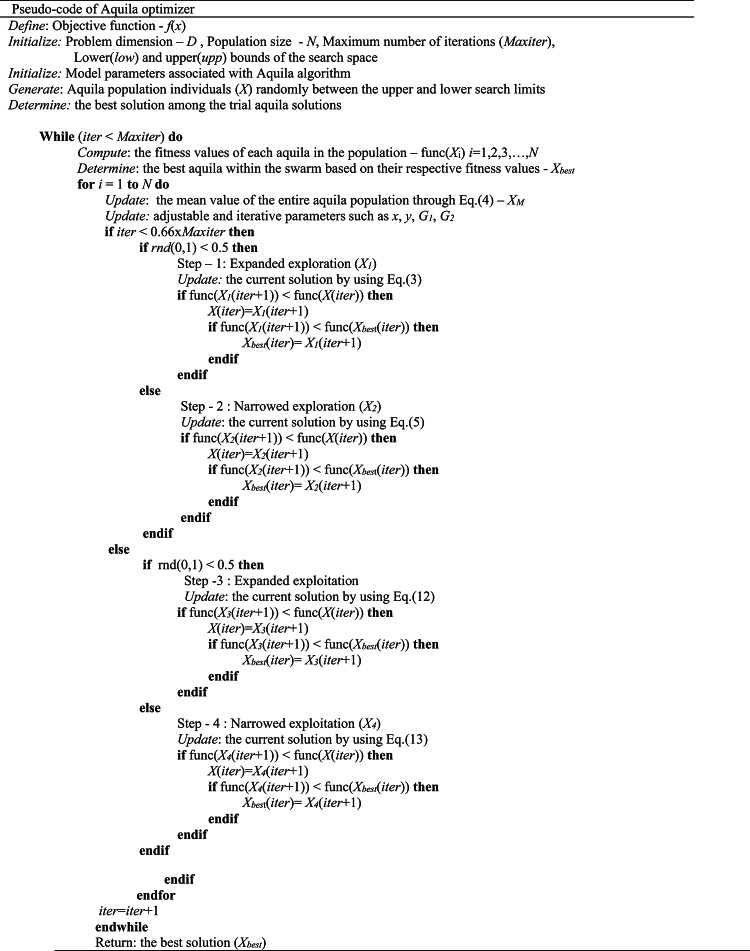
Aquila optimizer pseudo-code.

## Sine cosine algorithm

The Sine Cosine Optimization (SCA) algorithm was proposed by Mirjalili^62^ to solve real-world optimization problems. It is a new-generation population-based metaheuristic optimizer that relies on the functional behaviors of the trigonometric sine and cosine functions, enabling the algorithm to explore undiscovered regions of the search space quickly. The key point in this newly emerged algorithm is adjusting the spatial distance between the best solution obtained so far and each trial solution throughout the iterations, directing their movements towards the global best answer to the problem. The algorithm can maintain a plausible balance between exploration and exploitation phases, thanks to the practical probing features resulting from the favorable merits of sine and cosine functions, and by employing fewer model parameters compared to other available metaheuristic optimizers in the literature. The algorithm utilizes the equations below concurrently to update the current set of solutions.

17 $$\:\begin{array}{c}{\text{X}}_{i}^{t+1}={X}_{i}^{t}+{r}_{1}\cdot\:\text{s}\text{i}\text{n}\left({r}_{2}\right)\cdot\:\left|{r}_{3}\cdot\:{X}_{best}^{t}-{X}_{i}^{t}\right|\\\:{\text{X}}_{i}^{t+1}={X}_{i}^{t}+{r}_{1}\cdot\:\text{c}\text{o}\text{s}\left({r}_{2}\right)\cdot\:\left|{r}_{3}\cdot\:{X}_{best}^{t}-{X}_{i}^{t}\right|\end{array}\:$$

Where $\:{X}_{i}^{t}$ and $\:{\text{X}}_{i}^{t+1}$ are, respectively, the locations of the current solutions at iteration *iter* and *iter*+1. Model parameters *r*_*1*_, *r*_*2*_, *r*_*3*_, and *r*_*4*_ are decisive expressions used for determining new positions of candidate solutions. The following expression emerges for the ruling solution update mechanism by combining these two equations within a single framework.

18 $$\:{\text{X}}_{i}^{t+1}=\left\{\begin{array}{c}{X}_{i}^{t}+{r}_{1}\cdot\:\text{sin}\left({r}_{2}\right)\cdot\:\left|{r}_{3}\cdot\:{X}_{best}^{t}-{X}_{i}^{t}\right|,\:\:\:\:{r}_{4}\le\:0.5\\\:{X}_{i}^{t}+{r}_{1}\cdot\:\text{cos}\left({r}_{2}\right)\cdot\:\left|{r}_{3}\cdot\:{X}_{best}^{t}-{X}_{i}^{t}\right|,\:\:\:\:{r}_{4}>0.5\end{array}\right.$$

Where r_4_ is a random value in [0,1] and a switch parameter used for shifting between sine and cosine-based manipulation equations defined in Eq. (18). Parameter r_1_ is conducive to locating a position between the current best and new solution, for which *r*_*1*_ < 1 means the exploitation phase is activated. At the same time, for *r*_*1*_ > 1, the exploration mechanism is dominant. To maintain a balance between these two phases, the following equation is proposed for calculating the numerical value of *r*_*1*_

19 $$\:{r}_{1}=2\left(1-\frac{t}{{t}_{Max}}\right)$$

Another parameter, r_2,_ takes a random value within the interval [0,2π]. It determines a random location in the search space that decides how far the candidate-generated solution moves toward or away from the destination point. A random number specified in the range [0,1], *r*_*3*,_ is utilized to determine the degree of contribution of the best solution (*X*_*best*_) to the produced trial solution for the next iteration. The periodic motion generated by trigonometric sine and cosine functions entails a prolific ability to exploit the fertile search regions on which candidate solutions are developed in relation to one another. Suppose a new solution is produced outside the search space between the best and current solutions. In that case, the global search mechanism is intensified to diversify the solution domain as much as possible, facilitating the exploration mechanism. A classical pseudo-code representation of the Sine-Cosine algorithm is provided in Table 2.

**Table 2:**
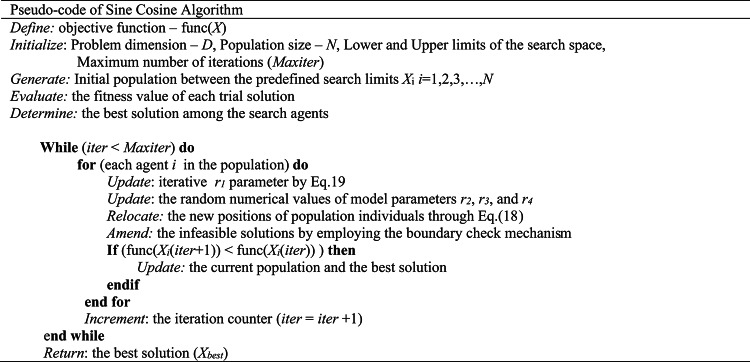
Pseudo-code of Sine-Cosine Algorithm.

### The proposed sine cosine algorithm enhanced hybrid optimizer (AQSCA)

This research study aims to improve the intrinsic search deficiencies of the AQUILA algorithm by integrating the modified manipulation schemes of SCA. Several promising attempts in the literature have been made to enhance the quality of the solution by incorporating SCA into base metaheuristic optimizers. The primary aim in these cases is to leverage the SCA’s dexterous probing capabilities over promising search regions, thereby enhancing the algorithm’s exploitation capabilities to a greater extent. Previous researchers have also revealed that SCA can be combined with an algorithm that requires more substantial or sufficient conditions to overcome its characteristics and algorithmic disadvantages, thanks to the complementary functional behaviors of the trigonometric sine and cosine functions in generating more diversified trial solutions. To provide a few examples of past studies concerning these types of hybridizations, previous research is a prominent example. Nenevath and Jatoth^63^ hybridized DE with the SCA to attain better capability to avoid local optima entrapment and premature convergence. They employed their hybrid method on multi-dimensional global optimization problems, and in a particular case, this involved tracking an object in video sequences. Mawgoud et al.^64^ proposed a hybrid methodology that combines the Arithmetic Optimization Algorithm with the Sine Cosine Algorithm to determine the optimal allocations and sizes of battery energy storage systems in radial energy distribution systems. A hybrid algorithm combining Whale Optimization, SCA, and a search scheme based on the Levy flight mechanism is presented by Seyyedabbasi^65^ within a single optimization framework to solve twenty-three global optimization problems. Vandrasi et al.^66^ constructed a hybrid framework that relies on a combination of Chimp and sine Cosine optimization algorithms for parameter extraction of solar photovoltaic modules. Fakhouri et al.^67^ attempted to address some algorithmic drawbacks of PSO, including a low convergence rate and an imbalance between exploration and exploitation, by integrating the SCA and Nelder-Mead simplex algorithms. SCA supports the base PSO algorithm in augmenting the exploration phase, while the Nelder-Mead method is utilized to intensify promising regions in the search space. Singh and Kaur^68^ hybridized the SCA with the Harmony Search optimizer to solve real-world constrained engineering design problems. Secui and Rancov^69^ proposed a hybrid SCA–Flower Pollination algorithm for solving economic dispatch problems.

Encouraged by previous studies on the variety of hybridization schemes of SCA, this research study aims to enhance the optimization capability of the AQUILA algorithm by concurrently using two modified variants of the SCA. Due to its ease of implementation and ability to generate sufficient solution diversity within the population, the SCA is a favorable candidate for improving general solution quality within the evolving population. This study also introduces the Levy flight concept into various variants of SCA to further enhance the diversity of solutions in the matrix. Long et al.^70^ proposed an improved SCA for solving high-dimensional optimization problems. The position update mechanism of SCA is facilitated by introducing an inertia weight, which accelerates global convergence and reduces the likelihood of local minimum entrapment to some degree.

The PSO algorithm is one of the most popular swarm intelligence-based algorithms, which different researchers have consistently modified to improve overall solution quality in the swarming particle population. Empirical studies suggest that assigning a relatively large inertia weight to the population individuals in PSO promotes better exploration, while lower values of this parameter give rise to faster convergence. Therefore, Shi and Eberhart^71^ proposed a linearly decreasing weight parameter to balance these complementary search mechanisms throughout iterations. Long et al.^70^ borrowed the concept of iteratively adjusting weight parameters and adapted this model to SCA, aiming to maximize the diversification of the search space. The modified solution update mechanism takes the final form of

20 $$\:{\text{X}}_{i}^{t+1}=\left\{\begin{array}{l}{w\left(t\right)\cdot\:X}_{i}^{t}+{r}_{1}\cdot\:\text{sin}\left({r}_{2}\right)\cdot\:\left|{r}_{3}\cdot\:{X}_{best}^{t}-{X}_{i}^{t}\right|,\:\:\:\:{r}_{4}\le\:0.5\\\:{w\left(t\right)\cdot\:X}_{i}^{t}+{r}_{1}\cdot\:\text{cos}\left({r}_{2}\right)\cdot\:\left|{r}_{3}\cdot\:{X}_{best}^{t}-{X}_{i}^{t}\right|,\:\:\:\:{r}_{4}>0.5\end{array}\right.$$

Where *w*(t) is an iterative parameter descending from 1 to 0, and *t* is the current iteration. As mentioned, assigning larger inertia weight values promotes global search, while its lower numerical values facilitate polishing local solutions. This parameter can be calculated by

21 $$\:w\left(t\right)={w}_{end}+({w}_{init}-{w}_{end})\times\:\frac{t}{{t}_{max}}$$

Long et al.^70^ realized that conventional iteratively decreasing r_1_ in SCA provides a reasonable exploration at the initial stages of the iterations yet yields inferior convergence performance as iterations proceed, which results in good global exploration at the incipient phases of evolving iterations; however, it fails to circumvent the local pitfalls at later stages, deteriorating the local search mechanism. Furthermore, the nonlinear probing characteristics of the SCA algorithm do not allow for iteratively decreasing parameters to perform well on highly complex solution domains. To overcome these algorithmic drawbacks, they introduce a modified conversion parameter that is based on the Gaussian function formulated by

22 $$r_{1} \left( t \right) = \left( {a_{{init}} - a_{{end}} } \right) \times \exp \left[ { - \frac{{t^{2} }}{{(k \times t_{{\max }} )^{2} }}} \right] + a_{{end}}$$

Where *t* is the current iteration; *t*_*max*_ is the maximum number of iterations; and *k* is the modulation parameter that shapes the inclination of the Gaussian curve; and *a*_*init*_ and *a*_*end*_ are the initial and final points of the adjustable parameter “a”. After exhaustive numerical experiments, *a*_*init*,_ *a*_*end*,_ and model parameter *k* are respectively set to 0.2, 0.0, and 5.

Another novel approach proposed in SCA is the incorporation of dynamic inertia weight, rather than using iteratively modified parameters, as first introduced by Li et al.^72^. Like the previous case, they have observed that the success of inertia weight has been well established in earlier studies, most of which are associated with the Particle Swarm Optimization algorithm^73–76^. Inertia weight has been meticulously employed in these literature approaches to enhance the diversity of general solutions within the swarming particles. A suitable conclusion can be drawn from these past studies that an appropriate inertia weight value may significantly accelerate the convergence rate and enable the algorithm to explore undiscovered regions in the search domain. Concurring with the past researchers on this issue, it has been proven in these studies that larger inertia weight values increase the probability of arriving at unknown regions, empowering the exploration phase; on the contrary, smaller values facilitate exploitation, which is drawn from their numerical experiments established upon solving artificially generated optimization test problems. As a result of the low convergent characteristics of SCA, they proposed dynamically adjusting weight parameters, the explanatory formulation of which is given below.

23 $$w_{i} \left( t \right) = \left\{ {\begin{array}{*{20}l} {w_{{\min }} + \left( {w_{{\max }} - w_{{\min }} } \right)\frac{{f_{i} \left( t \right) - f_{{\min }} \left( t \right)}}{{f_{{mean}} \left( t \right) - f_{{\min }} \left( t \right)}},} & {f_{i} \left( t \right) \le f_{{mean}} \left( t \right)} \\ {w_{{\max }} ,} & {f_{i} \left( t \right) > f_{{mean}} \left( t \right)} \\ \end{array} } \right.$$

In Eq. (23), *w*_*min*_ and *w*_*max*_ are, respectively, user-defined numerical values of minimum and maximum weights, $\:{f}_{i}\left(t\right)\:$is the current fitness value of *i*^th^ population member; $\:{f}_{mean}\left(t\right)$is the mean fitness value of the population individuals; and $\:{f}_{min}\left(t\right)$ represents the population member with the minimum objective function value. Normalization between the lowest and mean objective function values among the population establishes a relationship between current fitness values and the optimal solution obtained so far, which is also found to be conducive to improving the algorithm’s convergence rate. Equation (23) states that if the current fitness rate of the solution is better than the average fitness rate, then the respective fitness rate tends to get lower values. This tendency in the weight value has a relatively minor influence on the objective function rate, so the newly generated solutions are carefully and tentatively developed within the neighborhood space. On the contrary, when the current fitness rate is higher than the mean fitness value of the population, the dynamic weight operator takes a more significant value to arrive at unexplored regions within the search space, creating disturbance to eliminate infeasible solutions when the worst fitness is encountered, enabling the algorithm to augment its ruling exploration mechanism. Therefore, it can be reliably concluded that employing a dynamic weight operator is beneficial for maintaining diversity within the population and facilitating global convergence, thereby significantly reducing the likelihood of being trapped in a local minimum within the solution domain.

This study proposes a novel and innovative solution generation scheme that integrates the two above-mentioned dynamic weight parameters defined in Eqs. (21) and (23) into the improved search equation of SCA given in Eq. (20). Furthermore, this study also aims to collectively introduce the Levy flight concept and chaotic numbers into the SCA to diversify the search space further and avoid premature convergence. The Levy flight search mechanism has been widely utilized in metaheuristic algorithms to obtain trial solutions with higher accuracy in past literature studies. Moreover, numerous successful attempts have been made to enhance the general optimization performance of SCA through the favorable properties of Levy flights^77–80^. The Levy flight search mechanism is widely accepted as one of the most influential random distribution methods based on the Gaussian distribution^81^. The implementation of Levy flight distribution to perform random walks requires two main characteristics to be explicitly identified. These determine the step length based on the ruling Levy distribution and the direction of the Levy motion towards the target location throughout the iterative process, which can be drawn from the uniform distribution^82^. The Levy flight distribution is incorporated into the proposed scheme to increase solution diversity within the trial population, thereby varying alternative answers in the design space and enhancing the exploration ability. Another valuable contribution integrated within the mutation scheme is taking advantage of chaotic numbers rather than uniformly distributed Gaussian random numbers. Chaotic maps have wide-ranging feasible applications in metaheuristic algorithm concepts, which rely on their conducive functional features such as ergodicity, regularity, and stochasticity^83^. The proclivities of sequential, random numbers are highly dependent on the initial conditions. Chaotic maps enticed algorithms to perform higher-speed, downhill searches compared to standard metaheuristic methods. Moreover, a wide range of various number sequences can be generated by just adjusting their preliminary conditions, which proves their versatility over contemporary alternatives. A large family of chaotic maps can be encountered for their successful integration into various metaheuristic algorithms in past literature approaches^84,85^. In this study, another novelty is proposed, namely the integration of chaotic numbers generated by the Ikeda map^86^ into the SCA algorithm. Despite its limited applications in literature studies regarding the chaos map-enhanced metaheuristic algorithms, the Ikeda map can provide promising solution outcomes by yielding robust and stable estimations and producing many sequences with higher unpredictability. The mathematical formulation of this 2D discrete-time dynamical chaotic map can be given as

24 $$\begin{aligned} x_{{i + 1}} = & 1 + 0.7\left( {x_{t} \cos \left( {\theta _{t} } \right) - y_{t} \sin \left( {\theta _{t} } \right)} \right) \\ y_{{i + 1}} = & 0.7\left( {x_{t} \sin \left( {\theta _{t} } \right) + y_{t} \cos \left( {\theta _{t} } \right)} \right) \\ \theta _{t} = & 0.4 - \frac{6}{{1 + x_{t}^{2} + y_{t}^{2} }}. \\ \end{aligned}$$

In standard SCA, uniformly distributed numbers are used to generate randomness and diversity in the population matrix, which comprises a set of trial solutions. These random parameters directly influence the tendencies of the position update mechanism of the ruling algorithm, playing a crucial role in balancing the exploration and exploitation phases. This study aims to enhance the overall effectiveness of SCA by utilizing these complementary yet contradictory search mechanisms, leveraging the merits of chaotic numbers generated from the Ikeda map. The solution update scheme expressed in pseudo-code form in Table 3 is proposed in this study, considering the combination of different types of adjustable inertia weight parameters, chaotic numbers, and the contribution of Levy flight-based random numbers.

**Table 3:**
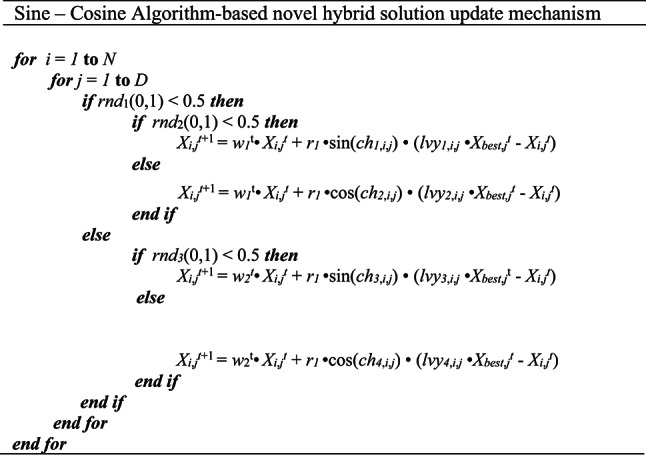
The proposed solution update mechanism.

**Fig. 1:**
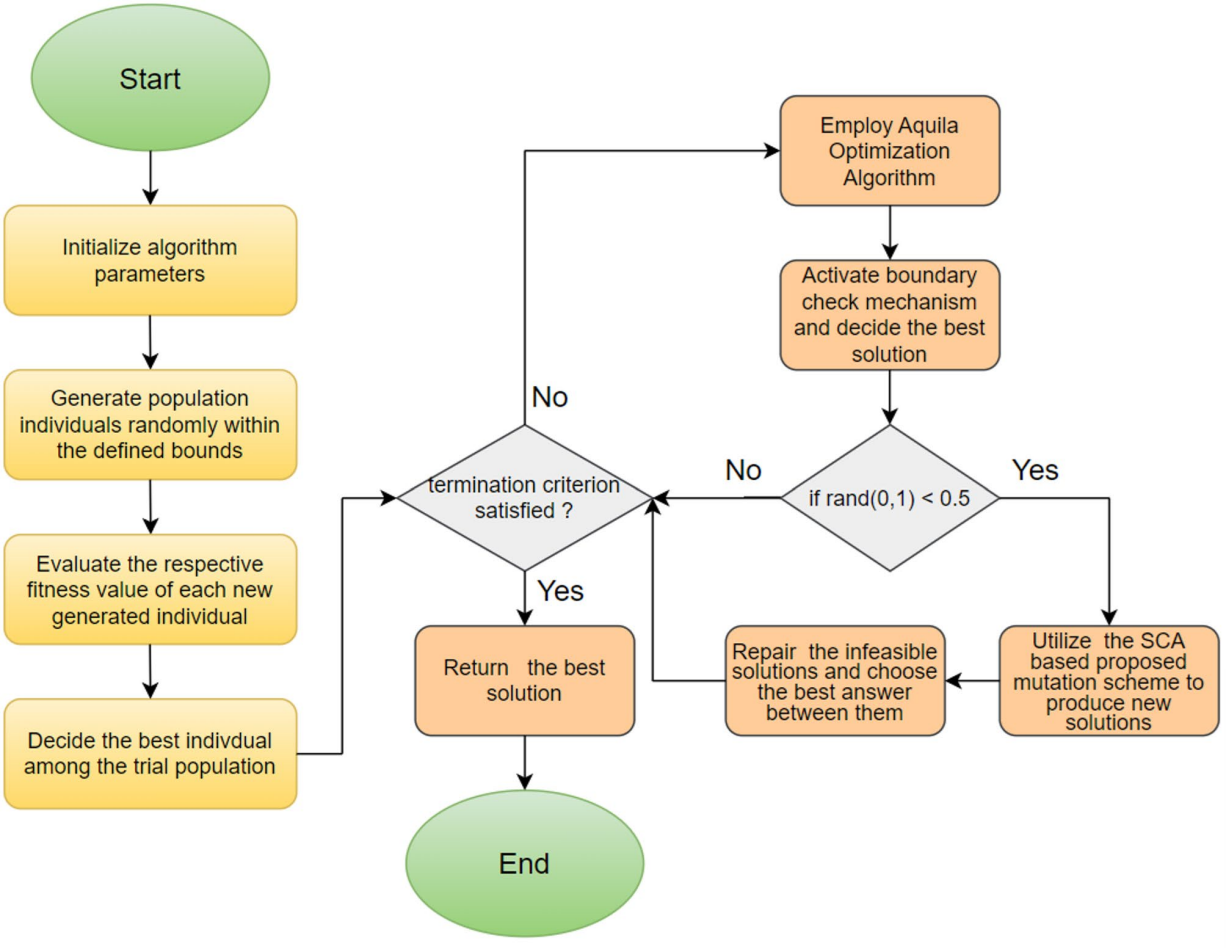
Schematic flowchart representation of the proposed AQSCA algorithm.

In Table 3, N is the population size; *D* is the dimensionality of the problem; *rnd*_*1,2,3*_(0,1) are different random numbers drawn from a Gaussian distribution in the range [0,1] used to decide on which manipulation is performed in the current iteration; *w*_*1*_ and *w*_*2*_ are inertia weight parameters, respectively computed by Eqs. (21) and (23); r_1_ is the modified conversion parameter calculated by Eq. (22) employed to transition between exploration and exploitation phases; ch_1,2,3,4_ are chaotic random numbers extracted from the Ikeda chaotic map; *lvy*_1,2,3,4_ are different random numbers produced from the Levy flight distribution; and *X*_*best*_ is the current best solution. In the proposed algorithm presented in Table 3, Levy numbers are utilized to roam more freely towards the current best solution (*X*_*best*_), thereby intensifying the search direction in previously explored promising regions. It is also observed from the numerical experiments that benefiting from the chaotic number sequences generated from the Ikeda map considerably improves diversity within the population, thanks to the significant augmentation in the exploration mechanism resulting from the integration of chaotic numbers. Experimental outcomes of the test problems, which will also be discussed in the next section, also reveal that the probability of visitation to fertile neighboring spaces is much more likely to occur if random numbers from the Levy flight distribution are utilized rather than uniform random numbers. This proposed search scheme is integrated into the standard Aquila optimizer to compensate for its algorithmic deficiencies. Previous experiences also reveal that this proposed SCA-based solution update scheme is a versatile, practical, and viable alternative that can be successfully implemented in most methods to improve their general optimization performance. Table 4 provides a brief overview of the basic steps of the proposed hybrid algorithm. Figure 1 schematizes the algorithmic steps of the proposed AQSCA hybrid optimization procedure.

### The time complexity of the proposed AQSCA

The time complexity analysis of the proposed method will be presented in this section of the research study. It is known that the complexity of the standard AQUILA optimizer is $\:O(T\cdot\:N\cdot\:D)$ where T is the maximum number of iterations to terminate the algorithm run, N is the population size, and D is the problem dimension. Before the iterative process proceeds, random generation of the population individuals has the corresponding complexity of $\:O(N\cdot\:D)$, when the proposed method is employed to the base AQUILA optimizer with a probability of 0.5, it is only utilized in half of the iterations on average, and its respective time complexity becomes $\:O(0.5\cdot\:T\cdot\:N\cdot\:D)$. Sorting all population individuals based on their corresponding fitness values to determine the best member requires time complexity $\:O(T\cdot\:N\cdot\:\text{l}\text{o}\text{g}(N\left)\right)$. Then, the overall time complexity becomes $\:O(T\cdot\:N\cdot\:D)$+$\:O(0.5\cdot\:T\cdot\:N\cdot\:D)$+ $\:O(T\cdot\:N\cdot\:\text{l}\text{o}\text{g}(N\left)\right)$. Since $\:O(0.5\cdot\:T\cdot\:N\cdot\:D)$is still on the same order and log(N) is significantly smaller than N, the total complexity of the hybrid algorithm remains $\:O(T\cdot\:N\cdot\:D)$, which is the same as the original AQUILA algorithm.

**Table 4:**
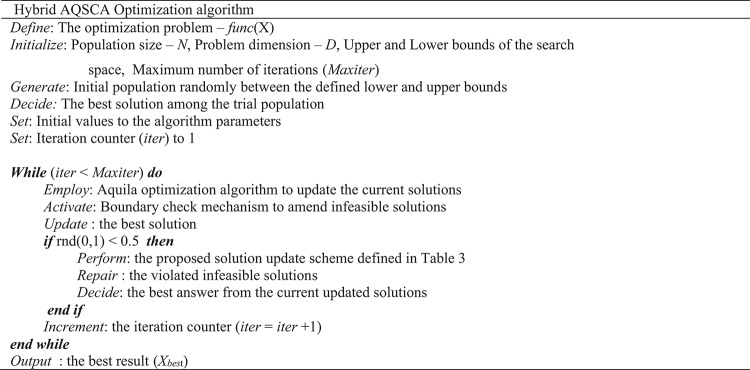
The proposed hybrid AQSCA optimization algorithm.

### Numerical experiments over the proposed hybrid

#### Evaluation of global optimization problems

This section evaluates the optimization efficiency of the proposed hybrid algorithm over multidimensional and hyperdimensional optimization problems. It compares the optimum results found by some of the well-reputed literature metaheuristic optimization methods, such as the original AQUILA algorithm, Multi-Verse Optimization Algorithm (MVO)^87^, Spotted Hyena Optimization (SPOTTED)^88^, SCA, Jaya Optimization (JAYA)^89^, Equilibrium Optimizer (EQUIL)^90^, and Moth-Flame Optimization (MOTH)^91^. A widely employed procedure in the existing literature utilizes optimization benchmark functions with various functional characteristics to assess the predictive performance of stochastic metaheuristic algorithms. Using these functions provides reliable insights into the general tendencies of the compared algorithm; therefore, researchers have developed numerous artificially generated multidimensional optimization benchmark functions with different features. In this section, a total of 100 test functions is introduced for performance evaluation. Tables 5 and 6 report the function titles of the utilized multimodal and unimodal optimization benchmark problems and their corresponding allowable search ranges. All numerical simulations were performed in the Windows 10 Professional Operating System environment using an Intel processor 2.7 GHz 8.0 GB RAM, and compared algorithms, including the proposed hybrid method, have been developed in a MATLAB environment. The population size of each algorithm is fixed at *N* = 20, and the maximum number of iterations is defined as the termination criterion, with Maxiter = 100. Numerical experiments have been performed for 30D and 500D test problems to validate the estimation accuracy of the compared algorithms for high- and hyper-dimensional optimization benchmark problems. Table 7 reports the parameter settings of the metaheuristic algorithms used in the performance comparison.

**Fig. 2:**
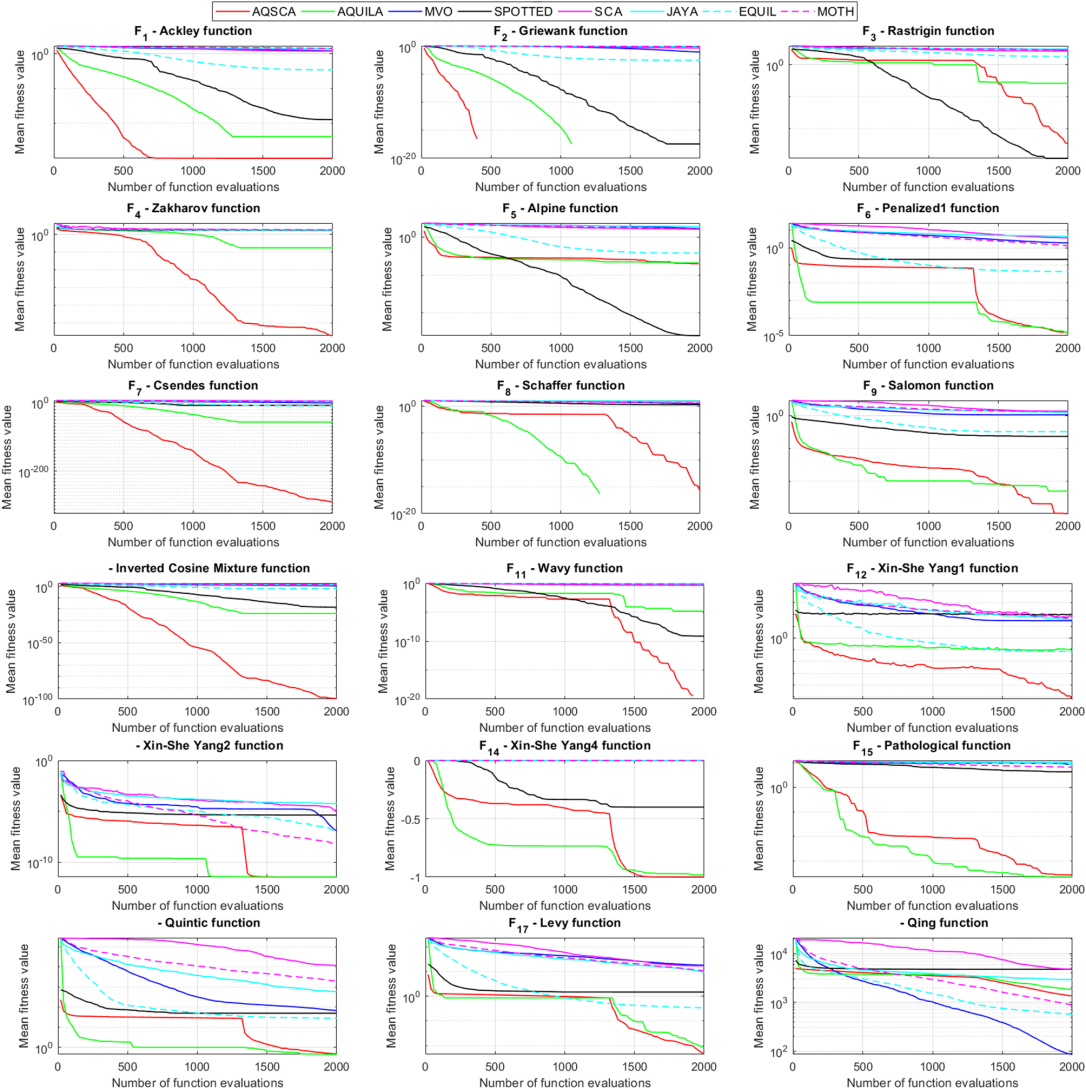
Evolution histories of the compared algorithm for 30D multimodal test functions.

**Fig. 3:**
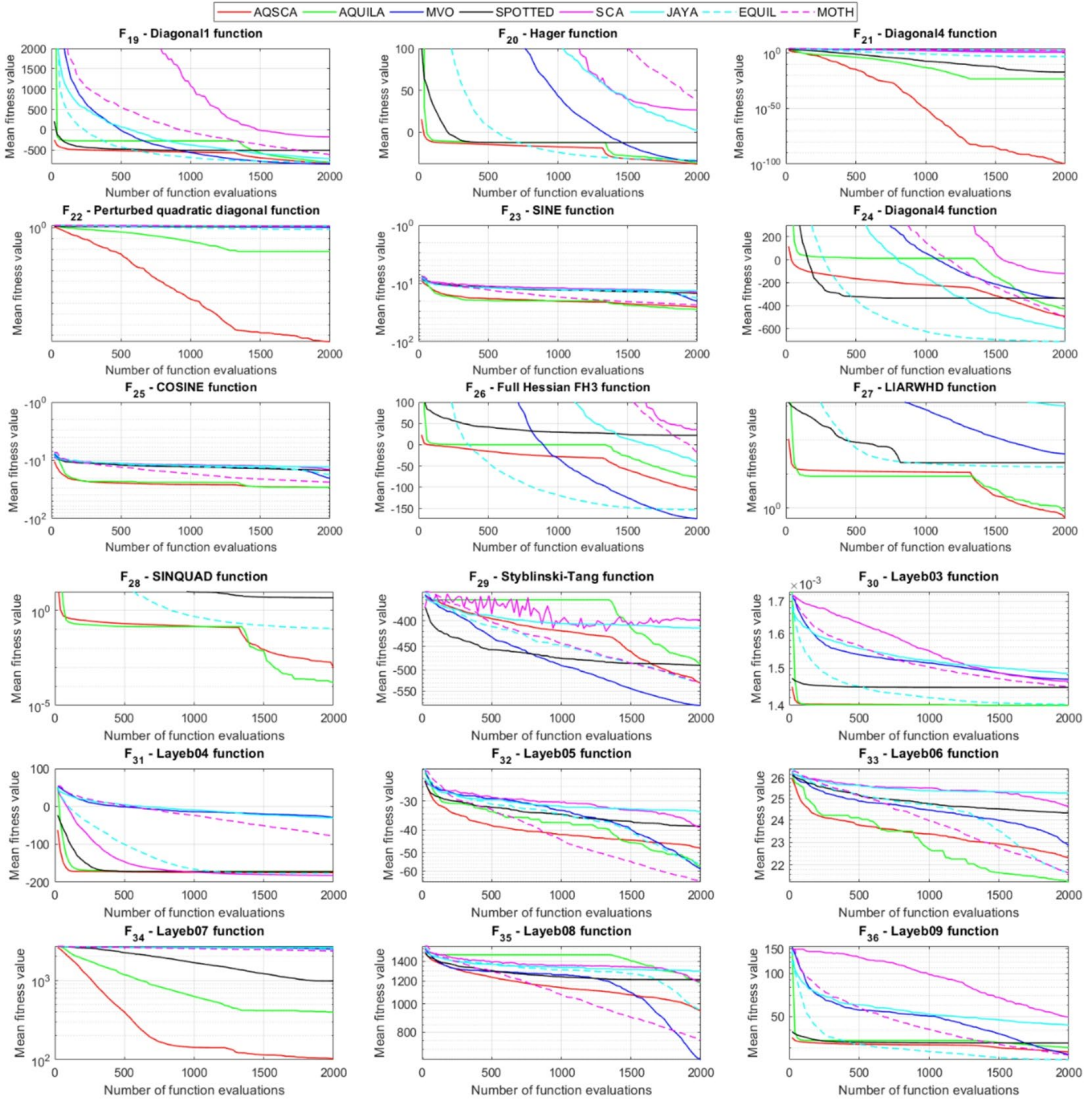
Convergence performance of the algorithms with respect to increasing iterations for 30D multimodal test functions from F_19_ – Diagonal to F_36_ – Layeb09.

**Fig. 4:**
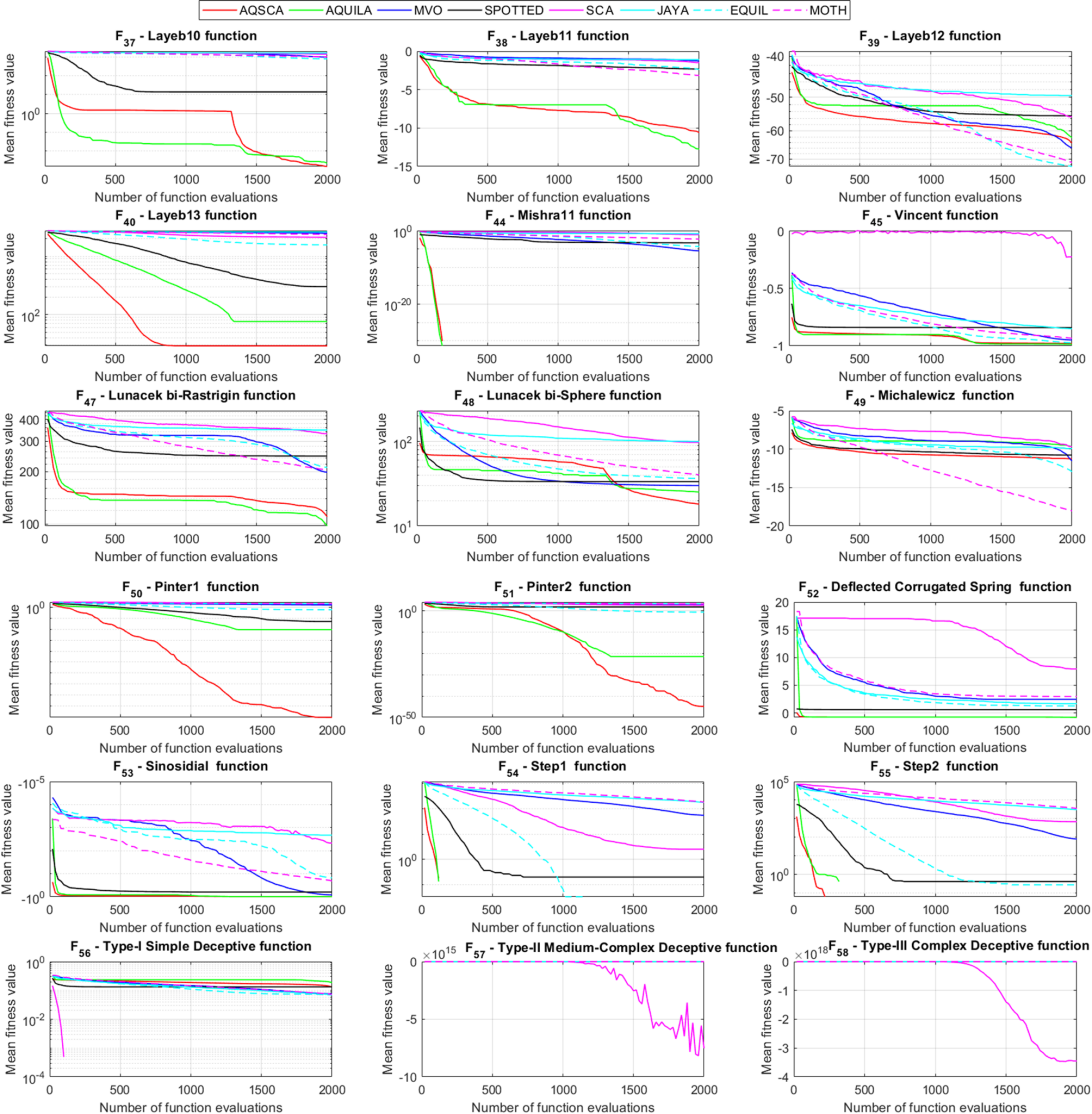
Iterative declines in objective function values with increasing number of function evaluations for 30D multimodal benchmark functions between F_37_-Layeb10 and F_58_ – Type-III complex Deceptive.

**Fig. 5:**
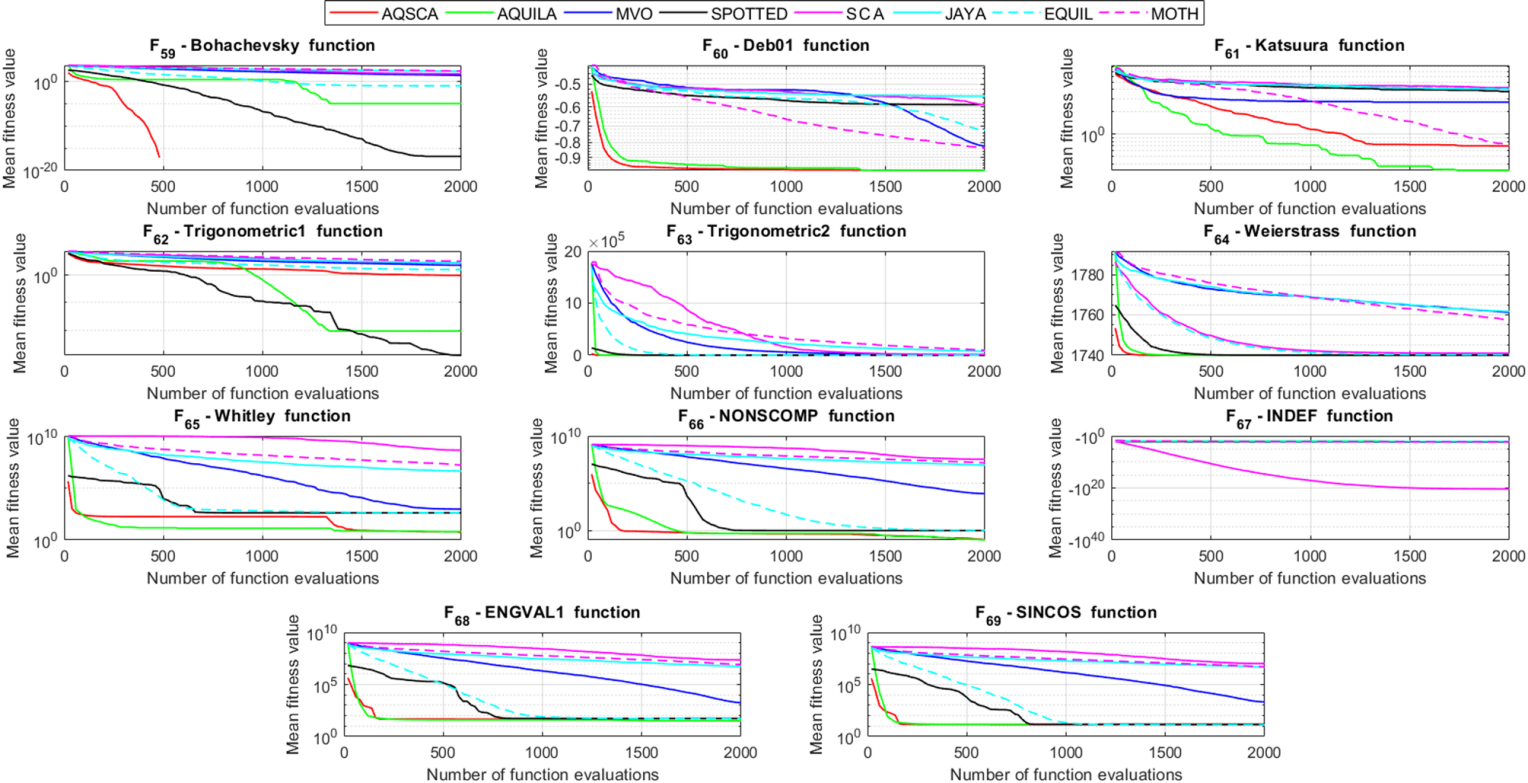
Optimal convergence rates for 30D multimodal test functions between F_59_ – Bohachevsky and F_69_ – SINCOS.

**Fig. 6:**
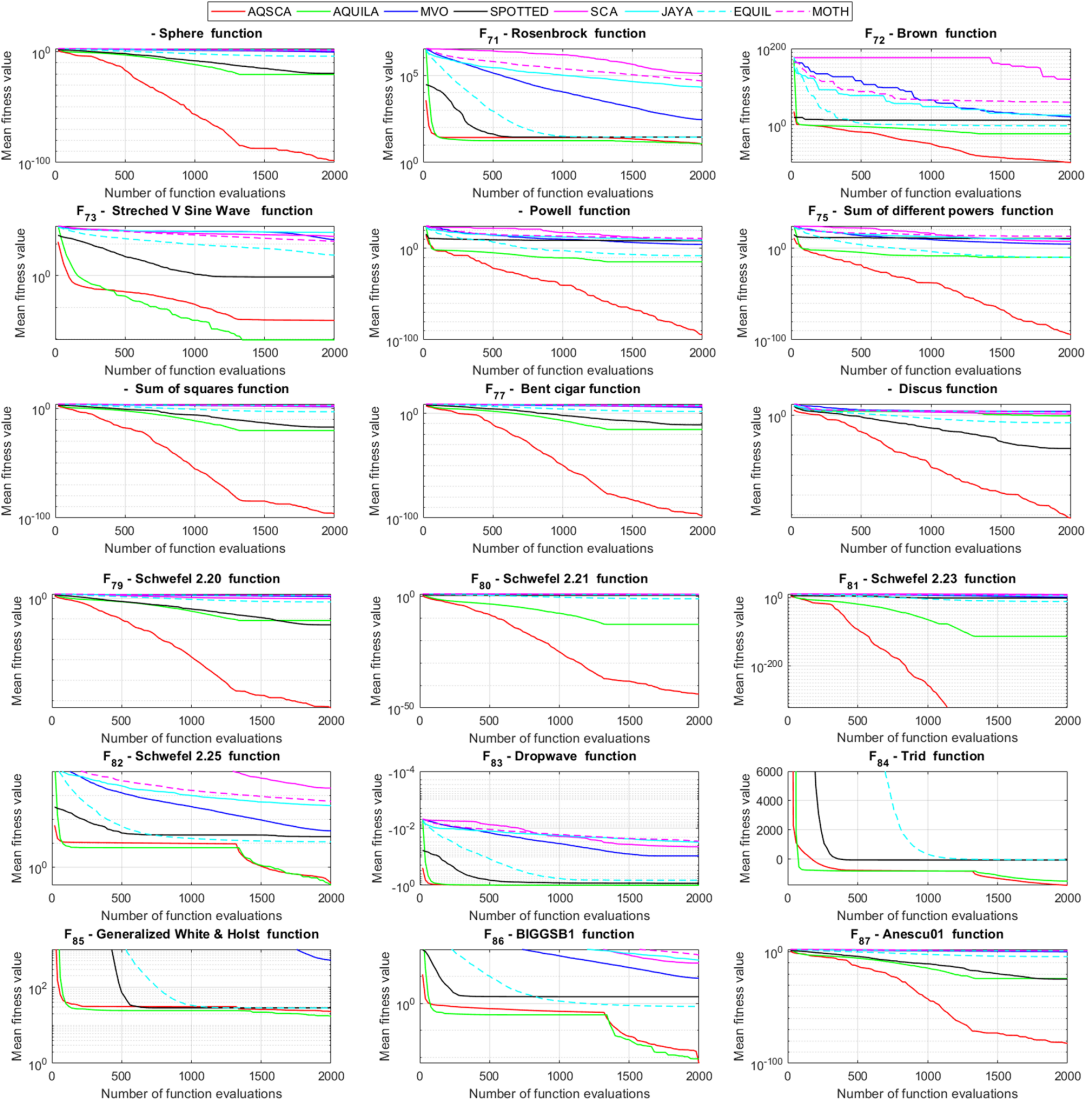
Evolution of the fitness values with increasing iterations for 30D unimodal test functions.

**Fig. 7:**
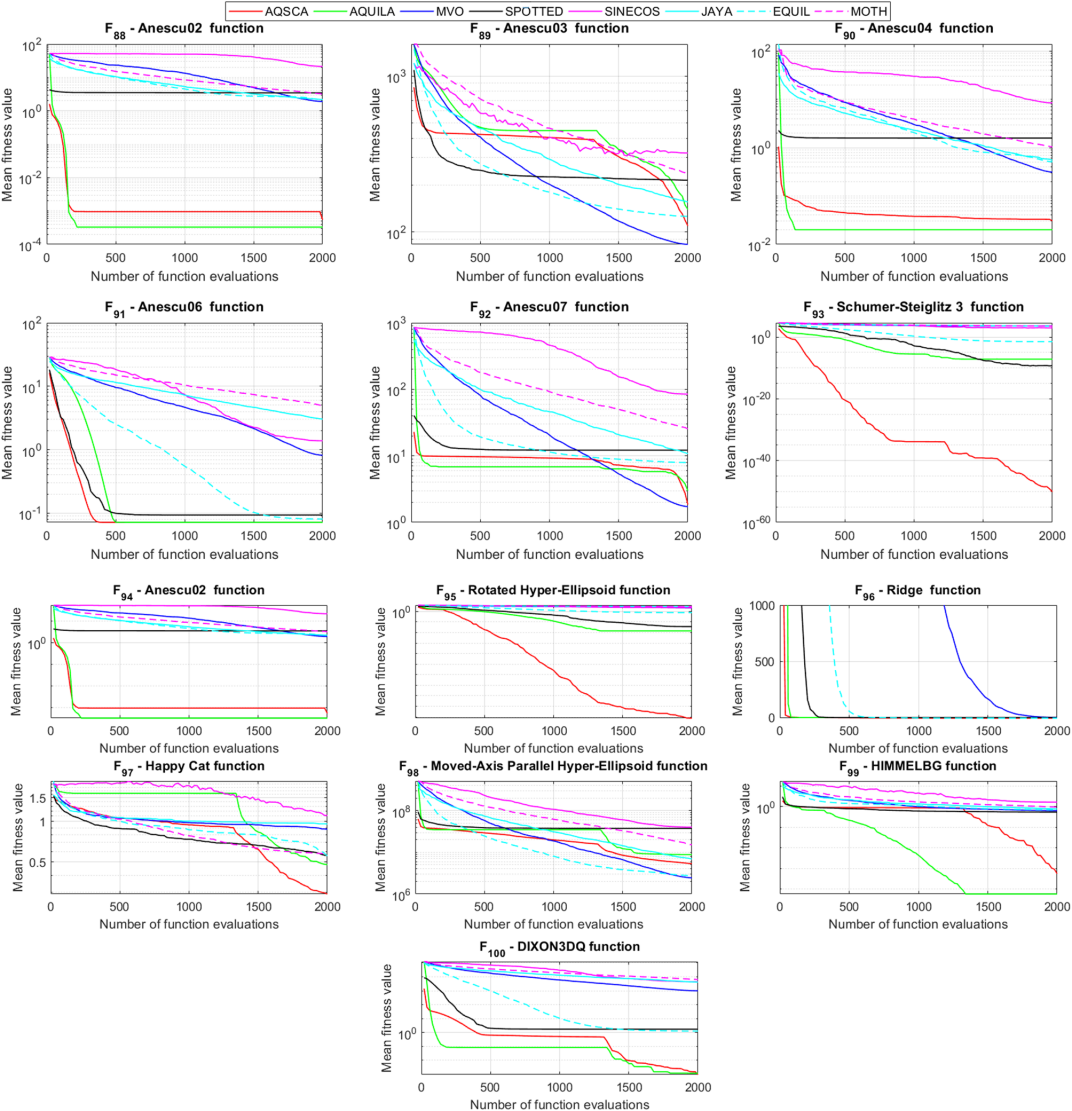
Iterative declines in fitness values with increasing number of function evaluations for 30D test functions.

To validate the performance of the proposed AQSCA, sixty-nine 30D multimodal benchmark problems were solved, and the respective statistical results retrieved from 50 independent algorithm runs for each optimizer are reported in Table 8. It is seen that AQSCA obtains the globally optimal solutions of F_1_, F_2_, F_3_, F_7_, F_8_, F_11_, F_14_, F_15_, F_40_, F_44_, F_54_, F_55_, F_59_, and F_60_ test functions at least once within successive iterations. Furthermore, very close predictions to the known optimum solutions of F_4_, F_5_, F_9_, F_10_, F_12_, F_21_, F_22_, F_50_, F_51_, and F_62_ test problems. In terms of mean results, it is observed that the AQSCA algorithm dominates the remaining methods in thirty-two out of sixty-nine test functions and outperforms the compared optimizers. JAYA is the worst performer among them, considering the mean results. The reason behind considering mean results rather than other statistical measures is to evaluate the robustness and consistency of the predictions. Considering the best performance evaluation results gives readers insights into the degree of convergence rates of the compared algorithms. It is also interesting to note that AQSCA surpasses the original AQUILA algorithm in forty-eight out of sixty-nine test functions, considering the mean results, which also demonstrates the indisputable achievement of integrating different variants of SCA into the standard AQUILA algorithm, resulting in enhanced global exploration mechanisms to much higher levels. Figures 2, 3 and 4, and Fig. 5 illustrate the convergence rates of the compared algorithms for 30D multimodal test problems. First and foremost, it can be stated that the AQSCA algorithm achieves the best convergence rate compared to the remaining optimization algorithms. Different convergence behaviors are observed for different test functions for the proposed algorithm. Prevalent convergence tendency for 30D F_3_, F_5_, F_6_, F_8_, F_9_, F_11_, F_12_, F_13_, F_14_, F_15_, F_16_, F_17_, F_19_, F_20_, F_26_, F_27_, F_28_, F_31_, F_37_, F_45_, F_47_, F_48_, F_65_, F_66_, and F_68_ test functions is that rapid declines at the early phases of the iterations, following with entering a stagnation zone with a little progress in objective function values, and finally abrupt convergence to the optimal solution of the problem. This convergence behavior can be explained by the balanced distribution of exploration and exploitation mechanisms achieved through the hybridization of two complementary algorithms. The exploration phase dominates in the early and middle phases, emphasizing roaming around the defined search domain too much to improve the fitness values. Then, the exploitation mechanism plays a decisive role in intensifying the fertile regions visited during the exploration phase, making a favorable contribution to the governing search characteristics of the proposed algorithm. Table 9 presents the Wilcoxon signed-rank test results, chosen to evaluate the statistical performance of the AQSCA algorithm for 30D multidimensional test functions. This is a pairwise comparison between two selected algorithms for analyzing the significant differences between predictive results. A comparative analysis between them enables readers to comprehend that the significance of the collected solutions does not occur by chance and that mathematical logic lies beyond the degree of predictive significance. In the numerical experiments, a *p*-value < 0.05 indicates that two datasets comprising the estimated fitness values are significant. Table 9 shows that most of the benchmark functions reject the null hypothesis of equal medians at the default 5% significance level. Predictions indicate a significant difference between the algorithms considered. Table 10 presents the reported prediction results for the 30D unimodal test problems using the compared algorithms. AQSCA emerges as the best performer in 21 out of 30 test problems, based on mean results, and declares dominance over the remaining algorithms. AQSCA obtains the best-known answer to the optimization test problems of F_81_, F_83_, and F_96_ functions at least once in successive algorithm runs. Furthermore, accurate and robust estimations are also observed for F_70_, F_72_, F_74_, F_75_, F_76_, F_77_, F_78_, F_79_, F_80_, F_87_, F_93_, F_94_, and F_95_. Standard AQUILA is the second-best performer after the proposed method for the 30D unimodal test function, while JAYA yields the worst predictions between them, considering the mean results. Figures 6 and 7 visually compare the convergence rates of the contestant algorithms, including the proposed AQSCA for 30D unimodal test problems. It is observed that AQSCA consistently employs the fast-convergent algorithm in most cases, reaching its optimum point more quickly than other methods, thereby demonstrating its ability to exploit the SCA-based mutation scheme. Table 11 reports the Wilcoxon signed-rank test results obtained for 30D unimodal problems. Statistical significance of the AQSCA is evident as there is a considerable difference in fitness values between the algorithms considered in comparison, which are also corroborated by their respective *p*-values.

Solving optimization problems with design variables reaching up to a million dimensions is challenging in terms of computational efficiency and heuristic-based search in huge solution spaces. One common benchmark topic should be evaluating the general performance of metaheuristic algorithms over hyperdimensional optimization test problems. Suppose the employed algorithm provides promising capabilities to overcome the characteristics and drawbacks of highly high-dimensional problems. This demonstrates that the overwhelmingly increased search spaces do not compromise the quality of general solutions, thanks to the balanced exploration and exploitation mechanisms. Table 12 provides a comparative analysis of the prediction performances of the given metaheuristic algorithm over 500D hyperdimensional multimodal benchmark functions. Considering hyperdimensional problems in evaluating the relative performances of the algorithms offers valuable insights into which optimizer is severely influenced by the disadvantageous condition known as “the curse of dimensionality.” It allows us to understand how the proposed hybrid optimizer performs when solving hyperdimensional problems. It is seen that increased problem dimensionality does not negatively influence the prediction performance of the AQSCA hybrid as it acquires the global optimum points of F_1_, F_2_, F_3_, F_7_, F_8_, F_11_, F_14_, F_15_, F_40_, F_44_, F_54_, F_55_, F_59_, and F_64_ at least one time and show that it can conquer the adverse effects of the higher dimensionality of the employed problems and prove that probing ability algorithm is not deteriorated by increased problem dimensionalities Furthermore, accurate estimations performed by the proposed AQSCA is evident for 500D test problems of F_10_, F_12_, F_13_, F_14_, F_21_, F_22_, F_25_, F_34_, F_45_, F_46_, F_50_, and F_52_ as it reaches close predictions to the known best solutions for these given test problems. In terms of mean results, AQSCA outperforms the remaining algorithms in 53 out of 69 test functions, demonstrating that the enhancement in the probing ability of the hybrid algorithm through the SCA-induced mutation scheme avoids being trapped in exponentially increasing local pitfalls over the solution domain, resulting from the hyper-dimensionality of the problem. Table 13 presents the optimal solutions obtained for the comparison algorithms on 500D unimodal benchmark problems. The exploitative searchability of the proposed AQSCA is verified by the predictions obtained for higher-dimensional unimodal test functions, which surpass those of the other methods in Table 13 in most test cases, considering the mean results. It is also interesting to see that while AQSCA is converging to its optimum point, the other methods expect AQUILA to be far away from its optimum solutions, significantly lacking in balancing the exploration and exploitation mechanisms for these cases. This behavior is evident for almost all benchmark problems in Table 13. Wilcoxon signed-rank test results at a 0.05 significance level for 500D multimodal and unimodal test functions are reported in Tables 14 and 15, respectively. The statistical significance of the proposed method is approved when respective *p*-values are investigated, which are obtained after exhaustive pairwise comparisons between the proposed AQSCA and the other matched optimizer, as shown in Tables 14 and 15.

**Table 5:** Multimodal benchmark functions used for performance evaluations.

Function name	Dimension (D)	Range
F_1_ – Ackley	30, 500	[−32,32]^D^
F_2_ – Griewank	30, 500	[−600,600]^D^
F_3_ – Rastrigin	30, 500	[−5.12,5.12]^D^
F_4_ – Zakharov	30, 500	[−5.0,10.0]^D^
F_5_ – Alpine	30, 500	[0,10]^D^
F_6_ – Penalized1	30, 500	[−50.0,50.0]^D^
F_7_ – Csendes	30, 500	[−5.0,5.0]^D^
F_8_ – Schaffer	30, 500	[−100.0,100.0]
F_9_ – Salomon	30, 500	[−50.0,50.0]^D^
F_10_ – Inverted cosine mixture	30, 500	[−10.0,10.0]^D^
F_11_ – Wavy	30, 500	[−3.14,3.14] ^D^
F_12_ – Xin She Yang1	30, 500	[−5.0,5.0]^D^
F_13_ – Xin She Yang2	30, 500	[−6.28,6.28]^D^
F_14_ – Xin She Yang4	30, 500	[−10.0,10.0]^D^
F_15_ – Pathological	30, 500	[−10.0,10.0]^D^
F_16_ – Quintic	30, 500	[−10.0,10.0]^D^
F_17_ – Levy	30, 500	[−10.0,10.0]^D^
F_18_ – Qing	30, 500	[−500.0,500.0]^D^
F_19_ – Diagonal1	30, 500	[−10.0,10.0]^D^
F_20_ – Hager	30, 500	[−10.0,10.0]^D^
F_21_ – Diagonal4	30, 500	[−10.0,10.0]^D^
F_22_ – Perturbed Quadratic Diagonal	30, 500	[−10.0,10.0]^D^
F_23_ – SINE	30, 500	[−10.0,10.0]^D^
F_24_ – Diagonal9	30, 500	[−10.0,10.0]^D^
F_25_ – COSINE	30, 500	[−10.0,10.0]^D^
F_26_ – Full Hessian FH3	30, 500	[−10.0,10.0]^D^
F_27_ – LIARWHD	30, 500	[−10.0,10.0]^D^
F_28_ – SINQUAD	30, 500	[−10.0,10.0]^D^
F_29_ – Styblinski –Tang	30, 500	[−2.0,2.0]^D^
F_30_ – Layeb03	30, 500	[−10.0,10.0]^D^
F_31_ – Layeb04	30, 500	[−10.0,10.0]^D^
F_32_ – Layeb05	30, 500	[−10.0,10.0]^D^
F_33_ – Layeb06	30, 500	[−10.0,10.0]^D^
F_34_ – Layeb07	30, 500	[−10.0,10.0]^D^
F_35_ – Layeb08	30, 500	[−10.0,10.0]^D^
F_36_ – Layeb09	30, 500	[−10.0,10.0]^D^
F_37_ – Layeb10	30, 500	[−10.0,10.0]^D^
F_38_ – Layeb11	30, 500	[−10.0,10.0]^D^
F_39_ – Layeb12	30, 500	[−5.0,5.0]^D^
F_40_ – Layeb13	30, 500	[−10.0,10.0]^D^
F_41_ – Mishra1	30, 500	[0.0,1.0]^D^
F_42_ – Mishra2	30, 500	[0.0,1.0]^D^
F_43_ – Mishra7	30, 500	[−10.0,10.0]^D^
F_44_ – Mishra11	30, 500	[−10.0,10.0]^D^
F_45_ – Vincent	30, 500	[ 0.0,10.0]^D^
F_46_ – F2	30, 500	[ 0.0,1.0]^D^
F_47_ – Lunacek’s bi Rastigin	30, 500	[−5.12,5.12]^D^
F_48_ - Lunacek’s bi Sphere	30, 500	[−10.0,10.0]^D^
F_49_ – Michalewicz	30, 500	[0.0,3.14]^D^
F_50_ – Pinter1	30, 500	[−10.0,10.0]^D^
F_51_ – Pinter2	30, 500	[−10.0,10.0]^D^
F_52_ – Deflected Corrugated Spring	30, 500	[ 0.0,10.0]^D^
F_53_ – Sinosidial	30, 500	[0.0,3.14]^D^
F_54_ – Step1	30, 500	[−100.0,100.0]^D^
F_55_ – Step2	30, 500	[−100.0,100.0]^D^
F_56_ – Type – I Simple Deceptive	30, 500	[0.0,1.0]^D^
F_57_– Type – II Medium-Complex Deceptive	30, 500	[0.0,1.0]^D^
F_58_ – Type – III Complex Deceptive	30, 500	[0.0,1.0]^D^
F_59_ – Bohachevsky	30, 500	[−15.0,15.0]^D^
F_60_ – Deb01	30, 500	[−1.0,1.0]^D^
F_61_ – Katsuura	30, 500	[0.0,100.0]^D^
F_62_ – Trigonometric1	30, 500	[0.0,3.14]^D^
F_63_ – Trigonometric2	30, 500	[−500.0,500.0]^D^
F_64_ – Weierstrass	30, 500	[−5.0,5.0]^D^
F_65_ – Whitley	30, 500	[−10.0,10.0]^D^
F_66_ – NONSCOMP	30, 500	[−100.0,100.0]^D^
F_67_ – INDEF	30, 500	[−10.0,10.0]^D^
F_68_ – ENGVAL1	30, 500	[−100.0,100.0]^D^
F_69_ – SINCOS	30, 500	[−100.0,100.0]^D^

**Table 6:** Unimodal optimization benchmark functions for evaluating the prediction performances of the algorithms.

Function name	Dimension	Range
F_70_ – Sphere	30, 500	[−5.12,5.12]^D^
F_71_ – Rosenbrock	30, 500	[−30.0,30.0]^D^
F_72_ – Brown	30, 500	[−1.0,4.0]^D^
F_73_ – Streched V Sine Wave	30, 500	[−10.0,10.0]^D^
F_74_ – Powell	30, 500	[ 0.0,10.0]^D^
F_75_ – Sum of Different Powers	30, 500	[ -1.0,1.0]^D^
F_76_ – Sum of Squares	30, 500	[−10.0,10.0]^D^
F_77_ – Bent cigar	30, 500	[ -5.0,5.0]^D^
F_78_ – Discus	30, 500	[−100.0,100.0]^D^
F_79_ – Schwefel 2.20	30, 500	[−100.0,100.0]^D^
F_80_ – Schwefel 2.21	30, 500	[−100.0,100.0]^D^
F_81_ – Schwefel 2.23	30, 500	[−10.0,10.0]^D^
F_82_ – Schwefel 2.25	30, 500	[ 0.0,10.0]^D^
F_83_ – Dropwave	30, 500	[−5.12,5.12]^D^
F_84_ – Trid	30, 500	[D^2^,D^2^]^D^
F_85_ – Generalized White & Holst	30, 500	[−10.0,10.0]^D^
F_86_ – BIGGSB1	30, 500	[−10,10]^D^
F_87_ – Anescu01	30, 500	[−2.0,2.0]^D^
F_88_ – Anescu02	30, 500	[1.39,4.0]^D^
F_89_ – Anescu03	30, 500	[−4.0,1.39]^D^
F_90_ – Anescu04	30, 500	[0.001,2.0]^D^
F_91_ – Anescu06	30, 500	[0.001,2.0]^D^
F_92_ – Anescu07	30, 500	[−2.0,2.0]^D^
F_93_ – Schumer-Steiglitz 3	30, 500	[−100.0,100.0]^D^
F_94_ – Schumer-Steiglitz 2	30, 500	[−100.0,100.0]^D^
F_95_ – Rotated Hyper-Ellipsoid	30, 500	[−100.0,100.0]^D^
F_96_ – Ridge	30, 500	[−5.0,5.0]^D^
F_97_ – HappyCat	30, 500	[−2.0,2.0]^D^
F_98_ – Moved-Axis Parallel Hyperellipsoid	30, 500	[−100.0,100.0]^D^
F_99_ – HIMMELBG	30, 500	[−10.0,10.0]^D^
F_100_ – DIXON3DQ	30, 500	[−100.0,100.0]^D^

**Table 7:** Parameter settings of the compared algorithms.

Algorithm	Model parameters
AQSCA	Model parameter – *U* = 0.00565 Model parameter – *ω* = 0 0.005
AQUILA	Model parameter – *U* = 0.00565 Model parameter – *ω* = 0 0.005
MVO	Upper limit of Wormhole Existence Probability (*WEP*_*max*_ = 1.2) Lower limit of Wormhole Existence Probability (*WEP*_*min*_ = 0.2)
SPOTTED	No tunable parameter involved
SINECOS	No tunable parameter involved
JAYA	No tunable parameter involved
EQUIL	No tunable parameter involved
MOTH	No tunable parameter involved

**Table 8:** Prediction performances of the compared algorithms over 30D multimodal test functions.

		AQSCA	AQUILA	MVO	SPOTTED	SINECOS	JAYA	EQUIL	MOTH
F_1_	Min	8.88E-16	8.88E-16	1.85E + 00	8.88E-16	7.41E-02	3.98E + 00	1.58E-03	4.63E + 00
	Mean	8.88E-16	1.21E-12	2.70E + 00	3.34E-10	2.41E + 00	5.35E + 00	4.88E-03	5.97E + 00
	Std.dev	0.00E + 00	6.62E-12	4.66E-01	8.07E-10	1.75E + 00	4.97E-01	1.97E-03	5.80E-01
	Max	8.88E-16	3.62E-11	3.56E + 00	3.52E-09	6.43E + 00	6.49E + 00	1.05E-02	7.16E + 00
F_2_	Min	0.00E + 00	0.00E + 00	4.44E-02	0.00E + 00	5.60E-03	6.32E-01	7.20E-07	5.36E-01
	Mean	0.00E + 00	0.00E + 00	9.57E-02	3.70E-18	4.71E-01	8.48E-01	3.17E-03	8.03E-01
	Std.dev	0.00E + 00	0.00E + 00	3.32E-02	2.02e-17	3.04E-01	7.91E-02	7.42E-03	1.20E-01
	Max	0.00E + 00	0.00E + 00	1.68E-01	1.11E-16	9.33E-01	9.55E-01	2.72E-02	9.79E-01
F_3_	Min	0.00E + 00	0.00E + 00	1.56E + 02	0.00E + 00	2.88E + 01	2.64E + 02	2.57E + 00	1.64E + 02
	Mean	3.89E-13	1.25E-03	2.30E + 02	1.89E-15	1.49E + 02	3.12E + 02	2.10E + 01	2.33E + 02
	Std.dev	3.34E-12	6.87E-03	4.34E + 01	1.03E-14	8.25E + 01	3.34E + 01	1.35E + 01	3.61E + 01
	Max	3.30E-11	3.76E-02	3.18E + 02	5.68E-14	3.74E + 02	4.04E + 02	7.45E + 01	3.34E + 02
F_4_	Min	1.95E-96	3.97E-29	1.52E + 02	3.80E + 01	1.58E + 02	2.41E + 02	5.01E + 01	1.66E + 02
	Mean	1.03E-57	2.79E-08	2.66E + 02	2.16E + 02	2.85E + 02	4.04E + 02	1.24E + 02	4.21E + 02
	Std.dev	7.33E-57	1.05E-07	8.90E + 01	1.64E + 02	6.75E + 01	6.43E + 01	4.93E + 01	9.69E + 01
	Max	5.94E-56	5.38E-07	5.04E + 02	5.91E + 02	4.29E + 02	5.04E + 02	2.66E + 02	5.77E + 02
F_5_	Min	1.97E-73	7.17E-19	6.20E + 00	4.83E-22	1.18E-01	1.89E + 01	4.11E-03	6.50E + 00
	Mean	3.08E-04	3.13E-04	1.29E + 01	1.08E-13	1.07E + 01	2.46E + 01	7.78E-03	1.17E + 01
	Std.dev	1.01E-03	6.57E-04	3.17E + 00	3.11E-13	9.06E + 00	3.73E + 00	2.57E-03	2.47E + 00
	Max	5.69E-03	2.23E-03	2.25E + 01	1.53E-12	2.64E + 01	3.30E + 01	1.47E-02	1.63E + 01
F_6_	Min	4.29E-08	8.21E-09	2.40E-01	5.29E-02	7.77E-01	2.88E + 00	1.78E-02	4.41E-01
	Mean	1.53E-05	1.49E-05	1.90E + 00	2.18E-01	3.70E + 00	4.40E + 00	4.38E-02	1.29E + 00
	Std.dev	3.12E-05	2.92E-05	8.47E-01	1.06E-01	2.95E + 00	8.42E-01	3.18E-02	5.34E-01
	Max	2.61E-04	1.27E-04	3.56E + 00	5.89E-01	1.31E + 01	6.09E + 00	1.89E-01	3.02E + 00
F_7_	Min	0.00E + 00	8.18E-109	1.17E-02	7.00E-81	5.67R-01	1.05E + 03	5.41E-14	1.08E + 03
	Mean	3.21E-291	4.99E-57	1.97E-01	1.43E-08	1.48E + 05	6.34E + 03	5.12E-10	1.58E + 04
	Std.dev	0.00E + 00	2.19E-56	3.16E-01	4.39E-08	1.94E + 05	5.36E + 03	1.34E-09	1.95e + 04
	Max	2.56E-289	1.20E-55	1.43E + 00	2.01E-07	7.69E + 05	2.24E + 04	5.65E-09	1.06e + 05
F_8_	Min	0.00E + 00	0.00E + 00	1.98E + 00	0.00E + 00	5.39E + 00	6.22E + 01	1.11E + 00	2.37E + 00
	Mean	1.68E-16	0.00E + 00	2.75E + 00	1.78E + 00	6.73E + 00	8.02E + 00	3.66E + 00	3.41E + 00
	Std.dev	1.68E-15	0.00E + 00	4.35E-01	3.02E + 00	6.65E-01	6.18E-01	1.00E + 00	6.14E-01
	Max	1.68E-14	0.00E + 00	3.82E + 00	7.74E + 00	7.96E + 00	9.53E + 00	5.49E + 00	4.73E + 00
F_9_	Min	3.62E-72	3.45E-19	6.99E-01	1.61E-15	9.99E-01	9.08E-01	1.99E-01	9.99E-01
	Mean	1.00E-03	4.87E-03	1.06E + 00	2.33E-01	1.38E + 00	1.26E + 00	3.26E-01	1.36E + 00
	Std.dev	9.98E-03	1.62E-02	2.01E-01	2.15E-01	1.90E-01	1.65E-01	5.83E-02	1.57E-01
	Max	9.98E-02	7.75E-02	1.59E + 00	7.99E-01	1.63E + 00	1.51E + 00	4.99E-01	1.59E + 00
F_10_	Min	3.97E-147	1.41E-37	2.17E + 00	1.14E-32	4.70E-01	2.05E + 01	1.06E-04	2.57E + 01
	Mean	3.54E-100	9.85E-25	3.68E + 00	3.81E-19	9.46E + 00	3.39E + 01	1.07E-02	4.60E + 01
	Std.dev	3.53E-99	4.57E-24	6.04E-01	1.73E-18	1.54E + 01	7.82E + 00	3.81E-02	1.43E + 01
	Max	3.53E-98	2.47E-23	5.07E + 00	9.45E-18	8.53E + 01	4.90E + 01	1.51E-01	7.18E + 01
F_11_	Min	0.00E + 00	0.00E + 00	6.61E-01	0.00E + 00	9.71E-02	7.78E-01	3.70E-01	5.69E-01
	Mean	0.00E + 00	0.00E + 00	7.31E-01	7.38E-10	4.20E-01	8.23E-01	5.61E-01	6.46E-01
	Std.dev	0.00E + 00	0.00E + 00	3.46E-02	4.04E-09	2.00E-01	1.83E-02	9.19E-02	4.26E-02
	Max	0.00E + 00	0.00E + 00	7.93E-01	2.21E-08	7.79E-01	8.53E-01	7.40E-01	7.23E-01
F_12_	Min	8.87E-37	1.11E-11	9.56E + 00	3.56E-18	3.07E-01	1.10E + 04	4.57E-10	8.53E + 03
	Mean	9.85E-27	7.44E-05	2.02E + 07	1.15E + 10	1.14E + 09	7.30E + 07	2.03E-06	3.27E + 08
	Std.dev	5.62E-26	3.65E-04	6.04E + 07	3.65E + 10	5.94E + 09	1.30E + 08	6.04E-06	6.50E + 08
	Max	4.15E-25	2.00E-03	3.04E + 08	1.80E + 11	3.26E + 10	5.02E + 08	2.58E-05	2.36E + 09
F_13_	Min	3.51E-12	3.51E-12	1.73E-09	4.02E-08	6.41E-07	3.96E-06	1.06E-10	6.33E-11
	Mean	3.52E-12	3.51E-12	1.21E-07	4.34E-06	1.27E-05	5.86E-05	1.07E-07	6.12E-09
	Std.dev	2.51E-14	7.40E-15	2.19E-07	5.27E-06	1.54E-05	5.55E-05	3.82E-07	1.19E-08
	Max	3.65E-12	3.54E-12	1.02E-06	1.77E-05	5.98E-05	2.35E-04	2.09E-06	6.64E-08
F_14_	Min	−1.00E + 00	−1.00E + 00	1.60E-13	−1.00E + 00	1.05E-11	3.44E-11	6.27E-13	1.39E-12
	Mean	−9.99E-01	−9.81E-01	4.21E-13	−4.00E-01	3.07E-11	1.36E-10	1.03E-12	3.21E-12
	Std.dev	8.75E-06	3.64E-02	1.92E-13	4.98E-01	1.50E-11	7.33E-11	2.63E-13	1.53E-12
	Max	−9.99E-01	−8.87E-01	9.27E-13	1.10e-09	8.03E-11	3.32E-10	1.87E-12	8.03E-12
F_15_	Min	0.00E + 00	0.00E + 00	5.78E + 00	0.00E + 00	8.82E + 00	9.05E + 00	5.57E + 00	5.15E + 00
	Mean	2.65E-04	2.21E-04	8.13E + 00	4.24E + 00	9.82E + 00	9.99E + 00	8.24E + 00	6.48E + 00
	Std.dev	9.05E-04	5.13E-04	8.57E-01	4.18E + 00	4.41E-01	4.12E-01	8.24E-01	6.42E-01
	Max	7.73E-03	2.33E-03	9.81E + 00	9.51E + 00	1.05E + 01	1.07E + 01	9.84E + 00	7.66E + 00

		AQSCA	AQUILA	MVO	SPOTTED	SINECOS	JAYA	EQUIL	MOTH
F_16_	Min	4.93E-02	2.86E-02	4.48E + 01	3.18E + 01	1.06E + 02	3.51E + 00	1.24E + 01	2.75E + 02
	Mean	4.75E-01	4.43E-01	6.74E + 01	4.97E + 01	1.19E + 04	9.55E + 00	2.79E + 01	1.90E + 03
	Std.dev	4.16E-01	4.43E-01	1.06E + 01	2.03E + 01	1.63E + 04	5.83E + 00	7.16E + 00	1.52E + 03
	Max	1.83E + 00	2.23E + 00	8.67E + 01	1.24E + 02	5.66E + 04	3.40E + 01	4.15E + 01	6.11E + 03
F_17_	Min	2.09E-06	1.19E-05	7.12E-01	6.38E-01	5.33E + 00	3.51E + 00	1.25E-01	2.71E + 00
	Mean	4.38E-03	8.20E-03	1.80E + 01	1.47E + 00	1.68E + 01	9.55E + 00	3.41E-01	1.06E + 01
	Std.dev	4.27E-03	1.19E-02	9.02E + 00	7.43E-01	1.18E + 01	5.83E + 00	1.27E-01	3.91E + 00
	Max	1.86E-02	4.78E-02	3.63E + 01	4.42E + 00	4.85E + 01	3.40E + 01	7.16E-01	1.75E + 01
F_18_	Min	2.67E + 02	1.13E + 03	3.14E + 01	3.28E + 03	2.96E + 03	1.79E + 03	1.73E + 02	4.53E + 02
	Mean	1.34E + 03	1.83E + 03	8.53E + 01	4.73E + 03	4.80E + 03	2.91E + 03	5.70E + 02	8.51E + 02
	Std.dev	4.31E + 02	5.23E + 02	3.99E + 01	9.03E + 02	8.45E + 02	4.91E + 02	2.16E + 02	3.14E + 02
	Max	2.50E + 03	2.92E + 03	1.63E + 02	6.89E + 03	6.56E + 03	3.84E + 03	9.77E + 02	1.77E + 03
F_19_	Min	−8.69E + 02	−8.30E + 02	−8.80E + 02	−6.73E + 02	−3.89E + 02	−8.20E + 02	−8.42E + 02	−7.57E + 02
	Mean	−8.34E + 02	−8.06E + 02	−8.48E + 02	−5.08E + 02	−1.78E + 02	−7.16E + 02	−7.98E + 02	−6.24E + 02
	Std.dev	2.59E + 01	1.59E + 01	2.33E + 01	8.83E + 01	1.24E + 02	4.28E + 01	2.99E + 01	8.22E + 01
	Max	−7.45E + 02	−7.74E + 02	−7.89E + 02	−3.49E + 02	1.22E + 02	−6.29E + 02	−7.26E + 02	−3.39E + 02
F_20_	Min	−4.05E + 01	−3.84E + 01	−4.08E + 01	−2.24E + 01	2.21E + 00	−2.11E + 01	−3.77E + 01	2.83E + 00
	Mean	−3.77E + 01	−3.61E + 01	−3.40E + 01	−1.21E + 01	2.68E + 01	2.90E + 00	−3.29E + 01	3.80E + 01
	Std.dev	1.77E + 00	1.40E + 00	5.04E + 00	4.72E + 00	2.59E + 01	1.54E + 01	3.83E + 00	2.03E + 01
	Max	−0.341E + 01	−3.26E + 01	−2.08E + 01	*4.56E + 00	1.42E + 02	4.82E + 01	−2.02E + 01	8.48E + 01
F_21_	Min	4.94E-138	1.52E-37	6.85E + 01	1.16E-31	1.62E-01	1.00E + 02	1.03E-04	9.42E + 01
	Mean	2.78E-100	4.58E-24	1.26E + 02	7.13E-18	5.56E + 00	1.63E + 02	6.74E-04	1.90E + 02
	Std.dev	1.25E-99	2.50E-23	3.17E + 01	3.87E-17	5.75E + 00	3.27E + 01	6.38E-04	7.25E + 01
	Max	2.02E-98	1.37E-22	2.09E + 02	2.12E-16	1.90E + 01	2.18E + 02	2.39E-03	3.85E + 02
F_22_	Min	4.63E-138	1.47E-36	8.00E-01	5.76E + 00	1.43E + 00	1.27E + 01	3.85E-03	7.96E + 00
	Mean	1.92E-106	8.26E-23	1.89E + 00	1.06E + 01	1.34E + 01	2.86E + 01	2.13E-02	1.66E + 01
	Std.dev	1.92E-105	4.50E-22	9.09E-01	5.50E + 00	8.53E + 00	7.59E + 00	1.37E-02	3.63E + 00
	Max	1.92E-104	2.46E-21	4.38E + 00	3.51E + 01	3.58E + 01	4.27E + 01	5.70E-02	2.51E + 01
F_23_	Min	−2.89E + 01	−2.89E + 01	−2.46E + 01	−1.72E + 01	−1.70E + 01	−1.49E + 01	−2.31E + 01	−2.51E + 01
	Mean	−2.53E + 01	−2.80E + 01	−2.00E + 01	−1.48E + 01	−1.40E + 01	−1.32E + 02	−1.77E + 01	−2.33E + 01
	Std.dev	5.45E + 00	1.17E + 00	2.01E + 00	1.53E + 00	1.30E + 00	9.85E-01	2.58E + 00	1.20E + 00
	Max	−1.17E + 01	−2.42E + 01	−1.51E + 01	−1.22E + 01	−1.22E + 01	−1.11E + 01	−1.36E + 01	−2.04E + 01
F_24_	Min	−6.48E + 02	−6.01E + 02	−6.25E + 02	−5.03E + 02	−3.37E + 02	−7.02E + 01	1.03E-04	−6.58E + 02
	Mean	−4.91E + 02	−4.28E + 02	−3.37E + 02	−3.35E + 02	−1.19E + 02	−6.05E + 02	6.74E-04	−5.06E + 02
	Std.dev	6.52E + 01	1.10E + 02	1.46E + 02	9.07E + 01	1.98E + 02	5.11E + 01	6.38E-04	8.96E + 01
	Max	−3.19E + 02	−2.08E + 02	−1.16E + 01	−1.02E + 02	5.55E + 02	−5.17E + 02	2.39E-03	−2.98E + 02
F_25_	Min	−0.2.90E + 01	−2.90E + 01	−2.45E + 01	−1.87E + 01	−1.89E + 01	−1.47E + 01	−2.17E + 01	−2.61E + 01
	Mean	−2.89E + 01	−2.89E + 01	−2.02E + 01	−1.47E + 01	−1.42E + 01	−1.28E + 01	−1.77E + 01	−2.37E + 01
	Std.dev	3.98E-01	5.24E-02	1.83E + 00	1.67E + 00	1.42E + 00	9.89E-01	1.74E + 00	1.44E + 00
	Max	−2.51E + 01	−2.87E + 01	−1.64E + 01	−1.18E + 01	−1.21E + 01	−1.05E + 01	−1.46E + 01	−2.01E + 01
F_26_	Min	−1.82E + 02	−1.25E + 02	−2.24E + 02	−2.53E-01	−3.17E + 02	−1.08E + 02	−2.05E + 02	−1.12E + 02
	Mean	−1.07E + 02	−7.66E + 01	−1.73E + 02	2.20E + 01	3.56E + 01	−4.05E + 01	−1.53E + 02	−1.87E + 01
	Std.dev	2.59E + 01	2.60E + 01	2.72E + 01	2.26E + 01	1.53E + 02	3.63E + 01	2.65E + 01	5.35E + 01
	Max	−6.24E + 01	2.89E + 01	−1.12E + 02	8.59E + 01	5.92E + 02	3.68E + 01	−8.68E + 01	8.95E + 01
F_27_	Min	9.43E-04	7.87E-04	2.13E + 01	1.63E + 01	5.07E + 01	3.51E + 02	1.29E + 01	5.59E + 02
	Mean	4.97E-01	8.01E-01	3.95E + 01	2.16E + 01	7.45E + 03	1.00E + 03	1.63E + 01	1.76E + 03
	Std.dev	5.20E-01	9.25E-01	1.55E + 01	2.58E + 00	7.15E + 03	5.16E + 02	1.54E + 00	9.25E + 02
	Max	2.17E + 00	3.22E + 00	8.47E + 01	2.62E + 01	3.07E + 04	2.31E + 03	1.87E + 01	5.41E + 03
F_28_	Min	7.07E-12	3.11E-10	2.26E + 00	3.68E-01	1.61E + 01	1.01E + 02	2.84E-02	2.48E + 02
	Mean	9.81E-04	1.66E-04	6.55E + 01	4.76E + 00	2.90E + 03	4.78E + 02	1.17E-01	6.37E + 02
	Std.dev	2.32E-03	3.21E-04	6.37E + 01	1.19E + 01	1.77E + 03	3.36E + 02	5.74E-02	2.95E + 02
	Max	1.34E-02	1.25E-03	3.03E + 02	6.22E + 01	6.99E + 03	1.54E + 03	2.47E-01	1.36E + 03
F_29_	Min	−7.06E-02	−6.15E-02	−6.54E + 02	−6.00E + 02	−4.66E + 02	−4.79E + 02	−5.86E + 02	−5.70E + 02
	Mean	−5.30E-02	−4.85E-02	−5.86E + 02	−4.89E + 02	−3.98E + 02	−4.13E + 02	−5.26E + 02	−5.31E + 02
	Std.dev	4.92E + 01	4.89E + 01	3.92E + 01	3.63E + 01	3.08E + 01	2.02E + 01	3.84E + 01	2.88E + 01
	Max	−4.42E + 02	−4.11E + 02	−5.06E + 02	−4.38E + 02	−3.38E + 02	−3.80E + 02	−4.28E + 02	−4.51E + 02

		AQSCA	AQUILA	MVO	SPOTTED	SINECOS	JAYA	EQUIL	MOTH
F_30_	Min	1.39E-03	1.39E-03	1.43E-03	1.42E-03	1.44E-03	1.45E-03	1.39e-03	1.42E-03
	Mean	1.39E-03	1.39E-03	1.46E-03	1.44E-03	1.46E-03	1.48E-03	1.40E-03	1.44E-03
	Std.dev	2.40E-08	2.92E-09	2.20E-05	1.39E-05	1.34E-05	1.34E-05	1.46E-06	1.21E-05
	Max	1.39E-03	1.39E-03	1.51E-03	1.48E-03	1.50E-03	1.50E-03	1.40E-03	1.48E-03
F_31_	Min	−1.90E + 02	−1.71E + 02	−7.59E + 01	−1.75E + 02	−1.91E + 02	−4.62E + 01	−1.83E + 02	−9.68E + 01
	Mean	−1.75E + 02	−1.71E + 02	−2.99E + 01	−1.72E + 02	−1.81E + 02	−2.99E + 01	−1.76E + 02	−7.88E + 01
	Std.dev	5.31E + 00	2.89E-14	1.65E + 01	1.54E + 00	4.50E + 00	8.46E + 00	4.28E + 00	1.12E + 01
	Max	−1.71E + 02	−1.71E + 02	−5.68E + 00	−1.71E + 02	−1.70E + 02	−9.39E + 00	−1.71E + 02	−4.61E + 01
F_32_	Min	−8.50E + 01	−7.65E + 01	−7.93E + 01	−4.65E + 01	−5.57E + 01	−4.19E + 01	−7.84E + 01	−8.71E + 01
	Mean	−4.70E + 01	−5.67E + 01	−5.83E + 01	−3.87E + 01	−3.88E + 01	−3.33E + 01	−5.65E + 01	−6.68E + 01
	Std.dev	1.02E + 01	1.15E + 01	9.83E + 00	3.99E + 00	4.55E + 00	3.21E + 00	1.09E + 01	9.48E + 00
	Max	−3.30E + 01	2.90E + 01	−3.42E + 01	−2.98E + 01	−3.28E + 01	−2.67E + 01	−3.75E + 01	−4.89E + 01
F_33_	Min	1.52E + 01	1.16E + 01	2.05E + 01	2.28E + 01	2.34E + 01	2.49E + 01	1.96E + 01	2.05E + 01
	Mean	2.23E + 01	2.12E + 01	2.28E + 01	2.43E + 01	2.46E + 01	2.52E + 01	2.16E + 01	2.16E + 01
	Std.dev	2.55E + 00	4.46E + 00	8.81E-01	5.73E-01	4.09E-01	1.70E-01	8.32E-01	6.26E-01
	Max	2.49E + 01	2.57E + 01	2.43E + 01	2.52E + 01	2.54E + 01	2.55E + 01	2.35E + 01	2.28e + 01
F_34_	Min	6.93E + 01	6.93E + 01	2.34E + 03	6.93E + 01	2.51E + 03	2.53E + 03	2.38E + 03	2.19E + 03
	Mean	1.01E + 02	3.98E + 02	2.45E + 02	9.80E + 02	2.56E + 03	2.58E + 03	2.48E + 03	2.32E + 03
	Std.dev	2.01E + 02	5.83E + 02	5.69E + 01	1.06E + 03	2.44E + 01	1.91E + 01	4.60E + 01	6.04E + 01
	Max	1.61E + 03	1.78E + 03	2.56E + 03	2.59E + 03	2.60E + 03	2.61E + 03	2.56E + 03	2.49E + 03
F_35_	Min	7.26E + 02	9.85E + 02	5.06E + 02	9.76E + 02	8.50E + 02	1.11E + 03	7.73E + 02	4.94E + 02
	Mean	9.44E + 02	1.18E + 03	6.42E + 02	1.21E + 03	1.19E + 03	1.29E + 03	9.53E + 02	7.51E + 02
	Std.dev	9.64E + 01	1.10E + 02	7.78E + 01	9.27E + 01	9.28E + 01	6.87E + 01	9.89E + 01	1.16E + 02
	Max	1.16E + 03	1.35E + 03	8.25E + 02	1.33E + 03	1.32E + 03	1.38E + 03	1.15E + 03	9.48E + 02
F_36_	Min	2.48E + 01	2.77E + 01	2.29E + 01	2.80E + 01	3.28E + 01	3.28E + 01	2.30E + 01	2.24E + 01
	Mean	2.81E + 01	3.01E + 01	2.70E + 01	3.25E + 01	4.97E + 01	4.35E + 01	2.48E + 01	2.65e + 01
	Std.dev	1.31E + 00	1.31E + 00	2.54E + 00	2.12E + 00	1.16E + 01	5.16E + 00	1.05E + 00	2.15E + 00
	Max	3.17E + 01	3.31E + 01	3.30E + 01	3.65E + 01	7.29E + 01	5.57E + 03	2.74E + 01	3.20E + 01
F_37_	Min	4.67E-06	4.69E-05	7.60E + 02	1.39E + 01	4.71E + 02	1.45E + 03	2.73E + 02	8.02E + 02
	Mean	1.56E-03	2.29E-03	9.66E + 02	1.39E + 01	1.39E + 03	1.60E + 03	7.86E + 02	1.00E + 03
	Std.dev	1.89E-03	2.90E-03	1.05E + 02	5.11E-11	2.15E + 02	7.33E + 01	2.33E + 02	9.91E + 01
	Max	4.68E-03	1.25E-02	1.17E + 03	1.39E + 01	1.60E + 03	1.73E + 03	1.22E + 03	1.23E + 03
F_38_	Min	−2.49E + 01	−2.63E + 01	−2.81E + 00	−3.81E + 00	−2.31E + 00	−1.78E + 00	−5.08E + 00	−4.64E + 01
	Mean	−1.05E + 01	−1.27E + 01	−1.30E + 00	−2.31E + 00	−1.49E + 00	−1.10E + 00	−2.37E + 00	−3.15E + 00
	Std.dev	4.19E + 00	5.15E + 00	4.72E-01	4.90E-01	3.65E-01	2.47E-01	7.78E-01	7.38E-01
	Max	−6.07E + 01	−7.18E + 00	−8.87E-01	−1.76E + 00	−9.81E-01	−9.09E + 01	−1.43E + 00	−1.87E + 00
F_39_	Min	−9.93E + 01	−8.37E + 01	−7.41E + 01	−6.15E + 01	−6.45E + 01	−5.32E-01	−8.26E + 01	−7.91E + 01
	Mean	−6.40E + 01	−6.22E + 01	−6.58E + 01	−5.52E + 01	−5.55E + 01	−4.95E + 01	−7.28E + 01	−7.12E + 01
	Std.dev	1.03E + 01	1.06E + 00	4.88E + 00	3.48E + 00	3.07E + 00	1.62E + 00	6.23E + 00	4.13E + 00
	Max	−5.12E + 01	−5.05E + 01	−5.73E + 01	−4.85E + 01	−5.02E + 01	−4.55E + 01	−5.69E + 01	−6.38E + 01
F_40_	Min	2.90E + 01	2.90E + 01	2.35E + 03	2.90E + 01	1.33E + 03	2.53E + 03	1.37E + 03	2.19E + 03
	Mean	2.90E + 01	7.59E + 01	2.47E + 03	3.00E + 02	2.11E + 03	2.61E + 03	1.56E + 03	2.36E + 03
	Std.dev	0.00E + 00	6.84E + 01	6.11e + 01	4.39E + 02	3.84E + 02	2.67E + 01	1.13E + 02	6.42E + 01
	Max	2.90E + 01	2.62e + 02	2.60E + 03	1.94E + 03	2.54E + 03	2.64E + 03	1.86E + 03	2.48E + 03
F_41_	Min	9.32E + 02	8.58E + 04	8.99E + 01	7.58E + 02	0.00E + 00	5.81E + 02	5.64E + 02	1.76E + 03
	Mean	8.54E + 07	2.85E + 09	1.33E + 05	1.16E + 08	0.00E + 00	9.31E + 03	4.30E + 03	5.36E + 04
	Std.dev	3.63E + 08	7.94E + 09	4.67E + 05	4.44E + 08	0.00E + 00	1.03E + 04	4.46E + 03	5.80E + 04
	Max	3.16E + 09	3.52E + 10	2.24E + 06	2.28E + 09	0.00E + 00	4.52E + 04	2.17E + 04	2.28E + 05
F_42_	Min	5.01E + 02	2.76E + 05	2.89E + 02	5.04E + 02	0.00E + 00	1.07E + 03	1.07E + 03	4.54E + 03
	Mean	2.34E + 08	1.45E + 09	3.55E + 04	4.74E + 06	0.00E + 00	1.10E + 04	9.07E + 03	5.38E + 04
	Std.dev	1.08E + 09	3.50E + 09	9.53E + 04	8.89E + 06	0.00E + 00	1.42E + 04	1.52E + 04	7.12E + 04
	Max	9.57E + 09	1.78E + 10	4.91E + 05	3.75E + 07	0.00E + 00	6.82E + 04	7.71E + 04	3.71E + 05
F_43_	Min	7.03E + 64	7.03E + 64	7.02E + 64	7.03E + 64	5.81E + 64	7.03E + 64	7.03E + 64	7.03E + 64
	Mean	7.03E + 64	7.03E + 64	7.03E + 64	7.03E + 64	6.96E + 64	7.03E + 64	7.03E + 64	7.03E + 64
	Std.dev	1.57E + 59	8.84E + 58	1.31E + 61	1.26E + 58	2.30e + 63	1.44E + 56	1.40E + 58	1.53E + 58
	Max	7.03E + 64	7.03E + 64	7.03E + 64	7.03E + 64	7.03E + 64	7.03E + 64	7.03E + 64	7.03E + 64
F_44_	Min	0.00E + 00	0.00E + 00	2.05E-06	1.06E-22	2.36E-02	5.56E-02	4.33E-07	9.53E-04
	Mean	0.00E + 00	0.00E + 00	3.05E-06	4.82E-04	7.98E-02	1.42E-01	4.99E-05	4.75E-03
	Std.dev	0.00E + 00	0.00E + 00	1.99E-06	1.63E-03	3.24E-02	4.46E-02	5.05E-05	4.14E-03
	Max	0.00E + 00	0.00E + 00	7.45E-06	8.20E-03	1.77E-01	2.27E-01	1.83E-04	2.11E-02

		AQSCA	AQUILA	MVO	SPOTTED	SINECOS	JAYA	EQUIL	MOTH
F_45_	Min	−9.99E-01	−9.99E-01	−9.93E-01	−9.02E-01	−6.89E-01	−9.53E-01	−9.85E-01	−9.64E-01
	Mean	−9.79E-01	−9.86E-01	−9.48E-01	−8.41E-01	−2.25E-01	−8.51E-01	−9.79E-01	−9.33E-01
	Std.dev	2.15E-02	2.06E-02	3.60E-02	5.38E-02	2.26E-01	5.38E-02	9.05E-03	2.25E-02
	Max	−8.99E-01	−9.17E-01	−8.67E-01	−6.59E-01	5.77E-04	−7.33E-01	−9.44E-01	−8.77E-01
F_46_	Min	−9.99E-01	−9.99E-01	−2.32E-12	−1.62E-01	−3.59E-18	−2.37E-29	−3.37E-07	−2.36E-15
	Mean	−9.20E-01	−8.78E-01	−7.85E-14	−2.83E-02	−1.19E-19	−1.48E-30	−1.18E-08	−1.40E-16
	Std.dev	1.20E-01	2.17E-01	4.20E-13	4.28E-02	6.56E-19	5.18E-30	6.16E-08	4.69E-16
	Max	−3.68E-01	−5.08E-02	−3.67E-34	−6.50E-14	−3.42E-37	−1.43E-39	−8.73E-25	−1.61E-25
F_47_	Min	2.78E + 01	4.56E + 01	1.66E + 02	2.11E + 02	3.09E + 02	2.95E + 02	1.63E + 02	1.40E + 02
	Mean	1.10E + 02	9.75E + 01	1.98E + 02	2.46E + 02	3.30E + 02	3.47E + 02	2.11E + 02	2.03E + 02
	Std.dev	2.71E + 01	2.43E + 01	2.35E + 01	1.82E + 01	1.32E + 01	2.04E + 01	2.32E + 01	2.63E + 01
	Max	1.61E + 02	1.23E + 02	2.51E + 02	2.79E + 02	3.53E + 02	3.77E + 02	2.53E + 02	2.61E + 02
F_48_	Min	1.73E-02	8.39E-02	3.01E + 01	1.99E + 01	7.33E + 01	7.63E + 01	3.06E + 01	3.46E + 01
	Mean	1.81E + 01	2.51E + 01	3.08E + 01	3.34E + 01	9.76E + 01	1.00E + 02	3.64E + 01	4.01E + 01
	Std.dev	1.66E + 01	1.37E + 01	3.31E-02	8.77E + 00	9.51E + 00	1.02E + 01	3.62E + 00	2.76E + 00
	Max	4.25E + 01	4.01E + 01	3.01E + 01	5.18E + 01	1.13E + 02	1.18E + 02	4.63E + 01	4.80E + 01
F_49_	Min	−1.32E + 01	−1.09E + 01	−1.38E + 01	−1.33E + 01	−1.14E + 02	−1.20E + 01	−1.60E + 01	−2.27E + 01
	Mean	−1.12E + 01	−9.63E + 00	−1.15E + 01	−1.07E + 01	−9.65E + 00	−9.92E + 00	−1.29E + 01	−1.80E + 01
	Std.dev	8.37E-01	5.78E-01	1.14E + 00	9.57E-01	8.05E-01	7.30E-01	1.52E + 00	1.57E + 00
	Max	−8.98E + 00	−8.62E + 00	−9.25E + 00	−9.58E + 00	−8.43E + 00	−8.79E + 00	−1.01E + 01	−1.48E + 01
F_50_	Min	8.17E-146	1.18E-37	2.95E + 01	1.09E-25	8.20E + 00	8.35E + 02	9.26E-04	8.59E + 02
	Mean	4.66E-101	4.07E-21	1.08E + 02	9.74E-14	1.30E + 03	1.37E + 03	5.44E-03	1.54E + 03
	Std.dev	4.66E-100	1.81E-20	5.73E + 01	5.29E-13	1.50E + 03	3.98E + 02	5.74E-03	4.76E + 02
	Max	4.66E-99	9.89E-20	2.69E + 02	2.90E-12	6.39E + 03	2.09E + 03	2.65E-02	2.79E + 03
F_51_	Min	1.47E-120	1.69E-37	6.14E + 02	2.52E-35	3.19E + 01	1.40E + 03	4.10E-02	1.39E + 03
	Mean	1.41E-45	3.84E-22	1.26E + 03	7.76E + 01	5.01E + 02	2.12E + 03	2.83E-01	2.32E + 03
	Std.dev	1.41E-44	1.20E-21	2.85E + 02	4.25E + 02	5.91E + 02	3.45E + 02	4.29E-01	3.24E + 02
	Max	1.41E-43	4.97E-21	1.65E + 03	2.33E + 03	2.26E + 03	2.91E + 03	2.36E + 00	3.11E + 03
F_52_	Min	−9.98E-01	−9.98E-01	1.50E + 00	−3.73E-01	4.64E + 00	4.10E-01	4.09E-01	1.50E + 00
	Mean	−7.76E-01	−7.94E-01	2.46E + 00	6.00E-01	7.94E + 00	1.66E + 00	1.27E + 00	2.97E + 00
	Std.dev	2.11E-01	2.49E-01	9.04E-01	5.01E-01	1.39E + 00	8.77E-01	6.73E-01	1.01E + 00
	Max	4.09E-01	4.10E-01	4.63E + 00	1.50E + 00	1.02E + 01	2.91E + 00	2.91E + 00	4.63E + 00
F_53_	Min	−9.99E-01	−9.99E-01	−9.00E-01	−8.79E-01	−3.00E-02	−6.20E-03	−4.32E-01	−4.11E-01
	Mean	−9.88E-01	−9.77E-01	−8.31E-01	−6.26E-01	−4.65E-03	−2.11E-03	−1.49E-01	−2.04E-01
	Std.dev	2.25E-02	4.48E-02	4.10E-02	1.90E-01	5.24E-03	1.32E-03	1.02E-01	1.04E-01
	Max	−8.73E-01	−8.01E-01	−7.45E-01	−3.08E-03	−4.77E-04	−4.20E-04	−2.64E-01	−3.79E-02
F_54_	Min	0.00E + 00	0.00E + 00	2.50E + 01	0.00E + 00	0.00E + 00	1.28E + 02	0.00E + 00	1.27E + 02
	Mean	0.00E + 00	0.00E + 00	5.55E + 01	2.00E-01	2.53E + 00	1.80E + 02	0.00E + 00	1.83E + 02
	Std.dev	0.00E + 00	0.00E + 00	2.04E + 01	4.84E-01	5.23E + 00	2.93E + 01	0.00E + 00	3.97E + 01
	Max	0.00E + 00	0.00E + 00	9.81E + 01	2.00E + 00	2.40E + 01	2.50E + 02	0.00E + 00	2.70E + 02
F_55_	Min	0.00E + 00	0.00E + 00	2.30E + 01	0.00E + 00	1.10E + 01	1.64E + 03	0.00E + 00	1.50E + 03
	Mean	0.00E + 00	0.00E + 00	7.90E + 01	4.00E-01	6.76E + 02	3.04E + 03	2.66E-01	3.51E + 03
	Std.dev	0.00E + 00	0.00E + 00	2.67E + 01	1.61E + 00	1.27E + 03	7.86E + 02	5.20E-01	1.31E + 03
	Max	0.00E + 00	0.00E + 00	1.46E + 02	8.00E + 00	6.47E + 03	5.33E + 03	2.00E + 00	7.47E + 03

		AQSCA	AQUILA	MVO	SPOTTED	SINECOS	JAYA	EQUIL	MOTH
F_56_	Min	6.66E-02	1.38E-01	4.59E-02	9.73E-02	0.00E + 00	5.88E-02	5.11E-02	3.94E-02
	Mean	1.32E-01	1.88E-01	7.62E-02	1.33E-01	0.00E + 00	7.23E-02	7.02E-02	7.28E-02
	Std.dev	2.18E-02	2.82E-01	1.76E-02	1.82E-02	0.00E + 00	8.26E-03	1.18E-02	1.46E-02
	Max	1.78E-01	2.58E-01	1.29E-01	1.75E-01	0.00E + 00	9.43E-02	9.98E-02	1.01E-01
F_57_	Min	1.26E-01	1.24E-01	1.89E-01	2.38E-01	−8.41E + 16	2.32E-01	1.69E-01	1.80E-01
	Mean	2.19E-01	2.04E-01	2.16E-01	2.98E-01	−7.52E + 15	3.30E-01	2.09E-01	2.10E-01
	Std.dev	5.94E-02	6.04E-02	1.47E-02	2.48E-02	1.72E + 16	3.34E-02	1.25E-02	1.33E-02
	Max	3.62E-01	3.45E-01	2.43E-01	3.34E-01	−3.64E + 13	3.84E-01	2.29E-01	2.34E-01
F_58_	Min	2.16E-02	4.41E-02	5.57E-02	9.81E-02	−2.19E + 19	9.21E-02	6.16E-02	5.58E-02
	Mean	7.12E-02	8.57E-02	9.71E-02	1.31E-01	−3.44E + 18	1.26E-01	8.14E-02	8.07E-02
	Std.dev	1.78E-02	2.93E-02	2.22E-02	1.78E-02	4.43E + 18	2.13E-02	1.32E-02	1.17E-02
	Max	1.12E-01	1.43E-01	1.49E-01	1.63E-01	−1.48E + 16	1.68E-01	1.07E-01	9.81E-02
F_59_	Min	0.00E + 00	0.00E + 00	2.04E + 01	0.00E + 00	2.01E + 00	1.48E + 02	3.93E-04	1.26E + 02
	Mean	0.00E + 00	1.14E-05	2.59E + 01	1.48E-17	5.94E + 01	2.23E + 02	1.16E-01	2.44E + 02
	Std.dev	0.00E + 00	5.97E-05	4.84E + 00	8.10E-17	4.74E + 01	5.26E + 01	4.38E-01	5.02E + 01
	Max	0.00E + 00	3.27E-04	3.69E + 01	4.44E-16	1.61E + 02	3.96E + 02	2.19E + 00	3.32E + 02
F_60_	Min	−1.00E + 00	−9.99E-01	−9.04E-01	−6.63E-01	−6.53E-01	−5.90E-01	−8.17E-01	−9.18E-01
	Mean	−9.99E-01	−9.99E-01	−8.22E-01	−5.89E-01	−5.89E-01	−5.50E-01	−7.24E-01	−8.35E-01
	Std.dev	4.06E-04	3.73E-04	4.09E-02	3.20E-02	3.04E-02	1.85E-02	4.71E-02	4.49E-02
	Max	−9.96E-01	−9.99E-01	−6.94E-01	−5.21E-02	−5.36E-01	−5.13E-01	−6.35E-01	−7.43E-02
F_61_	Min	4.67E-05	9.87E-05	1.53E + 00	2.50E + 00	3.25E + 00	2.34E + 00	2.53E + 00	2.58E-01
	Mean	6.94E-01	3.24E-01	2.70E + 00	3.76E + 00	4.25E + 00	4.02E + 00	4.04E + 00	6.90E-01
	Std.dev	1.53E + 00	1.11E + 00	5.05E-01	6.42E-01	5.09E-01	5.88E-01	6.42E-01	2.39E-01
	Max	4.91E + 00	4.54E + 00	3.78E + 00	5.04E + 00	5.23E + 00	5.02E + 00	5.07E + 00	1.21E + 00
F_62_	Min	1.54E-23	7.88E-15	2.58E + 01	7.80E-34	2.61E + 00	1.11E + 02	1.60E + 00	2.12E + 02
	Mean	8.23E-01	6.42E-11	6.09E + 01	2.61E-15	1.60E + 02	1.76E + 02	1.02E + 01	3.12E + 02
	Std.dev	1.86E + 00	2.33E-10	2.14E + 01	1.42E-14	9.76E + 01	3.11E + 01	9.66E + 00	6.60E + 01
	Max	8.89E + 00	1.28E-09	1.09E + 02	7.79E-14	4.74E + 02	2.54E + 02	5.40E + 01	4.52E + 02
F_63_	Min-	−6.38E + 00	−6.38E + 00	8.48E + 02	4.94E + 01	2.69E + 02	3.46E + 04	2.07E + 01	3.99E + 04
	Mean	−3.83E + 00	−6.12E + 00	1.66E + 03	8.07E + 01	1.50E + 04	7.53E + 04	4.20E + 01	9.40E + 04
	Std.dev	3.03E + 00	6.32E-01	4.73E + 02	2.46E + 01	1.87E + 04	2.30E + 04	1.57E + 01	2.87E + 04
	Max	9.98E + 00	−3.40E + 00	2.71E + 03	1.57E + 02	6.53E + 04	1.41E + 05	9.01E + 01	1.62E + 05
F_64_	Min	1.74E + 03	1.74E + 03	1.75E + 03	1.74E + 03	1.74E + 03	1.75E + 03	1.74E + 03	1.75E + 03
	Mean	1.74E + 03	1.74E + 03	1.76E + 03	1.74E + 03	1.74E + 03	1.76E + 03	1.74E + 03	1.75E + 03
	Std.dev	0.00E + 00	0.00E + 00	3.25E + 00	2.38E-11	6.86E-01	1.91E + 00	6.70E-02	1.86E + 00
	Max	1.74E + 03	1.74E + 03	1.76E + 03	1.73E + 03	1.74E + 03	1.76E + 03	1.74E + 03	1.76E + 03
F_65_	Min	3.99E-07	2.98E-06	8.00E + 02	4.08E + 02	2.13E + 04	8.64E + 04	4.10E + 02	1.03E + 06
	Mean	5.20E + 00	6.44E + 00	9.57E + 02	4.25E + 02	4.95E + 08	4.48E + 06	4.38E + 02	1.70E + 07
	Std.dev	4.27E + 00	4.00E + 00	2.83E + 02	9.12E + 01	6.09E + 08	1.46E + 07	2.80E + 01	2.58E + 07
	Max	9.81E + 00	9.59E + 00	2.42E + 03	9.09E + 02	2.37E + 09	8.15E + 07	5.11E + 02	1.40E + 08
F_66_	Min	6.31E-05	1.10E-05	5.49E + 02	5.08E-01	1.91E + 04	2.43E + 06	2.55E-01	4.63E + 06
	Mean	9.39E-02	9.67E-02	7.69E + 03	9.26E-01	3.38E + 07	8.09E + 06	1.02E + 00	1.41E + 07
	Std.dev	1.20E-01	1.66E-01	6.39E + 03	1.25E-01	5.58E + 07	4.45E + 06	8.90E-01	9.26E + 06
	Max	4.61E-01	6.86E-01	2.37E + 04	9.97E-01	2.36E + 08	1.83E + 07	4.65E + 00	4.80E + 07
F_67_	Min	−2.19E + 02	−1.98E + 02	−2.42E + 02	−2.30E + 02	−1.06E + 21	−1.50E + 02	−2.52E + 02	−2.19E + 02
	Mean	−1.72E + 02	−1.53E + 02	−2.12E + 02	−1.86E + 02	−2.41E + 20	−1.18E + 02	−2.21E + 02	−1.98E + 02
	Std.dev	2.02E + 01	2.33E + 01	1.82E + 01	2.07E + 01	3.26E + 20	1.23E + 01	1.35E + 01	1.18E + 01
	Max	−1.16E + 02	−1.15E + 02	−1.57E + 02	−1.44E + 02	−2.58E + 18	−9.31E + 01	−1.99E + 02	−1.71E + 02
F_68_	Min	3.17E + 01	3.21E + 01	5.38E + 02	4.49E + 01	1.46E + 04	1.30E + 06	4.21E + 01	8.65E + 05
	Mean	3.21E + 01	3.22E + 01	1.75E + 03	5.34E + 01	2.29E + 07	5.26E + 06	5.11E + 01	7.89E + 06
	Std.dev	2.56E-01	3.40E-01	1.44E + 03	5.99E + 00	4.56E + 07	2.24E + 06	4.84E + 00	4.39E + 06
	Max	3.30E + 01	3.40E + 01	5.90E + 03	7.00E + 01	2.14E + 08	1.14E + 07	6.08E + 01	1.83E + 07
F_69_	Min	1.27E + 01	1.33E + 01	5.54E + 02	1.44E + 01	4.79E + 03	1.46E + 06	1.25E + 01	1.39E + 06
	Mean	1.34E + 01	1.43E + 01	2.06E + 03	1.48E + 01	1.03E + 07	4.72E + 06	1.29E + 01	4.89E + 06
	Std.dev	2.32E-01	3.43E-01	1.48E + 03	1.77E-01	1.82E + 07	2.84E + 06	2.78E-01	2.47E + 06
	Max	1.40E + 01	1.48E + 01	7.93E + 03	1.50E + 01	8.34E + 07	1.05E + 07	1.36E + 01	1.25E + 07

**Table 9:** Wilcoxon signed rank test results for 30D multi modal problems.

	AQSCA	AQUILA	MVO	SPOTTED	SINECOS	JAYA	EQUIL	MOTH
F_1_	N/A	0.323	< 0.05	< 0.05	< 0.05	< 0.05	< 0.05	< 0.05
	1	2( +)	6( +)	3( +)	5( +)	7( +)	4( +)	8( +)
F_2_	N/A	1.00	< 0.05	1.00	< 0.05	< 0.05	< 0.05	< 0.05
	1	1( =)	5( +)	1( +)	6( +)	8( +)	4( +)	7( +)
F_3_	N/A	0.500	< 0.05	1.00	< 0.05	< 0.05	< 0.05	< 0.05
	2	3( +)	6( +)	1(−)	5( +)	8( +)	4( +)	7( +)
F_4_	N/A	< 0.05	< 0.05	< 0.05	< 0.05	< 0.05	< 0.05	< 0.05
	1	2( +)	5( +)	4( +)	6( +)	7( +)	3( +)	8( +)
F_5_	N/A	< 0.05	< 0.05	< 0.05	< 0.05	< 0.05	< 0.05	< 0.05
	2	3( +)	7( +)	1(−)	5( +)	8( +)	4( +)	6( +)
F_6_	N/A	< 0.05	< 0.05	< 0.05	< 0.05	< 0.05	< 0.05	< 0.05
	2	1(−)	6( +)	4( +)	7( +)	8( +)	3( +)	5( +)
F_7_	N/A	< 0.05	< 0.05	< 0.05	< 0.05	< 0.05	< 0.05	< 0.05
	1	2( +)	4( +)	5( +)	8( +)	6( +)	3( +)	7( +)
F_8_	N/A	1.00	< 0.05	< 0.05	< 0.05	< 0.05	< 0.05	< 0.05
	1	1( =)	3( +)	6( +)	7( +)	8( +)	5( +)	4( +)
F_9_	N/A	< 0.05	< 0.05	< 0.05	< 0.05	< 0.05	< 0.05	< 0.05
	1	2( +)	5( +)	6( +)	4( +)	7( +)	3( +)	8( +)
F_10_	N/A	< 0.05	< 0.05	< 0.05	< 0.05	< 0.05	< 0.05	< 0.05
	1	2( +)	5( +)	6( +)	4( +)	7( +)	3( +)	8( +)
F_11_	N/A	1.00	< 0.05	< 0.05	< 0.05	< 0.05	< 0.05	< 0.05
	1	1( =)	7( +)	6( +)	4( +)	8( +)	3( +)	5( +)
F_12_	N/A	< 0.05	< 0.05	< 0.05	< 0.05	< 0.05	< 0.05	< 0.05
	1	2( +)	6( +)	5( +)	4( +)	7( +)	3( +)	8( +)
F_13_	N/A	< 0.05	0.321	< 0.05	< 0.05	< 0.05	< 0.05	< 0.05
	5	2(−)	4(−)	6( +)	7( +)	8( +)	3(−)	1(−)
F_14_	N/A	1.00	< 0.05	< 0.05	< 0.05	< 0.05	< 0.05	< 0.05
	1	1( =)	3( +)	4( +)	8( +)	7( +)	4( +)	6( +)
F_15_	N/A	< 0.05	< 0.05	< 0.05	< 0.05	< 0.05	< 0.05	< 0.05
	1	2( +)	4( +)	6( +)	7( +)	8( +)	5( +)	3( +)
F_16_	N/A	< 0.05	< 0.05	< 0.05	< 0.05	< 0.05	< 0.05	< 0.05
	1	2( +)	4( +)	5( +)	8( +)	6( +)	3( +)	7( +)
F_17_	N/A	< 0.05	< 0.05	< 0.05	< 0.05	< 0.05	< 0.05	< 0.05
	1	2( +)	8( +)	4( +)	5( +)	6( +)	3( +)	7( +)
F_18_	N/A	< 0.05	< 0.05	< 0.05	< 0.05	< 0.05	< 0.05	< 0.05
	6	2(−)	1(−)	3(−)	8( +)	7( +)	4(−)	5(−)
F_19_	N/A	< 0.05	< 0.05	< 0.05	< 0.05	< 0.05	< 0.05	< 0.05
	1	2( +)	3( +)	5( +)	8( +)	6( +)	4( +)	7( +)
F_20_	N/A	< 0.05	< 0.05	< 0.05	< 0.05	< 0.05	< 0.05	< 0.05
	1	2( +)	3( +)	5( +)	7( +)	6( +)	4( +)	8( +)
F_21_	N/A	< 0.05	< 0.05	< 0.05	< 0.05	< 0.05	< 0.05	< 0.05
	1	2( +)	6( +)	5( +)	4( +)	7( +)	3( +)	8( +)
F_22_	N/A	< 0.05	< 0.05	< 0.05	< 0.05	< 0.05	< 0.05	< 0.05
	1	2( +)	4( +)	6( +)	5( +)	8( +)	3( +)	7( +)
F_23_	N/A	0.775	< 0.05	< 0.05	< 0.05	< 0.05	< 0.05	< 0.05
	5	6( +)	2(−)	4(−)	8( +)	7( +)	3(−)	1(−)
F_24_	N/A	< 0.05	< 0.05	< 0.05	< 0.05	< 0.05	< 0.05	< 0.05
	1	2( +)	7( +)	4( +)	8( +)	5( +)	3( +)	6( +)
F_25_	N/A	0.754	< 0.05	< 0.05	< 0.05	< 0.05	< 0.05	< 0.05
	5	6( +)	2(−)	4(−)	8( +)	7( +)	3(−)	1(−)
F_26_	N/A	< 0.05	< 0.05	< 0.05	< 0.05	< 0.05	< 0.05	< 0.05
	1	3( +)	2( +)	6( +)	5( +)	7( +)	4( +)	8( +)
F_27_	N/A	< 0.05	< 0.05	< 0.05	< 0.05	< 0.05	< 0.05	< 0.05
	1	2( +)	4( +)	5( +)	8( +)	6( +)	3( +)	7( +)
F_28_	N/A	< 0.05	< 0.05	< 0.05	< 0.05	< 0.05	< 0.05	< 0.05
	2	1(−)	4( +)	5( +)	8( +)	6( +)	3( +)	7( +)
F_29_	N/A	0.06	< 0.05	< 0.05	< 0.05	< 0.05	0.236	0.891
	2	5( +)	1(−)	6( +)	8( +)	7( +)	4( +)	3( +)
F_30_	N/A	< 0.05	< 0.05	< 0.05	< 0.05	< 0.05	< 0.05	< 0.05
	3	1(−)	7( +)	4( +)	6( +)	8( +)	2(−)	5( +)

	AQSCA	AQUILA	MVO	SPOTTED	SINECOS	JAYA	EQUIL	MOTH
F_31_	N/A	< 0.05	< 0.05	< 0.05	< 0.05	< 0.05	0.672	< 0.05
	3	4( +)	7( +)	5( +)	1(−)	8( +)	2(−)	6( +)
F_32_	N/A	< 0.05	< 0.05	< 0.05	< 0.05	< 0.05	< 0.05	< 0.05
	6	4(−)	2(−)	5(−)	7( +)	8( +)	3(−)	1(−)
F_33_	N/A	< 0.365	< 0.05	< 0.901	< 0.05	< 0.05	< 0.05	< 0.05
	4	3(−)	6( +)	5( +)	7( +)	8( +)	1(−)	2(−)
F_34_	N/A	1.00	< 0.05	< 0.05	< 0.05	< 0.05	< 0.05	< 0.05
	1	1( =)	4( +)	6( +)	8( +)	7( +)	5( +)	3( +)
F_35_	N/A	< 0.05	< 0.05	0.762	< 0.05	< 0.05	< 0.05	< 0.05
	6	3(−)	1(−)	5(−)	8( +)	7( +)	4(−)	2(−)
F_36_	N/A	< 0.05	0.185	< 0.05	< 0.05	< 0.05	< 0.05	0.183
	6	3(−)	5(−)	2(−)	8( +)	7( +)	1(−)	4(−)
F_37_	N/A	1.00	< 0.05	< 0.05	< 0.05	< 0.05	< 0.05	< 0.05
	1	2( +)	4( +)	6( +)	7( +)	8( +)	3( +)	5( +)
F_38_	N/A	0.679	< 0.05	< 0.05	< 0.05	< 0.05	< 0.05	< 0.06
	2	3( +)	7( +)	5( +)	6( +)	8( +)	4( +)	1(−)
F_39_	N/A	< 0.294	< 0.05	< 0.05	< 0.05	< 0.05	< 0.05	< 0.05
	3	4( +)	5( +)	6( +)	7( +)	8( +)	1(−)	2(−)
F_40_	N/A	1.00	< 0.05	< 0.05	< 0.05	< 0.05	< 0.05	< 0.05
	1	1( =)	7( +)	6( +)	4( +)	8( +)	3( +)	5( +)
F_41_	N/A	< 0.05	< 0.05	< 0.05	< 0.05	< 0.05	< 0.05	< 0.05
	1	3( +)	5( +)	8( +)	2( +)	5( +)	4( +)	7( +)
F_42_	N/A	< 0.05	< 0.05	< 0.05	0.483	< 0.05	< 0.05	< 0.05
	2	3( +)	7( +)	8( +)	1(−)	5( +)	4( +)	6( +)
F_43_	N/A	< 0.05	< 0.05	< 0.05	< 0.05	< 0.05	< 0.05	< 0.05
	1	4( +)	3( +)	5( +)	2( +)	6( +)	7( +)	8( +)
F_44_	N/A	< 0.05	< 0.05	< 0.05	< 0.05	< 0.05	< 0.05	< 0.05
	1	2( +)	3( +)	6( +)	8( +)	7( +)	4( +)	5( +)
F_45_	N/A	< 0.05	< 0.05	< 0.05	< 0.05	< 0.05	< 0.05	< 0.05
	1	2( +)	5( +)	3( +)	8( +)	7( +)	4( +)	6( +)
F_46_	N/A	0.167	< 0.05	< 0.05	< 0.05	< 0.05	< 0.05	< 0.05
	1	2( +)	4( +)	6( +)	7( +)	8( +)	3( +)	5( +)
F_47_	N/A	< 0.05	< 0.05	< 0.05	< 0.05	< 0.05	< 0.05	< 0.05
	1	3( +)	2( +)	6( +)	8( +)	7( +)	5( +)	4( +)
F_48_	N/A	< 0.05	< 0.05	< 0.05	< 0.05	< 0.05	< 0.05	< 0.05
	1	2( +)	3( +)	4( +)	8( +)	7( +)	5( +)	6( +)
F_49_	N/A	< 0.198	< 0.05	< 0.05	< 0.05	< 0.05	< 0.05	< 0.05
	5	4( +)	6( +)	2( +)	8( +)	7( +)	3( +)	1(−)
F_50_	N/A	< 0.05	< 0.05	< 0.05	< 0.05	< 0.05	< 0.05	< 0.05
	1	2( +)	4( +)	6( +)	5( +)	8( +)	3( +)	7( +)
F_51_	N/A	< 0.05	< 0.05	< 0.05	< 0.05	< 0.05	< 0.05	< 0.05
	1	2( +)	6( +)	5( +)	4( +)	7( +)	3( +)	8( +)
F_52_	N/A	< 0.05	< 0.05	< 0.05	< 0.05	< 0.05	< 0.05	< 0.05
	1	2( +)	6( +)	4( +)	8( +)	5( +)	3( +)	7( +)
F_53_	N/A	< 0.05	< 0.05	< 0.05	< 0.05	< 0.05	< 0.05	< 0.05
	1	2( +)	3( +)	6( +)	8( +)	7( +)	4( +)	5( +)
F_54_	N/A	1.00	< 0.05	< 0.05	< 0.05	< 0.05	1.00	< 0.05
	1	1( =)	5( +)	6( +)	4( +)	7( +)	1( =)	8( +)
F_55_	N/A	1.00	< 0.05	< 0.05	< 0.05	< 0.05	< 0.05	< 0.05
	1	1( =)	5( +)	6( +)	4( +)	7( +)	3( +)	8( +)
F_56_	N/A	< 0.05	< 0.05	0.362	< 0.05	< 0.05	< 0.05	< 0.05
	4	2(−)	6(−)	3(−)	1(−)	5( +)	8( +)	7( +)
F_57_	N/A	< 0.05	< 0.05	< 0.05	< 0.05	< 0.05	< 0.05	< 0.05
	2	4( +)	6( +)	7( +)	1(−)	8( +)	3( +)	5( +)
F_58_	N/A	< 0.05	< 0.05	< 0.05	< 0.05	< 0.05	< 0.05	< 0.05
	2	3( +)	7( +)	4( +)	1(−)	8( +)	5( +)	6( +)
F_59_	N/A	1.00	< 0.05	< 0.05	< 0.05	< 0.05	< 0.05	< 0.05
	1	1( =)	5( +)	6( +)	4( +)	7( +)	3( +)	8( +)
F_60_	N/A	< 0.05	< 0.05	< 0.05	< 0.05	< 0.05	< 0.05	< 0.05
	6	5(−)	2(−)	4(−)	7( +)	8( +)	3(−)	1(−)
F_61_	N/A	< 0.05	< 0.05	< 0.05	< 0.05	< 0.05	< 0.05	< 0.05
	4	5( +)	2(−)	3(−)	8( +)	6( +)	7( +)	1(−)
F_62_	N/A	0.165	< 0.05	< 0.05	< 0.05	< 0.05	< 0.05	< 0.05
	3	2(−)	5( +)	1(−)	6( +)	7( +)	4( +)	8( +)
F_63_	N/A	0.896	< 0.05	< 0.05	< 0.05	< 0.05	< 0.05	< 0.05
	2	1(−)	5( +)	4( +)	6( +)	7( +)	3( +)	8( +)
F_64_	N/A	< 0.05	0.093	< 0.05	0.093	0.093	0.093	0.093
	1	2( +)	7( +)	3( +)	5( +)	8( +)	4( +)	6( +)
F_65_	N/A	0.207	< 0.05	< 0.05	< 0.05	< 0.05	< 0.05	< 0.05
	1	2( +)	5( +)	3( +)	8( +)	6( +)	4( +)	7( +)
F_66_	N/A	< 0.05	< 0.05	< 0.05	< 0.05	< 0.05	< 0.05	< 0.05
	1	2( +)	5( +)	3( +)	8( +)	6( +)	4( +)	7( +)
F_67_	N/A	< 0.05	0.08	< 0.05	< 0.05	< 0.05	0.09	< 0.05
	6	7( +)	3(−)	5(−)	1(−)	8( +)	2(−)	4(−)
F_68_	N/A	< 0.05	< 0.05	0.201	< 0.05	< 0.05	0.09	< 0.05
	1	2( +)	5( +)	4( +)	8( +)	6( +)	3( +)	7( +)
F_69_	N/A	0.09	< 0.05	0.09	< 0.05	< 0.05	0.09	< 0.05
	2	3( +)	5( +)	4( +)	8( +)	6( +)	1(−)	7( +)
Aver. rank	2.07	2.47	4.59	4.65	5.95	7.01	3.47	5.53
Ranking	1	2	4	5	7	8	3	6
** + / = /-**		48/9/12	57/0/12	57/0/12	63/0/6	69/0/0	54/1/14	56/0/13

**Table 10:** Comparative analysis of the prediction results for 30D unimodal test problems.

		AQSCA		AQUILA	MVO	SPOTTED	SINECOS	JAYA		EQUIL	MOTH
F_70_	Min	1.25E-143		2.68E-36	2.32E-01	7.17E-37	7.65E-02	2.10E + 01		1.46E-05	2.04E + 01
	Mean	7.44E-99		3.11E-21	5.80E-01	3.00E-20	9.97E + 00	3.16E + 01		9.25E-05	3.97E + 01
	Std.dev	7.44E-98		1.45E-20	2.51E-01	8.18E-20	1.52E + 01	6.14E + 00		1.28E-04	1.38E + 01
	Max	7.44E-97		7.86E-20	1.08E + 00	4.15E-19	5.80E + 01	4.47E + 01		6.82E-04	7.49E + 01
F_71_	Min	2.66E-03		5.34E-05	8.77E + 01	2.87E + 01	4.21E + 01	7.45E + 03		2.78E + 01	1.10E + 04
	Mean	1.07E + 01		1.18E + 01	2.87E + 02	2.88E + 01	1.31E + 05	2.07E + 04		2.85E + 01	4.62E + 04
	Std.dev	1.22E + 01		1.27E + 01	1.80E + 02	2.40E-02	1.36E + 05	1.21E + 04		3.79E-01	2.16E + 04
	Max	2.87E + 01		2.87E + 01	8.62E + 02	2.88E + 01	4.53E + 05	6.15E + 04		2.90E + 01	9.43E + 04
F_72_	Min	4.72E-139		5.08E-36	1.80E + 01	2.17E-23	6.79E + 05	2.13E + 08		1.83E-04	6.94E + 17
	Mean	1.28E-99		3.09E-24	1.96E + 21	1.46E + 12	1.51E + 119	2.40E + 25		2.12E-03	1.01E + 59
	Std.dev	1.28E-98		1.68E-23	1.07E + 22	3.79E + 12	8.31E + 119	1.31E + 26		2.74E-03	5.55E + 59
	Max	1.28E-97		9.25E-23	5.89E + 22	1.21E + 13	4.55E + 120	7.18E + 26		1.47E-02	3.04E + 60
F_73_	Min	6.81E-30		6.14E-08	1.05E + 01	5.62E-10	3.60E + 00	1.77E + 01		2.07E + 00	8.21E + 00
	Mean	3.83E-02		9.33E-03	1.35E + 01	8.96E-01	1.70E + 01	2.27E + 01		4.38E + 00	1.19E + 01
	Std.dev	4.22E-02		2.19E-02	2.27E + 00	2.73E + 00	4.81E + 00	2.01E + 00		1.55E + 00	1.86E + 00
	Max	1.24E-01		1.04E-01	2.11E + 01	1.39E + 01	2.27E + 01	2.56E + 01		7.94E + 00	1.55E + 01
F_74_	Min	2.63E-135		3.54E-34	2.73E + 01	1.03E-60	4.51E + 00	3.76E + 06		3.99E-16	2.84E + 06
	Mean	4.88E-95		2.61E-15	2.65E + 04	2.94E + 08	2.56E + 09	1.07E + 09		1.22E-08	7.72E + 10
	Std.dev	4.58E-94		1.18E-14	7.09E + 04	1.56E + 09	1.29E + 10	2.95E + 09		6.69E-08	3.34E + 11
	Max	4.58E-93		6.47E-14	3.25E + 05	8.60E + 09	7.10E + 10	1.52E + 10		3.66E-07	1.84E + 12
F_75_	Min	2.42E-132		5.35E-38	4.76E + 00	1.18E-34	2.00E-06	4.10E + 05		1.50E-16	2.43E + 06
	Mean	6.41E-95		1.34E-10	2.46E + 04	3.66E + 10	2.49E + 07	2.03E + 09		9.30E-11	8.12E + 12
	Std.dev	6.35E-94		7.37E-10	7.90E + 04	1.37E + 11	5.73E + 07	8.99E + 09		4.20E-10	4.35E + 13
	Max	6.35E-93		4.03E-09	4.29E + 05	7.32E + 11	2.39E + 08	4.91E + 10		2.30E-09	2.38E + 14
F_76_	Min	3.31E-142		2.26E-38	1.17E + 01	1.92E-28	6.03E-01	1.70E + 02		2.02E-04	2.26E + 02
	Mean	6.13E-97		6.76E-21	2.96E + 01	8.84E-18	6.57E + 01	3.40E + 02		8.59E-04	4.88E + 02
	Std.dev	6.05E-96		2.70E-20	1.28E + 01	2.86E-17	8.88E + 01	8.18E + 01		8.78E-04	1.53E + 02
	Max	6.05E-95		1.33E-19	7.08E + 01	1.38E-16	3.19E + 02	5.45E + 02		4.80E-03	8.14E + 02
F_77_	Min	6.46E-136		8.96E-32	2.91E + 05	4.40E-29	1.32E + 04	1.43E + 07		7.51E + 00	1.76E + 07
	Mean	1.11E-98		3.17E-16	5.41E + 05	9.18E-12	4.94E + 06	2.80E + 07		5.99E + 01	3.48E + 07
	Std.dev	1.10E-97		1.66E-15	1.79E + 05	4.96E-11	9.31E + 06	1.02E + 07		4.18E + 01	9.14E + 06
	Max	1.10E-96		9.13E-15	1.15E + 06	2.71E-10	4.64E + 07	6.31E + 07		1.74E + 02	5.48E + 07
F_78_	Min	4.68E-81		1.05E-33	5.66E + 01	3.70E-31	1.57E-02	2.52E + 01		2.61E-05	2.75E + 01
	Mean	2.17E-52		6.39E-01	1.20E + 02	2.23E-17	6.01E + 00	5.24E + 01		2.16E-04	5.14E + 01
	Std.dev	2.08E-51		2.36E + 00	4.06E + 01	1.21E-16	1.04E + 01	1.24E + 01		1.92E-04	1.53E + 01
	Max	2.08E-50		1.19E + 01	2.15E + 02	6.67E-16	5.14E + 01	7.85E + 01		9.77E-04	1.01E + 02
F_79_	Min	1.10E-73		3.33E-18	3.54E + 00	1.08E-18	5.48E-02	1.52E + 01		7.21E-03	1.39E + 01
	Mean	3.58E-54		1.44E-11	6.38E + 00	1.02E-13	6.56E-01	1.98E + 01		1.60E-02	1.97E + 01
	Std.dev	2.90E-53		5.59E-11	2.20E + 00	2.39E-13	7.93E-01	2.96E + 00		7.04E-03	2.78E + 00
	Max	2.81E-52		2.96E-10	1.26E + 01	1.11E-12	3.10E + 00	2.52E + 01		3.53E-02	2.44E + 01
F_80_	Min	2.97E-66		6.68E-21	1.15E + 00	8.72E-02	2.78E + 00	3.02E + 00		2.22E-02	3.60E + 00
	Mean	1.55E-44		2.29E-13	2.36E + 00	2.41E + 00	6.72E + 00	3.94E + 00		5.91E-02	5.11E + 00
	Std.dev	1.39E-43		1.03E-12	6.61E-01	7.73E-01	1.12E + 00	4.47E-01		2.74E-02	5.89E-01
	Max	1.39E-42		5.68E-12	4.31E + 00	3.44E + 00	8.22E + 00	5.05E + 00		1.10E-01	6.20E + 00
F_81_	Min	0.00E + 00		3.30E-209	7.00E-04	1.43E-55	7.58E + 00	7.60E + 03		4.87E-20	2.26E + 05
	Mean	0.00E + 00		6.51E-115	5.09E + 00	9.30E-03	3.11E + 08	9.92E + 05		4.58E-13	3.06E + 06
	Std.dev	0.00E + 00		2.48E-114	1.57E + 01	5.08E-02	4.37E + 08	1.80E + 06		2.38E-12	3.02E + 06
	Max	0.00E + 00		1.06E-113	8.10E + 01	2.78E-01	1.46E + 09	9.82E + 06		1.30E-11	1.02E + 07
F_82_	Min	4.66E-05		8.39E-04	8.04E + 00	1.09E + 01	2.65E + 01	9.03E + 01		6.21E + 00	2.53E + 02
	Mean	2.09E-01		1.72E-01	3.24E + 01	1.85E + 01	1.99E + 03	3.66E + 02		1.12E + 01	5.50E + 02
	Std.dev	2.07E-01		2.14E-01	2.75E + 01	1.65E + 01	2.07E + 03	1.88E + 02		1.89E + 00	2.44E + 02
	Max	9.94E-01		7.95E-01	1.23E + 02	1.04E + 02	7.97E + 03	8.40E + 02		1.60E + 01	1.34E + 03
F_83_	Min	−1.00E + 00		−1.00E + 00	−2.29E-01	−1.00E + 00	−2.29E-01	−5.72E-02		−7.85E-01	−4.16E-02
	Mean	−1.00E + 00		−9.99E-01	−9.29E-01	−8.60E-01	−4.43E-02	−3.09E-02		−6.65E-01	−2.86E-02
	Std.dev	3.72E-09		2.20E-04	4.16E-02	2.11E-01	5.43E-02	1.00E-02		9.34E-02	6.58E-03
	Max	−1.00E + 00		−9.98E-01	−4.31E-02	−9.20E-02	−1.05E-02		−1.8E-02	−4.77E-02	−1.59E-02

		AQSCA	AQUILA	MVO	SPOTTED	SINECOS	JAYA	EQUIL	MOTH
F_84_	Min	−2.26E + 03	−1.77E + 03	9.00E + 02	−1.29E + 02	6.41e + 02	5.44E + 04	−1.67E + 02	1.93E + 05
	Mean	−1.77E + 03	−1.49E + 03	3.27E + 04	−5.08E + 01	9.17E + 04	1.56E + 05	−3.62E + 01	2.82E + 05
	Std.dev	1.89E + 02	1.67E + 02	1.84E + 04	3.23E + 01	1.53E + 05	4.85E + 04	5.69E + 01	6.45E + 04
	Max	−1.38E + 03	−1.20E + 03	8.18E + 04	2.99E + 00	6.08E + 05	2.71E + 05	5.26E + 01	4.29E + 05
F_85_	Min	2.39E-03	5.41E-02	1.01E + 02	2.87E + 01	1.28E + 03	1.01E + 05	2.75E + 01	1.05E + 05
	Mean	2.30E + 01	1.72E + 01	5.22E + 02	2.88E + 01	3.72E + 06	2.45E + 05	2.85E + 01	7.02E + 05
	Std.dev	1.02E + 01	1.24E + 01	3.86E + 02	2.90E-02	4.66E + 06	1.44E + 05	4.01E-01	6.09e + 05
	Max	2.88E + 01	2.87E + 01	1.80E + 03	2.88E + 01	1.71E + 07	6.19E + 05	2.90E + 01	2.27E + 06
F_86_	Min	9.91E-08	2.07E-05	2.61E + 00	1.36E + 00	2.07E + 00	1.78E + 01	4.53E-01	3.36E + 01
	Mean	6.02E-03	8.84E-03	8.55E + 00	1.75E + 00	3.12E + 01	4.02E + 01	7.69E-01	6.18E + 01
	Std.dev	8.92E-03	1.65E-02	4.54E + 00	1.74E-01	2.50E + 01	1.55E + 01	2.29E-01	1.95E + 01
	Max	4.01E-02	7.18E-02	2.34E + 01	1.99E + 00	9.78E + 01	8.27E + 01	1.37E + 00	9.56E + 01
F_87_	Min	1.37E-134	3.45E-38	8.06E-02	2.42E-38	3.40E-04	2.40E + 00	1.90E-06	3.12E + 00
	Mean	7.06E-83	9.57E-25	8.41E-01	2.71E-25	3.76E + 00	4.24E + 00	3.93E-05	6.56E + 00
	Std.dev	7.06E-82	4.92E-24	3.32E + 00	1.05E-24	5.10E + 00	1.13E + 00	6.04E-05	2.37E + 00
	Max	7.06E-81	2.69E-23	1.83E + 01	5.06E-24	1.90E + 01	7.41E + 00	3.21E-04	1.27E + 01
F_88_	Min	9.49E-08	3.26E-06	1.13E + 00	1.51E + 00	1.32E + 01	1.45E + 00	1.61E + 00	2.28E + 00
	Mean	5.48E-04	3.28E-04	1.90E + 00	3.46E + 00	2.14E + 01	2.19E + 00	2.14E + 00	3.16E + 00
	Std.dev	9.07E-04	4.38E-04	2.86E-01	1.06E + 00	3.87E + 00	3.25E-01	4.08E-01	4.59E-01
	Max	4.88E-03	1.50E-03	2.41E + 00	6.24E + 00	3.03E + 01	2.93E + 00	3.37E + 00	4.12E + 00
F_89_	Min	5.19E + 01	7.16E + 01	5.51E + 01	9.71E + 01	9.88E + 01	9.28E + 01	9.03E + 01	1.90E + 02
	Mean	1.09E + 02	1.39E + 02	8.35E + 01	2.15E + 02	3.21E + 02	1.56E + 02	1.26E + 02	2.34E + 02
	Std.dev	2.95E + 01	4.47E + 01	1.57E + 01	6.37E + 01	6.84E + 01	1.94E + 01	2.05E + 01	3.01E + 01
	Max	1.84E + 02	3.08E + 02	1.25E + 02	3.63E + 02	4.26E + 02	1.94E + 02	1.62E + 02	2.95E + 02
F_90_	Min	2.35E-05	2.04E-05	7.22E-02	4.42E-01	3.78E + 01	2.22E-01	1.32E-01	2.80E-01
	Mean	3.07E-02	2.00E-02	3.11E-01	1.58E + 00	8.47E + 00	5.48E-01	5.08E-01	1.03E + 00
	Std.dev	3.06E-02	4.79E-02	1.72E-01	5.66E-01	2.54E + 00	1.92E-01	2.40E-01	3.85E-01
	Max	1.46E-01	2.51E-01	9.45E-01	2.80E + 00	1.45E + 01	9.01E-01	1.18E + 00	1.77E + 00
F_91_	Min	7.13E-02	7.13E-02	4.93E-01	7.47E-02	1.74E-01	1.79E + 00	7.42E-02	3.45E + 00
	Mean	7.13E-02	7.13E-02	8.16E-01	9.29E-02	1.37E + 00	3.02E + 00	8.06E-02	4.87E + 00
	Std.dev	2.32E-05	3.37E-05	2.45E-01	1.69E-02	1.02E + 00	6.29E-01	4.83E-03	9.03E-01
	Max	7.14E-02	7.14E-02	1.42E + 00	1.47E-01	3.58E + 00	4.53E + 00	9.45E-02	7.13E + 00
F_92_	Min	3.03E-01	5.03E-01	9.81E-01	7.49E + 00	2.50E + 01	6.05E + 00	6.03E + 00	1.25E + 01
	Mean	1.85E + 00	2.98E + 00	1.72E + 00	1.21E + 01	8.47E + 01	1.07E + 01	7.93E + 00	2.53E + 01
	Std.dev	1.45E + 00	1.79E + 00	5.14E-01	2.42E + 00	5.71E + 01	3.09E + 00	1.19E + 00	1.04E + 01
	Max	7.61E + 00	7.40E + 00	3.07E + 00	1.66E + 01	2.69E + 02	1.90E + 01	1.15E + 01	5.42E + 01
F_93_	Min	3.09E-91	2.20E-15	8.29E + 02	3.17E-27	3.18E + 01	1.49E + 03	2.88E-03	1.76E + 03
	Mean	7.71E-51	7.25E-08	4.16E + 03	5.51E-10	9.63E + 02	3.39E + 03	3.32E-02	3.52E + 03
	Std.dev	3.93E-50	2.13E-07	3.22E + 03	1.98E-09	1.15E + 03	9.84E + 02	2.71E-02	1.14E + 03
	Max	2.49E-49	1.07E-06	1.14E + 04	9.77E-09	4.04E + 03	5.02E + 03	1.04E-01	6.32E + 03
F_94_	Min	4.68E-265	5.03E-77	1.39E + 02	3.14E-45	7.76E + 03	6.59E + 05	8.50E-06	1.03E + 06
	Mean	8.41E-196	7.64E-37	1.03E + 03	3.25E-18	1.20E + 07	2.33E + 06	3.63E-04	3.80E + 06
	Std.dev	2.76E-196	4.18E-36	7.59E + 02	1.70E-17	1.80E + 07	1.09E + 06	5.07E-04	2.10E + 06
	Max	3.57E-194	2.29E-35	3.43E + 03	9.35E-17	6.90E + 07	4.29E + 06	2.28E-03	9.00E + 06

		AQSCA	AQUILA	MVO	SPOTTED	SINECOS	JAYA	EQUIL	MOTH
F_95_	Min	6.52E-137	5.47E-35	7.40E + 02	9.10E-31	8.19E + 00	2.31E + 04	1.22E-01	2.08E + 05
	Mean	6.03E-102	3.80E-19	2.77E + 03	5.27E-15	4.62E + 03	4.01E + 04	9.05E-02	4.70E + 04
	Std.dev	4.26E-101	2.06E-18	1.44E + 03	2.86E-14	5.69E + 03	9.21E + 03	7.08E-02	1.69E + 04
	Max	3.01E-100	1.13E-17	6.49E + 03	1.57E-13	2.07E + 04	5.98E + 04	2.41E-01	1.14E + 05
F_96_	Min	−4.99E + 00	−5.14E-02	−3.22E + 00	−3.13E-01	−7.64E + 00	7.64E + 02	−4.99E + 00	1.88E + 03
	Mean	−4.98E + 00	−1.41E-02	1.61E + 00	−2.44E-02	1.11E + 03	5.40E + 03	−4.99E + 00	8.77E + 03
	Std.dev	9.89E-03	9.12E-03	3.00E + 00	5.66E-02	2.90E + 03	3.41E + 03	5.05E-03	4.64E + 03
	Max	−4.95E + 00	−9.59E-03	7.99E + 00	−2.76E-04	1.35E + 04	1.43E + 04	−4.97E + 00	1.89E + 04
F_97_	Min	1.20E-01	2.41E-01	5.92E-01	3.39E-01	8.34E-01	7.97E-02	3.29E-01	3.97E-01
	Mean	2.90E-01	4.78E-01	8.79E-01	5.61E-01	1.11E + 01	9.59E-01	5.53E-01	5.74E-01
	Std.dev	1.38E-01	1.39E-01	1.36E-01	1.44E-01	1.43E-01	9.56E-02	9.98E-02	1.29E-01
	Max	7.59E-01	7.91E-01	1.12E + 00	9.69E-01	1.47E + 00	1.13E + 00	7.75E-01	9.01E-01
F_98_	Min	6.60E + 05	6.29E + 06	1.03E + 06	1.80E + 07	2.60E + 07	3.79E + 06	9.54E + 05	8.47E + 06
	Mean	5.36E + 06	8.84E + 06	2.44E + 06	3.76E + 07	4.03E + 07	6.89E + 06	2.76E + 06	1.50E + 07
	Std.dev	6.29E + 06	3.37E + 06	8.71E + 05	1.24E + 07	9.86E + 06	2.02E + 06	1.35E + 06	4.67E + 06
	Max	3.40E + 07	1.79E + 07	3.85E + 06	6.27E + 07	6.97E + 07	1.26E + 07	5.97E + 06	2.51E + 07
F_99_	Min	1.97E-138	1.45E-36	8.83E-04	3.07E-29	6.74E-03	3.01E-02	3.44E-02	6.32E-02
	Mean	5.90E-17	6.90E-22	1.08E-01	5.01E-02	1.27E + 01	2.81E-01	1.73E-01	7.91E-01
	Std.dev	3.67E-16	2.54E-21	1.63E-01	1.17E-01	1.46E + 01	3.65E-01	9.55E-02	7.44E-01
	Max	3.42E-15	1.04E-20	6.14E-01	5.53E-01	6.21E + 01	1.54E + 00	4.59E-01	2.95E + 00
F_100_	Min	1.26E-06	4.90E-07	2.62E + 02	1.02E + 00	3.23E + 00	1.87E + 03	5.64E-01	3.61E + 03
	Mean	9.05E-04	1.13E-03	9.90E + 02	1.70E + 00	4.44E + 03	4.21E + 03	1.25E + 00	6.37E + 03
	Std.dev	3.14E-03	2.56E-03	5.70E + 02	2.60E-01	5.43E + 03	1.67E + 03	4.51E-01	1.88E + 03
	Max	2.80E-02	9.63E-03	2.15E + 03	1.99E + 00	1.80E + 04	1.02E + 04	2.29E + 00	1.21E + 04

**Table 11:** Wilcoxon sum rank test results for 30D unimodal test problems.

	AQSCA	AQUILA	MVO	SPOTTED	SINECOS	JAYA	EQUIL	MOTH
F_70_	N/A	< 0.05	< 0.05	< 0.05	< 0.05	< 0.05	< 0.05	< 0.05
	1	2( +)	5( +)	3( +)	6( +)	7( +)	4( +)	8( +)
F_71_	N/A	0.078	< 0.05	< 0.05	< 0.05	< 0.05	< 0.05	< 0.05
	1	2( +)	5( +)	4( +)	8( +)	6( +)	3( +)	7( +)
F_72_	N/A	< 0.05	< 0.05	< 0.05	< 0.05	< 0.05	< 0.05	< 0.05
	1	2( +)	5( +)	4( +)	8( +)	6( +)	3( +)	7( +)
F_73_	N/A	< 0.05	< 0.05	0.168	< 0.05	< 0.05	< 0.05	< 0.05
	2	1(−)	6( +)	3( +)	7( +)	8( +)	4( +)	5( +)
F_74_	N/A	< 0.05	< 0.05	< 0.05	< 0.05	< 0.05	< 0.05	< 0.05
	1	2( +)	4( +)	5( +)	7( +)	6( +)	3( +)	8( +)
F_75_	N/A	< 0.05	< 0.05	< 0.05	< 0.05	< 0.05	< 0.05	< 0.05
	1	3( +)	4( +)	7( +)	5( +)	6( +)	2( +)	8( +)
F_76_	N/A	< 0.05	< 0.05	< 0.05	< 0.05	< 0.05	< 0.05	< 0.05
	1	2( +)	5( +)	3( +)	6( +)	7( +)	4( +)	8( +)
F_77_	N/A	< 0.05	< 0.05	< 0.05	< 0.05	< 0.05	< 0.05	< 0.05
	1	2( +)	5( +)	3( +)	6( +)	7( +)	4( +)	8( +)
F_78_	N/A	< 0.05	< 0.05	< 0.05	< 0.05	< 0.05	< 0.05	< 0.05
	1	4( +)	8( +)	2( +)	5( +)	7( +)	3( +)	8( +)
F_79_	N/A	< 0.05	< 0.05	< 0.05	< 0.05	< 0.05	< 0.05	< 0.05
	1	3( +)	6( +)	2( +)	5( +)	8( +)	4( +)	7( +)
F_80_	N/A	< 0.05	< 0.05	< 0.05	< 0.05	< 0.05	< 0.05	< 0.05
	1	2( +)	4( +)	5( +)	8( +)	6( +)	3( +)	7( +)
F_81_	N/A	< 0.05	< 0.05	< 0.05	< 0.05	< 0.05	< 0.05	< 0.05
	1	2( +)	5( +)	4( +)	8( +)	6( +)	3( +)	7( +)
F_82_	N/A	< 0.05	< 0.05	< 0.05	< 0.05	< 0.05	< 0.05	< 0.05
	2	1(−)	5( +)	4( +)	8( +)	6( +)	3( +)	7( +)
F_83_	N/A	< 0.05	< 0.05	< 0.05	< 0.05	< 0.05	< 0.05	< 0.05
	1	2( +)	5( +)	3( +)	6( +)	7( +)	4( +)	8( +)
F_84_	N/A	< 0.05	< 0.05	< 0.05	< 0.05	< 0.05	< 0.05	< 0.05
	1	2( +)	5( +)	3( +)	6( +)	7( +)	4( +)	8( +)
F_85_	N/A	< 0.05	< 0.05	< 0.05	< 0.05	< 0.05	< 0.05	< 0.05
	2	1(−)	5( +)	4( +)	8( +)	6( +)	3( +)	7( +)
F_86_	N/A	< 0.05	< 0.05	< 0.05	< 0.05	< 0.05	< 0.05	< 0.05
	1	2( +)	5( +)	4( +)	6( +)	7( +)	3( +)	8( +)
F_87_	N/A	< 0.05	< 0.05	< 0.05	< 0.05	< 0.05	< 0.05	< 0.05
	1	3( +)	5( +)	2( +)	6( +)	7( +)	4( +)	8( +)
F_88_	N/A	< 0.05	< 0.05	< 0.05	< 0.05	< 0.05	< 0.05	< 0.05
	2	1(−)	3( +)	7( +)	8( +)	5( +)	4( +)	6( +)
F_89_	N/A	< 0.05	< 0.05	0.461	< 0.05	0.197	< 0.05	0.08
	2	4( +)	1(−)	6( +)	8( +)	5( +)	3( +)	7( +)
F_90_	N/A	< 0.05	< 0.05	< 0.05	< 0.05	< 0.05	< 0.05	< 0.05
	2	1(−)	3( +)	7( +)	8( +)	5( +)	4( +)	6( +)
F_91_	N/A	< 0.05	< 0.05	0.141	< 0.05	< 0.05	0.09	< 0.05
	1	2( +)	5( +)	4( +)	6( +)	7( +)	3( +)	8( +)
F_92_	N/A	0.277	< 0.05	< 0.05	< 0.05	< 0.05	< 0.05	< 0.05
	2	3( +)	1(−)	6( +)	8( +)	5( +)	4( +)	7( +)
F_93_	N/A	< 0.05	< 0.05	< 0.05	< 0.05	< 0.05	< 0.05	< 0.05
	1	3( +)	8( +)	2( +)	5( +)	6( +)	4( +)	7( +)
F_94_	N/A	< 0.05	< 0.05	< 0.05	< 0.05	< 0.05	< 0.05	< 0.05
	1	2( +)	5( +)	3( +)	8( +)	6( +)	4( +)	7( +)
F_95_	N/A	< 0.05	< 0.05	< 0.05	< 0.05	< 0.05	< 0.05	< 0.05
	1	2( +)	5( +)	3( +)	6( +)	7( +)	4( +)	8( +)
F_96_	N/A	< 0.05	< 0.05	< 0.05	< 0.05	< 0.05	< 0.05	< 0.05
	2	4( +)	5( +)	3( +)	6( +)	7( +)	1(−)	8( +)
F_97_	N/A	0.919	< 0.05	0.313	< 0.05	< 0.05	0.256	0.168
	1	2( +)	6( +)	4( +)	8( +)	7( +)	3( +)	5( +)
F_98_	N/A	0.403	< 0.05	< 0.05	< 0.05	0.903	< 0.05	< 0.05
	3	5( +)	1(−)	7( +)	8( +)	4( +)	2(−)	6( +)
F_99_	N/A	< 0.05	< 0.05	< 0.05	< 0.05	0.903	< 0.05	< 0.05
	2	1(−)	4( +)	3( +)	8( +)	6( +)	5( +)	7( +)
F_100_	N/A	< 0.05	< 0.05	< 0.05	< 0.05	0.903	< 0.05	< 0.05
	1	2( +)	5( +)	4( +)	7( +)	6( +)	3( +)	8( +)
Aver. rank	1.35	2.25	4.64	4.00	6.87	6.32	3.38	7.22
Ranking	1	2	5	4	7	6	3	8
+ / = / −		26/0/5	28/0/3	31/0/0	31/0/0	31/0/0	29/0/2	31/0/0

**Table 12:** Statistical results for 500D benchmark functions.

		AQSCA	AQUILA	MVO	SPOTTED	SINECOS	JAYA	EQUIL	MOTH
F_1_	Min	8.88E-16	8.88E-16	1.13E + 01	8.88E-16	1.35E + 01	8.28E + 00	2.77E-01	1.12E + 01
	Mean	9.59E-16	1.53E-13	1.18E + 01	3.21E-10	1.42E + 01	8.72E + 00	4.21E-01	1.20E + 01
	Std.dev	5.02E-16	8.09E-14	1.96E-01	1.04E-09	2.89E-01	2.26E-01	1.09E-01	1.58E-01
	Max	4.44E-15	4.43E-12	1.22E + 01	5.39E-09	1.46E + 01	9.26E + 00	6.64E-01	1.24E + 01
F_2_	Min	0.00E + 00	0.00E + 00	2.18E + 00	0.00E + 00	3.30E + 00	1.49E + 00	4.22E-03	2.51E + 00
	Mean	0.00E + 00	0.00E + 00	2.31E + 00	3.70E-18	4.12E + 00	1.57E + 00	1.33E-02	2.65E + 00
	Std.dev	0.00E + 00	0.00E + 00	6.80E-02	2.02E-17	3.21E-01	4.55E-02	2.12E-02	6.85E-02
	Max	0.00E + 00	0.00E + 00	2.45E + 01	1.11E-16	4.62E + 00	1.69E + 00	1.21E-01	2.80E + 00
F_3_	Min	0.00E + 00	0.00E + 00	1.11E + 04	0.00E + 00	1.47E + 04	6.86E + 03	3.05E + 02	1.05E + 04
	Mean	0.00E + 00	0.00E + 00	1.21E + 04	1.21E-13	1.71E + 04	7.25E + 03	5.08E + 02	1.13E + 04
	Std.dev	0.00E + 00	0.00E + 00	5.47E + 02	3.94E-13	1.04E + 03	2.06E + 02	1.43E + 02	2.86E + 02
	Max	0.00E + 00	0.00E + 00	1.31E + 04	1.81E-12	1.85E + 04	7.68E + 03	8.48E + 02	1.19E + 04
F_4_	Min	7.65E-34	1.32E-32	1.18E + 04	7.82E + 02	7.93E + 03	1.04E + 04	3.79E + 03	1.05E + 04
	Mean	1.00E + 03	1.02E + 05	1.33E + 04	5.15E + 04	1.41Ê + 06	3.48E + 04	5.84E + 03	1.20E + 04
	Std.dev	2.33E + 03	3.96E + 05	8.56E + 02	1.20E + 05	5.95E + 06	5.39E + 04	1.26E + 03	9.76E + 02
	Max	1.21E + 04	1.93E + 06	1.60E + 04	5.69E + 05	3.22E + 07	2.44E + 05	8.84E + 03	1.46E + 04
F_5_	Min	1.81E-74	3.32E-18	8.30E + 02	1.69E-19	6.47E + 02	5.70E + 02	1.18E + 00	8.03E + 02
	Mean	7.21E-05	8.64E-04	8.91E + 02	6.73E-12	1.22E + 03	6.01E + 02	3.04E + 00	8.56E + 02
	Std.dev	2.80E-02	3.80E-03	4.61E + 01	2.35E-11	1.35E + 02	1.97E + 01	1.38E + 00	2.76E + 01
	Max	1.58E-01	2.06E-02	1.00E + 03	1.10E-10	1.35E + 03	6.51E + 02	7.64E + 00	9.09E + 02
F_6_	Min	8.91E-08	1.72E-09	1.19E + 01	1.31E-01	3.22E + 01	9.86E + 00	8.87E-01	1.28E + 01
	Mean	4.61E-07	5.98E-06	1.38E + 01	2.02E-01	3.50E + 01	1.09E + 01	9.48E-01	1.46E + 01
	Std.dev	9.98E-06	9.41E-06	1.14E + 00	5.40E-02	1.41E + 00	6.20E-01	3.16E-02	8.46E-01
	Max	6.50E-05	3.42E-05	1.62E + 01	3.54E-01	3.77E + 01	1.25E + 01	1.00E + 00	1.63E + 01
F_7_	Min	0.00E + 00	8.25E-123	1.35E + 07	5.60E-57	9.94E + 07	1.46E + 06	7.69E-01	1.68E + 07
	Mean	0.00E + 00	4.41E-67	1.74E + 07	1.50E-08	1.13E + 08	2.29E + 06	6.63E + 00	2.15E + 07
	Std.dev	0.00E + 00	2.41E-66	2.35E + 06	8.11E-08	6.34E + 06	6.38E + 05	7.10E + 00	2.55E + 06
	Max	0.00E + 00	1.32E-65	2.24E + 07	4.44E-07	1.22E + 08	4.32E + 06	3.64E + 01	2.73E + 07
F_8_	Min	0.00E + 00	0.00E + 00	1.67E + 02	0.00E + 00	2.09E + 02	2.16E + 02	1.91E + 02	1.83E + 02
	Mean	0.00E + 00	0.00E + 00	1.73E + 02	1.07E-03	2.17E + 02	2.20E + 02	2.04E + 02	1.93E + 02
	Std.dev	0.00E + 00	0.00E + 00	3.66E + 00	5.86E-03	3.48E + 00	2.24E + 00	5.67E + 00	5.97E + 00
	Max	0.00E + 00	0.00E + 00	1.80E + 02	3.21E-02	2.23E + 02	2.24E + 02	2.14E + 02	2.08E + 02
F_9_	Min	4.44E-72	1.92E-18	8.68E + 00	9.29E-10	9.00E + 00	5.86E + 00	1.39E + 00	8.52E + 00
	Mean	1.59E-04	2.01E-03	9.23E + 00	8.38E-01	9.91E + 00	6.30E + 00	1.66E + 00	8.91E + 00
	Std.dev	3.69E-02	1.00E-02	2.89E-01	1.29E + 00	4.00E-01	3.09E-01	1.82E-01	1.74E-01
	Max	9.98E-02	5.48E-02	9.79E + 00	3.79E + 00	1.07E + 01	6.94E + 00	2.09E + 00	9.24E + 00
F_10_	Min	4.40E-131	1.37E-39	4.72E + 03	4.35E-32	9.67E + 03	1.90E + 03	9.96E + 00	6.29E + 03
	Mean	4.08E-95	6.15E-23	5.30E + 03	9.65E-18	1.23E + 04	2.38E + 03	1.55E + 01	6.67E + 03
	Std.dev	2.67E-94	3.21E-22	2.71E + 02	4.91E-17	1.01E + 03	1.89E + 02	2.64E + 00	2.13E + 02
	Max	1.89E-93	1.76E-21	5.90E + 03	2.69E-16	1.45E + 04	2.83E + 03	2.18E + 01	7.18E + 03
F_11_	Min	0.00E + 00	0.00E + 00	9.26E-01	0.00E + 00	6.57E-02	9.46E-01	6.79E-01	8.94E-01
	Mean	0.00E + 00	0.00E + 00	9.37E-01	6.39E-04	1.30E-01	9.61E-01	8.45E-01	9.19E-01
	Std.dev	0.00E + 00	0.00E + 00	5.02E-03	3.57E-03	4.28E-02	4.36E-03	6.66E-02	8.16E-03
	Max	0.00E + 00	0.00E + 00	9.49E-01	1.91E-02	2.79E-01	9.68E-01	9.30E-01	9.34E-01
F_12_	Min	2.42E-36	1.79E-09	2.21E + 143	4.86E + 95	N/A	N/A	2.99E-12	N/A
	Mean	2.09E-22	8.76E-06	2.16E + 255	7.20E + 241	N/A	N/A	3.60E-07	N/A
	Std.dev	1.48E-21	2.47E-05	2.34E + 255	6.62E + 241	N/A	N/A	1.57E-06	N/A
	Max	1.04E-20	1.21E-04	6.48E + 256	2.16E + 243	N/A	N/A	8.62E-06	N/A
F_13_	Min	4.46E-215	4.46E-215	1.18E-62	1.73E-106	1.44E-57	3.58E-50	3.79E-93	1.01E-77
	Mean	4.72E-215	4.79E-215	5.03E-45	6.83E-73	1.37E-46	3.24E-38	1.08E-76	8.97E-57
	Std.dev	0.00E + 00	0.00E + 00	2.07E-44	3.74E-72	6.21E-46	1.69E-37	5.91E-76	4.57E-56
	Max	7.93E-215	6.63E-215	1.09E-43	2.05E-71	3.38E-45	9.28E-37	3.24E-75	2.50E-55
F_14_	Min	−1.00E + 00	−1.00E + 00	5.76E-169	−1.00E + 00	1.78E-156	1.22E-165	1.18E-177	8.86E-165
	Mean	−9.99E-01	−6.28E-01	4.47E-158	−4.99E-01	1.72E-152	5.43E-156	1.03E-170	2.34E-156
	Std.dev	4.77E-03	4.84E-01	2.44E-157	5.07E-01	3.91E-152	2.88E-155	0.00E + 00	1.04E-155
	Max	−9.66E-01	−1.77E-06	1.34E-156	1.00E-162	1.52E-151	1.57E-154	2.72E-169	5.65E-155
F_15_	Min	0.00E + 00	0.00E + 00	2.15E + 02	0.00E + 00	2.25E + 02	2.27E + 02	2.18E + 02	2.02E + 02
	Mean	5.77E-04	1.09E-03	2.21E + 02	1.54E + 01	2.29E + 02	2.31E + 02	2.25E + 02	2.11E + 02
	Std.dev	2.97E-02	4.13E-03	2.94E + 00	5.72E + 01	2.15E + 00	1.44E + 00	2.96E + 00	4.11E + 00
	Max	2.07E-01	2.06E-02	2.27E + 02	2.29E + 02	2.33E + 02	2.34E + 02	2.30E + 02	2.19E + 02

		AQSCA	AQUILA	MVO	SPOTTED	SINECOS	JAYA	EQUIL	MOTH
F_16_	Min	1.29E + 00	7.99E-01	9.46E + 05	6.96E + 02	6.12E + 06	1.20E + 05	1.81E + 03	1.30E + 06
	Mean	7.71E + 00	8.83E + 00	1.25E + 06	8.52E + 02	6.90E + 06	1.99E + 05	1.88E + 03	1.60E + 06
	Std.dev	7.30E + 00	9.53E + 00	1.50E + 05	8.74E + 01	4.26E + 05	5.22E + 04	4.03E + 01	1.23E + 05
	Max	4.04E + 01	4.64E + 01	1.60E + 06	1.01E + 03	7.63E + 06	3.49E + 05	1.97E + 03	1.78E + 06
F_17_	Min	4.96E-04	8.41E-05	1.96E + 03	1.36E + 01	3.58E + 03	3.76E + 02	1.40E + 02	2.01E + 03
	Mean	1.22E-01	8.13E-02	2.20E + 03	2.85E + 01	4.12E + 03	5.19E + 02	1.48E + 02	2.27E + 03
	Std.dev	1.20E-01	2.04E-01	1.81E + 02	7.96E + 00	3.01E + 02	6.64E + 01	3.74E + 00	1.07E + 02
	Max	4.60E-01	1.11E + 00	2.75E + 03	4.71E + 01	4.75E + 03	6.94E + 02	1.55E + 02	2.45E + 03
F_18_	Min	3.09E + 07	3.05E + 07	3.30E + 07	3.11E + 07	3.41E + 07	3.33E + 07	3.35E + 07	3.32E + 07
	Mean	3.19E + 07	3.19E + 07	3.35E + 07	3.20E + 07	3.49E + 07	3.36E + 07	3.37E + 07	3.37E + 07
	Std.dev	4.85E + 05	5.67E + 05	1.93E + 05	4.80E + 05	3.89E + 05	1.84E + 05	1.58E + 05	1.76E + 05
	Max	3.31E + 07	3.28E + 07	3.38E + 07	3.30E + 07	3.56E + 07	3.39E + 07	3.41E + 07	3.41E + 07
F_19_	Min	−5.66E + 05	−4.90E + 05	−9.66E + 04	−3.24E + 05	2.64E + 05	−2.59E + 05	−1.27E + 05	−9.15E + 04
	Mean	−4.20E + 05	−4.19E + 05	−7.26E + 04	−2.77E + 05	3.74E + 05	−2.35E + 05	−1.07E + 05	−4.88E + 04
	Std.dev	4.29E + 04	4.87E + 04	1.40E + 04	3.08E + 04	4.48E + 04	1.06E + 04	8.45E + 03	2.08E + 04
	Max	−3.17E + 05	−2.86E + 05	−4.77E + 04	−1.91E + 05	4.43E + 05	−2.14E + 05	−9.43E + 04	3.34E + 01
F_20_	Min	−1.27E + 04	−1.27E + 04	2.02E + 04	−7.73E + 03	2.60E + 05	−3.98E + 03	−1.61E + 03	2.84E + 04
	Mean	−1.13E + 04	−1.12E + 04	2.78E + 04	−6.47E + 03	3.76E + 05	−2.25E + 03	−1.11E + 03	4.74E + 04
	Std.dev	1.08E + 03	1.04E + 03	4.64E + 03	1.01E + 03	3.50E + 04	1.03E + 03	2.08E + 02	7.79E + 03
	Max	−8.27E + 03	−8.99E + 03	4.00E + 04	−2.34E + 03	4.49E + 05	4.49E + 02	−5.22E + 02	6.22E + 04
F_21_	Min	8.22E-133	5.43E-38	6.74E + 04	6.44E-30	1.53E + 05	4.46E + 04	1.40E + 01	1.19E + 05
	Mean	1.01E-92	1.72E-27	8.31E + 04	3.47E-17	2.46E + 05	5.30E + 04	2.73E + 01	1.33E + 05
	Std.dev	6.89E-92	8.91E-27	7.85E + 03	1.72E-16	4.55E + 04	5.95E + 03	9.99E + 00	6.07E + 03
	Max	4.87E-91	4.89E-26	1.05E + 05	9.49E-16	3.18E + 05	7.01E + 04	6.27E + 01	1.44E + 05
F_22_	Min	2.82E-142	1.21E-35	1.15E + 04	1.82E + 03	1.30E + 04	1.62E + 04	6.22E + 00	1.53E + 04
	Mean	1.71E-108	3.40E-22	1.33E + 04	2.35E + 03	2.02E + 04	2.27E + 04	1.39E + 01	1.70E + 04
	Std.dev	1.14E-107	1.65E-21	8.78E + 02	3.40E + 02	2.82E + 03	3.51E + 03	4.91E + 00	9.74E + 02
	Max	8.08E-107	9.07E-21	1.51E + 04	3.06E + 03	2.53E + 04	2.94E + 04	2.57E + 01	1.94E + 04
F_23_	Min	−4.98E + 02	−4.97E + 02	−1.22E + 02	−1.01E + 02	−7.21E + 01	−7.43E + 01	−9.92E + 01	−1.42E + 02
	Mean	−4.59E + 02	−4.79E + 02	−9.57E + 01	−6.93E + 01	−6.09E + 01	−5.87E + 01	−7.61E + 01	−1.19E + 02
	Std.dev	7.11E + 01	2.42E + 01	1.08E + 01	1.04E + 01	5.40E + 00	6.08E + 00	9.67E + 00	1.16E + 01
	Max	−6.35E + 01	−4.03E + 02	−7.09E + 01	−5.40E + 01	−5.12E + 01	−5.03E + 01	−5.17E + 01	−9.84E + 01
F_24_	Min	−4.74E + 05	−4.15E + 05	−1.13E + 05	−3.10E + 05	2.89E + 05	−2.53E + 05	−1.25E + 05	−7.30E + 04
	Mean	−3.48E + 05	−3.47E + 05	−5.78E + 04	−2.47E + 05	4.30E + 05	−2.34E + 05	−1.09E + 05	−4.15E + 04
	Std.dev	5.00E + 04	3.44E + 04	2.61E + 04	3.74E + 04	5.87E + 04	9.86E + 03	8.39E + 03	1.78E + 04
	Max	−2.55E + 05	−2.79E + 05	−6.23E + 03	−1.79E + 05	5.41E + 05	−2.08E + 05	−9.62E + 04	−1.05E + 04
F_25_	Min	−4.98E + 02	−4.98E + 02	−1.16E + 02	−9.28E + 01	−7.56E + 01	−6.93E + 01	−1.00E + 02	−1.43E + 02
	Mean	−4.98E + 02	−4.98E + 02	−9.80E + 01	−7.12E + 01	−6.17E + 01	−5.85E + 01	−7.61E + 01	−1.21E + 02
	Std.dev	9.96E-01	9.52E-01	8.68E + 00	7.46E + 00	6.37E + 00	4.48E + 00	1.02E + 01	1.07E + 01
	Max	−4.94E + 02	−4.94E + 02	−7.76E + 01	−5.77E + 01	−5.00E + 01	−4.91E + 01	−6.14E + 01	−9.59E + 01
F_26_	Min	−1.91E + 02	−8.07E + 01	1.31E + 05	4.96E + 02	2.50E + 06	1.03E + 05	−3.47E + 02	2.23E + 05
	Mean	−9.71E + 01	−9.54E + 00	1.97E + 05	3.34E + 03	3.18E + 06	1.95E + 05	−2.31E + 02	3.19E + 05
	Std.dev	4.87E + 01	1.93E + 01	4.75E + 04	1.90E + 03	3.24E + 05	4.67E + 04	5.62E + 01	6.62E + 04
	Max	−2.50E-01	−2.47E-01	3.02E + 05	8.59E + 03	3.66E + 06	3.17E + 05	−1.27E + 02	4.68E + 05
F_27_	Min	4.24E-03	2.55E-03	4.33E + 05	3.26E + 02	1.71E + 06	7.33E + 04	4.79E + 02	5.07E + 05
	Mean	8.99E + 00	8.44E + 00	5.59E + 05	3.85E + 02	2.46E + 06	1.18E + 05	5.11E + 02	6.20E + 05
	Std.dev	1.14E + 01	1.30E + 01	8.10E + 04	3.14E + 01	2.02E + 05	2.38E + 04	1.44E + 01	5.48E + 04
	Max	5.06E + 01	5.78E + 01	7.69E + 05	4.41E + 02	2.84E + 06	1.66E + 05	5.44E + 02	7.55E + 05
F_28_	Min	8.56E-12	5.73E-11	1.20E + 05	9.07E-01	2.49E + 05	2.06E + 04	4.71E + 00	1.24E + 05
	Mean	2.88E-06	3.93E-05	1.32E + 05	1.23E + 01	2.93E + 05	4.17E + 04	1.45E + 01	1.40E + 05
	Std.dev	5.12E-05	7.89E-05	7.62E + 03	4.53E + 01	2.33E + 04	1.24E + 04	9.60E + 00	1.05E + 04
	Max	2.13E-03	3.49E-04	1.46E + 05	2.23E + 02	3.31E + 05	6.71E + 04	3.80E + 01	1.59E + 05
F_29_	Min	−1.06E + 04	−9.21E + 03	−5.16E + 03	−8.11E + 03	−3.76E + 03	−5.08E + 03	−5.02E + 03	−5.07E + 03
	Mean	−8.23E + 03	−8.03E + 03	−4.95E + 03	−7.23E + 03	−3.52E + 03	−4.91E + 03	−4.84E + 03	−4.91E + 03
	Std.dev	7.96E + 02	5.86E + 02	9.90E + 01	3.86E + 02	1.34E + 02	9.20E + 01	8.68E + 01	9.28E + 01
	Max	−7.09E + 03	−6.45E + 03	−4.80E + 03	−6.57E + 03	−3.25E + 03	−4.76E + 03	−4.66E + 03	−4.75E + 03

		AQSCA	AQUILA	MVO	SPOTTED	SINECOS	JAYA	EQUIL	MOTH
F_30_	Min	2.40E-02	2.40E-02	2.80E-02	2.47E-02	2.76E-02	2.63E-02	2.56E-02	2.78E-02
	Mean	2.40E-02	2.40E-02	2.85E-02	2.52E-02	2.85E-02	2.66E-02	2.57E-02	2.80E-02
	Std.dev	7.68E-08	5.60E-08	1.97E-04	2.19E-04	3.52E-04	1.46E-04	7.19E-05	1.18E-04
	Max	2.40E-02	2.40E-02	2.89E-02	2.56E-02	2.91E-02	2.70E-02	2.59E-02	2.82E-02
F_31_	Min	−2.97E + 03	−2.94E + 03	5.12E + 02	−2.95E + 03	−2.95E + 03	−2.07E + 01	−2.83E + 03	4.14E + 02
	Mean	−2.94E + 03	−2.94E + 03	6.41E + 02	−2.94E + 03	−2.93E + 03	8.19E + 01	−2.79E + 03	5.05E + 02
	Std.dev	5.42E + 00	4.62E-13	6.85E + 01	2.43E + 00	1.34E + 01	4.37E + 01	2.31E + 01	3.87E + 01
	Max	−2.94E + 03	−2.94E + 03	8.15E + 02	−2.94E + 03	−2.90E + 03	1.75E + 02	−2.74e + 03	5.53E + 02
F_32_	Min	−1.18E + 03	−1.22E + 03	−3.87E + 02	−3.38E + 02	−3.16E + 02	−3.05E + 02	−3.78E + 02	−4.12E + 02
	Mean	−9.46E + 02	−9.71E + 02	−3.57E + 02	−3.07E + 02	−3.01E + 02	−2.88E + 02	−3.40E + 02	−3.79E + 02
	Std.dev	1.34E + 02	1.12E + 02	2.11E + 00	1.36E + 01	7.35E + 00	6.48E + 00	1.67E + 01	1.93E + 01
	Max	−6.92E + 02	−7.70E + 02	−3.13E + 02	−2.84E + 02	−2.88E + 02	−2.76E + 02	−3.08E + 02	−3.33E + 02
F_33_	Min	4.13E + 02	2.84E + 02	4.54E + 02	4.21E + 02	4.65E + 02	4.66E + 02	4.55E + 02	4.56E + 02
	Mean	4.39E + 02	4.49E + 02	4.60E + 02	4.44E + 02	4.67E + 02	4.68E + 02	4.61E + 02	4.61E + 02
	Std.dev	7.03E + 00	3.67E + 01	3.19E + 00	9.63E + 00	1.35E + 00	8.51E-01	2.86E + 00	2.60E + 00
	Max	4.66E + 02	4.66E + 02	4.67E + 02	4.58E + 02	4.69E + 02	4.69E + 02	4.66E + 02	4.67E + 02
F_34_	Min	1.19E + 03	1.19E + 03	4.58E + 04	1.19E + 03	4.65E + 04	4.67E + 04	4.62E + 04	4.58E + 04
	Mean	1.19E + 03	1.39E + 03	4.62E + 04	2.24E + 03	4.67E + 04	4.68E + 04	4.64E + 04	4.60E + 04
	Std.dev	9.18E-13	5.04E + 02	1.76E + 02	2.78E + 03	7.78E + 01	5.99E + 01	1.34E + 02	1.22E + 02
	Max	1.19E + 03	3.77E + 03	4.65E + 04	1.60E + 04	4.68E + 04	4.69E + 04	4.67E + 04	4.63E + 04
F_35_	Min	2.70E + 04	2.80E + 04	2.54E + 04	2.81E + 04	2.89E + 04	2.91E + 04	2.72E + 04	2.61E + 04
	Mean	2.82E + 04	2.92E + 04	2.64E + 04	2.94E + 04	2.96E + 04	3.00E + 04	2.82E + 04	2.70E + 04
	Std.dev	3.96E + 02	5.69E + 02	4.39E + 02	4.83E + 02	2.73E + 02	2.71E + 02	5.81E + 02	5.17E + 02
	Max	2.90E + 04	3.01E + 04	2.73E + 04	3.00E + 04	3.01E + 04	3.05E + 04	2.93E + 04	2.82E + 04
F_36_	Min	5.78E + 02	5.80E + 02	1.44E + 03	5.83E + 02	4.02E + 03	9.22E + 02	5.59E + 02	1.55E + 03
	Mean	5.83E + 02	5.83E + 02	1.75E + 03	5.96E + 02	4.21E + 03	1.03E + 03	5.68E + 02	1.82E + 03
	Std.dev	1.04E + 00	9.70E-01	1.26E + 02	3.86E + 01	1.08E + 02	6.61E + 01	4.93E + 00	1.22E + 02
	Max	5.83E + 02	5.83E + 02	1.97E + 03	7.16E + 02	4.44E + 03	1.21E + 03	5.81E + 02	2.10E + 03
F_37_	Min	1.28E-04	1.03E-04	3.18E + 04	2.39E + 02	1.11E + 04	3.34E + 04	3.71E + 03	3.15E + 04
	Mean	2.77E-02	3.34E-02	3.25E + 04	2.39E + 02	3.28E + 04	3.41E + 04	6.94E + 03	3.24E + 04
	Std.dev	3.28E-02	5.90E-02	4.10E + 02	2.71E-11	5.05E + 03	3.35E + 02	2.89E + 03	5.79E + 02
	Max	1.54E-01	3.17E-01	3.35E + 04	2.39E + 02	3.55E + 04	3.49E + 04	1.69E + 04	3.39E + 04
F_38_	Min	−3.53E + 02	−2.49E + 02	−3.21E + 01	−1.92E + 01	−4.96E + 00	−3.53E + 00	−1.10E + 01	−8.20E + 00
	Mean	−1.78E + 02	−1.91E + 02	−2.39E + 00	−1.25E + 01	−3.24E + 00	−2.42E + 00	−6.62E + 00	−6.18E + 00
	Std.dev	4.75E + 01	4.11E + 01	−3.93E-01	2.70E + 00	5.49E-01	3.46E-01	1.86E + 00	1.07E + 00
	Max	−1.03E + 02	−1.03E + 02	−1.73E + 00	−8.12E + 00	−2.19E + 00	−1.98E + 00	−3.56E + 00	−3.81E + 00
F_39_	Min	−1.41E + 03	−1.17E + 03	−6.92E + 02	−8.94E + 02	−6.42E + 02	−6.04E + 02	−7.24E + 02	−7.12E + 02
	Mean	−1.03E + 03	−9.83E + 02	−6.53E + 02	−7.93E + 02	−6.09E + 02	−5.82E + 02	−6.92E + 02	−6.82E + 02
	Std.dev	1.07E + 02	8.79E + 01	2.12E + 01	5.92E + 01	1.21E + 01	9.33E + 00	1.85E + 01	1.68E + 01
	Max	−7.76E + 02	−7.74E + 02	−6.10E + 02	−6.23E + 02	−5.90E + 02	−5.66E + 02	−6.47E + 02	−6.37E + 02
F_40_	Min	4.99E + 02	4.99E + 02	4.64E + 04	4.99E + 02	3.73E + 04	4.63E + 04	3.11E + 04	4.63E + 04
	Mean	4.99E + 02	1.28E + 03	4.67E + 04	1.12E + 03	4.63E + 04	4.66E + 04	3.17E + 04	4.65E + 04
	Std.dev	0.00E + 00	8.50E + 02	1.78E + 02	8.08E + 02	2.60E + 03	1.47E + 02	4.85E + 02	1.23E + 02
	Max	4.99E + 02	3.06E + 03	4.71E + 04	3.30E + 03	4.76E + 04	4.68E + 04	3.29E + 04	4.67E + 04
F_41_	Min	N/A	N/A	N/A	N/A	N/A	N/A	N/A	N/A
	Mean	N/A	N/A	N/A	N/A	N/A	N/A	N/A	N/A
	Std.dev	N/A	N/A	N/A	N/A	N/A	N/A	N/A	N/A
	Max	N/A	N/A	N/A	N/A	N/A	N/A	N/A	N/A
F_42_	Min	N/A	N/A	N/A	N/A	N/A	N/A	N/A	N/A
	Mean	N/A	N/A	N/A	N/A	N/A	N/A	N/A	N/A
	Std.dev	N/A	N/A	N/A	N/A	N/A	N/A	N/A	N/A
	Max	N/A	N/A	N/A	N/A	N/A	N/A	N/A	N/A
F_43_	Min	N/A	N/A	N/A	N/A	N/A	N/A	N/A	N/A
	Mean	N/A	N/A	N/A	N/A	N/A	N/A	N/A	N/A
	Std.dev	N/A	N/A	N/A	N/A	N/A	N/A	N/A	N/A
	Max	N/A	N/A	N/A	N/A	N/A	N/A	N/A	N/A
F_44_	Min	0.00E + 00	0.00E + 00	2.54E-01	5.35E-38	8.57E-01	3.76E-01	2.86E-02	3.44E-01
	Mean	3.70E-125	0.00E + 00	3.54E-01	4.78E-03	1.00E + 00	5.69E-01	3.54E-02	4.39E-01
	Std.dev	2.62E-124	0.00E + 00	5.24E-02	4.66E-03	6.13E-02	1.72E-01	4.41E-03	4.50E-02
	Max	1.85E-123	0.00E + 00	4.47E-01	1.43E-02	1.11E + 00	9.95E-01	4.80E-02	5.08E-01

		AQSCA	AQUILA	MVO	SPOTTED	SINECOS	JAYA	EQUIL	MOTH
F_45_	Min	−9.99E-01	−9.99E-01	−5.32E-01	−8.61E-01	−1.07E-01	−5.18E-01	−5.63E-01	−5.12E-01
	Mean	−9.87E-01	−9.85E-01	−4.84E-01	−8.21E-01	−8.21E-02	−4.81E-01	−5.39E-01	−4.85E-01
	Std.dev	2.42E-02	2.87E-02	2.81E-02	2.43E-02	1.44E-02	1.85E-02	1.63E-02	1.80E-02
	Max	−8.79E-01	−8.89E-01	−4.08E-01	−7.64E-01	−5.53E-02	−4.44E-01	−5.09E-01	−4.46E-01
F_46_	Min	−9.93E-01	−9.77E-01	0.00E + 00	−2.40E-46	0.00E + 00	0.00E + 00	0.00E + 00	0.00E + 00
	Mean	−4.60E-01	−4.18E-01	0.00E + 00	−8.02E-48	0.00E + 00	0.00E + 00	0.00E + 00	0.00E + 00
	Std.dev	4.02E-01	3.37E-01	0.00E + 00	4.36E-47	0.00E + 00	0.00E + 00	0.00E + 00	0.00E + 00
	Max	−5.36E-19	−7.61E-03	0.00E + 00	0.00E + 00	0.00E + 00	0.00E + 00	0.00E + 00	0.00E + 00
F_47_	Min	5.70E + 02	7.70E + 02	6.71E + 03	4.36E + 03	7.54E + 03	7.78E + 03	6.67E + 03	6.81E + 03
	Mean	1.84E + 03	1.93E + 03	7.01E + 03	5.28E + 03	7.77E + 03	7.89E + 03	6.96E + 03	7.10E + 03
	Std.dev	3.96E + 02	3.87E + 02	1.34E + 02	3.22E + 02	9.48E + 01	7.22E + 01	1.39E + 02	1.17E + 02
	Max	2.69E + 03	2.69E + 03	7.33E + 03	5.77E + 03	8.00E + 03	8.04E + 03	7.27E + 03	7.32E + 03
F_48_	Min	4.83E + 01	2.29E + 01	2.36E + 03	3.78E + 02	4.12E + 03	3.61E + 03	2.59E + 03	2.83E + 03
	Mean	4.59E + 02	2.48E + 02	2.59E + 03	6.67E + 02	4.62E + 03	3.89E + 03	2.68E + 03	3.18E + 03
	Std.dev	3.34E + 02	1.83E + 02	1.22E + 02	1.30E + 02	2.16E + 02	9.17E + 01	4.20E + 01	1.29E + 02
	Max	1.11E + 03	8.09E + 02	2.79E + 03	9.40E + 02	5.08E + 03	4.04E + 03	2.76E + 03	3.47E + 03
F_49_	Min	−1.18E + 02	−1.13E + 02	−8.83E + 01	−1.11E + 02	−8.39E + 01	−9.54E + 01	−1.01E + 02	−1.15E + 02
	Mean	−1.09E + 02	−1.03E + 02	−8.34E + 01	−1.06E + 02	−7.99E + 01	−8.71E + 01	−9.40E + 01	−1.06E + 02
	Std.dev	2.77E + 00	3.77E + 00	2.46E + 00	2.58E + 00	1.90E + 00	3.34E + 00	3.27E + 00	4.22E + 00
	Max	−1.04E + 02	−9.77E + 01	−7.82E + 01	−1.02E + 02	−7.54E + 01	−8.06E + 01	−8.86E + 01	−9.82E + 01
F_50_	Min	5.50E-125	1.80E-36	1.26E + 06	6.36E-27	3.22E + 06	5.87E + 05	3.92E + 02	1.68E + 06
	Mean	1.09E-92	8.28E-20	1.42E + 06	2.43E-15	3.99E + 06	6.54E + 05	8.17E + 02	1.86E + 06
	Std.dev	7.13E-82	4.36E-19	8.45E + 04	7.76E-15	3.67E + 05	4.25E + 04	2.34E + 02	8.14E + 04
	Max	5.04E-91	2.39E-18	1.67E + 06	3.65E-14	4.60E + 06	7.98E + 05	1.35E + 03	1.99E + 06
F_51_	Min	1.90E-107	2.87E-39	2.33E + 06	8.91E-32	2.77E + 06	1.58E + 06	9.95E + 04	2.68E + 06
	Mean	2.39E-01	3.17E-24	2.50E + 06	5.61E-18	4.31E + 06	1.75E + 06	1.47E + 05	2.91E + 06
	Std.dev	1.69E + 00	1.44E-23	6.79E + 04	2.78E-17	4.90E + 05	6.62E + 04	2.88E + 04	9.09E + 04
	Max	1.19E + 01	7.81E-23	2.62E + 06	1.52E-16	4.90E + 06	1.90E + 06	2.02E + 05	3.16E + 06
F_52_	Min	−8.42E-01	−9.78E-01	1.34E + 02	1.81E + 01	3.39E + 02	5.31E + 01	9.88E + 01	1.48E + 02
	Mean	6.00E-02	−3.96E-01	1.47E + 02	2.57E + 01	3.54E + 02	6.17E + 01	1.14E + 02	1.63E + 02
	Std.dev	8.04E-01	4.54E-01	6.51E + 00	3.22E + 00	9.85E + 00	6.31E + 00	6.79E + 00	6.58E + 00
	Max	2.91E + 00	8.90E-01	1.59E + 02	3.17E + 01	3.74E + 02	8.01E + 01	1.30E + 02	1.78E + 02
F_53_	Min	−9.99E-01	−9.99E-01	−9.30E-83	−2.83E-02	−3.19E-95	3.26E-98	−6.66E-83	−7.08E-90
	Mean	−6.88E-01	−5.86E-01	−3.60E-84	−4.03E-03	−1.06E-96	−1.08E-99	−4.51E-84	−2.79E-91
	Std.dev	3.24E-01	3.59E-01	1.69E-83	6.57E-03	5.82E-96	5.96E-99	1.65E-83	1.30E-90
	Max	−6.83E-03	−7.14E-04	−2.7E-102	−7.62E-20	−3.82E-114	−1.36E-111	−2.16E-102	−1.22E-103
F_54_	Min	0.00E + 00	0.00E + 00	1.14E + 04	0.00E + 00	1.84E + 03	7.72E + 03	3.80E + 01	1.31E + 04
	Mean	0.00E + 00	0.00E + 00	1.21E + 04	6.66E-02	3.62E + 03	8.29E + 03	6.00E + 01	1.40E + 04
	Std.dev	0.00E + 00	0.00E + 00	3.12E + 02	3.65E-01	9.81E + 02	3.76E + 02	1.22E + 01	3.15E + 02
	Max	0.00E + 00	0.00E + 00	1.27E + 04	2.00E + 00	5.60E + 03	9.06E + 03	9.80E + 01	1.48E + 04
F_55_	Min	0.00E + 00	0.00E + 00	4.53E + 05	0.00E + 00	1.02E + 06	2.00E + 05	1.26E + 02	6.09E + 05
	Mean	0.00E + 00	0.00E + 00	5.28E + 05	1.00E-01	1.27E + 06	2.33E + 05	2.50E + 02	6.62E + 05
	Std.dev	0.00E + 00	0.00E + 00	3.11E + 04	4.02E-01	9.67E + 04	1.54E + 05	7.61E + 01	2.95E + 04
	Max	0.00E + 00	0.00E + 00	5.75E + 05	2.00E + 00	1.42E + 06	2.63E + 05	4.31E + 02	7.46E + 05

		AQSCA	AQUILA	MVO	SPOTTED	SINECOS	JAYA	EQUIL	MOTH
F_56_	Min	1.20E-01	1.60E-01	2.41E-01	1.26E-01	0.00E + 00	2.58E-01	2.50E-01	2.54E-01
	Mean	2.04E-01	2.31E-01	2.56E-01	1.45E-01	0.00E + 00	2.64E-01	2.62E-01	2.65E-01
	Std.dev	3.39E-02	2.89E-02	7.43E-03	1.09E-02	0.00E + 00	4.34E-03	7.29E-03	4.96E-03
	Max	2.66E-01	2.76E-01	2.67E-01	1.65E-01	0.00E + 00	2.73E-01	2.74E-01	2.75E-01
F_57_	Min	2.55E-01	2.55E-01	3.12E-01	2.90E-01	−5.85E + 15	2.90E-01	2.78E-01	2.99E-01
	Mean	2.81E-01	2.73E-01	3.21E-01	3.00E-01	−6.04E + 14	3.05E-01	2.87E-01	3.11E-01
	Std.dev	1.61E-02	1.31E-02	5.12E-03	6.41E-03	1.16E + 15	6.23E-03	4.29E-03	5.19E-03
	Max	3.22E-01	3.12E-01	3.33E-01	3.16E-01	−3.65E + 11	3.16E-01	2.96E-01	3.22E-01
F_58_	Min	7.35E-02	9.47E-02	2.59E-01	1.27E-01	−1.48E + 18	1.97E-01	2.27E-01	2.55E-02
	Mean	1.46E-01	1.45E-01	2.75E-01	1.47E-01	−2.58E + 17	2.16E-01	2.41E-01	2.65E-02
	Std.dev	2.13E-02	2.09E-02	1.01E-02	1.09E-02	4.29E + 17	7.93E-03	6.99E-03	6.23E-03
	Max	1.87E-01	1.77E-01	3.04E-01	1.76e-01	−1.56E + 15	2.33E-01	2.56E-01	2.77E-01
F_59_	Min	0.00E + 00	0.00E + 00	3.28E + 04	0.00E + 00	6.95E + 04	1.45E + 04	6.07E + 01	4.18E + 04
	Mean	0.00E + 00	0.00E + 00	3.60E + 04	2.03E-16	8.23E + 04	1.65E + 04	9.47E + 01	4.55E + 04
	Std.dev	0.00E + 00	0.00E + 00	1.93E + 03	8.19E-16	6.98E + 03	1.09E + 03	1.57E + 01	1.67E + 03
	Max	0.00E + 00	0.00E + 00	4.02E + 04	4.40E-15	9.62E + 04	1.97E + 04	1.29E + 02	4.81E + 04
F_60_	Min	−9.99E-01	−9.99E-01	−4.70E-01	−5.50E-01	−3.96E-01	−3.85E-01	−4.52E-01	−4.66E-01
	Mean	−9.99E-01	−9.99E-01	−4.51E-01	−4.77E-01	−3.80E-01	−3.70E-01	−4.11E-01	−4.42E-01
	Std.dev	1.73E-03	1.21E-03	9.83E-04	4.39E-02	6.15E-03	6.04E-03	1.32E-02	1.24E-02
	Max	−9.89E-01	−9.94E-01	−4.35E-01	−3.64E-01	−3.73E-01	−3.61E-01	−3.86E-01	−4.25E-01
F_61_	Min	4.36E-03	4.46E-03	1.56E + 00	5.66E-01	1.61E + 00	1.67E + 00	1.66E + 00	1.12E + 00
	Mean	5.27E-03	5.40E-03	1.64E + 00	1.67E + 00	1.72E + 00	1.74E + 00	1.73E + 00	1.31E + 00
	Std.dev	1.18E-04	3.87E-04	4.33E-02	2.15E-01	4.01E-02	2.97E-02	3.90E-02	8.45E-02
	Max	1.33E-02	1.67E-02	1.72E + 00	1.82E + 00	1.81E + 00	1.80E + 00	1.80E + 00	1.54E + 00
F_62_	Min	2.21E-18	1.41E-14	5.46E + 07	2.38E-30	1.43E + 06	6.71E + 07	5.93E + 05	7.40E + 07
	Mean	1.39E-10	1.20E-09	6.19E + 07	4.36E-10	9.95E + 06	7.38E + 07	7.13E + 05	8.41E + 07
	Std.dev	8.94E-10	3.92E-09	3.96E + 06	2.39E-09	6.28E + 06	3.69E + 06	6.37E + 04	6.27E + 06
	Max	6.30E-09	1.83E-08	6.93E + 07	1.30E-08	2.50E + 07	8.46E + 07	8.46E + 05	9.99E + 07
F_63_	Min	−1.22E + 02	−1.22E + 02	1.19E + 07	1.41E + 03	2.11E + 07	4.64E + 06	5.08E + 03	1.56E + 07
	Mean	−6.54E + 01	−1.17E + 02	1.30E + 07	1.87E + 03	2.98E + 07	5.71E + 06	8.13E + 03	1.64E + 07
	Std.dev	8.38E + 01	8.76E + 00	6.13E + 05	4.09E + 02	3.44E + 06	4.37E + 05	1.85E + 03	5.77E + 05
	Max	3.73E + 02	−7.83E + 01	1.41E + 07	3.30E + 03	3.46E + 07	6.62E + 06	1.14E + 04	1.78E + 07
F_64_	Min	4.99E + 05	4.99E + 05	4.99E + 05	4.99E + 05	4.99E + 05	4.99E + 05	4.99E + 05	4.99E + 05
	Mean	4.99E + 05	4.99E + 05	4.99E + 05	4.99E + 05	4.99E + 05	4.99E + 05	4.99E + 05	4.99E + 05
	Std.dev	1.17E-08	3.93E-07	1.42E + 01	1.53E-09	8.29E + 00	1.27E + 01	5.05E + 00	9.51E + 00
	Max	4.99E + 05	4.99E + 05	4.99E + 05	4.99E + 05	4.99E + 05	4.99E + 05	4.99E + 05	4.99e + 05
F_65_	Min	8.99E-02	1.90E-01	5.02E + 11	1.13E + 05	4.90E + 12	2.79E + 10	1.89E + 05	6.36E + 11
	Mean	1.10E + 01	1.59E + 03	6.70E + 11	1.18E + 05	5.61E + 12	6.88E + 10	2.66E + 05	7.44E + 11
	Std.dev	2.45E + 01	1.16E + 03	9.81E + 10	2.72E + 04	3.22E + 11	3.16E + 10	5.68E + 04	6.99E + 10
	Max	9.96E + 01	2.65E + 03	9.43E + 11	2.62E + 05	6.22E + 12	1.59E + 11	4.52E + 05	9.07E + 11
F_66_	Min	3.06E-05	7.71E-04	6.31E + 09	9.94E-01	2.57E + 10	8.03E + 08	1.13E + 04	7.96E + 09
	Mean	2.96E-01	3.90E-01	7.39E + 09	9.98E-01	3.19E + 10	1.52E + 09	4.94E + 04	9.22E + 09
	Std.dev	3.02E-01	4.01E-01	5.48E + 08	1.31E-03	2.02E + 09	2.71E + 08	3.71E + 04	5.65E + 08
	Max	1.66E + 00	9.99E-01	8.61E + 09	9.99E-01	3.46E + 10	2.12E + 09	1.82E + 05	1.06E + 10
F_67_	Min	−3.38E + 03	−3.19E + 03	−1.20E + 03	−3.20E + 03	−4.17E + 22	−5.78E + 02	−1.16E + 03	−1.01E + 03
	Mean	−2.21E + 03	−2.41E + 03	−1.02E + 03	−2.56E + 03	−2.07E + 21	−5.09E + 02	−9.98E + 02	−9.00E + 02
	Std.dev	5.08E + 02	3.57E + 02	9.04E + 01	2.73E + 02	7.62E + 21	3.89E + 01	6.74E + 01	6.64E + 01
	Max	−1.16E + 03	−1.45E + 03	−8.48E + 02	−2.08E + 03	−1.24E + 18	−4.07E + 02	−8.76E + 02	−7.25E + 02
F_68_	Min	5.53E + 02	5.53E + 02	3.59E + 09	8.39E + 02	1.90E + 10	7.90E + 08	7.38E + 03	5.52E + 09
	Mean	5.52E + 02	5.54E + 02	4.79E + 09	9.57E + 02	2.35E + 10	1.02E + 09	2.01E + 04	6.34E + 09
	Std.dev	5.15E + 00	9.12E-01	5.49E + 08	6.74E + 01	1.67E + 09	1.57E + 08	1.13E + 04	5.02E + 08
	Max	5.84E + 02	5.57E + 02	5.56E + 09	1.14E + 03	2.63E + 10	1.28E + 09	5.29E + 04	7.71E + 09
F_69_	Min	2.45E + 02	2.49E + 02	1.51E + 09	2.49E + 02	8.51E + 09	4.75E + 08	0.1.14E + 03	2.43E + 09
	Mean	2.46E + 02	2.49E + 02	1.93E + 09	2.49E + 02	1.17E + 10	6.77E + 08	1.00E + 04	2.95E + 09
	Std.dev	5.64E-01	1.35E-01	2.00E + 08	2.49E-02	1.41E + 09	1.23E + 08	6.88E + 03	3.08E + 08
	Max	2.47E + 02	2.50E + 02	2.28E + 09	2.50E + 02	1.41E + 10	9.60E + 08	2.55E + 04	3.67E + 09

**Table 13:** Predictive results for 500D unimodal test functions.

		AQSCA		AQUILA	MVO	SPOTTED	SINECOS	JAYA		EQUIL	MOTH
F_70_	Min	4.39E-136		2.69E-39	4.74E + 03	1.49E-26	9.04E + 03	1.95E + 03		1.03E + 00	6.12E + 03
	Mean	4.91E-90		1.75E-24	5.22E + 03	7.05E-18	1.20E + 04	2.32E + 03		2.21E + 00	6.58E + 03
	Std.dev	3.47E-89		7.10E-24	2.49E + 02	1.86E-17	1.17E + 03	2.23E + 02		7.41E-01	2.44E + 02
	Max	2.45E-88		3.56E-23	5.78E + 03	6.39E-17	1.36E + 04	2.97E + 03		4.46E + 00	7.12E + 03
F_71_	Min	4.01E-03		1.31E + 00	1.42E + 07	4.95E + 02	6.94E + 07	2.52E + 06		8.85E + 02	2.12E + 07
	Mean	1.96E + 02		1.88E + 00	1.88E + 07	4.96E + 02	8.17E + 07	3.56E + 06		1.24E + 03	2.37E + 07
	Std.dev	2.15E + 02		2.06E + 02	2.36E + 06	3.02E-01	5.53E + 06	6.91E + 05		3.20E + 02	1.79E + 06
	Max	4.98E + 02		4.93E + 02	2.45E + 07	4.96E + 02	9.09E + 07	5.14E + 06		2.14E + 03	2.75E + 07
F_72_	Min	2.68E-135		1.12E-38	1.12E + 140	3.58E + 02	5.69E + 194	2.66E + 123		1.34E + 03	3.60E + 139
	Mean	4.82E-88		1.87E-21	3.99E + 171	4.32E + 31	2.91E + 199	2.44E + 165		2.55E + 22	7.40E + 175
	Std.dev	3.34E-87		7.06E-21	4.98E + 171	2.36E + 32	3.78E + 199	3.87E + 165		1.40E + 23	5.65E + 176
	Max	2.36E-86		3.64E-20	1.19E + 173	1.29E + 33	2.37E + 200	7.33E + 166		7.67E + 23	2.21E + 177
F_73_	Min	9.29E-24		9.03E-07	5.43E + 02	2.44E-09	3.39E + 02	4.71E + 02		6.94E + 01	5.17E + 02
	Mean	9.76E-01		1.79E-01	5.78E + 02	1.69E-02	5.18E + 02	5.02E + 02		8.46E + 01	5.50E + 02
	Std.dev	8.43E-01		4.06E-01	1.59E + 01	3.45E-02	1.04E + 02	1.75E + 01		7.22E + 00	1.76E + 01
	Max	2.60E + 00		1.90E + 00	6.17E + 02	9.44E-02	6.52E + 02	5.51E + 02		9.77E + 01	5.84E + 02
F_74_	Min	3.14E-132		6.56E-35	4.24E + 128	1.66E + 164	N/A	N/A		9.79E-22	N/A
	Mean	7.13E-95		9.45E-11	4.31E + 245	1.75E + 264	N/A	N/A		1.79E-10	N/A
	Std.dev	5.04E-94		4.78E-10	8.97E + 245	1.89E + 264	N/A	N/A		7.43E-10	N/A
	Max	3.56E-93		2.62E-09	1.29E + 247	5.26E + 265	N/A	N/A		3.86E-09	N/A
F_75_	Min	1.63E-132		1.57E-36	1.75E + 117	6.90E-25	N/A	N/A		1.10E-21	N/A
	Mean	4.17E-94		6.38E-12	3.09E + 231	2.43E + 20	N/A	N/A		8.58E-13	N/A
	Std.dev	2.95E-93		3.45E-11	8.77E + 231	2.68E + 20	N/A	N/A		3.99E-12	N/A
	Max	2.08E-92		1.89E-10	9.26E + 232	7.56E + 21	N/A	N/A		2.17E-11	N/A
F_76_	Min	2.32E-125		7.07E-36	1.01E + 06	2.34E-28	2.16E + 06	4.81E + 05		2.79E + 02	1.40E + 06
	Mean	1.52E-91		3.85E-25	1.12E + 06	1.45E-13	2.82E + 06	5.68E + 05		4.78E + 02	1.56E + 06
	Std.dev	1.03E-90		1.81E-24	5.90E + 04	5.34E-13	3.69E + 05	4.42E + 04		1.40E + 02	6.71E + 04
	Max	7.33E-90		9.87E-24	1.23E + 06	2.21E-12	3.35E + 06	6.55E + 05		7.38E + 02	1.70E + 06
F_77_	Min	4.99E-125		2.16E-31	4.65E + 09	1.51E-23	8.97E + 09	2.01E + 09		9.70E + 05	5.94E + 09
	Mean	9.80E-83		1.96E-18	5.27E + 09	1.41E-10	1.23E + 10	2.30E + 09		2.30E + 06	6.58E + 09
	Std.dev	6.93E-82		1.03E-17	2.79E + 08	3.75E-10	1.19E + 09	1.87E + 08		7.39E + 05	3.08E + 08
	Max	4.90E-81		5.67E-17	5.77E + 09	1.52E-09	1.38E + 10	2.84E + 09		4.03E + 06	7.35E + 09
F_78_	Min	1.69E-121		3.79E-37	7.95E + 03	4.47E-31	5.30E + 03	2.26E + 03		1.73E + 00	6.39E + 03
	Mean	2.40E-74		3.17E-16	8.57E + 03	8.96E-17	8.27E + 03	2.77E + 03		3.52E + 00	6.87E + 03
	Std.dev	1.69E-73		1.12E-15	3.46E + 02	2.85E-16	1.27E + 03	2.52E + 02		1.29E + 00	2.51E + 02
	Max	1.20E-72		5.76E-15	9.36E + 03	1.37E-15	9.90E + 03	3.25E + 03		8.96E + 00	7.52E + 03
F_79_	Min	6.58E-73		2.37E-18	1.17E + 03	5.36E-17	1.82E + 02	7.89E + 02		1.00E + 01	1.32E + 03
	Mean	1.18E-48		4.88E-12	1.22E + 03	1.14E-11	3.66E + 02	8.55E + 02		1.41E + 01	1.42E + 03
	Std.dev	8.31E-48		2.34E-11	2.96E + 01	3.94E-11	9.20E + 01	3.05E + 01		2.52E + 00	3.79E + 01
	Max	5.87E-47		1.28E-10	1.27E + 03	1.58E-10	5.86E + 02	9.15E + 02		2.22E + 01	1.48E + 03
F_80_	Min	5.15E-58		2.21E-21	8.59E + 00	2.24E + 00	9.86E + 00	7.31E + 00		2.21E + 00	8.75E + 00
	Mean	1.54E-14		4.87E-14	9.05E + 00	3.69E + 00	9.92E + 00	8.58E + 00		3.47E + 00	9.06E + 00
	Std.dev	5.03E-14		2.20E-13	1.77E-01	4.65E-01	2.84E-02	4.23E-01		7.79E-01	1.72E-01
	Max	3.07E-13		1.19E-12	9.46E + 00	4.65E + 00	9.96E + 00	9.22E + 00		5.67E + 00	9.37E + 00
F_81_	Min	0.00E + 00		3.29E-198	1.92E + 10	5.61E-97	3.00E + 11	7.84E + 08		2.37E-01	2.31E + 10
	Mean	0.00E + 00		8.94E-109	2.91E + 10	3.99E + 04	3.50E + 11	2.57E + 09		1.87E + 03	3.37E + 10
	Std.dev	0.00E + 00		4.88E-108	6.43E + 09	2.18E + 05	2.46E + 10	1.13E + 09		8.51E + 03	6.11E + 09
	Max	0.00E + 00		2.68E-107	4.23E + 10	1.19E + 06	4.11E + 11	5.22E + 09		4.68E + 04	4.37E + 10
F_82_	Min	4.46E-02		7.51E-04	1.25E + 05	2.70E + 02	4.97E + 05	1.83E + 04		4.73E + 02	1.17e + 05
	Mean	5.15E + 00		1.97E + 00	1.50e + 05	3.32E + 02	6.13E + 05	2.88E + 04		4.92E + 02	1.59E + 05
	Std.dev	5.07E + 00		2.67E + 00	1.70E + 04	2.87E + 01	5.65E + 04	5.55E + 03		9.40E + 00	1.53E + 03
	Max	1.86E + 01		1.00E + 01	1.80E + 05	4.03E + 02	6.98E + 05	4.00E + 04		5.07E + 02	1.81E + 05
F_83_	Min	−1.00E + 00		−1.00E + 00	−4.92E-04	−1.00E + 00	−5.59E-04	−1.12E-03		−2.28E-02	−5.35E-04
	Mean	−1.00E + 00		−9.99E-01	−4.54E-04	−7.76E-01	−4.81E-04	−8.47E-04		−1.38E-02	−4.84E-04
	Std.dev	0.00E + 00		2.42E-05	2.17E-05	2.92E-01	3.23E-05	9.11E-05		3.83E-03	2.05E-05
	Max	−1.00E + 00		−9.99E-01	−4.12E-04	−2.88E-03	−4.21E-04		−6.4E-04	−5.16E-03	−4.47E-04

		AQSCA	AQUILA	MVO	SPOTTED	SINECOS	JAYA	EQUIL	MOTH
F_84_	Min	−3.23E + 05	−3.21E + 06	2.23E + 12	−6.38E + 02	5.25E + 12	6.94E + 11	8.27E + 08	3.26E + 12
	Mean	−2.57E + 05	−2.56E + 05	2.60E + 12	−1.79E + 02	7.20E + 12	8.79E + 11	1.37E + 09	3.67E + 12
	Std.dev	1.68E + 04	1.32E + 04	1.66E + 11	2.50E + 02	7.12E + 11	8.16E + 10	4.47E + 08	2.08E + 11
	Max	−2.41E + 05	−2.45E + 05	2.89E + 12	2.19E + 02	8.38E + 12	1.06E + 12	2.75E + 09	4.12E + 12
F_85_	Min	1.69E-121	5.61E-01	6.76E + 08	4.95E + 02	4.86E + 09	4.76E + 07	1.21E + 03	7.60E + 08
	Mean	2.40E-73	3.57E + 02	8.71E + 08	4.96E + 02	5.59E + 09	1.22E + 08	2.88E + 03	1.06E + 09
	Std.dev	1.69E-73	1.97E + 02	1.16E + 08	4.65E-01	3.13E + 08	3.85E + 07	1.37E + 03	1.33E + 08
	Max	1.20E-72	4.94E + 02	1.09E + 09	4.97E + 02	6.11E + 09	2.10E + 08	7.71E + 03	1.35E + 09
F_86_	Min	0.00E + 00	1.14E-05	7.41E + 03	1.92E + 00	1.66E + 04	2.14E + 03	4.04E + 00	9.84E + 03
	Mean	5.77E-03	7.39E-03	8.19E + 03	1.98E + 00	2.30E + 05	2.74E + 03	6.93E + 00	1.13E + 04
	Std.dev	2.97E-02	1.47E-02	4.25E + 02	1.86E-02	2.73E + 03	2.46E + 02	1.47E + 00	5.57E + 02
	Max	2.07E-01	5.46E-02	9.15E + 03	1.99E + 00	2.65E + 04	3.29E + 03	1.21E + 01	1.24E + 04
F_87_	Min	1.58E-121	9.02E-41	8.98E + 02	1.50E-33	2.26E + 03	2.79E + 02	2.44E-01	1.04E + 03
	Mean	2.36E-74	5.69E-29	9.99E + 02	1.12E-21	2.70E + 03	3.30E + 02	4.84E-01	1.18E + 03
	Std.dev	1.67E-73	3.09E-28	5.52E + 01	3.44E-21	2.28E + 02	3.81E + 01	1.70E-01	4.85E + 01
	Max	1.18E-72	1.69E-27	1.10E + 03	1.35E-20	3.15E + 03	4.22E + 02	9.68E-01	1.25E + 03
F_88_	Min	1.96E-07	1.37E-05	5.26E + 02	6.19E + 01	1.11E + 03	1.61E + 02	2.97E + 02	5.18E + 02
	Mean	8.85E-03	7.48E-03	6.09E + 02	8.07E + 01	1.23E + 03	2.08E + 02	3.86E + 02	5.62E + 02
	Std.dev	1.69E-02	1.18E-02	3.62E + 01	1.34E + 01	5.73E + 01	2.15E + 01	3.03E + 01	2.33E + 01
	Max	8.51E-02	5.41E-02	6.76E + 02	1.26E + 02	1.33E + 03	2.64E + 02	4.41E + 02	6.09E + 02
F_89_	Min	1.69E + 03	1.88E + 03	2.06E + 04	2.31E + 03	1.59E + 04	1.02E + 04	7.18E + 03	2.34E + 04
	Mean	3.35E + 03	3.49E + 03	2.27E + 04	4.61E + 03	2.21E + 04	1.11E + 05	7.49E + 03	2.56E + 04
	Std.dev	1.05E + 03	1.01E + 03	1.04E + 03	8.43E + 02	3.64E + 03	4.45E + 02	7.70E + 01	9.16E + 02
	Max	6.38E + 03	6.13E + 03	2.47E + 04	6.09E + 03	3.00E + 04	1.21E + 04	7.59E + 03	2.73E + 04
F_90_	Min	1.44E-02	1.27E-02	7.24E + 02	3.15E + 01	6.54E + 03	9.46E + 01	3.39E + 02	6.60E + 02
	Mean	1.90E + 00	7.73E-01	1.20E + 03	4.33E + 01	1.59E + 04	1.33E + 02	4.82E + 02	9.70E + 02
	Std.dev	2.54E + 02	1.32E + 00	3.37E + 02	7.94E + 00	5.82E + 03	2.26E + 01	1.00E + 02	1.87E + 02
	Max	9.42E + 00	7.05E + 00	2.13E + 03	5.76E + 01	2.78E + 04	1.74E + 02	7.55E + 02	1.38E + 03
F_91_	Min	4.06E-03	4.01E-03	3.56E + 02	4.24E-03	4.12E + 02	3.92E + 02	1.35E + 01	4.07E + 02
	Mean	4.01E-03	4.01E-03	3.93E + 02	7.48E-03	5.08E + 02	4.09E + 02	1.83E + 01	4.33E + 02
	Std.dev	1.65E-06	2.76E-06	2.03E + 01	3.76E-03	5.33E + 01	9.00E + 00	2.78E + 00	1.35E + 01
	Max	4.02E-03	4.03E-03	4.36E + 02	1.78E-02	5.96E + 02	4.30E + 02	2.55E + 01	4.67E + 02
F_92_	Min	8.75E-01	2.10E + 01	4.91E + 03	2.09E + 02	2.00E + 04	9.60E + 02	4.60E + 02	6.23E + 03
	Mean	4.65E + 01	1.04E + 02	5.89E + 03	2.48E + 02	2.27E + 04	1.18E + 03	4.69E + 02	7.35E + 03
	Std.dev	3.65E + 01	5.77E + 01	5.28E + 02	2.18E + 01	1.25E + 03	1.68E + 02	4.17E + 00	4.98E + 02
	Max	1.39E + 02	2.11E + 02	7.03E + 03	2.83E + 02	2.51E + 04	1.70E + 03	4.75E + 02	8.42E + 03
F_93_	Min	1.08E-89	6.61E-13	3.93E + 05	1.03E-29	3.95E + 05	1.18E + 05	1.41E + 02	3.46E + 05
	Mean	1.92E-48	4.89E-06	4.48E + 05	2.47E-15	5.16E + 05	1.34E + 05	2.69E + 02	3.82E + 05
	Std.dev	6.08E-48	1.77E-05	2.99E + 04	1.06E-14	6.52E + 04	1.11E + 04	7.99E + 01	1.64E + 04
	Max	1.92E-47	8.99E-05	5.08E + 05	5.75E-14	6.38E + 05	1.57E + 05	5.58E + 02	4.20E + 05
F_94_	Min	2.15E-243	4.83E-77	1.56E + 09	8.78E-37	6.57E + 09	2.35E + 08	1.48E + 03	2.09E + 09
	Mean	3.84E-181	8.20E-39	1.86E + 09	2.39E-18	8.01E + 09	3.94E + 08	9.67E + 03	2.31E + 09
	Std.dev	0.00E + 00	4.44E-38	1.72E + 08	1.14E-17	4.84E + 08	6.55E + 07	7.45E + 03	1.43E + 08
	Max	1.79E-180	2.43E-37	2.28E + 09	6.30E-17	8.71E + 09	5.18E + 08	3.52E + 04	2.63E + 09

		AQSCA	AQUILA	MVO	SPOTTED	SINECOS	JAYA	EQUIL	MOTH
F_95_	Min	3.93E-119	2.43E-32	9.67E + 07	2.10E-26	2.21E + 08	4.69E + 07	1.76E + 04	1.42E + 08
	Mean	8.56E-95	3.83E-18	1.13E + 08	3.28E-14	2.87E + 08	5.56E + 07	4.69E + 04	1.57E + 08
	Std.dev	2.70E-94	2.07E-17	5.37E + 06	7.98E-14	3.24E + 07	4.52E + 06	1.72E + 04	7.69E + 06
	Max	8.56E-94	1.13E-16	1.22E + 08	3.88E-13	3.54E + 08	6.29E + 07	7.90E + 04	1.72E + 08
F_96_	Min	−4.99E + 00	−3.41E-03	1.45E + 08	−1.49E-02	4.28E + 08	2.79E + 07	1.05E + 01	2.26E + 08
	Mean	−4.96E + 00	−1.90E-03	1.73E + 08	−3.52E-03	9.07E + 08	3.52E + 07	3.99E + 01	2.70E + 08
	Std.dev	2.57E-02	6.44E-04	1.39E + 07	3.79E-03	1.99E + 08	4.54E + 06	2.09E + 01	1.93E + 07
	Max	−4.92E + 00	−4.04E-04	1.92E + 08	−1.93E-04	1.29E + 09	5.17E + 07	9.53E + 01	3.01E + 08
F_97_	Min	1.76E-01	5.05E-01	1.02E + 00	5.55E-01	1.63E + 00	1.09E + 00	1.05E + 00	9.57E-01
	Mean	7.59E-01	1.12E + 00	1.15E + 00	8.72E-01	2.95E + 00	1.69E + 00	1.36E + 00	1.14E + 00
	Std.dev	3.89E-01	3.41E-01	7.10E-02	1.92E-01	6.78E-01	3.08E-01	1.24E-01	1.12E-01
	Max	1.30E + 00	1.83E + 00	1.29E + 00	1.39E + 00	4.09E + 00	2.59E + 00	1.54E + 00	1.37E + 00
F_98_	Min	1.20E + 14	9.48E + 13	1.29E + 14	1.13E + 14	1.45E + 14	1.28E + 14	1.34E + 14	1.34E + 14
	Mean	1.40E + 14	1.23E + 14	1.33E + 14	1.19E + 14	1.47E + 14	1.31E + 14	1.36E + 14	1.37E + 14
	Std.dev	9.60E + 12	1.86E + 13	1.98E + 12	4.03E + 12	1.09E + 12	1.06E + 12	1.08E + 12	1.06E + 12
	Max	1.46E + 14	1.46E + 14	1.37E + 14	1.28E + 14	1.49E + 14	1.33E + 14	1.38E + 14	1.39E + 14
F_99_	Min	3.27E-135	5.60E-37	3.41E + 05	1.80E-24	1.59E + 08	6.66E + 04	2.40E + 01	5.21E + 06
	Mean	1.41E-100	7.83E-23	2.00E + 06	1.31E + 01	2.37E + 09	3.80E + 05	5.15E + 01	1.45E + 07
	Std.dev	4.36E-100	3.21E-22	1.65E + 06	2.31E + 01	1.95E + 09	2.87E + 05	2.51E + 01	6.65E + 06
	Max	1.38E-99	1.68E-21	6.43E + 06	6.38E + 01	6.81E + 09	1.26E + 06	1.57E + 02	3.78E + 07
F_100_	Min	9.18E-07	9.45E-08	7.26E + 05	1.92E + 00	1.74E + 06	2.31E + 05	2.01E + 02	1.01E + 06
	Mean	2.98E-03	6.65E-04	8.19E + 05	1.98E + 00	2.29E + 06	2.82E + 05	4.20E + 02	1.14E + 06
	Std.dev	7.10E-03	1.14E-03	4.87E + 04	1.60E-02	2.45E + 05	2.76E + 05	1.37E + 02	5.66E + 04
	Max	2.22E-02	5.23E-03	9.14E + 05	1.99E + 00	2.74E + 06	3.23E + 05	8.22E + 02	1.25E + 06

**Table 14:** Results of the Wilcoxon rank sum test results at %5 significance level for 500D multimodal benchmark problems.

	AQSCA	AQUILA	MVO	SPOTTED	SINECOS	JAYA	EQUIL	MOTH
F_1_	N/A	0.128	< 0.05	< 0.05	< 0.05	< 0.05	< 0.05	< 0.05
	1	2( +)	6( +)	3( +)	8( +)	5( +)	4( +)	7( +)
F_2_	N/A	1.00	< 0.05	< 0.05	< 0.05	< 0.05	< 0.05	< 0.05
	1	1( =)	6( +)	3( +)	8( +)	5( +)	4( +)	7( +)
F_3_	N/A	1.00	< 0.05	0.250	< 0.05	< 0.05	< 0.05	< 0.05
	1	1( =)	7( +)	3( +)	8( +)	5( +)	4( +)	6( +)
F_4_	N/A	0.711	< 0.05	< 0.05	< 0.05	< 0.05	< 0.05	< 0.05
	1	7( +)	4( +)	6( +)	8( +)	5( +)	2( +)	3( +)
F_5_	N/A	0.248	< 0.05	0.520	< 0.05	< 0.05	< 0.05	< 0.05
	2	3( +)	7( +)	1(−)	8( +)	5( +)	4( +)	6( +)
F_6_	N/A	0.148	< 0.05	< 0.05	< 0.05	< 0.05	< 0.05	< 0.05
	1	2( +)	6( +)	3( +)	8( +)	5( +)	4( +)	7( +)
F_7_	N/A	< 0.05	< 0.05	< 0.05	< 0.05	< 0.05	< 0.05	< 0.05
	1	2( +)	6( +)	3( +)	8( +)	5( +)	4( +)	7( +)
F_8_	N/A	1.00	< 0.05	< 0.05	< 0.05	< 0.05	< 0.05	< 0.05
	1	1( =)	4( +)	3( +)	7( +)	8( +)	6( +)	5( +)
F_9_	N/A	0.484	< 0.05	< 0.05	< 0.05	< 0.05	< 0.05	< 0.05
	1	2( +)	7( +)	3( +)	8( +)	5( +)	4( +)	6( +)
F_10_	N/A	< 0.05	< 0.05	< 0.05	< 0.05	< 0.05	< 0.05	< 0.05
	1	2( +)	6( +)	3( +)	8( +)	5( +)	4( +)	7( +)
F_11_	N/A	1.00	< 0.05	< 0.05	< 0.05	< 0.05	< 0.05	< 0.05
	1	1( =)	7( +)	3( +)	4( +)	8( +)	5( +)	6( +)
F_12_	N/A	< 0.05	< 0.05	< 0.05	< 0.05	< 0.05	< 0.05	< 0.05
	1	4( +)	3( +)	5( +)	8( +)	8( +)	2( +)	8( +)
F_13_	N/A	< 0.05	< 0.05	< 0.05	< 0.05	< 0.05	< 0.05	< 0.05
	1	2( +)	7( +)	4( +)	6( +)	8( +)	3( +)	5( +)
F_14_	N/A	< 0.05	< 0.05	< 0.05	< 0.05	< 0.05	< 0.05	< 0.05
	1	2( +)	5( +)	3( +)	8( +)	7( +)	4( +)	6( +)
F_15_	N/A	0.273	< 0.05	< 0.05	< 0.05	< 0.05	< 0.05	< 0.05
	1	2( +)	5( +)	3( +)	7( +)	8( +)	6( +)	4( +)
F_16_	N/A	< 0.05	< 0.05	< 0.05	< 0.05	< 0.05	< 0.05	< 0.05
	1	2( +)	6( +)	3( +)	8( +)	5( +)	4( +)	7( +)
F_17_	N/A	< 0.05	< 0.05	< 0.05	< 0.05	< 0.05	< 0.05	< 0.05
	2	1(−)	6( +)	3( +)	8( +)	5( +)	4( +)	7( +)
F_18_	N/A	< 0.05	0.09	< 0.05	0.09	0.09	0.09	0.09
	1	1( =)	4( +)	3( +)	8( +)	5( +)	6( +)	7( +)
F_19_	N/A	< 0.05	< 0.05	< 0.05	< 0.05	< 0.05	< 0.05	< 0.05
	1	2( +)	6( +)	3( +)	8( +)	4( +)	5( +)	7( +)
F_20_	N/A	< 0.05	< 0.05	< 0.05	< 0.05	< 0.05	< 0.05	< 0.05
	1	2( +)	6( +)	3( +)	8( +)	4( +)	5( +)	7( +)
F_21_	N/A	< 0.05	< 0.05	< 0.05	< 0.05	< 0.05	< 0.05	< 0.05
	1	2( +)	6( +)	3( +)	8( +)	5( +)	4( +)	7( +)
F_22_	N/A	< 0.05	< 0.05	< 0.05	< 0.05	< 0.05	< 0.05	< 0.05
	1	2( +)	5( +)	4( +)	7( +)	8( +)	3( +)	6( +)
F_23_	N/A	< 0.05	< 0.05	< 0.05	< 0.05	< 0.05	< 0.05	< 0.05
	2	1(−)	4( +)	6( +)	7( +)	8( +)	5( +)	3( +)
F_24_	N/A	< 0.05	< 0.05	< 0.05	< 0.05	< 0.05	< 0.05	< 0.05
	1	2( +)	6( +)	3( +)	8( +)	4( +)	5( +)	7( +)
F_25_	N/A	< 0.05	< 0.05	< 0.05	< 0.05	< 0.05	< 0.05	< 0.05
	1	2( +)	4( +)	6( +)	7( +)	8( +)	5( +)	3( +)
F_26_	N/A	< 0.05	< 0.05	< 0.05	< 0.05	< 0.05	< 0.05	< 0.05
	2	3( +)	6( +)	4( +)	8( +)	5( +)	1(−)	7( +)
F_27_	N/A	< 0.05	< 0.05	< 0.05	< 0.05	< 0.05	< 0.05	< 0.05
	2	1(−)	6( +)	3( +)	8( +)	5( +)	4( +)	7( +)
F_28_	N/A	< 0.05	< 0.05	< 0.05	< 0.05	< 0.05	< 0.05	< 0.05
	1	2( +)	6( +)	3( +)	8( +)	5( +)	4( +)	7( +)
F_29_	N/A	< 0.05	< 0.05	< 0.05	< 0.05	< 0.05	< 0.05	< 0.05
	1	2( +)	4( +)	3( +)	8( +)	5( +)	7( +)	6( +)
F_30_	N/A	< 0.05	< 0.05	< 0.05	< 0.05	< 0.05	< 0.05	< 0.05
	1	2( +)	7( +)	3( +)	8( +)	5( +)	4( +)	6( +)

	AQSCA	AQUILA	MVO	SPOTTED	SINECOS	JAYA	EQUIL	MOTH
F_31_	N/A	< 0.05	< 0.05	< 0.05	< 0.05	< 0.05	< 0.05	< 0.05
	1	2( +)	8( +)	3( +)	4( +)	6( +)	5( +)	7( +)
F_32_	N/A	< 0.05	< 0.05	< 0.05	< 0.05	< 0.05	< 0.05	< 0.05
	2	1(−)	4( +)	6( +)	7( +)	8( +)	5( +)	3( +)
F_33_	N/A	< 0.05	< 0.05	< 0.05	< 0.05	< 0.05	< 0.05	< 0.05
	1	3( +)	4( +)	2( +)	7( +)	8( +)	5( +)	6( +)
F_34_	N/A	< 0.05	< 0.05	< 0.05	< 0.05	< 0.05	< 0.05	< 0.05
	1	2( +)	5( +)	3( +)	7( +)	8( +)	6( +)	4( +)
F_35_	N/A	0.078	< 0.05	0.092	0.094	0.096	< 0.05	< 0.05
	3	5( +)	1(−)	6( +)	7( +)	8( +)	4( +)	2(−)
F_36_	N/A	< 0.05	< 0.05	< 0.05	< 0.05	0.137	< 0.05	< 0.05
	2	3( +)	6( +)	4( +)	8( +)	5( +)	1(−)	7( +)
F_37_	N/A	< 0.05	< 0.05	< 0.05	< 0.05	< 0.05	< 0.05	< 0.05
	1	2( +)	6( +)	3( +)	7( +)	8( +)	4( +)	5( +)
F_38_	N/A	< 0.05	< 0.05	< 0.05	< 0.05	< 0.05	< 0.05	< 0.05
	2	1(−)	8( +)	3( +)	6( +)	7( +)	4( +)	5( +)
F_39_	N/A	< 0.05	< 0.05	< 0.05	< 0.05	< 0.05	< 0.05	< 0.05
	1	2( +)	6( +)	3( +)	7( +)	8( +)	4( +)	5( +)
F_40_	N/A	0.071	< 0.05	0.582	< 0.05	< 0.05	< 0.05	< 0.05
	1	3( +)	8( +)	2( +)	5( +)	7( +)	4( +)	6( +)
F_41_	N/A	N/A	N/A	N/A	N/A	N/A	N/A	N/A
	8	8( =)	8( =)	8( =)	8( =)	8( =)	8( =)	8( =)
F_42_	N/A	N/A	N/A	N/A	N/A	N/A	N/A	N/A
	8	8( =)	8( =)	8( =)	8( =)	8( =)	8( =)	8( =)
F_43_	N/A	N/A	N/A	N/A	N/A	N/A	N/A	N/A
	8	8( =)	8( =)	8( =)	8( =)	8( =)	8( =)	8( =)
F_44_	N/A	0.500	< 0.05	< 0.05	< 0.05	< 0.05	< 0.05	< 0.05
	2	1(−)	5( +)	3( +)	8( +)	7( +)	4( +)	6( +)
F_45_	N/A	< 0.05	< 0.05	< 0.05	< 0.05	< 0.05	< 0.05	< 0.05
	1	2( +)	6( +)	3( +)	8( +)	7( +)	4( +)	5( +)
F_46_	N/A	< 0.05	< 0.05	< 0.05	< 0.05	< 0.05	< 0.05	< 0.05
	1	2( +)	4( +)	3( +)	5( +)	6( +)	7( +)	8( +)
F_47_	N/A	< 0.05	< 0.05	< 0.05	< 0.05	< 0.05	< 0.05	< 0.05
	1	2( +)	5( +)	3( +)	7( +)	8( +)	4( +)	6( +)
F_48_	N/A	< 0.05	< 0.05	< 0.05	< 0.05	< 0.05	< 0.05	< 0.05
	2	1(−)	4( +)	3( +)	8( +)	7( +)	5( +)	6( +)
F_49_	N/A	< 0.05	< 0.05	< 0.05	< 0.05	< 0.05	< 0.05	< 0.05
	1	4( +)	7( +)	2( +)	8( +)	6( +)	5( +)	3( +)
F_50_	N/A	< 0.05	< 0.05	< 0.05	< 0.05	< 0.05	< 0.05	< 0.05
	1	2( +)	6( +)	3( +)	8( +)	5( +)	4( +)	7( +)
F_51_	N/A	< 0.05	< 0.05	< 0.05	< 0.05	< 0.05	< 0.05	< 0.05
	3	1(−)	6( +)	2(−)	8( +)	5( +)	4( +)	7( +)
F_52_	N/A	0.116	< 0.05	< 0.05	< 0.05	< 0.05	< 0.05	< 0.05
	2	1(−)	6( +)	3( +)	8( +)	4( +)	5( +)	7( +)
F_53_	N/A	< 0.05	< 0.05	< 0.05	< 0.05	< 0.05	< 0.05	< 0.05
	1	2( +)	5( +)	3( +)	7( +)	8( +)	4( +)	6( +)
F_54_	N/A	1.00	< 0.05	1.00	< 0.05	< 0.05	< 0.05	< 0.05
	1	1( =)	7( +)	1( =)	5( +)	6( +)	4( +)	8( +)
F_55_	N/A	1.00	< 0.05	0.500	< 0.05	< 0.05	< 0.05	< 0.05
	1	1( =)	6( +)	3( +)	8( +)	5( +)	4( +)	7( +)
F_56_	N/A	< 0.05	0.09	0.500	< 0.05	< 0.05	< 0.05	< 0.05
	3	4( +)	5( +)	2(−)	1(−)	7( +)	6( +)	8( +)
F_57_	N/A	< 0.05	0.09	< 0.05	< 0.05	0.055	< 0.05	0.076
	3	2(−)	8( +)	5( +)	1(−)	6( +)	5( +)	7( +)
F_58_	N/A	< 0.05	0.949	< 0.05	< 0.05	0.121	0.222	0.592
	4	3(−)	8( +)	5( +)	1(−)	6( +)	7( +)	2(−)
F_59_	N/A	1.00	< 0.05	0.125	< 0.05	< 0.05	< 0.05	< 0.05
	1	1( =)	6( +)	3( +)	8( +)	5( +)	4( +)	7( +)
F_60_	N/A	< 0.05	< 0.05	< 0.05	< 0.05	< 0.05	< 0.05	< 0.05
	1	2( +)	4( +)	3( +)	7( +)	8( +)	6( +)	5( +)
F_61_	N/A	< 0.05	< 0.05	< 0.05	< 0.05	< 0.05	< 0.05	< 0.05
	1	2( +)	4( +)	5( +)	6( +)	8( +)	7( +)	3( +)
F_62_	N/A	< 0.05	< 0.05	< 0.05	< 0.05	< 0.05	< 0.05	< 0.05
	1	3( +)	6( +)	2( +)	5( +)	7( +)	4( +)	8( +)
F_63_	N/A	0.675	< 0.05	< 0.05	< 0.05	< 0.05	< 0.05	< 0.05
	2	1(−)	6( +)	3( +)	8( +)	5( +)	4( +)	7( +)
F_64_	N/A	< 0.05	< 0.05	< 0.05	< 0.05	< 0.05	< 0.05	< 0.05
	1	2( +)	4( +)	5( +)	6( +)	7( +)	8( +)	3( +)
F_65_	N/A	< 0.05	< 0.05	< 0.05	< 0.05	< 0.05	< 0.05	< 0.05
	1	2( +)	6( +)	3( +)	8( +)	5( +)	4( +)	7( +)
F_66_	N/A	0.264	< 0.05	< 0.05	< 0.05	< 0.05	< 0.05	< 0.05
	1	2( +)	6( +)	3( +)	8( +)	5( +)	4( +)	7( +)
F_67_	N/A	< 0.05	< 0.05	< 0.05	< 0.05	< 0.05	< 0.05	< 0.05
	4	3(−)	5( +)	2(−)	1(−)	8( +)	6( +)	7( +)
F_68_	N/A	< 0.05	< 0.05	0.140	< 0.05	< 0.05	< 0.05	< 0.05
	1	2( +)	6( +)	3( +)	8( +)	5( +)	4( +)	7( +)
F_69_	N/A	< 0.05	< 0.05	< 0.05	< 0.05	< 0.05	< 0.05	< 0.05
	1	2( +)	6( +)	3( +)	8( +)	5( +)	4( +)	7( +)
Aver. rank	1.68	2.30	5.68	3.47	6.97	6.28	4.65	6.05
Ranking	1	2	5	3	8	7	4	6
+ / = / −		46/11/12	65/3/1	63/4/2	62/3/4	66/3/0	64/3/2	64/3/2

**Table 15:** Results of the Wilcoxon sum rank test for 500D benchmark cases.

	AQSCA	AQUILA	MVO	SPOTTED	SINECOS	JAYA	EQUIL	MOTH
F_70_	N/A	< 0.05	< 0.05	< 0.05	< 0.05	< 0.05	< 0.05	< 0.05
	1	2( +)	6( +)	3( +)	8( +)	5( +)	4( +)	7( +)
F_71_	N/A	< 0.05	< 0.05	0.190	< 0.05	< 0.05	< 0.05	< 0.05
	2	1(−)	6( +)	3( +)	8( +)	5( +)	4( +)	7( +)
F_72_	N/A	< 0.05	< 0.05	< 0.05	< 0.05	< 0.05	< 0.05	< 0.05
	1	2( +)	6( +)	4( +)	8( +)	5( +)	3( +)	7( +)
F_73_	N/A	< 0.05	< 0.05	< 0.05	< 0.05	< 0.05	< 0.05	< 0.05
	3	2(−)	8( +)	1(−)	6( +)	5( +)	4( +)	7( +)
F_74_	N/A	< 0.05	< 0.05	0.190	< 0.05	< 0.05	< 0.05	< 0.05
	1	2( +)	4( +)	5( +)	6( +)	7( +)	8( +)	3( +)
F_75_	N/A	< 0.05	< 0.05	< 0.05	< 0.05	< 0.05	< 0.05	< 0.05
	1	3( +)	4( +)	5( +)	6( +)	7( +)	2( +)	8( +)
F_76_	N/A	< 0.05	< 0.05	< 0.05	< 0.05	< 0.05	< 0.05	< 0.05
	1	2( +)	6( +)	3( +)	8( +)	5( +)	4( +)	7( +)
F_77_	N/A	< 0.05	< 0.05	< 0.05	< 0.05	< 0.05	< 0.05	< 0.05
	1	2( +)	6( +)	3( +)	8( +)	5( +)	4( +)	7( +)
F_78_	N/A	< 0.05	< 0.05	< 0.05	< 0.05	< 0.05	< 0.05	< 0.05
	1	3( +)	8( +)	2( +)	7( +)	5( +)	4( +)	6( +)
F_79_	N/A	< 0.05	< 0.05	< 0.05	< 0.05	< 0.05	< 0.05	< 0.05
	1	2( +)	7( +)	3( +)	5( +)	6( +)	4( +)	8( +)
F_80_	N/A	0.299	< 0.05	< 0.05	< 0.05	< 0.05	< 0.05	< 0.05
	1	2( +)	6( +)	4( +)	8( +)	5( +)	3( +)	7( +)
F_81_	N/A	< 0.05	< 0.05	< 0.05	< 0.05	< 0.05	< 0.05	< 0.05
	1	2( +)	6( +)	4( +)	8( +)	5( +)	3( +)	7( +)
F_82_	N/A	< 0.05	< 0.05	< 0.05	< 0.05	< 0.05	< 0.05	< 0.05
	2	1(−)	6( +)	3( +)	8( +)	5( +)	4( +)	7( +)
F_83_	N/A	< 0.05	< 0.05	< 0.05	< 0.05	< 0.05	< 0.05	< 0.05
	1	2( +)	8( +)	3( +)	7( +)	5( +)	4( +)	6( +)
F_84_	N/A	< 0.05	< 0.05	< 0.05	< 0.05	< 0.05	< 0.05	< 0.05
	1	2( +)	6( +)	3( +)	8( +)	5( +)	4( +)	7( +)
F_85_	N/A	< 0.05	< 0.05	< 0.05	< 0.05	< 0.05	< 0.05	< 0.05
	2	1(−)	6( +)	3( +)	8( +)	5( +)	4( +)	7( +)
F_86_	N/A	< 0.05	< 0.05	< 0.05	< 0.05	< 0.05	< 0.05	< 0.05
	1	2( +)	6( +)	3( +)	8( +)	5( +)	4( +)	7( +)
F_87_	N/A	< 0.05	< 0.05	< 0.05	< 0.05	< 0.05	< 0.05	< 0.05
	1	2( +)	6( +)	3( +)	8( +)	5( +)	4( +)	7( +)
F_88_	N/A	< 0.05	< 0.05	< 0.05	< 0.05	< 0.05	< 0.05	< 0.05
	2	1(−)	7( +)	3( +)	8( +)	4( +)	5( +)	6( +)
F_89_	N/A	< 0.05	< 0.05	0.168	< 0.05	< 0.05	< 0.05	< 0.05
	1	2( +)	7( +)	3( +)	6( +)	5( +)	4( +)	8( +)
F_90_	N/A	< 0.05	< 0.05	< 0.05	< 0.05	< 0.05	< 0.05	< 0.05
	2	1(−)	7( +)	3( +)	8( +)	4( +)	5( +)	6( +)
F_91_	N/A	< 0.05	< 0.05	< 0.05	< 0.05	< 0.05	< 0.05	< 0.05
	1	2( +)	5( +)	3( +)	8( +)	6( +)	4( +)	7( +)
F_92_	N/A	< 0.05	< 0.05	< 0.05	< 0.05	< 0.05	< 0.05	< 0.05
	1	2( +)	6( +)	3( +)	8( +)	5( +)	4( +)	7( +)
F_93_	N/A	< 0.05	< 0.05	< 0.05	< 0.05	< 0.05	< 0.05	< 0.05
	1	3( +)	7( +)	2( +)	8( +)	5( +)	4( +)	6( +)
F_94_	N/A	< 0.05	< 0.05	< 0.05	< 0.05	< 0.05	< 0.05	< 0.05
	1	2( +)	6( +)	3( +)	8( +)	5( +)	4( +)	7( +)
F_95_	N/A	< 0.05	< 0.05	< 0.05	< 0.05	< 0.05	< 0.05	< 0.05
	1	2( +)	6( +)	3( +)	8( +)	5( +)	4( +)	7( +)
F_96_	N/A	< 0.05	< 0.05	< 0.05	< 0.05	< 0.05	< 0.05	< 0.05
	1	3( +)	6( +)	2( +)	8( +)	5( +)	4( +)	7( +)
F_97_	N/A	< 0.05	< 0.05	0.695	< 0.05	< 0.05	< 0.05	< 0.05
	1	3( +)	5( +)	2( +)	8( +)	7( +)	6( +)	4( +)
F_98_	N/A	< 0.05	< 0.05	< 0.05	< 0.05	< 0.05	< 0.05	< 0.05
	7	2(−)	4(−)	1(−)	8( +)	3(−)	5(−)	6(−)
F_99_	N/A	< 0.05	< 0.05	< 0.05	< 0.05	< 0.05	< 0.05	< 0.05
	1	2( +)	6( +)	3( +)	8( +)	5( +)	4( +)	7( +)
F_100_	N/A	0.695	< 0.05	< 0.05	< 0.05	< 0.05	< 0.05	< 0.05
	2	1(−)	6( +)	3( +)	8( +)	5( +)	4( +)	7( +)
Aver. rank	1.45	1.96	6.09	2.96	7.58	5.12	4.12	6.67
Ranking	1	2	6	3	8	5	4	7
+ / = / −		22/1/8	30/0/1	29/0/2	31/0/0	30/0/1	30/0/1	30/0/1

### Performance evaluation on CEC-2013 test problems

**Fig. 8:**
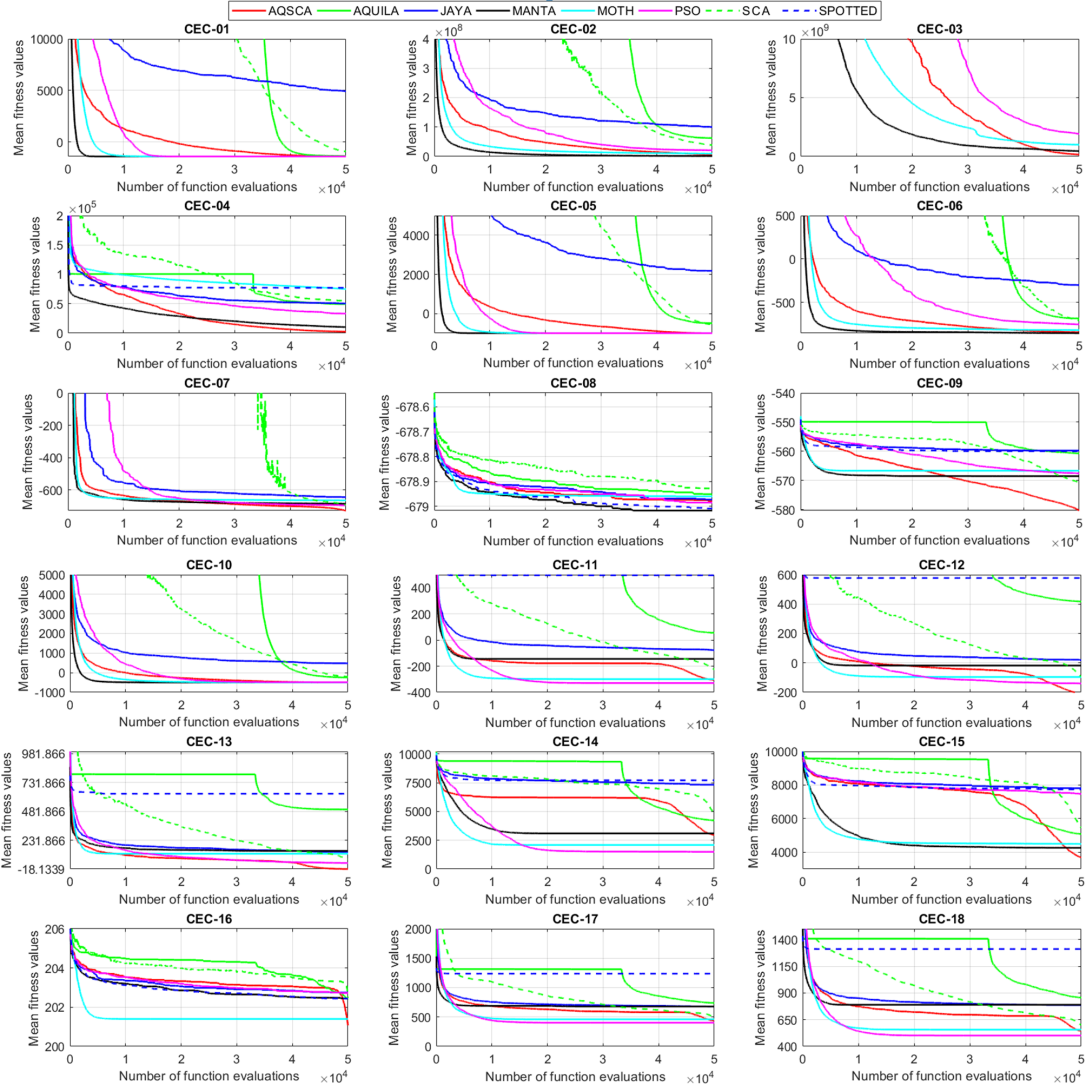
Convergence curves obtained from the compared methods from CEC–01 to CEC-18.

**Fig. 9:**
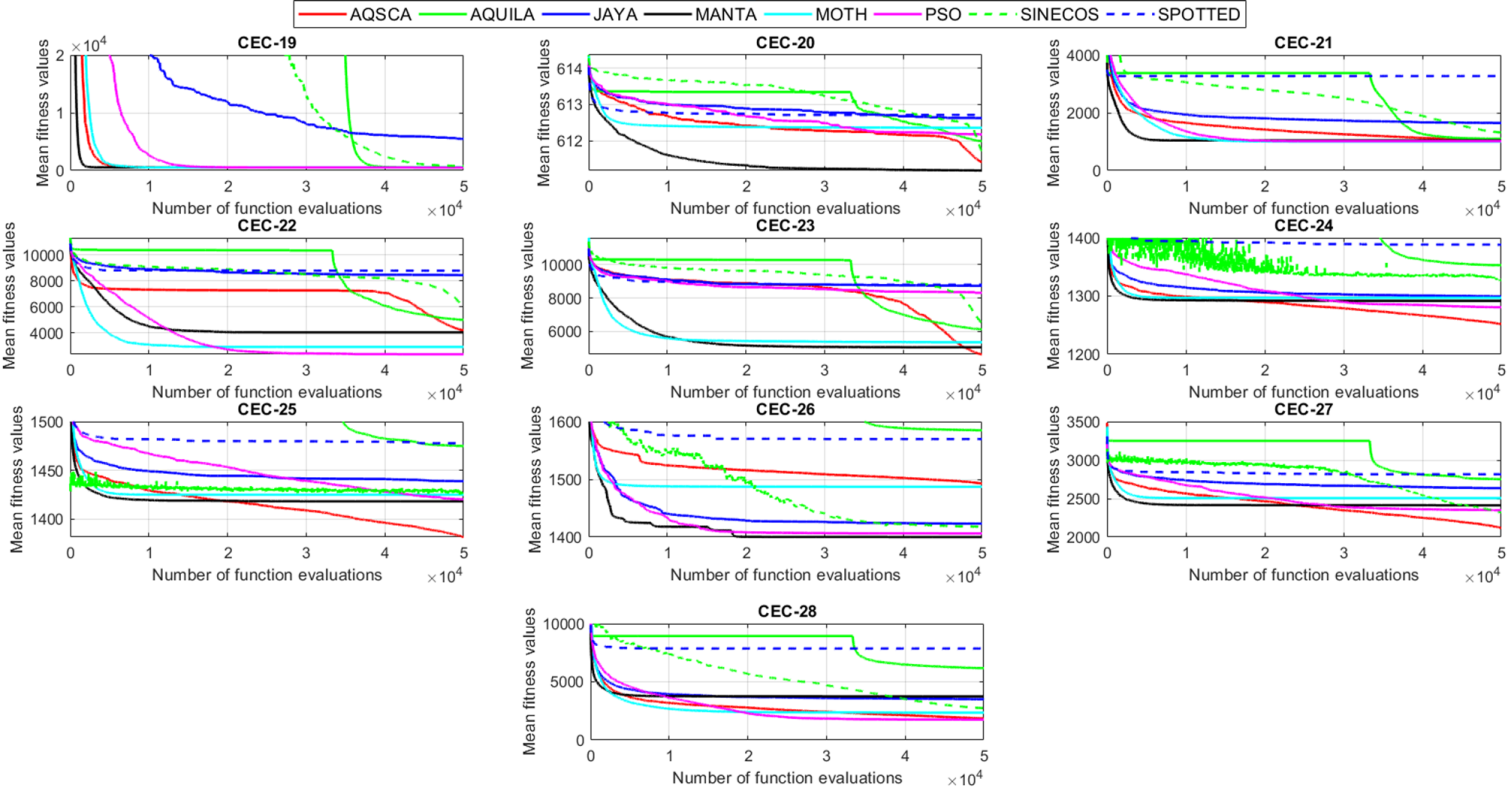
Evolution of the objective function values with the increasing number of iterations for 30D composite test problems.

This section evaluates the prediction efficiency of the proposed AQSCA algorithm on actual parameter unconstrained optimization problems that were first introduced in the IEEE Congress on Evolutionary Computation (CEC-2013)^92^. The unconstrained test problems of the CEC 2013 competitions are divided into three main categories of shift functions: unimodal, multimodal, and composition. To verify the estimation accuracy of the proposed AQSCA, a suite of 28 test problems utilized in CEC 2013 has been solved, and the respective results have been compared against the predictions found by the reputed metaheuristic algorithms of AQUILA, SPOTTED, MOTH, SINECOS, JAYA, MVO, and PSO. Definitions of the benchmark problems employed in CEC-2013 are briefly explained in Table 16. Each algorithm is run 30 times, and 50,000 function evaluations have been performed for each test problem. A performance measure based on the statistical analysis of the collected results, including the best, worst, mean, and standard deviation, is used to determine the efficiency of the competing algorithms. A total of 28 test problems consists of five benchmark problems (F_1_–F_5_), fifteen multimodal benchmark problems (F_6_–F_20_), and eight composite benchmark problems (F_20_–F_28_). It is worth mentioning that eight composite test functions from CEC 2013 are essentially integrated multimodal problems, indicating that twenty-three problems are multimodal, while the remaining five are unimodal. This means that the exploration capabilities of the compared algorithms are the primary concern to be deeply investigated when the CEC 2013 test problems are practiced. Table 17 reports the optimization results obtained for 30D unimodal problems for the compared algorithms. The balanced exploration and exploitation capabilities have been verified by the accuracy of the predictive solution obtained by the AQSCA algorithm, as it yields the best estimation results for five unimodal functions (CEC01–CEC05). Upon examining general definitions of the employed CEC problems, it becomes apparent that most test cases are rotated versions of the original standard benchmark functions, whose successful solution requires a high degree of probing ability from the algorithm. The integration of the SCA-guided search mechanism in the original AQUILA yields very accurate predictions, resulting from the distinct search characteristics of the constituent methods. This enables the exploration of various locations within the solution domain during the current iteration, thereby significantly enhancing the global exploration capability of the hybrid algorithm. This superior behavior of the proposed method can also be observed in the statistical results of the multimodal test functions (CEC06-CEC19) given in Tables 18 and 19. The apparent dominance of the proposed AQSCA is evident when considering both best and mean predictions for each benchmark problem. Composite test functions are challenging cases that involve numerous local optimum points in their solution spaces. Benchmark problems between CEC20 and CEC28 are composite test functions composed of combinations of different test functions frequently employed in literature studies for algorithm benchmarking. Despite having difficulties reaching the global best answer to some problems reported in Table 20, AQSCA proves its superiority over the comparative algorithms by successfully coping with the extreme nonlinearities of the complex composite test problems more reliably than the other algorithms in most test cases. Figures 8 and 9 illustrate the convergence curves of the algorithm in comparison to the CEC 2013 test problems, which are obtained from the average solution of thirty independent algorithm runs.

**Table 16:** Description of CEC 2013 benchmark functions.

	No	Functions	f*(x)
Unimodal Functions	1	Sphere function	−1400
	2	Rotated High Conditioned Elliptic function	−1300
	3	Rotated Bent Cigar function	−1200
	4	Rotated Discus function	−1100
	5	Different Powers function	−1000
	6	Rotated Rosenbrock function	−900
	7	Rotated Schaffers F7 function	−800
	8	Rotated Ackley function	−700
	9	Rotated Weirstrass function	−600
	10	Rotated Griewank function	−500
Basic Multimodal Functions	11	Rastrigin function	−400
	12	Rotated Rastrigin function	−300
	13	Non-Continuous rotated Rastrigin function	−200
	14	Schwefel function	−100
	15	Rotated Schwefel function	100
	16	Rotated Katsuura function	200
	17	Lunacek Bi Rastrigin function	300
	18	Rotated Lunacek Bi Rastrigin function	400
	19	Expanded Griewank plus Rosenbrock function	500
	20	Expanded Schaffer F6 function	600
Composition Functions	21	Composition function 1 (n = 5. Rotated)	700
	22	Composition function 2 (n = 3. Unrotated)	800
	23	Composition function 3 (n = 3. Rotated)	900
	24	Composition function 4 (n = 3. Rotated)	1000
	25	Composition function 5 (n = 3. Rotated)	1100
	26	Composition function 6 (n = 5. Rotated)	1200
	27	Composition function 7 (n = 5. Rotated)	1300
	28	Composition function 8 (n = 5. Rotated)	1400
		Search range : [-100.100]^D^	

**Table 17:** Comparison of the estimations performed by the competitive algorithms for 30D unimodal functions.

CEC01	AQSCA	AQUILA	SPOTTED	MOTH	SINECOS	JAYA	MVO	PSO
Min	−1.40E + 03	−1.38E + 03	3.55E + 04	−1.40E + 03	−1.28E + 03	2.33E + 03	−1.40E + 03	−1.40E + 03
Mean	−1.40E + 03	−1.35E + 03	5.13E + 04	−1.40E + 03	−9.09E + 02	4.92E + 03	−1.39E + 03	−1.40E + 03
Std. dev	6.08E-12	1.82E + 01	7.49E + 03	4.12E-10	5.06E + 02	1.46E + 03	1.80E-02	1.01E-05
Max	−1.40E + 03	−1.29E + 03	6.38E + 04	−1.40E + 03	8.53E + 02	8.55E + 03	−1.39E + 03	−1.40E + 03
CEC02	**AQSCA**	**AQUILA**	**SPOTTED**	**MOTH**	**SINECOS**	**JAYA**	**MVO**	**PSO**
Min	2.65E + 05	2.03E + 07	3.24E + 08	2.54E + 06	2.05E + 07	3.55E + 07	1.52E + 06	3.43E + 06
Mean	1.36E + 06	6.26E + 07	8.97E + 08	9.38E + 06	3.87E + 07	1.00E + 08	6.30E + 06	2.09E + 07
Std. dev	5.97E + 05	2.24E + 07	3.35E + 08	7.62E + 06	1.37E + 07	3.50E + 07	2.57E + 06	1.30E + 07
Max	2.53E + 06	1.13E + 08	1.97E + 09	3.08E + 07	7.64E + 07	1.97E + 08	1.29E + 07	5.05E + 07
CEC03	**AQSCA**	**AQUILA**	**SPOTTED**	**MOTH**	**SINECOS**	**JAYA**	**MVO**	**PSO**
Min	2.54E + 07	1.09E + 10	1.16E + 12	2.65E + 07	5.90E + 09	1.09E + 10	4.10E + 06	1.38E + 08
Mean	4.60E + 08	1.33E + 11	3.22E + 18	1.00E + 09	1.20E + 10	2.76E + 10	1.66E + 08	1.91E + 09
Std. dev	6.21E + 08	5.35E + 11	1.72E + 19	1.10E + 09	3.49E + 09	8.75E + 09	2.09E + 08	2.54E + 09
Max	2.82E + 09	2.96E + 12	9.46E + 19	4.53E + 09	1.94E + 10	5.36E + 10	9.09E + 08	1.02E + 10
CEC04	**AQSCA**	**AQUILA**	**SPOTTED**	**MOTH**	**SINECOS**	**JAYA**	**MVO**	**PSO**
Min	3.50E + 03	3.77E + 04	6.07E + 04	4.08E + 04	3.44E + 04	3.67E + 04	−7.25E + 02	1.77E + 04
Mean	1.03E + 04	4.95E + 04	7.70E + 04	7.50E + 04	5.51E + 04	5.04E + 04	2.60E + 03	3.33E + 04
Std. dev	4.54E + 03	4.14E + 03	1.14E + 04	1.63E + 04	8.58E + 03	7.77E + 03	1.88E + 03	8.03E + 03
Max	1.96E + 04	5.81E + 04	9.81E + 04	1.06E + 05	7.49E + 04	6.84E + 04	8.04E + 03	4.81E + 04
CEC05	**AQSCA**	**AQUILA**	**SPOTTED**	**MOTH**	**SINECOS**	**JAYA**	**MVO**	**PSO**
Min	−1.00E + 03	−8.00E + 02	1.14E + 04	−1.00E + 03	−7.87E + 02	8.72E + 02	−9.99E + 02	−1.00E + 03
Mean	−1.00E + 03	−4.90E + 02	6.70E + 04	−1.00E + 03	−5.51E + 02	2.18E + 03	−9.99E + 02	−1.00E + 03
Std. dev	1.58E-12	2.06E + 02	3.06E + 04	2.16E-08	2.72E + 02	8.25E + 02	1.45E-02	4.94E-05
Max	−1.00E + 03	1.40E + 02	1.44E + 05	−1.00E + 03	6.76E + 02	4.31E + 03	−9.99E + 02	−1.00E + 03

**Table 18:** Multimodal test functions employed in CEC competitions from CEC06 – CEC12.

CEC06	AQSCA	AQUILA	SPOTTED	MOTH	SINECOS	JAYA	MVO	PSO
Min	−8.84E + 02	−7.86E + 02	3.01E + 03	−8.93E + 02	−7.88E + 02	−4.76E + 02	−8.92E + 02	−8.35E + 02
Mean	−8.55E + 02	−6.85E + 02	8.46E + 03	−8.20E + 02	−7.07E + 02	−3.00E + 02	−8.44E + 02	−7.54E + 02
Std. dev	2.78E + 01	8.04E + 01	2.66E + 03	6.39E + 01	6.60E + 01	8.69E + 01	2.97E + 01	5.67E + 01
Max	−8.18E + 02	−3.97E + 02	1.46E + 04	−5.61E + 02	−4.58E + 02	−1.15E + 02	−7.99E + 02	−6.54E + 02
CEC07	**AQSCA**	**AQUILA**	**SPOTTED**	**MOTH**	**SINECOS**	**JAYA**	**MVO**	**PSO**
Min	−7.20E + 02	−6.09E + 02	−5.20E + 02	−7.34E + 02	−7.33E + 02	−6.87E + 02	−7.78E + 02	−7.43E + 02
Mean	−6.85E + 02	2.11E + 03	6.75E + 05	−6.64E + 02	−6.94E + 02	−6.44E + 02	−7.27E + 02	−6.95E + 02
Std. dev	2.15E + 01	3.41E + 03	1.79E + 06	2.96E + 01	1.48E + 01	2.49E + 01	2.78E + 01	2.56E + 01
Max	−6.45E + 02	1.34E + 04	6.98E + 06	−5.98E + 02	−6.56E + 02	−5.96E + 02	−6.77E + 02	−6.21E + 02
CEC08	**AQSCA**	**AQUILA**	**SPOTTED**	**MOTH**	**SINECOS**	**JAYA**	**MVO**	**PSO**
Min	−6.79E + 02	−6.79E + 02	−6.79E + 02	−6.79E + 02	−6.79E + 02	−6.79E + 02	−6.79E + 02	−6.79E + 02
Mean	−6.79E + 02	−6.78E + 02	−6.79E + 02	−6.78E + 02	−6.78E + 02	−6.78E + 02	−6.78E + 02	−6.78E + 02
Std. dev	5.55E-02	4.47E-02	6.67E-02	1.16E-01	5.58E-02	5.53E-02	5.18E-02	5.84E-02
Max	−6.78E + 02	−6.78E + 02	−6.78E + 02	−6.78E + 02	−6.78E + 02	−6.78E + 02	−6.78E + 02	−6.78E + 02
CEC09	**AQSCA**	**AQUILA**	**SPOTTED**	**MOTH**	**SINECOS**	**JAYA**	**MVO**	**PSO**
Min	−5.76E + 02	−5.69E + 02	−5.64E + 02	−5.79E + 02	−5.75E + 02	−5.63E + 02	−5.86E + 02	−5.86E + 02
Mean	−5.68E + 02	−5.60E + 02	−5.59E + 02	−5.66E + 02	−5.70E + 02	−5.59E + 02	−5.80E + 02	−5.67E + 02
Std. dev	3.60E + 00	3.82E + 00	2.20E + 00	4.20E + 00	2.12E + 00	1.53E + 00	3.36E + 00	4.49E + 00
Max	−5.62E + 02	−5.54E + 02	−5.55E + 02	−5.59E + 02	−5.67E + 02	−5.57E + 02	−5.72E + 02	−5.58E + 02
CEC10	**AQSCA**	**AQUILA**	**SPOTTED**	**MOTH**	**SINECOS**	**JAYA**	**MVO**	**PSO**
Min	−4.99E + 02	−4.09E + 02	5.40E + 03	−4.98E + 02	−4.63E + 02	8.66E + 01	−4.98E + 02	−4.98E + 02
Mean	−4.99E + 02	−2.42E + 02	8.60E + 03	−4.98E + 02	−2.03E + 02	4.78E + 02	−4.98E + 02	−4.86E + 02
Std. dev	2.27E-04	1.07E + 02	1.68E + 03	2.45E-01	1.65E + 02	2.05E + 02	1.14E-01	1.86E + 01
Max	−4.99E + 02	2.30E + 01	1.18E + 04	−4.97E + 02	2.71E + 02	1.01E + 03	−4.98E + 02	−4.15E + 02
CEC11	**AQSCA**	**AQUILA**	**SPOTTED**	**MOTH**	**SINECOS**	**JAYA**	**MVO**	**PSO**
Min	−2.82E + 02	−1.20E + 02	2.03E + 02	−3.47E + 02	−2.71E + 02	−1.35E + 02	−3.36E + 02	−3.65E + 02
Mean	−1.44E + 02	5.54E + 01	4.95E + 02	−2.99E + 02	−2.23E + 02	−7.72E + 01	−3.04E + 02	−3.29E + 02
Std. dev	9.24E + 01	8.84E + 01	1.26E + 02	2.87E + 01	3.07E + 01	3.63E + 01	2.46E + 01	1.61E + 01
Max	8.45E + 01	2.17E + 02	7.77E + 02	−2.48E + 02	−1.53E + 02	5.81E + 00	−2.52E + 02	−2.87E + 02
CEC12	**AQSCA**	**AQUILA**	**SPOTTED**	**MOTH**	**SINECOS**	**JAYA**	**MVO**	**PSO**
Min	−1.58E + 02	1.52E + 02	4.04E + 02	−1.76E + 02	−1.31E + 02	−2.43E + 01	−2.59E + 02	−2.39E + 02
Mean	−1.77E + 01	4.18E + 02	5.77E + 02	−9.57E + 01	−9.04E + 01	1.87E + 01	−2.05E + 02	−1.39E + 02
Std. dev	8.00E + 01	1.01E + 02	1.17E + 02	6.30E + 01	3.57E + 01	2.12E + 01	2.98E + 01	5.70E + 01
Max	−1.38E + 02	5.79E + 02	8.21E + 02	1.00E + 02	1.39E + 01	6.80E + 01	−1.47E + 02	−1.28E + 01

**Table 19:** Multimodal test function between CEC13 and CEC20.

CEC13	AQSCA	AQUILA	SPOTTED	MOTH	SINECOS	JAYA	MVO	PSO
Min	1.73E + 01	2.28E + 02	3.63E + 02	−2.01E + 01	1.37E + 01	4.21E + 01	−8.27E + 01	−7.03E + 01
Mean	1.38E + 02	4.97E + 02	6.33E + 02	1.14E + 02	6.81E + 01	1.28E + 02	−1.81E + 01	3.35E + 01
Std. dev	6.97E + 01	1.28E + 02	1.33E + 02	6.08E + 01	3.35E + 01	3.05E + 01	4.20E + 01	4.28E + 01
Max	3.10E + 02	7.87E + 02	8.80E + 02	2.32E + 02	1.34E + 02	2.04E + 02	8.53E + 01	1.30E + 02
CEC14	**AQSCA**	**AQUILA**	**SPOTTED**	**MOTH**	**SINECOS**	**JAYA**	**MVO**	**PSO**
Min	1.81E + 03	2.99E + 03	6.49E + 03	1.04E + 03	3.87E + 03	5.29E + 03	1.38E + 03	6.59E + 02
Mean	3.09E + 03	4.23E + 03	7.71E + 03	2.08E + 03	4.90E + 03	7.33E + 03	2.96E + 03	1.48E + 03
Std. dev	6.53E + 02	7.14E + 02	6.26E + 02	6.35E + 02	4.81E + 02	4.61E + 02	5.88E + 02	4.81E + 02
Max	4.34E + 03	5.26E + 03	8.65E + 02	3.73E + 03	5.78E + 03	7.80E + 03	4.11E + 03	2.52E + 03
CEC15	**AQSCA**	**AQUILA**	**SPOTTED**	**MOTH**	**SINECOS**	**JAYA**	**MVO**	**PSO**
Min	2.85E + 03	3.77E + 03	6.52E + 03	2.82E + 03	4.68E + 03	6.55E + 03	2.62E + 03	6.23E + 03
Mean	4.26E + 03	5.08E + 03	7.74E + 03	4.49E + 03	5.48E + 03	7.80E + 03	3.69E + 03	7.46E + 03
Std. dev	6.51E + 02	6.18E + 02	6.21E + 02	7.33E + 02	4.32E + 02	3.34E + 02	6.56E + 02	4.07E + 02
Max	5.39E + 03	6.38E + 03	9.15E + 03	5.93E + 03	6.48E + 03	8.15E + 03	5.29E + 03	8.18E + 03
CEC16	**AQSCA**	**AQUILA**	**SPOTTED**	**MOTH**	**SINECOS**	**JAYA**	**MVO**	**PSO**
Min	2.01E + 02	2.01E + 02	2.01E + 02	2.01E + 02	2.01E + 02	2.01E + 02	2.00E + 02	2.01E + 02
Mean	2.02E + 02	2.02E + 02	2.02E + 02	2.01E + 02	2.02E + 02	2.02E + 02	2.01E + 02	2.02E + 02
Std. dev	4.49E-01	5.90E-01	3.96E-01	5.60E-01	3.91E-01	3.91E-01	4.44E-01	3.40E-01
Max	2.03E + 04	2.03E + 02	2.02E + 02	2.02E + 02	2.03E + 02	2.03E + 02	2.02E + 92	2.03E + 02
CEC17	**AQSCA**	**AQUILA**	**SPOTTED**	**MOTH**	**SINECOS**	**JAYA**	**MVO**	**PSO**
Min	4.22E + 02	6.32E + 02	9.45E + 02	4.17E + 02	4.48E + 02	6.08E + 02	3.94E + 02	3.70E + 02
Mean	6.79E + 02	7.38E + 02	1.23E + 03	4.61E + 02	5.05E + 02	6.77E + 02	4.40E + 02	4.02E + 02
Std. dev	1.15E + 02	5.59E + 01	1.13E + 02	3.08E + 01	2.95E + 01	4.03E + 01	2.32E + 01	1.88E + 01
Max	9.39E + 02	8.56E + 02	1.43E + 03	5.41E + 02	5.89E + 02	7.97E + 02	4.79E + 02	4.48E + 02
CEC18	**AQSCA**	**AQUILA**	**SPOTTED**	**MOTH**	**SINECOS**	**JAYA**	**MVO**	**PSO**
Min	6.24E + 02	7.31E + 02	1.06E + 03	4.75E + 02	5.71E + 02	7.04E + 02	4.79E + 02	4.56E + 02
Mean	7.89E + 02	8.56E + 02	1.31E + 03	5.56E + 02	6.02e + 02	7.85E + 02	5.46E + 02	5.01E + 02
Std. dev	1.06E + 02	7.91E + 01	1.15E + 02	4.50E + 01	2.03E + 01	3.55E + 01	3.32e + 01	2.28E + 01
Max	1.10E + 03	9.91E + 02	1.55E + 03	6.51E + 02	6.53E + 02	8.71E + 02	6.03E + 02	5.57E + 02
CEC19	**AQSCA**	**AQUILA**	**SPOTTED**	**MOTH**	**SINECOS**	**JAYA**	**MVO**	**PSO**
Min	5.05E + 02	5.29E + 02	1.16E + 05	5.08E + 02	5.29E + 02	1.53E + 03	5.05E + 02	5.05E + 02
Mean	5.15E + 02	5.51E + 02	4.13E + 05	5.34E + 02	7.93E + 02	5.47E + 03	5.09E + 02	5.09E + 02
Std. dev	1.00E + 01	1.12E + 01	2.33E + 05	5.73E + 01	6.30E + 02	3.24E + 03	1.90E + 00	3.50E + 00
Max	5.54E + 02	5.79E + 02	1.00E + 06	8.16E + 02	3.86E + 03	1.31E + 04	5.13E + 02	5.20E + 02
CEC20	**AQSCA**	**AQUILA**	**SPOTTED**	**MOTH**	**SINECOS**	**JAYA**	**MVO**	**PSO**
Min	6.09E + 02	6.11E + 02	6.11E + 02	6.10E + 02	6.10E + 02	6.12E + 02	6.09E + 02	6.11E + 02
Mean	6.11E + 02	6.11E + 02	6.12E + 02	6.12E + 02	6.11E + 02	6.12E + 02	6.11E + 02	6.12E + 02
Std. dev	5.70E-01	3.02E-01	3.40E-01	6.18E-01	4.50E-01	2.27E-01	7.60E-01	3.76E-01
Max	6.11E + 02	6.12E + 02	6.13E + 02	6.13E + 02	6.12E + 02	6.13E + 02	6.12E + 02	6.13E + 02

**Table 20:** Composite test functions for performance benchmarking of the compared algorithms.

CEC21	AQSCA	AQUILA	SPOTTED	MOTH	SINECOS	JAYA	MVO	PSO
Min	8.00E + 02	1.06E + 03	3.05E + 03	9.00E + 02	1.11E + 03	1.28E + 03	8.08E + 02	9.00E + 02
Mean	1.04E + 03	1.09E + 03	3.26E + 03	1.00E + 03	1.32E + 03	1.64E + 03	1.01E + 03	1.01E + 03
Std. dev	6.74E + 01	1.28E + 01	7.52E + 01	5.87E + 01	1.95E + 02	3.53E + 02	7.97E + 01	6.47E + 01
Max	1.10E + 03	1.11E + 03	3.40E + 03	1.10E + 03	1.79E + 03	2.47E + 03	1.10E + 03	1.10E + 03
CEC22	**AQSCA**	**AQUILA**	**SPOTTED**	**MOTH**	**SINECOS**	**JAYA**	**MVO**	**PSO**
Min	2.72E + 03	3.79E + 03	8.05E + 03	2.14E + 03	4.96E + 03	8.01E + 03	2.43E + 03	1.72E + 03
Mean	4.05E + 03	5.01E + 03	8.78E + 03	2.93E + 03	5.97E + 03	8.44E + 03	4.21E + 03	2.37E + 03
Std. dev	5.73E + 02	7.61E + 02	4.70E + 02	4.20E + 02	5.22e + 02	2.63E + 02	7.77E + 02	3.97E + 02
Max	5.44E + 03	6.34E + 03	9.61E + 03	3.58E + 03	7.15E + 03	9.08E + 03	5.88E + 03	3.48E + 03
CEC23	**AQSCA**	**AQUILA**	**SPOTTED**	**MOTH**	**SINECOS**	**JAYA**	**MVO**	**PSO**
Min	3.81E + 03	4.24E + 03	7.82E + 03	4.02E + 03	5.22E + 03	7.96E + 03	3.35E + 03	5.13E + 03
Mean	5.05E + 03	6.13E + 03	8.80E + 03	5.36E + 03	6.54E + 03	8.73E + 03	4.64E + 03	8.31E + 03
Std. dev	6.43E + 02	8.03E + 02	4.92E + 02	6.70E + 02	5.00E + 02	2.90E + 02	7.19E + 02	6.79E + 02
Max	6.15E + 03	7.50E + 03	9.56E + 03	6.46E + 03	7.34E + 03	9.18E + 03	5.66E + 03	9.02E + 03
CEC24	**AQSCA**	**AQUILA**	**SPOTTED**	**MOTH**	**SINECOS**	**JAYA**	**MVO**	**PSO**
Min	1.26E + 03	1.30E + 03	1.32E + 03	1.26E + 03	1.31E + 03	1.28E + 03	1.22E + 03	1.25E + 03
Mean	1.29E + 03	1.35E + 03	1.38E + 03	1.29E + 03	1.32E + 03	1.29E + 03	1.25E + 03	1.28E + 03
Std. dev	1.24E + 01	5.25E + 01	6.84E + 01	1.42E + 01	6.91E + 00	8.25E + 00	1.41E + 01	1.38E + 01
Max	1.31E + 03	1.60E + 03	1.69E + 03	1.31E + 03	1.34E + 03	1.32e + 03	1.27E + 03	1.30E + 03
CEC25	**AQSCA**	**AQUILA**	**SPOTTED**	**MOTH**	**SINECOS**	**JAYA**	**MVO**	**PSO**
Min	1.39E + 03	1.42E + 03	1.45E + 03	1.39E + 03	1.40E + 03	1.42E + 03	1.36E + 03	1.40E + 03
Mean	1.41E + 03	1.47E + 03	1.47E + 03	1.42E + 03	1.42E + 03	1.43E + 03	1.38E + 03	1.42E + 03
Std. dev	1.38E + 01	2.77E + 01	1.98E + 01	1.28E + 01	7.06E + 00	6.04E + 00	1.04E + 01	1.02E + 01
Max	1.44E + 03	1.55E + 03	1.51E + 03	1.45E + 03	1.43E + 03	1.45E + 03	1.40E + 03	1.44E + 03
CEC26	**AQSCA**	**AQUILA**	**SPOTTED**	**MOTH**	**SINECOS**	**JAYA**	**MVO**	**PSO**
Min	1.40E + 03	1.40E + 03	1.43E + 03	1.40E + 03	1.40E + 03	1.40E + 03	1.40E + 03	1.40E + 03
Mean	1.40E + 03	1.58E + 03	1.56E + 03	1.48E + 03	1.41E + 03	1.42E + 03	1.49E + 03	1.40E + 03
Std. dev	2.36E-02	6.21E + 01	7.04E + 01	9.46E + 01	5.05E + 01	4.62E + 01	7.19E + 01	3.18E + 01
Max	1.40E + 02	1.62E + 03	1.64E + 03	1.60E + 03	1.57E + 03	1.59E + 03	1.56E + 03	1.57E + 03
CEC27	**AQSCA**	**AQUILA**	**SPOTTED**	**MOTH**	**SINECOS**	**JAYA**	**MVO**	**PSO**
Min	2.19E + 03	2.55E + 03	2.60E + 03	2.37E + 03	2.13E + 03	2.44E + 03	1.99E + 03	2.08E + 03
Mean	2.41E + 03	2.75E + 03	2.81E + 03	2.50E + 03	2.31E + 03	2.63E + 03	2.11E + 03	2.34E + 03
Std. dev	1.20E + 02	1.03E + 02	8.96E + 01	6.82E + 01	1.33E + 02	8.95E + 01	7.43E + 01	9.47E + 01
Max	2.66E + 03	2.92E + 03	3.04E + 03	2.62E + 03	2.75E + 03	2.74E + 03	2.26E + 03	2.54E + 03
CEC28	**AQSCA**	**AQUILA**	**SPOTTED**	**MOTH**	**SINECOS**	**JAYA**	**MVO**	**PSO**
Min	1.50E + 03	5.12E + 03	6.39E + 03	1.70E + 03	2.02E + 03	3.13E + 03	1.50E + 03	1.50E + 03
Mean	3.74E + 03	6.18E + 03	7.86E + 03	2.32E + 03	2.74E + 03	3.48E + 03	1.85E + 03	1.75E + 03
Std. dev	1.22E + 03	5.72E + 02	8.75E + 02	1.12E + 03	5.59E + 02	1.74E + 02	3.90E + 02	2.61E + 02
Max	5.76E + 03	7.37E + 03	1.01E + 04	5.42E + 03	4.10E + 03	3.91E + 03	2.89E + 03	3.09E + 03

### Comparative evaluation of the algorithms based on the CEC 2022 test problems

**Fig. 10:**
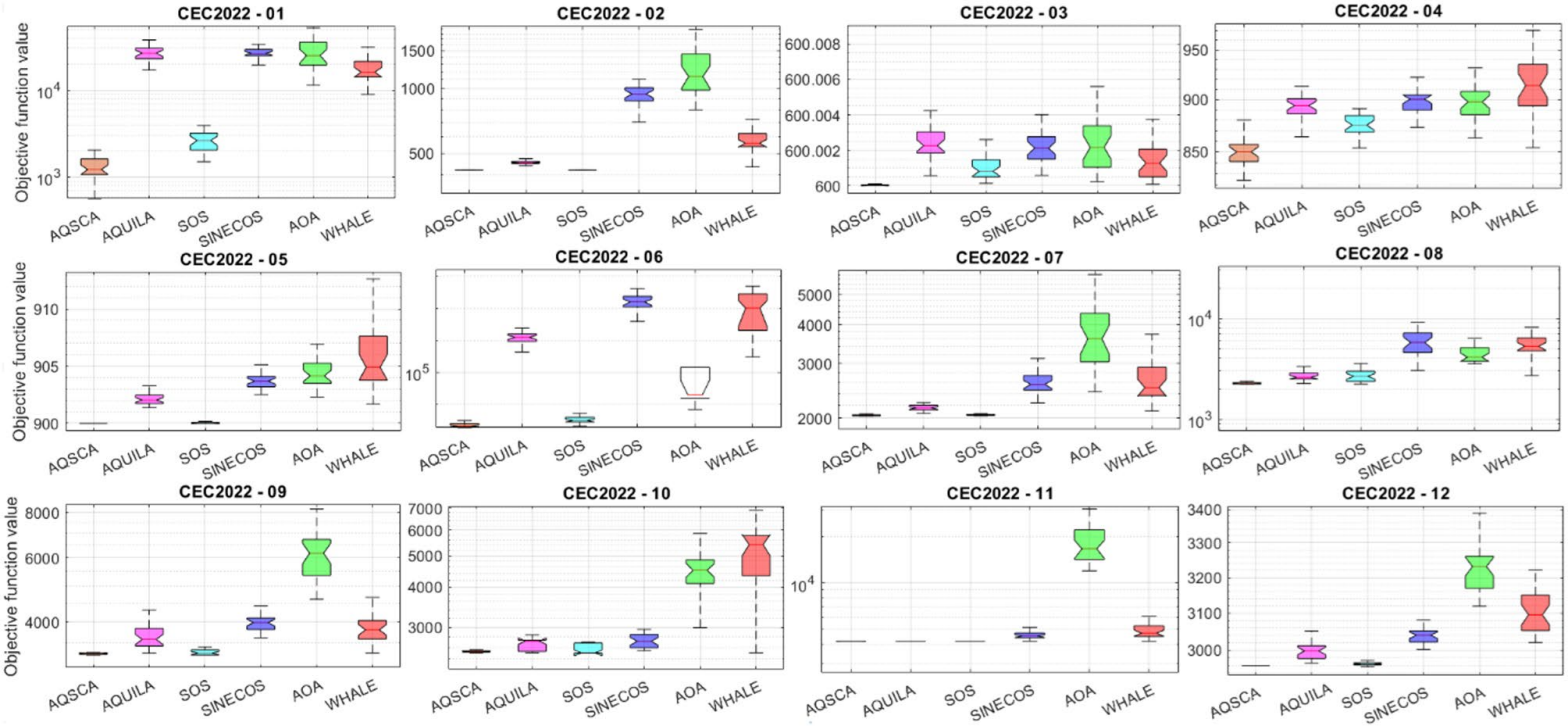
Statistical results for 20D CEC 2022 test problems.

This section compares the optimization performance of the proposed AQSCA algorithm by employing twelve 20D CEC2022 benchmark functions. It assesses its search efficiency through a comprehensive comparison with the estimations performed by the standard AQUILA algorithm, Symbiotic Organisms Search (SOS)^93^, SCA, Arithmetic Optimization Algorithm (AOA)^94^, and Whale Optimization Algorithm (WHALE)^95^. Figure 10 displays box-plot results comparing the statistical performance of the proposed hybrid AQSCA with that of other mentioned algorithms for the CEC 2022 test problems. The comparative evaluations are based on 30 independent runs, each with 200,000 function evaluations. The y-axis represents the objective function value on a logarithmic scale, where lower values indicate better performance. The CEC 2022 benchmark problems test the compared algorithms in various aspects. Unimodal benchmark functions assess exploitation ability, while multimodal functions evaluate the diversification to escape local optimum solutions. Hybrid and composition functions are used as effective test beds for determining the balance between exploration and exploitation capabilities in complex, shifted, rotated, and composite landscapes. Table 21 describes these twelve CEC 2022 test problems, along with the defined search bounds, problem dimensionalities, and known global optimum values. In most test problems, AQSCA consistently demonstrates superior predictive performance, with the lowest median values and the tightest spreads, which indicates better convergence and solution robustness. This behavior can be attributed to its hybrid structure, which integrates the intrinsic bio-hunting strategies of AQUILA with a sine-cosine-based solution update mechanism, enabling the enhancement of local refinement and the adaptive balancing of the exploration-exploitation trade-off. The first two benchmark functions are unimodal, testing the comparative algorithms’ ability to converge to an optimal solution quickly. AQSCA achieves the lowest median with the minimum spread for these 20D unimodal benchmarks thanks to the elite SCA components that refined AQUILA’s positions, reducing variance and improving exploitation. The third and fourth benchmark functions in Table 21 are multimodal test problems, practical for testing global exploration. The Shifted and Rotated Expanded Schaffer’s F6 function has many local optima and flat regions, which necessitate testing exploration to avoid traps in the search space. AQSCA finds the exact global optimum point in each independent run and confirms its superiority over other algorithms. An intelligent combination of Levy and trigonometric mechanisms, implemented within the AQSCA structure, enhances exploration, resulting in superior global search. The shifted and rotated non-continuous Rastrigin function exhibits a parabolic shape with cosine as bed ripples, creating numerous local minimum points, along with non-continuity characteristics that add irregularity, thereby testing robust exploration. AQSCA achieves the lowest median values with low variance. The discontinuities in the test function challenge continuous update algorithms, including WHALE and SOS. AQUILA’s randomized flight mechanism handles the irregular regions well, and SCA’s oscillations effectively navigate the multimodal search space. However, they become inferior to the AQSCA in terms of prediction performance, which utilizes sine-based oscillations to smooth AQUILA’s unpredictable jumps, thereby enabling it to handle discontinuities and multimodal traps. The Shifted and Rotated Multimodal Levy Function exhibits flat outer regions, which test the balance between escaping from flat areas and exploiting ridges. AQSCA achieves the lowest median, closely approaching the known global optimum value of 900, with minimal outlier predictions. SOS is the second-best optimizer, with a lower spread compared to those obtained for AQUILA, SCA, and AOA. WHALE lacks accurate predictions for this test function, with relatively higher mean values and a wider spread. Hybrid test function – C_6_ combines three test functions, generating an irregular fitness landscape to evaluate and an adaptive search mechanism of the AQSCA algorithm. AQSCA shows the lowest median by outperforming other optimizers. SOS is the second-best optimizer with a narrow box. Hybrid test functions penalize the rigid search scheme of AOA, which is composed of fundamental arithmetic operations or fixed search behaviors of WHALE. An intelligent switch between AQUILA’s global search and SCA’s local search mechanism adds flexibility to AQSCA, allowing it to balance the exploration and exploitation phases, whose effective integration is beneficial for accurately solving hybrid test functions. For Hybrid Function 2 – C_7_, AQSCA and SOS exhibit similar prediction performances; however, AQSCA has lower mean values compared to SOS. AOA has the highest spread and mean values compared to others and is significantly surpassed by the remaining algorithms. AQSCA achieves the lowest estimation values for Hybrid Function 3 – C_8_, followed by the standard AQUILA algorithm in second place in terms of mean results. The SCA algorithm is the worst predictor for this case. Composite test functions serve as effective test beds for evaluating the algorithm’s performance in terms of balancing exploration and exploitation. The algorithm with this capability can make a smooth transition between these complementary phases, enabling the achievement of accurate predictions by eliminating trapped local regions. The effectiveness of the composite test functions lies in their ability to reflect the true strengths of the compared optimizers by simulating complex heterogeneous search landscapes. Test functions C_9_ to C_12_ comprise benchmark problems relevant to the comparison of the optimization methods. AQSCA demonstrates its robust and accurate search ability for these cases by producing nearly identical optimal results for each algorithm run, which are significantly better than those obtained by the remaining methods in terms of precision and consistency.

**Table 21:** Description of CEC2022 benchmark problems.

Problem No	Problem name	Dim	Range	F_min_
CEC2022 – C_1_	Shifted and Full Rotated Zakharov	20	[−100,100]	300
CEC2022 – C_2_	Shifted and Full Rotated Rosenbrock	20	[−100,100]	400
CEC2022 – C_3_	Shifted and Full Rotated Expanded Schaffer’s F6	20	[−100,100]	600
CEC2022 – C_4_	Shifted and Full Rotated Non-continuous Rastrigin	20	[−100,100]	800
CEC2022 – C_5_	Shifted and Full Rotated Levy	20	[−100,100]	900
CEC2022 – C_6_	Hybrid Function 1	20	[−100,100]	1800
CEC2022 – C_7_	Hybrid Function 2	20	[−100,100]	2000
CEC2022 – C_8_	Hybrid Function 3	20	[−100,100]	2200
CEC2022 – C_9_	Composition Function 1	20	[−100,100]	2300
CEC2022 – C_10_	Composition Function 2	20	[−100,100]	2400
CEC2022 – C_11_	Composition Function 3	20	[−100,100]	2600
CEC2022 – C_12_	Composition Function 4	20	[−100,100]	2700

### Exploration-exploitation analysis of the proposed AQSCA algorithm

**Fig. 11:**
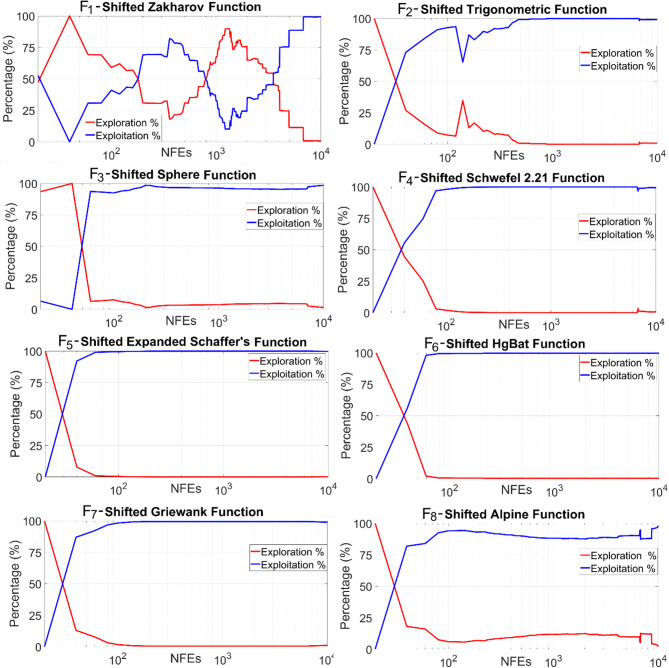
Exploration – Exploitation curves for various test problems for the AQSCA algorithm.

Evaluating optimization performance solely based on prediction results can be misleading. The assessment should also include exploration and exploitation evaluations, which help better understand why some metaheuristic algorithms fail at certain stages and perform poorly on specific problems. To evaluate the AQSCA, this study conducted an empirical analysis of its search features, focusing on their influence on exploration and exploitation. The resulting solutions are then discussed. To further analyze these two key factors in assessing AQSCA’s efficiency, eight standard benchmark functions and their shifted versions are used. The primary importance of shifted test problems in evaluating metaheuristic algorithms lies in their ability to test shift invariance. This key property ensures the algorithm produces robust and accurate solutions regardless of the optimum’s location. Shifting becomes essential in preventing algorithms from relying on predictable landscapes, forcing them to demonstrate true capabilities across varied and high-dimensional search spaces. This evaluation enhances the realism of benchmark cases, allowing researchers to explore the advantages and limitations of the algorithms more effectively. Figure 11 provides a detailed schematic analysis of the exploration and exploitation dynamics within the proposed AQSCA across eight shifted benchmark functions, each with a 100D search space and 10,000 function evaluations (NFEs). In this context, the exploration percentage (red line) indicates the search’s efforts to diversify and find promising new regions. In contrast, the exploitation percentage (blue line) shows efforts focused on refining the best current solutions. These ratios complement each other, summing to 100% at each NFE point, and are plotted logarithmically on the x-axis to clearly illustrate the shift from initial randomization to convergence. This schematic is vital for understanding AQSCA’s behavior, especially how it adaptively balances global exploration and local exploitation through iterations. The structure of AQSCA, which combines AQUILA and SCA, is crucial for maintaining this balance. AQUILA models eagle hunting behavior, emphasizing exploration early and shifting to exploitation later. SCA contributes additional adjustments using fluctuating sine and cosine functions for position updates, enabling adaptive control of movements. This hybrid approach mitigates the risk of AQUILA over-exploring in unimodal landscapes and of SCA converging prematurely in multimodal ones. The curves demonstrate AQSCA’s ability to sustain high exploration initially, helping it escape local optima, then transition to exploitation for precise convergence. F_1_ - Shifted Zakharov Function is a unimodal, convex function with no local minima other than the global optimum, making it ideal for assessing exploitation in higher-dimensional, smooth landscapes that quadratically scale with dimensions. The stepwise pattern in these curves reflects AQSCA’s hybrid design, which uses Levy flights for exploration and SCA-based refinements for exploitation, preventing unnecessary diversification in unimodal settings. F_2_ – Shifted Trigonometric Function is a unimodal test with a single global optimum. Its curve indicates exploration declines smoothly from 100% to 0% by a certain number of function evaluations, while exploitation steadily increases. F_3_ – Shifted Sphere Function is a classic unimodal function emphasizing pure exploitation within a parabolic basin, where premature exploration can delay convergence. Exploration percentages reach a minimum at around 10,000 function evaluations and stay there with minimal fluctuations, while exploitation rises linearly and becomes dominant after 10,000 evaluations. F_4_ – Shifted Schwefel 2.21 is a convex, separable, and unimodal function, helpful in evaluating variable interdependencies within a bounded domain and often challenging due to its asymmetric search space. Exploration decreases to its lowest point between 100 and 1000 evaluations, while exploitation peaks within this range. AQSCA’s success in such non-separable spaces results from its SCA-based fitness-scale movements. For shifted multimodal functions, from F_5_ - Shifted Expanded Schaffer’s to F_8_ - Shifted Alpine, similar convergence traits are observed. Exploration remains high for a longer period, with gradual fluctuations beyond 100 evaluations, as AQUILA’s spiral motions and Levy flights help escape local optima. At the same time, exploitation increases steadily, overtaking around 100 NFEs thanks to SCA’s oscillatory updates, which refine local solutions throughout the iterations.

### Ablation analysis and runtime complexity of the proposed AQSCA

This section provides a detailed analysis of the ablated versions of the proposed AQSCA algorithms and compares their performance when one of the two main parts of the search scheme is removed from the main algorithm. Additionally, it compares the average running time of each ablated algorithm, along with standard AQUILA and the proposed AQSCA, and investigates the most influential factors that determine the execution time of each algorithm. Table 22 exhibits the results of the ablation analysis concerning the proposed hybrid AQSCA optimization algorithm, evaluated across eight shifted variants of the 100D standard test problems. Eight shifted versions of standard test problems were executed thirty times each, with 10,000 function evaluations, to analyze how different components of the proposed search scheme, integrated into the original AQUILA optimizer, influence the overall solution quality of the hybrid AQSCA algorithm. Within this framework, AQSCA1 denotes the activation of only the initial part of the proposed search scheme, as outlined in Table 3, during iterations, whereas AQSCA2 pertains to the activation of only the second part. Additionally, Table 22 presents a comparison of the mean runtimes of each optimizer, over thirty independent runs, for each test function. It is discernible that AQSCA yields the most accurate and robust predictions across all instances, markedly outperforming the ablated AQSCA variants and the standard AQUILA algorithm. This superior performance primarily results from the synergistic integration of AQUILA and SCA algorithms, both of which are engineered to manage the challenges associated with shifting the global optimum. The robust exploration capability of the AQUILA algorithm, combined with the adaptive refinement mechanisms of the SCA algorithm, sustains an appropriate balance between diversification and intensification phases, thus facilitating a more consistent search process. This methodology permits the algorithm to attain the shifted optimum without premature convergence. The ablation analysis further corroborates that the two complementary components of the search scheme possess distinctive advantages that render their combination effective. It is also observed that AQSCA1 and AQSCA2 exhibit comparable computational times, indicating that their application does not impose additional computational burdens on the primary AQSCA algorithm, as evidenced by the average runtimes for each test function. A comparison of the average runtimes between AQSCA and the standard AQUILA algorithm reveals that the proposed method incurs slightly increased calculation times due to the incorporation of the additional scheme. Nevertheless, the overall enhancement in solution quality achieved by AQSCA relative to the original AQUILA justifies the increased runtime when addressing these test problems, such that even the worst result obtained by AQSCA is better than those obtained for the AQUILA algorithm for test problems F_2_ – Shifted Trigonometric Function, F_4_ – Shifted Schwefel 2.21 function, F_7_ – Shifted Griewank function, and F_8_ – Shifted Alpine function.

**Table 22:** Comparison of the statistical results and running times for the competitive algorithms.

	AQSCA	AQSCA1	AQSCA2	AQUILA
*F*_*1*_ – Shifted Zakharov function				
Min	1.191E + 00	1.912E + 00	1.656E + 00	2.627E + 00
Mean	3.158E + 02	1.451E + 04	1.525E + 03	8.741E + 03
Std. dev.	4.942E + 02	3.911E + 04	3.201E + 03	2.234E + 04
Max	2.000E + 03	1.768E + 05	1.475E + 04	9.477E + 04
Avg. run time (sec.)	1.231E + 00	1.022E + 00	8.971E-01	5.322E-01
*F*_*2*_ – Shifted Trigonometric function				
Min	1.000E + 00	1.001E + 00	1.000E + 00	1.012E + 00
Mean	1.000E + 00	1.003E + 00	1.004E + 00	1.098E + 00
Std. dev.	2.461E-05	6.846E-03	9.184E-03	1.342E-03
Max	1.000E + 00	1.035E + 00	1.048E + 03	1.112E + 00
Avg. run time (sec.)	1.214E + 00	1.001E + 00	1.042E + 00	4.222E-01
*F*_*3*_ – Shifted Sphere function				
Min	2.603E-09	1.042E-04	2.932E-05	3.532E-04
Mean	6.817E-06	2.883E-04	4.651E-02	3.802E-02
Std. dev.	1.018E-04	3.998E-05	5.396E-02	1.133E-01
Max	3.834E-04	9.542E-04	1.941E-01	6.241E-01
Avg. run time (sec.)	1.282E + 00	1.193E + 00	9.372E-01	5.953E-01
*F*_*4*_ – Shifted Schwefel 2.21 function				
Min	2.060E-06	3.926E-02	2.792E-03	4.123E-03
Mean	7.783E-05	9.223E-02	4.844E-02	5.782E-02
Std. dev.	5.029E-05	5.742E-02	4.361E-02	3.914E-02
Max	1.762E-04	2.781E-01	2.223E-01	1.508E-01
Avg. run time (sec.)	9.852E-01	7.89E-01	6.242E-01	3.826E-01
*F*_*5*_ – Shifted Expanded Schaffer’s F6 function				
Min	1.352E + 00	2.213E + 00	5.832E + 00	9.932E + 00
Mean	1.024E + 01	3.947E + 01	4.987E + 01	6.892E + 01
Std. dev.	4.106E + 00	4.634E + 00	1.054E + 01	2.483E + 01
Max	1.892E + 01	5.932E + 01	7.639E + 01	1.147E + 02
Avg. run time (sec.)	4.132E + 01	3.838E + 01	3.721E + 01	1.825E + 01
*F*_*6*_ – Shifted HgBat function				
Min	9.387E-02	1.239E-01	4.302E-01	3.024E-01
Mean	4.324E-01	4.781E-01	7.353E-01	7.642E-01
Std. dev.	1.901E-01	1.642E-01	2.169E-01	2.467E-01
Max	6.682E-01	7.308E-01	1.300E + 00	1.378E + 00
Avg. run time (sec.)	2.272E + 00	1.984E + 00	1.782E + 00	1.003E + 00
*F*_*7*_ – Shifted Griewank function				
Min	1.093E-11	1.073E-07	1.577E-06	2.278E-07
Mean	7.352E-09	7.758E-05	1.842E-04	1.055E-04
Std. dev.	9.093E-09	1.427E-04	2.664E-04	1.942E-04
Max	3.802E-08	7.183E-04	9.613E-04	8.903E-04
Avg. run time (sec.)	5.732E-01	4.968E-01	4.942E-01	3.742E-01
*F*_*8*_ – Shifted Alpine function				
Min	8.583E-06	7.643E-03	1.163E-03	3.494E-03
Mean	3.832E-05	3.058E-02	2.791E-02	2.078E-02
Std. dev.	2.967E-05	2.177E-02	1.799E-02	1.484E-02
Max	1.144E-05	8.832E-02	6.131E-02	6.572E-02
Avg. run time (sec.)	9.642E-01	6.762E-01	4.783E-01	1.237E-01

### Comparative evaluations of constrained engineering design problems

This section aims to discuss the effectiveness of the proposed hybrid method in solving three different real-world, complex, and nonlinear constrained engineering design problems with varying levels of difficulty. The respective feasible solutions found by the proposed AQSCA are compared with those obtained from AQUILA, Runge-Kutta Optimizer (RUNGE)^96^, Poor and Rich Optimizer (PRO)^97^, Reptile Search Optimizer (REPTILE)^98^, EQUIL, and Gradient-based Optimizer (GRAD)^99^. Each algorithm is run 100 times for 20,000 function evaluations, and feasible solutions that obey the defined problem constraints are considered for performance evaluations. Statistical analysis of the consecutive algorithm runs is performed for the abovementioned algorithms in terms of best, worst, mean, and standard deviation results. The constrained optimization problem is converted into an unconstrained optimization problem using a simple, yet effective constraint-handling strategy proposed by Kim et al.^100^, as formulated by the mathematical expressions below.

Assume that a nonlinear optimization problem is defined by the following

25 $$\:\text{arg}\text{min}f\left(x\right),\:x\in\:S\subseteq\:\:{R}^{np}$$

subject to inequality constraints

26 $$\:{g}_{k}\left(\overrightarrow{x}\right){\le\:0,\:{\:g}_{k\:}:{R}^{np}\to\:R,}_{}\:k=1,\dots\:,m$$

Where S donates the solution search space, np is the problem dimension, and m is the number of design constraints. A reasonable constraint handling model is developed for converting the defined constrained problem to an unconstrained problem

27 $$\min _{{x \in S}} F\left( x \right) = \left\{ {\begin{array}{*{20}l} {g_{{\max }} \left( {\vec{x}} \right),} & {ifg_{{\max }} \left( {\vec{x}} \right) > 0} \\ {a\tan \left( {f\left( {\vec{x}} \right)} \right) - \frac{\pi }{2}} & {otherwise} \\ \end{array} } \right.$$

where

28 $$\:{g}_{max}\left(\overrightarrow{x}\right)=max\left({g}_{1}\left(\overrightarrow{x}\right),{g}_{2}\left(\overrightarrow{x}\right),\dots\:,{g}_{k}\left(\overrightarrow{x}\right)\right)$$

Where atan() is the arctan function that operates the inverse tangent function of a variable. This constraint-handling technique avoids using problem-dependent parameters such as penalty coefficient or Lagrange multiplier.

### Heat exchanger design problem

This real-world engineering problem involves minimizing the heat exchange area of a heat exchange network with known inlet temperatures at the hot side. The network is composed of three series of operating shell and tube heat exchangers whose mathematical model is formulated by the following set of equations^101^

29 $$\:\text{arg}\text{min}\:\:\:\:\:\:\:f\left(\overrightarrow{x}\right)={x}_{1}+{x}_{2}+{x}_{3}\:$$

Subject to

$$\:{g}_{1}\left(\overrightarrow{x}\right)=-1+0.0025\left({x}_{4}+{x}_{6}\right)\le\:0$$

$$\:{g}_{2}\left(\overrightarrow{x}\right){=-1+0.0025(-x}_{4}+{x}_{5}+{x}_{7})\le\:0$$

$$\:{g}_{3}\left(\overrightarrow{x}\right){=-1+0.01(-x}_{5}+{x}_{8})\le\:0$$

$$\:{g}_{4}\left(\overrightarrow{x}\right)=100{x}_{1}-{x}_{1}{x}_{6}+833.33252{x}_{4}\le\:0$$

$$\:{g}_{5}\left(\overrightarrow{x}\right)={x}_{2}{x}_{4}-{x}_{2}{x}_{7}-1250{x}_{4}+1250{x}_{5}\le\:0$$

$$\:{g}_{6}\left(\overrightarrow{x}\right)={x}_{3}{x}_{5}-{x}_{3}{x}_{8}-2500{x}_{5}+1250000\le\:0.$$

Upper and lower bounds

$$\:100\le\:{x}_{1}\le\:10000,\:\:\:1000\le\:{x}_{2}\le\:10000\:$$

$$\:1000\le\:{x}_{3}\le\:10000,\:\:\:10\le\:{x}_{4}\le\:1000$$

$$\:10\le\:{x}_{5}\le\:1000,\:\:\:10\le\:{x}_{6}\le\:1000$$

$$\:10\le\:{x}_{7}\le\:1000,\:\:\:10\le\:{x}_{8}\le\:1000.$$

**Fig. 12:**
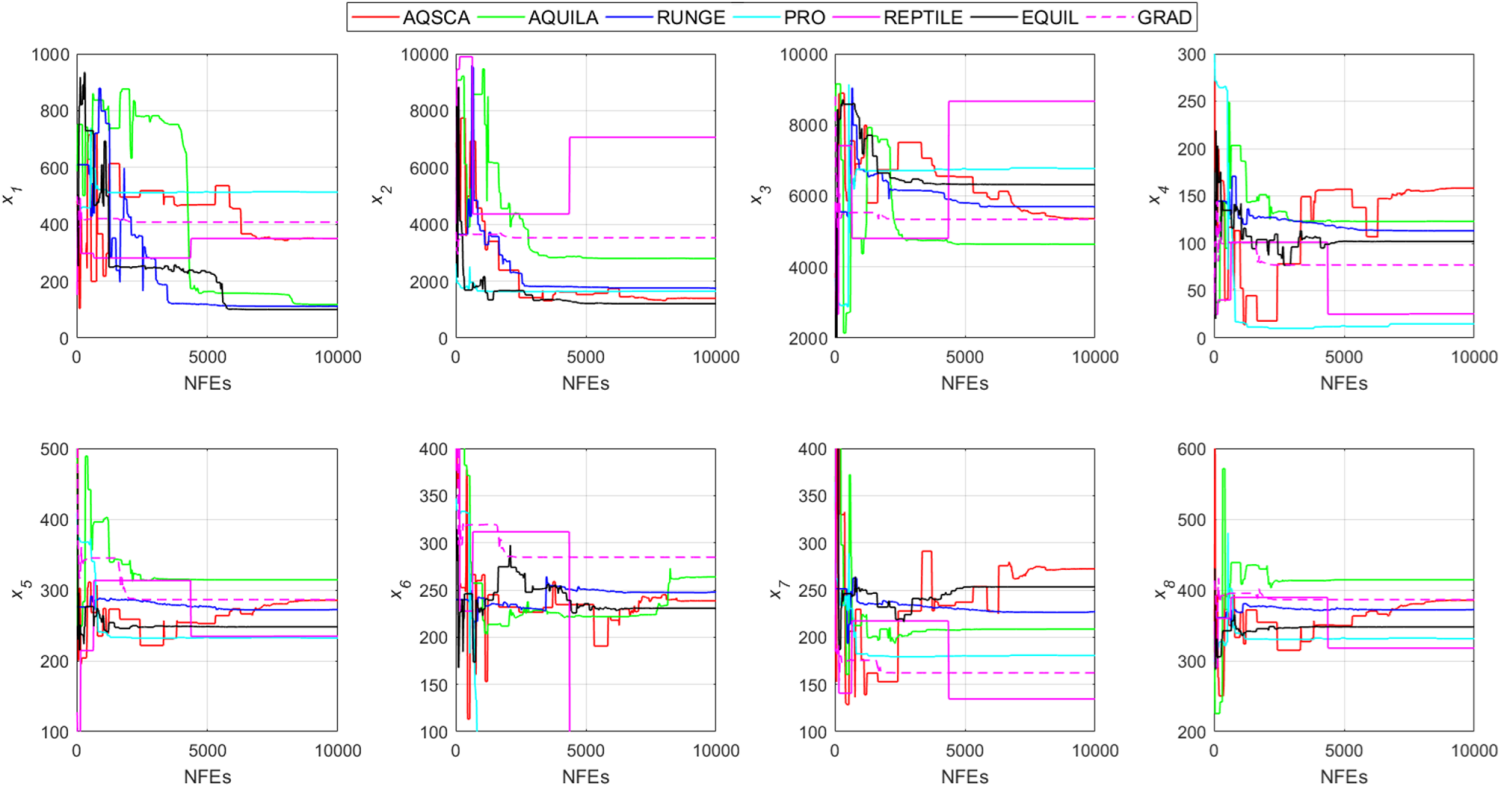
Evolution of the design variables with increasing function evaluations.

Table 23 reports the optimal results of the AQSCA algorithm and other remaining optimizers for this design problem. The best-known optimal value of this problem is given in Andrei^101^ as f(x) = 7049.24802. All compared algorithms fail to approach the most prominent solution to the problem. However, AQSCA achieves the minimum objective value of *f(x*) = 7103.44937 between them with their respective design variables $\:\overrightarrow{x}=\{349.71,\:\:1394.40,\:\:5359.33,\:\:158.12,\:\:285.62,\:\:238.52,\:\:272.42,\:\:385.62\}.$ The REPTILE algorithm is the worst predictor in terms of solution accuracy and consistency, with a corresponding minimum function value of f(x) = 16073.1682, consistently finding the same optimal solution in each run. It is also interesting to observe the performance improvement resulting from integrating the proposed scheme into the standard AQUILA algorithm, as evidenced by the estimations acquired by AQUILA, which yield the optimal value of f(x) = 7554.933. This significant increase in solution accuracy in the standard AQUILA optimizer verifies the dexterity of the proposed learning algorithm. Figure 12 shows the convergence histories of the design variables with increasing iterations.

**Table 23:** Statistical results for heat exchanger design problem.

	AQSCA	AQUILA	RUNGE	PRO	REPTILE	EQUIL	GRAD
*x*_*1*_	349.716645	117.585131	111.520963	513.32232	350.07645	100.07559	407.630083
*x*_*2*_	1394.40074	2797.74739	1745.68940	1641.8242	7058.9251	1207.0493	3524.78278
*x*_*3*_	5359.33195	4639.60095	5693.47034	6768.4227	8664.1665	6313.0133	5339.08673
*x*_*4*_	158.123079	123.049523	113.240972	14.858360	25.570234	101.81062	77.0467263
*x*_*5*_	285.627207	314.471656	272.336639	232.23111	234.77242	248.01661	286.436533
*x*_*6*_	238.521569	263.844073	247.814979	87.778583	93.567720	230.14599	284.813291
*x*_*7*_	272.425844	208.574794	227.192715	180.64079	134.70855	253.62034	162.254234
*x*_*8*_	385.627095	414.462656	372.327863	331.29404	318.02354	347.97917	386.436530
*g*_*1*_ *(x)*	−0.00009024	−0.0018628	−0.1972176	−1.435412	−1.8248044	−0.499985	−0.81364768
*g*_*2*_ *(x)*	−0.00000975	−0.0000007	−0.0001384	−0.001583	−0.5351748	−0.001582	−0.06749426
*g*_*3*_ *(x)*	−0.00000034	−0.0000506	−0.0000654	−0.000480	−0.0211339	−0.000503	0.00000000
*g*_*4*_ *(x)*	−0.00838838	−0.0327660	−0.0973601	−0.743407	−0.7021551	−0.170108	−0.09534996
*g*_*5*_ *(x)*	−0.00017507	−0.0000076	−0.0342790	−0.004966	−0.1402231	−0.000434	−0.07088990
*g*_*6*_ *(x)*	−0.00000112	−0.0000902	−0.0000877	−0.009370	−0.1674887	−0.000374	0.00000000
Min	7103.44937	7554.93342	7550.68074	8923.5693	16073.1682	7620.1383	9271.499600
Mean	7296.65487	8528.88786	8024.55843	11771.351	16073.1682	8311.9900	11340.30811
Std. Dev	145.248604	914.458300	350.707793	2466.7322	0.00000000	416.86546	1791.708734
Max	7538.79754	10260.6148	8606.19641	14864.024	16073.1682	9083.0504	12390.32289

### Industrial refrigeration system design problem

This complex optimization problem simulates the mathematical model of an industrial refrigeration system, first proposed by Paul and Tay^102^. The current form of the objective function and respective design constraints are expressed below.

30 $$\begin{aligned} \arg \min f\left( {\vec{x}} \right) = & 63098.88x_{2} x_{4} x_{{12}} + 5441.5x_{2}^{2} x_{{12}} \\ & + 115055.5x_{2}^{{1.664}} x_{6} + 6172.27x_{2}^{2} x_{6} \\ & + 3098.88x_{1} x_{3} x_{{11}} + 5441.5x_{1}^{2} x_{{11}} + 115055.5x_{1}^{{1.664}} x_{5} \\ & + 6172.27x_{1}^{2} x_{5} + 140.53x_{1} x_{{11}} \\ & + 281.29x_{3} x_{{11}} + 70.26x_{1}^{2} + 281.29x_{1} x_{3} \\ & + 281.20x_{3}^{2} + 14437x_{1}^{2} x_{7} x_{8}^{{1.8812}} x_{9}^{{ - 1}} x_{{10}} x_{{12}}^{{0.3424}} x_{{14}}^{{ - 1}} \\ & + 20470.2x_{1}^{2} x_{7}^{{2.893}} x_{{11}}^{{0.316}} \\ \end{aligned}$$

subject to.

$$\:{g}_{1}\left(\overrightarrow{x}\right)=1.524{x}_{7}^{-1}-1\le\:0,\:\:{g}_{2}\left(\overrightarrow{x}\right)=1.524{x}_{8}^{-1}-1\le\:0,\:$$

$$\:{g}_{3}\left(\overrightarrow{x}\right)=0.07789{x}_{1}-2{x}_{7}^{-1}{x}_{9}-\:1\le\:0,\:\:\:{g}_{4}\left(\overrightarrow{x}\right)=7.05305{x}_{1}^{2}{x}_{2}^{-1}{x}_{8}^{-1}{x}_{9}^{-1}{x}_{10}{x}_{14}^{-1}-1\:\:\le\:0$$

$$\:{g}_{5}\left(\overrightarrow{x}\right)=0.0833{x}_{13}^{-1}{x}_{14}-1\:\le\:0,$$

$$\:{g}_{6}\left(\overrightarrow{x}\right)=47.136{x}_{2}^{0.33}{x}_{10}^{-1}{x}_{12}-1.333{x}_{8}{x}_{13}^{2.1195}+62.08{x}_{8}^{0.2}\:{x}_{10}^{-1}{x}_{12}^{-1}{x}_{13}^{2.1195}-1\le\:0$$

$$\:{g}_{7}\left(\overrightarrow{x}\right)=0.0477{x}_{8}^{1.8812}{x}_{10}{x}_{12}^{0.3424}-1\:\le\:0,\:\:\:{g}_{8}\left(\overrightarrow{x}\right)=0.0488{x}_{7}^{1.893}{x}_{9}{x}_{11}^{0.316}-1\:\le\:0$$

$$\:{g}_{9}\left(\overrightarrow{x}\right)=0.0099{x}_{1}{x}_{3}^{-1}\:-1\:\le\:0,\:\:\:\:\:\:\:\:\:\:{g}_{10}\left(\overrightarrow{x}\right)=0.0193{x}_{2}{x}_{4}^{-1}\:-1\:\le\:0$$

$$\:{g}_{11}\left(\overrightarrow{x}\right)=0.0298{x}_{1}{x}_{5}^{-1}\:-1\:\le\:0,\:\:{g}_{12}\left(\overrightarrow{x}\right)=0.056{x}_{2}{x}_{6}^{-1}\:-1\:\le\:0$$

$$\:{g}_{13}\left(\overrightarrow{x}\right)=2{x}_{9}^{-1}\:-1\:\le\:0,\:\:\:\:{g}_{14}\left(\overrightarrow{x}\right)=2{x}_{10}^{-1}\:-1\:\le\:0,\:\:\:\:{g}_{15}\left(\overrightarrow{x}\right)={x}_{12}{x}_{11}^{-1}\:-1\:\le\:0$$

$$\:0.001\le\:{x}_{i}\le\:5,\:\:\:\:i=1,\dots\:.,14$$

**Fig. 13:**
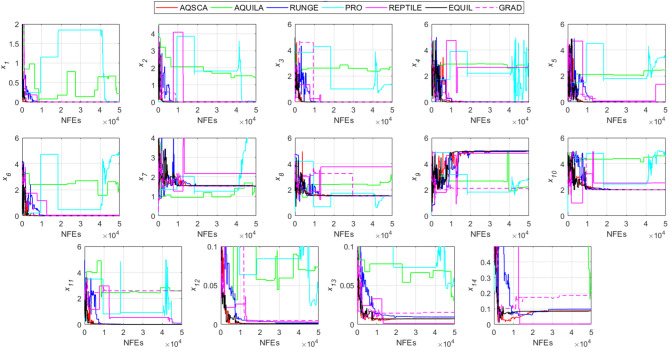
Convergence curves of the decision variables for the industrial refrigeration design problem.

Table 24 reports the optimal results retained by AQSCA and other competitive optimization algorithms. It is observed that the solution quality and consistency achieved by AQSCA are significantly better than those obtained from the remaining algorithms. The failure of AQUILA, PRO, REPTILE, and GRAD algorithms to capture accurate predictions for this problem is due to their inability to solve nonlinear optimization problems with binding design constraints. The persistence of the predictions performed by AQSCA is remarkable and much better than that of the remaining algorithms. AQSCA achieves the minimum objective function f(x) = 0.0322355 with a respective standard deviation rate of 0.0001405, considerably outperforming the contestant algorithms. As in the previous case, it is observed that the hybridization of the proposed learning scheme with the original AQUILA algorithm substantially increases the general estimation performance. Figure 13 visualizes the evolution of the design variables for each algorithm for this design problem.

**Table 24:** Comparison of the statistical results for the industrial refrigeration system design problem.

	AQSCA	AQUILA	RUNGE	PRO	REPTILE	EQUIL	GRAD
*x*_*1*_	0.0010000	0.2230191	0.0010000	0.0093770	0.0011671	0.001000	0.0010058
*x*_*2*_	0.0010014	1.4644140	0.0010001	0.0288833	0.0194901	0.001084	0.0011461
*x*_*3*_	0.0010004	2.7059838	0.0010319	1.3107969	0.0035212	0.001084	0.0010307
*x*_*4*_	0.0010134	2.6066739	0.0197763	3.0500354	2.6614174	0.001992	0.0012479
*x*_*5*_	0.0010070	2.4332023	0.0120954	3.3875874	1.3630796	0.001006	0.0010250
*x*_*6*_	0.0010054	2.2007491	0.0046441	4.6404930	0.0514722	0.001057	0.0029327
*x*_*7*_	1.5240000	1.5241881	1.5240020	4.1898463	2.1723434	1.524304	1.5240000
*x*_*8*_	1.5240000	2.9328247	1.5240157	1.5245133	3.7694761	1.525172	1.5240000
*x*_*9*_	4.9999997	2.2394464	4.9989289	2.7222870	4.8103557	4.981438	2.1236207
*x*_*10*_	2.0000095	4.6035436	2.0000062	4.6475565	2.5344107	2.003078	2.0005782
*x*_*11*_	0.0010069	2.5655983	0.0037727	0.0485353	0.1034858	0.001020	2.5933853
*x*_*12*_	0.0010066	0.0735072	0.0018812	0.0455211	0.0031357	0.001013	0.0050080
*x*_*13*_	0.0073160	0.0298123	0.0095186	0.0533784	0.0011493	0.007286	0.0155264
*x*_*14*_	0.0878272	0.2173488	0.0982802	0.6066652	0.0035503	0.087250	0.1863915
*g*_*1*_ *(x)*	0.0000000	−0.000123	−0.0000013	−0.636263	−0.2984534	−0.0001994	0.0000000
*g*_*2*_ *(x)*	0.0000000	−0.480364	−0.0000103	−0.000336	−0.5956997	−0.0007685	0.0000000
*g*_*3*_ *(x)*	−7.561601	−3.921172	−7.5601877	−2.298738	−5.4286338	−7.5359395	−3.7868255
*g*_*4*_ *(x)*	−0.978953	−0.227488	−0.9811620	−0.960365	−0.9805913	−0.9803251	−0.9793513
*g*_*5*_ *(x)*	−0.000001	−0.392696	−0.1399230	−0.053265	−0.7426809	−0.0024792	0.00000000
*g*_*6*_ *(x)*	−0.000019	−0.014812	−0.0631363	−0.221357	−0.9782950	−0.0166147	−0.0000454
*g*_*7*_ *(x)*	−0.980154	−0.319870	−0.9754161	−0.829818	−0.7961384	−0.9800494	−0.9656155
*g*_*8*_ *(x)*	−0.938803	−0.673144	−0.9071222	−0.230924	−0.5021424	−0.9387481	−0.6890652
*g*_*9*_ *(x)*	−0.990104	−0.999184	−0.9904062	−0.999929	−0.9967184	−0.9908662	−0.9903386
*g*_*10*_ *(x)*	−0.980927	−0.989157	−0.9990239	−0.999817	−0.9998586	−0.9894973	−0.9822739
*g*_*11*_ *(x)*	−0.970407	−0.997268	−0.9975362	−0.999917	−0.9999744	−0.9703838	−0.9707561
*g*_*12*_ *(x)*	−0.944220	−0.962736	−0.9879407	−0.999651	−0.9787954	−0.9426027	−0.9781137
*g*_*13*_ *(x)*	−0.599999	−0.106922	−0.5999143	−0.265323	−0.5842303	−0.5985095	−0.0582122
*g*_*14*_ *(x)*	−0.000004	−0.565552	−0.0000031	−0.569666	−0.2108619	−0.0015366	−0.0002890
*g*_*15*_ *(x)*	−0.000291	−0.971348	−0.5013585	−0.062103	−0.9696984	−0.0069406	−0.9980689
Min	0.0322355	688531.44	0.0595853	2506.5870	31.885900	0.03305483	1.4443998
Mean	0.0323734	688531.44	0.1687599	2506.5870	456791.51	0.04292656	11887.412
Std. Dev	0.0001405	0.0000000	0.0995829	0.000000	727036.73	0.00785083	22333.134
Max	0.0325918	688531.44	0.3660385	2506.5870	1730329.2	0.05525244	51501.972

### Car side impact design problem

**Fig. 14:**
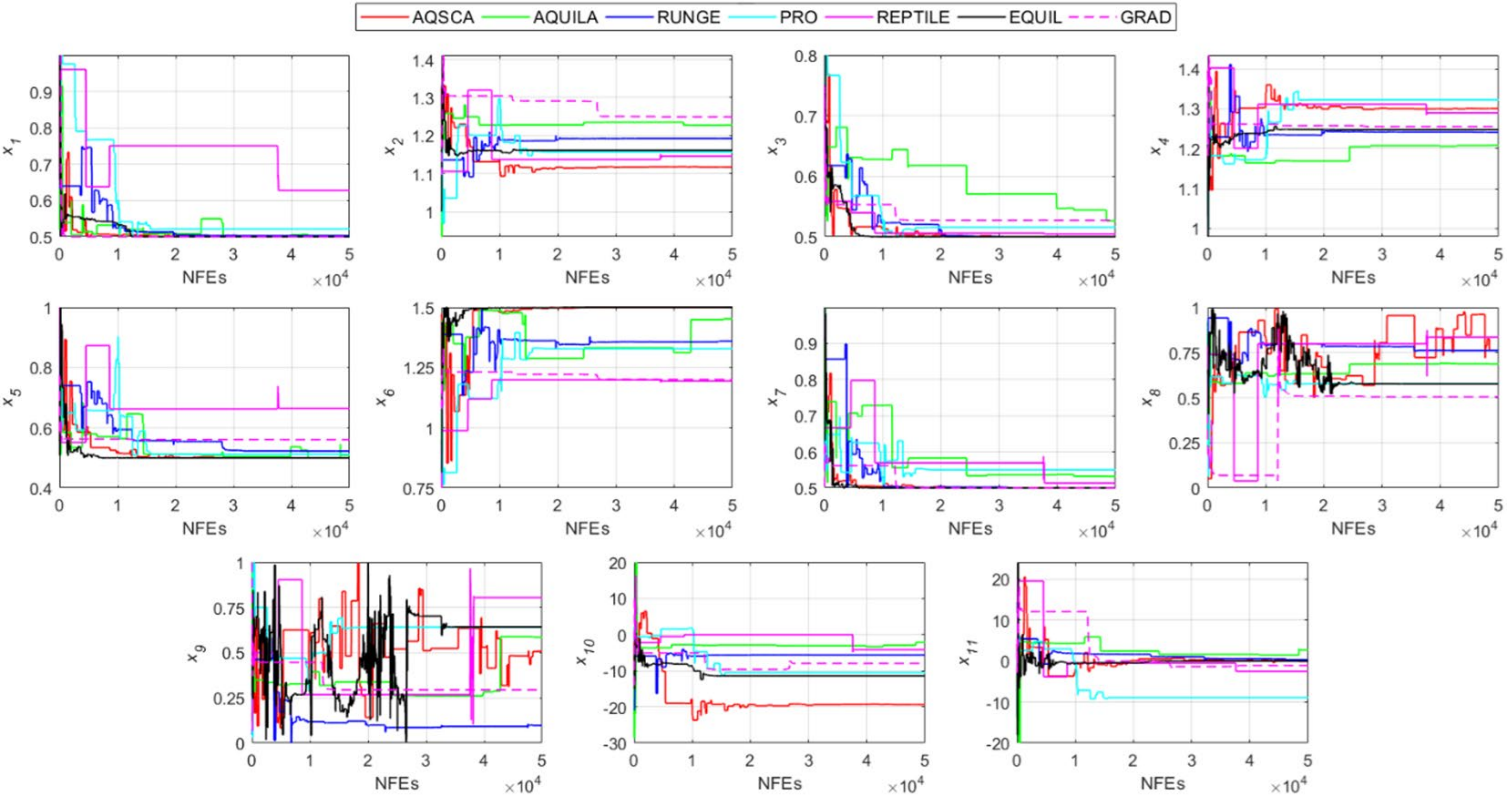
Convergence histories of the design variables for the car side impact design problem.

This design problem was initially proposed by Gu et al.^103^. A formative side impact criterion is defined to regulate the vehicle market and maintain reliable protection against side impact. For this aim, a promising attempt was made to reduce the total weight of a vehicle using nine decisive impact factors, which include the thickness of B-pillar (*x*_*1*_), The B-pillar reinforcement (*x*_*2*_), the floor side inner (*x*_*3*_), the cross members (*x*_*4*_), the door beam (*x*_*5*_), the door beltline reinforcement (*x*_*6*_), the roof rail (*x*_*7*_), the materials of B-pillar inner (*x*_*9*_), the barrier height (*x*_*10*_), and the hitting position (*x*_*11*_). This is a typical mechanical design problem with mixed continuous and integer decision variables. The following mathematical formulation describes the car side impact design problem.

31 $$\begin{aligned} \:Weight = & f\left( {\vec{x}} \right) = 1.98 + 4.9x_{1} + 6.67x_{2} + 6.98x_{3} + 4.01x_{4} \\ & + 1.78x_{5} + 2.73x_{7} \:subject\:toF_{a} = g_{1} \left( {\vec{x}} \right) = 1.16 - 0.3717x_{2} x_{4} \\ & - 0.00931x_{2} x_{{10}} - 0.484x_{3} x_{9} + 0.1343x_{6} x_{{10}} \le 1kNVC_{a} = g_{2} \left( {\vec{x}} \right) \\ = & 0.261 - 0.0159x_{1} x_{2} - 0.188x_{1} x_{8} - 0.019x_{2} x_{7} + 0.0144x_{3} x_{5} \\ & + 0.0008757x_{5} x_{{10}} + 0.08045x_{6} x_{9} + 0.00139x_{8} x_{{11}} + 0.00001575x_{{10}} x_{{11}} \\ & \le 0.32\frac{m}{s}VC_{m} = g_{3} \left( {\vec{x}} \right) = 0.214 + 0.00817x_{5} - 0.131x_{1} x_{8} - 0.0704x_{1} x_{9} \\ & + 0.03099x_{2} x_{6} - 0.018x_{2} x_{7} + 0.0208x_{3} x_{8} + 0.121x_{3} x_{9} \\ & - 0.00364x_{5} x_{6} + 0.0007715x_{5} x_{{10}} - 0.0005354x_{6} x_{{10}} \\ & + 0.00121x_{8} x_{{11}} + 0.00184x_{9} x_{{10}} - 0.02x_{2}^{2} \le 0.32\frac{m}{s}VC_{l} = g_{4} \left( {\vec{x}} \right) \\ = & 0.74 - 0.61x_{2} - 0.163x_{3} x_{8} + 0.001232x_{3} x_{{10}} - 0.166x_{7} x_{9} \\ & + 0.277x_{2}^{2} \le 0.32\frac{m}{s}\Delta _{{ur}} = g_{5} \left( {\vec{x}} \right) = 28.98 + 3.818x_{3} - 4.2x_{1} x_{2} \\ & + 0.0207x_{5} x_{{10}} + 6.63x_{6} x_{9} - 7.7x_{7} x_{8} + 0.32x_{9} x_{{10}} \le 32\frac{m}{s}\Delta _{{mr}} \\ = & g_{6} \left( {\vec{x}} \right) = 33.86 + 2.95x_{3} + 0.1792x_{{10}} - 5.05x_{1} x_{2} - 11x_{2} x_{8} \\ & - 0.0215x_{5} x_{{10}} - 9.98x_{7} x_{8} + 22x_{8} x_{9} \le 32\frac{m}{s}\Delta _{{l_{r} }} = g_{7} \left( {\vec{x}} \right) \\ & = 46.36 - 9.9x_{2} - 12.9x_{1} x_{8} + 0.1107x_{3} x_{{10}} \le 32 - F_{P} \\ = & g_{8} \left( {\vec{x}} \right) = 4.72 - 0.5x_{4} - 0.19x_{2} x_{3} - 0.0122x_{4} x_{{10}} + 0.009325x_{6} x_{{10}} \\ & + 0.000191x_{{11}}^{2} \le 32.4\:kN\;V_{{MBP}} = g_{9} \left( {\vec{x}} \right) = 10.58 - 0.674x_{1} x_{2} \\ & - 1.95x_{2} x_{8} + 0.02054x_{3} x_{{10}} + 0.0198x_{4} x_{{10}} + 0.028\:x_{6} x_{{10}} \le 9.9V_{{FD}} \\ = & g_{{10}} \left( {\vec{x}} \right) = 16.45 - 0.489x_{3} x_{7} - 0.843x_{5} x_{6} + 0.0432x_{9} x_{{10}} \\ & - 0.0556x_{9} x_{{11}} - 0.000786x_{{11}}^{2} \le 15.7 - 0.5 \le x_{i} \le 1.5,\:i \\ = & 1, \ldots \:,7x_{8} ,x_{9} \in \left( {{\text{0}}{\text{.192,0}}{\text{.345}}} \right) - 30 \le x_{{10}} ,\:x_{{11}} \le 30 \\ \end{aligned}$$

here *F*_*a*_ is the abdominal load, *VC*_*u*_ is the upper chest of the dummy, *VC*_*m*_ is the middle chest of the dummy, *VC*_*l*_ is the lower chest of the dummy, *Δ*_*ur*_ is the deflection of the upper rib, *Δ*_*mr*_ is the deflection of the middle rib, *Δ*_*lr*_ is the deflection of the lower rib, *F*_*p*_ is the public force, *V*_*MBP*_ is the V-Pillar velocity at the center, and *V*_*FD*_ is the front door velocity at the V-Pillar. Table 25 provides the optimal solution found by the competitive optimizers, including the proposed AQSCA. Regarding solution accuracy and persistence, the proposed hybrid AQSCA algorithm dominates. It achieves the best feasible objective function value of f(x) = 22.843049 and a standard deviation value of 0.0002035, which is significantly better than the values obtained by the compared methods. GRAD algorithm yields the worst minimum prediction value of f(*x*) = 23.852970 for this design case. The convergence of the design variables to their optimal values for each compared algorithm is depicted in Fig. 14 for this design problem.

**Table 25:** Optimal results for the car side impact design problem.

	AQSCA	AQUILA	RUNGE	PRO	REPTILE	EQUIL	GRAD
*x*_*1*_	0.500000	0.503483	0.503166	0.521394	0.628002	0.500000	0.500072
*x*_*2*_	1.117423	1.227737	1.192787	1.157382	1.146087	1.161808	1.249328
*x*_*3*_	0.500004	0.526423	0.500002	0.515665	0.504654	0.500038	0.527481
*x*_*4*_	1.300449	1.208172	1.241194	1.322470	1.289807	1.247374	1.255557
*x*_*5*_	0.500000	0.508327	0.522635	0.511863	0.664488	0.500000	0.560666
*x*_*6*_	1.499999	1.452353	1.359759	1.327604	1.194102	1.499999	1.200448
*x*_*7*_	0.500000	0.532318	0.500004	0.550195	0.513453	0.500000	0.500000
*x*_*8*_	0.345000	0.345000	0.345000	0.345000	0.345000	0.345000	0.345000
*x*_*9*_	0.345000	0.345000	0.192000	0.345000	0.345000	0.345000	0.192000
*x*_*10*_	−19.37213	−2.116638	−5.637923	−10.5374	−4.173905	−11.43262	−7.950057
*x*_*11*_	−0.032508	2.723213	0.378313	−8.88292	−2.478593	0.000306	−1.036399
*g*_*1*_ *(x)*	−0.615320	−0.457360	−0.477108	−0.531181	−0.458755	−0.531789	−0.507769
*g*_*2*_ *(x)*	−0.078258	−0.073438	−0.075783	−0.080826	−0.083947	−0.075557	−0.080101
*g*_*3*_ *(x)*	−0.092218	−0.095628	−0.099163	−0.103506	−0.109977	−0.093078	−0.106207
*g*_*4*_ *(x)*	−0.021705	−0.021567	−0.019695	−0.022297	−0.015698	−0.020927	−0.025394
*g*_*5*_ *(x)*	−3.678064	−2.696835	−3.047382	−3.468182	−3.669062	−3.201162	−3.421846
*g*_*6*_ *(x)*	−5.260704	−4.324963	−3.313267	−4.130438	−3.572745	−4.129163	−3.857792
*g*_*7*_ *(x)*	−0.986850	−0.835028	−0.993099	−1.009001	−1.225117	−0.986850	−0.288841
*g*_*8*_ *(x)*	0.000000	−0.002935	0.000000	0.000000	−0.014419	0.000000	0.000000
*g*_*9*_ *(x)*	−0.628708	−0.254622	−0.305094	−0.387446	−0.310429	−0.461740	−0.364551
*g*_*10*_ *(x)*	−0.164835	−0.061848	−0.022250	−0.016194	−0.058590	−0.099339	−0.002073
Min	22.843049	23.513332	23.16391	23.570175	23.980756	22.926503	23.852970
Mean	22.843281	23.814149	23.47849	24.487486	24.320548	23.069664	25.148991
Std. Dev	0.0002035	0.2617060	0.195075	0.7005446	0.2375918	0.1364153	0.9585307
Max	22.843558	24.439146	23.72673	25.561008	24.681176	23.318788	26.756085

#### Solving chemical equilibrium problems through the AQSCA optimizer

##### Fundamentals of Gibbs free energy minimization

This section evaluates the optimization success of the proposed AQSCA algorithm over highly nonlinear and inherently complex Gibbs Free Energy (GFE) minimization problems. In recent years, extensive research on designing and optimizing efficient energy systems has garnered significant attention, driven by the advancement of increasingly improved technology. In this regard, determining the equilibrium point in chemical reactions through GFE minimization is essential in evaluating energy systems at specified operating conditions. Biomass gasification and fuel cell applications in fuel reforming processes are prominent examples of employing chemical equilibrium calculations in a specific energy system^104^. Biomass gasification modeling and fuel reformer analysis can be conducted using well-defined reaction kinetics calculations; however, detailed chemical reaction kinetic experimental data are required for every reaction step. Utilizing chemical equilibrium in a steady state and specifying final products are the best approaches for chemical reaction modeling. The chemical equilibrium model is also suitable for fast chemical reactions.

The concept of GFE minimization for chemical equilibrium was first introduced by Dantzig et al.^105^, stating that the necessary condition for a given system to be at the equilibrium point is that the total GFE must be at the global minimum. However, as the GFE function is highly non-linear and non-convex, a reliable and efficient method for global minimization is highly desired. In this context, the thermodynamic quantity of primary significance is the GFE, which allows the description of equilibrium under conditions of constant temperature and pressure. The global minimum of the GFE corresponds to the actual solution for equilibrium^106^. With the growing importance of GFE modeling, researchers have developed various intelligently devised solution procedures, which can be primarily grouped into the sub-categories of deterministic and metaheuristic techniques. Previous literature studies have focused on applying deterministic optimization approaches for minimizing GFE as a restrictive optimization objective, which has been extensively used in various engineering fields. The following works exemplify significant examples of prominent past studies utilizing conventional optimization methods. Neron et al.^107^ implemented Gibbs energy minimization using the Newton–Raphson method to compute the equilibrium temperature and compositions. Mojaver et al.^108^ employed the Lagrange method to minimize the Gibbs free energy and determine the unknown parameters of an integrated energy system. Liu et al.^109^ conducted a detailed review of well-known techniques, such as Lagrange Multipliers, Morley, and Rand methods, used to resolve the Gibbs energy minimization problem.

As a reliable alternative to conventional optimization techniques, metaheuristic algorithms are being applied in various chemical engineering applications, including chemical stability, phase equilibrium, and reactive phase equilibrium problems, through the minimization of Gibbs Free Energy objective functions. Bonilla-Petriciolet et al.^110^ compared the search performances of different variants of Simulated Annealing (SA) algorithms in solving phase stability of reactive and non-reactive mixtures. They concluded that other SA-based methods can be conveniently utilized for phase stability problems. Teh and Rangaiah^111^ used an enhanced Tabu Search algorithm to calculate phase equilibrium calculations by optimizing Gibbs Free Energy functions. They compared the optimum results obtained by this method with those from the widely known Genetic Algorithm method. Moodley et al.^112^ applied the original and modified versions of the Krill Herd Optimization algorithm to phase stability and phase equilibrium calculations of reactive and non-reactive chemical systems. It was found that the improved Krill Herd algorithm and its standard version can effectively cope with the nonlinearities of the governing thermodynamic models for various types of phase equilibrium problems. Bamikole and Narasigadu^113^ aimed to overcome the challenging complexities of reactive phase equilibrium problems by employing newly emerged swarm-based metaheuristic optimizers, including the Honey Badger Algorithm, the Pathfinder Algorithm, the Horse Herd Optimization Algorithm, and the Red Fox Optimization Algorithm. A comparative analysis of the optimization performance of the methods mentioned was conducted using statistical measures. Turgut et al.^114^ introduced a hierarchical Manta-Ray Foraging Optimization algorithm to determine the optimal composition of gas mixtures for a given set of operational conditions.

When viewed from a general perspective, it is understood that heuristic-based solution strategies for solving reactive equilibrium problems can effectively overcome the characteristic functional drawbacks of extreme non-linearities and non-convexities in the governing objective function, compared to conventional derivative-based optimization methods. Therefore, this study aims to shed light on the challenging situation as to which type of optimization method performs well on chemical equilibrium problems and scrutinize the effectiveness of the relatively newly developed metaheuristic algorithms, along with the proposed AQSCA, in finding the equilibrium point of a chemical reaction operated in a particular condition through GFE minimization. The following section gives insights into the preliminaries of finding equilibrium points of the reacting components in a gas mixture through GFE minimization. Briefly, it explains the mathematical background of the governing thermodynamic model.

#### Problem definition

A gas mixture composed of reactive system components at a specific operating temperature and pressure tends to spontaneously proceed toward a state that minimizes the overall Gibbs free energy of all chemically interacting species^115^.

32 $$\:d{\left(G\right)}_{T,p}\le\:0$$

This indicates that the progression of the chemical reaction continues, and mole numbers of the species in the reactants and products are varied until the global minimum value of GFE is attained, reassuring no more change in GFE is possible. The GFE for a system that consists of a multi-component mixture that operates at a given temperature and pressure is stated as follows^113^

33 $$\:G=\sum\:_{j=1}^{NS}{\mu\:}_{j}{n}_{j}$$

Where NS represents the number of components in the chemical reaction; *µ is* designated as the chemical potential of the element, and *n* refers to the mole number of the *i*^*th*^ component. Also, in Eq. (33), the term *G* represents the GFE function that needs to be minimized. In the reacting mixture, the chemical equilibrium occurs when the GFE function attains its global optimum point.

A set of equations is constructed in terms of mole balance constraints as follows^115^.

34 $$\:\sum\:_{j=1}^{NS}{A}_{ij}{n}_{j}-{b}_{i}=0\:i=\text{1,2},3,\dots\:,NA$$

35 $$\:{b}_{i}=\sum\:_{j=1}^{NS}{A}_{ij}{n}_{j}$$

Also,

36 $$\:{b}_{i}-{b}_{i}^{o}=0\:\:\:\:i=\text{1,2},3,\dots\:,NA$$

*NA* is the number of chemical elements; *A*_*ij*_ is the number of kilogram atoms per mole of species *j*. And $b_{i}^{0}$ is the assigned number of kilogram atoms of element *i* per mole of total reactants. Equations 34–35 is developed to provide mole and mass balance equality.

Taking into account the ideal gas of the reactive species in the mixture, the chemical potential *µ* can be specified as follows^113^:

37 $$\:{\mu\:}_{j}={\mu\:}_{j}^{o}+R\cdot\:T\cdot\:ln\left(\frac{{n}_{j}\times\:P}{{n}_{tot}\times\:{P}_{ref}}\right)$$

Where *R* represents the universal gas constant; *n*_*tot*_ refers to the total mole number of the components in the mixture; *P*_*ref*_ stands for the reference pressure; *µ*_*j*_^*0*^ denotes the chemical potential of the pure species *j*; *T* and *P* refer to the working temperature and pressure for which chemical reaction occurs.

The final form of the GFE minimization function is given in^115^.

38 $$\:G=\sum\:_{j=1}^{NS}\left({\mu\:}_{j}{n}_{j}\right)={\sum\:}_{j=1}^{NS}\left({\mu\:}_{j}^{o}+R\cdot\:T\cdot\:ln\left(\frac{{n}_{j}\times\:P}{{n}_{tot}\times\:{P}_{ref}}\right)\right).{n}_{j}$$

Subjected to the mole balance constraints as defined below^115^

39 $$\:\sum\:_{i=1}^{NA}\left({b}_{i}-{b}_{i}^{o}\right)={\sum\:}_{i=1}^{NA}\left({\sum\:}_{j=1}^{NS}{A}_{ij\:}{n}_{j}-{b}_{i}^{o}\right)=0$$

The unconstrained GFE minimization problem is converted into a constrained optimization problem by considering the equality constraints defined in Eq. (39), which ensures the total mass balance in the reactive mixture. The Inverse Tangent Constraint Handling Mechanism^100^ maintains mass balance between the reactive components while minimizing the GFE objective function, as defined in Eq. (38).

#### Illustrative examples

This section evaluates the optimization performance of the proposed AQSCA algorithm over complex and challenging GFE minimization problems. The proposed AQSCA algorithm solves four different minimization cases represented by the definitive gas-phase reactions. The results are benchmarked against more than thirty-five metaheuristic algorithms in the literature. All compared algorithms are run 1000 times for 50,000 function evaluations, and predictive solutions found by the competitive optimizers are assessed in terms of best, worst, mean, and standard deviation results. The decisive term “Success rate (SR)” is also defined as the ratio between the number of feasible solutions (solutions that do not violate any specified constraint) and the total number of solutions generated by the specific metaheuristic optimizer. An algorithm with a higher SR has remarkable optimization accuracy and robustness in this context. All metaheuristic algorithms used in exhaustive comparisons and thermodynamic models for GFE calculations have been developed in a Java environment and are performed on a desktop computer with an Intel CPU processor at 2.50 GHz and 16 GB RAM. More than twenty existing nature-inspired metaheuristic algorithms have been utilized to solve the defined GFE minimization computations. Most of them have failed to provide a feasible solution satisfying the prescribed atom balance constraints in any successive run, and therefore, they are not considered for comparative performance evaluations. Between the competitive optimizers, the favorable algorithms providing the most accurate and feasible solutions are the proposed hybrid AQSCA, African Vultures Optimization Algorithm (AFRICAN)^116^, Crow Search Algorithm (CROW)^117^, GRAD, Manta Ray Foraging Optimizer (MANTA)^118^, Salp Swarm Optimizer (SALP)^119^, and EQUIL; those of which are taking into consideration for the comparative benchmark analysis. The following three case studies are associated with finding the equilibrium mole components in the gas mixture employing the core concepts of GFE minimization through the algorithms as mentioned earlier, including the proposed hybrid optimizer in this research study.

#### Oxidation of methane-propane-ethane gas mixture

Consider a gas-phase mixture that initially involves 1 mol of CH_4_ (Methane), 1 mol of C_2_H_6_ (Ethane), 1 mol of C_3_H_8_ (Propane), 7 moles of O_2_ (Oxygen), and 4 moles of N_2_ (Nitrogen) reacting under predefined operational temperatures varying from 600 K to 2000 K and working pressures from 1 bar to 100 bar. The equation below can express evolving chemical reactions between these gas mixture components.

40 $$\begin{aligned} CH_{4} & + C_{2} H_{6} + C_{3} H_{8} + 7O_{2} + 4N_{2} \to n_{{CH_{4} }} CH_{4} \\ & + n_{{C_{2} H_{6} }} C_{2} H_{6} + n_{{C_{3} H_{8} }} C_{3} H_{8} + n_{{O_{2} }} O_{2} + n_{{N_{2} }} N_{2} \\ & + n_{{CO_{2} }} CO_{2} + n_{{co}} co + n_{{N_{{2O}} }} N_{2} O + n_{{H_{{2O}} }} H_{2} O \\ \end{aligned}$$

**Fig. 15:**
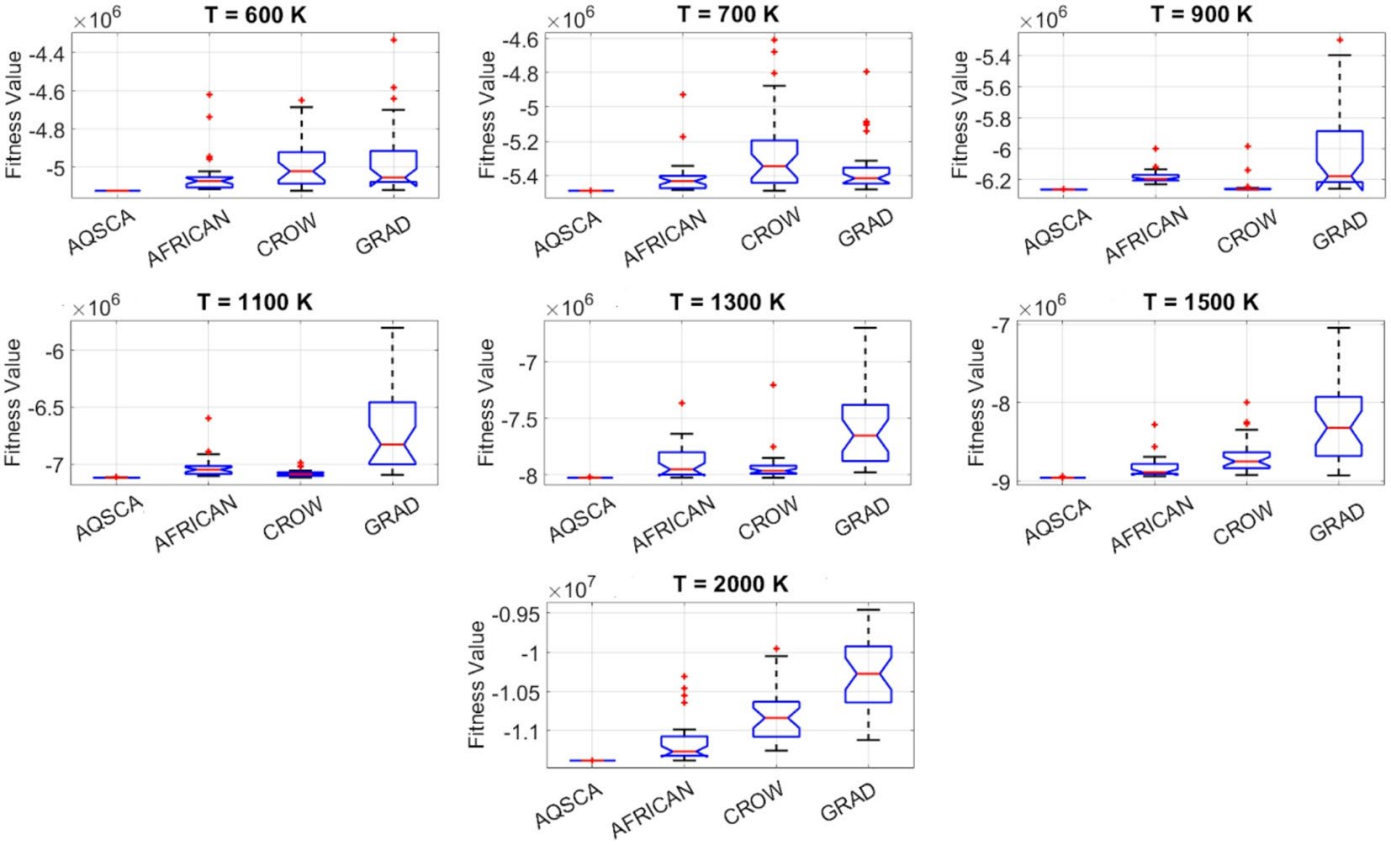
Statistical comparison of the fitness values in terms of box-plot representation for the first case study.

**Fig. 16:**
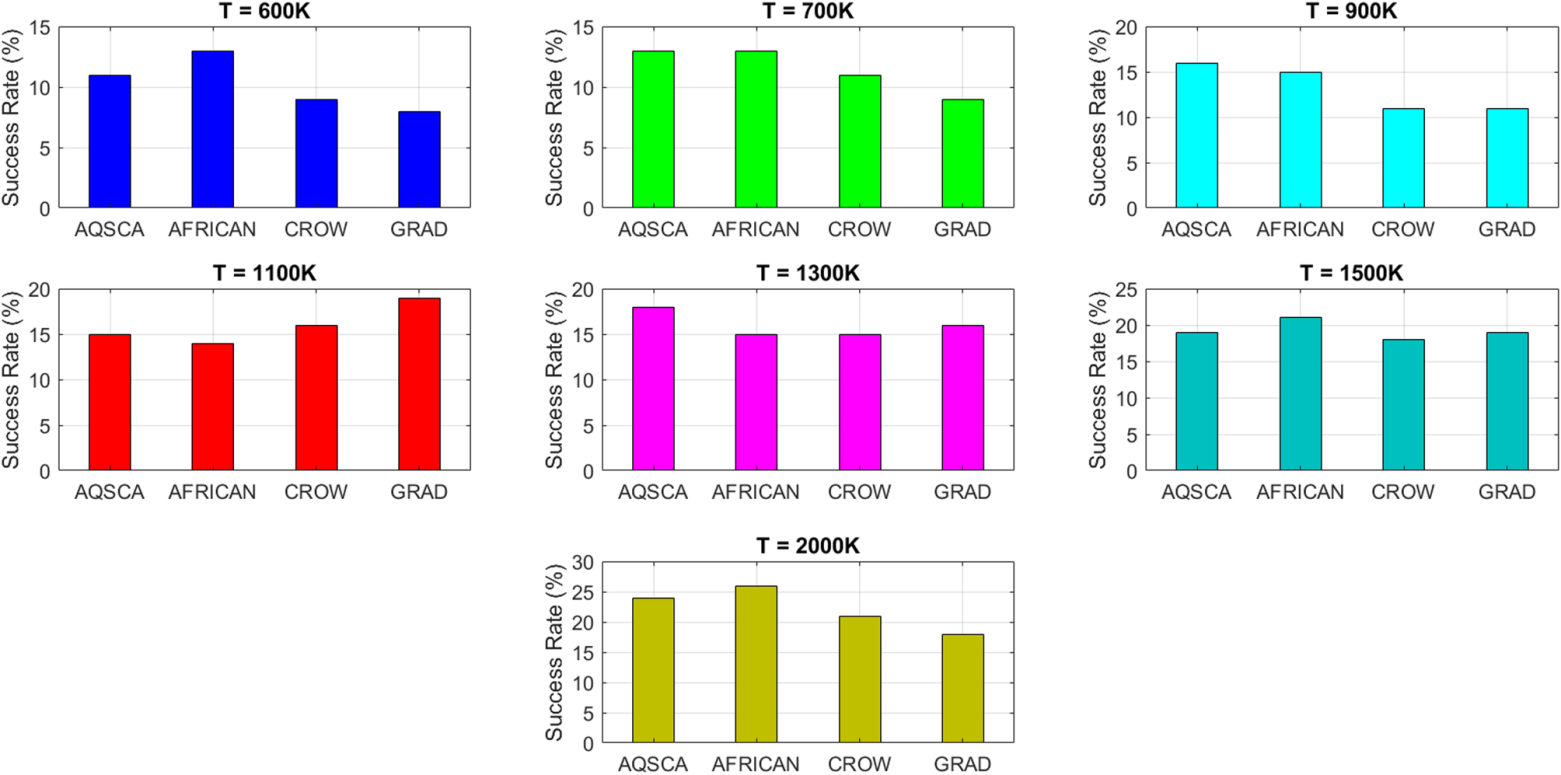
Success rates of the compared algorithms for different optimization cases.

**Fig. 17:**
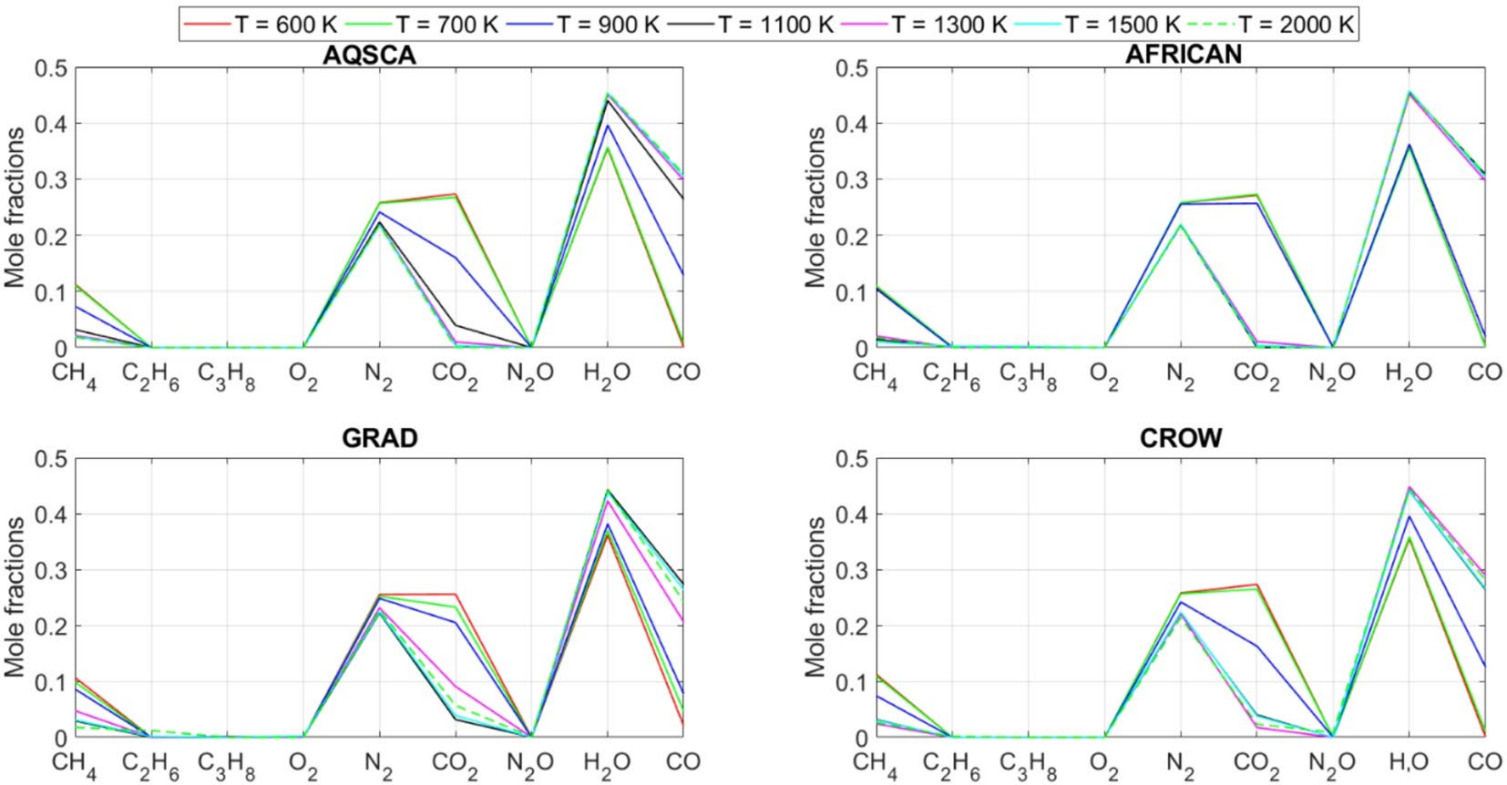
Variational changes of the mixture compositions for varying chemical system temperatures when the mixture pressure is kept constant at 1 bar.

**Fig. 18:**
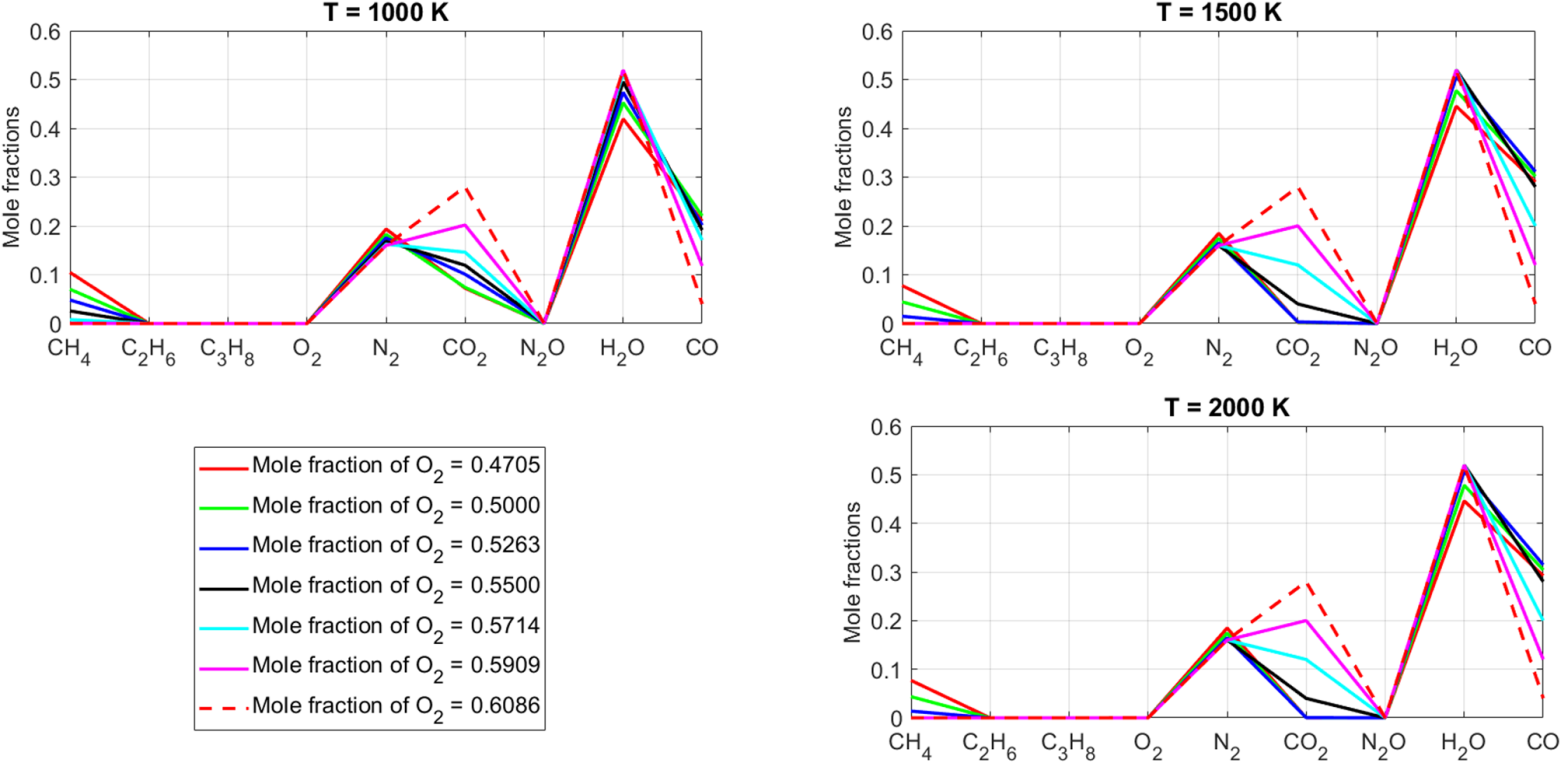
Influences of the varying mole fractions of the oxidants on the depletion rates of the fuels in the gas mixture.

**Fig. 19:**
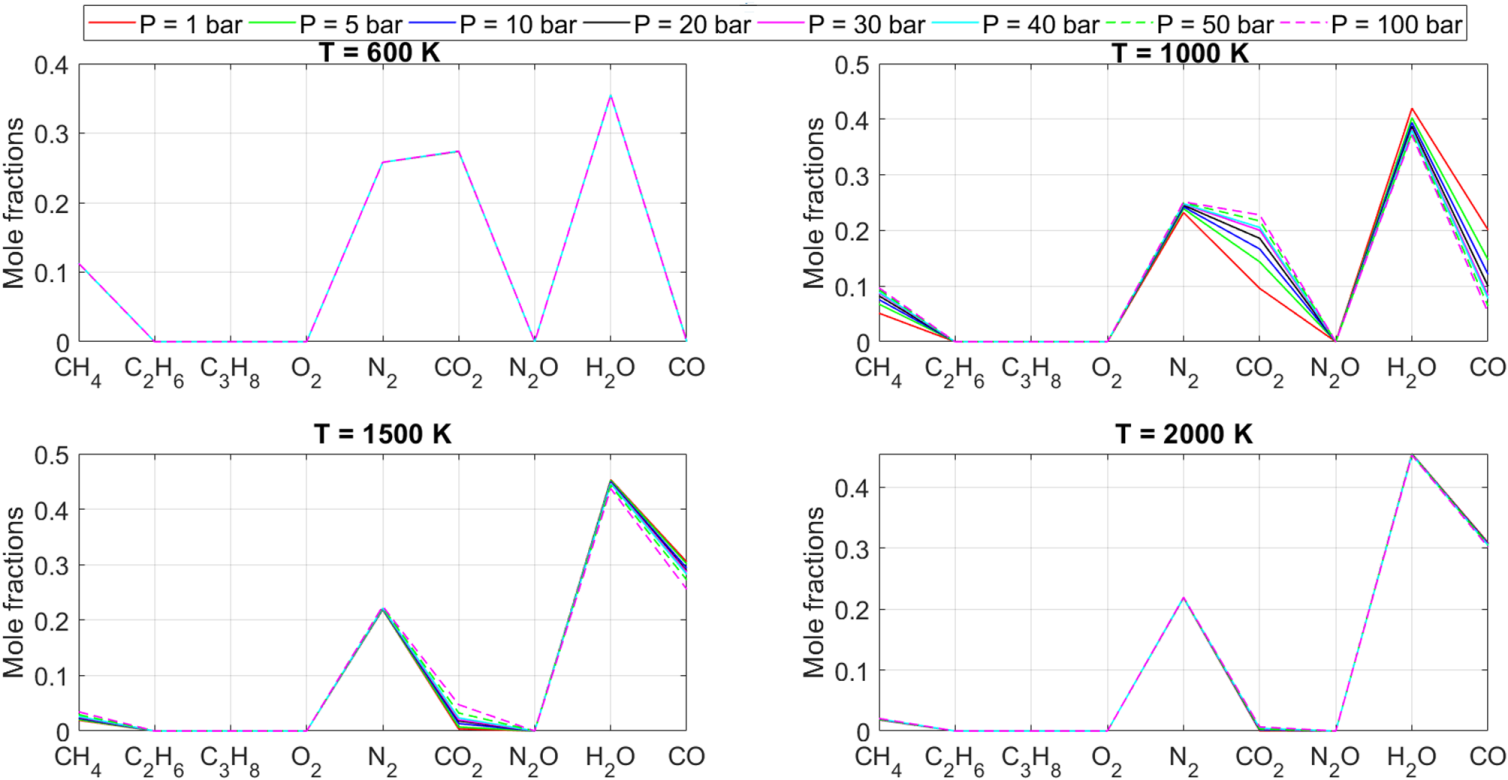
Variational compositions of the reacting components at the equilibrium point for different working pressures ranging from 1 bar to 100 bar.

The primary aim of this case is to determine the minimum value of the defined GFE minimization function using the employed metaheuristic algorithms, which provide the optimum equilibrium composition of the reacting components in the gas mixture. Table 26 reports the optimum values of the reacting species in the gas mixture, based on the minimum GFE function value, for varying working temperatures with the operation pressure fixed at 1 bar. Among the competing algorithms, which extend twenty-five different metaheuristic optimizers, only GRAD, CROW, AFRICAN, and the proposed AQSCA provide feasible solutions after 1000 consecutive iterations. When the reaction takes place at T = 600 K, T = 700 K, and T = 900 K, AQSCA and CROW obtain the same minimum objective function value, given in the respective order of *G*_*min*_ = −5.1233E + 06, *G*_*min*_ = −5.4892E + 06, *G*_*min*_ = −6.2647E + 06. In the remaining cases of working temperatures, AQSCA provides the most accurate predictions. Table 27 gives the best predictions of the four competing algorithms obtained after sequential algorithm runs for different working temperatures. The superiority of the AQSCA is evident when examining Table 27, as it provides the minimum fitness values in each optimization case. Statistical comparison between the competitive algorithms for this case is analyzed in terms of box plots in Fig. 15. Solution consistency and accuracy acquired by the proposed AQSCA are much better than the compared algorithms, as it obtains the same fitness value in each algorithm for different operational temperatures and becomes indisputably the dominant optimizer among them. Compared algorithms ' SR are shown in bar plot form for different optimization cases in Fig. 16. At relatively lower working temperatures, the SR of the algorithms could be more satisfactory and stay lower under 20% due to the deficiencies of the developed thermodynamic model, making it harder to satisfy atom and mole balance constraints. With increasing system temperatures, corresponding SR values increase yet remain within the 20%-25% level band. This outcome also proves the challenging nature of the defined objective function, which is highly nonlinear and involves restrictive non-convexities. Figure 17 visualizes the equilibrium compositions of the reacting species with varying system temperatures for different optimization methods. It is interesting to observe the stability of N_2_ molecules in the gas mixture, and no variation in the respective mole number values of N_2_ is noticed, even at increasing working temperatures, which results in the non-formation of the N_2_O compound. It is also seen that all available C_2_H_6_ and C_3_H_8_ are used out and converted into CO_2_ and H_2_O, whose respective mole fractions in the gas mixture are varied when working temperature increased to T = 600 K to T = 2000 K. Decomposition of CO_2_ into CO is evident at increasing temperatures, which also entails an increase in the formation of H_2_O vapor in the mixture. Another interesting point is the formation of CH_4_ while C_2_H_6_ and C_3_H_8_ are depleted in the mixture. However, increasing temperatures decrease CH_4_ production through the depletion of propane and ethane. Incomplete combustion is evident, particularly at lower temperatures, as the gas mixture has a significant amount of fuel (methane). At the same time, available oxygen reacts with the remaining organic compounds of ethane and propane and is depleted. Figure 18 shows the variations of the equilibrium mole fraction of the reacting mixtures with increasing oxidant mole numbers. As expected, complete combustion is achieved when the mole fraction of O_2_ increases from 0.4705 to 0.6086 in the gas mixture, resulting in a considerable reduction in the CH_4_ yield in the products. Figure 19 visually analyzes the influences of working pressure on the distribution of chemical products in the gas mixture for different reaction temperatures. At lower and higher system temperatures (600 K and 2000 K), no significant effect of operation pressures is observed over the overall mole composition. However, when the temperature is around 1000 K, the equilibrium pressure increases with the final composition as the system pressure rises, while the amount of available CO in the mixture decreases. This chemical tendency can be attributed to increasing system pressures, which favor the molecular interaction between CO and O_2_ in the mixture, leading to the formation of CO_2_.

**Table 26:** Optimum values of the reactive mixture components for varying operation temperatures.

	AQUILA-CROW	AQUILA-CROW	AQUILA-CROW	AQUILA-CROW
	T = 600 K	T = 700 K	T = 900 K	T = 1100 K
n_CH4_	1.74740306	1.72124297	1.21734205	0.56942084
n_C2H6_	0.00003111	0.00003227	0.00005237	0.00000283
n_C3H8_	0.00000091	0.00000127	0.00000486	0.00000126
n_O2_	0.00000000	0.00000138	0.00000268	0.00000375
n_N2_	3.99999999	3.99997551	3.99999528	3.99999843
n_CO2_	4.24237601	4.16404486	2.65235805	0.70827948
n_N2O_	0.00000000	0.00000902	0.00000081	0.00000117
n_H2O_	5.50509632	5.55741082	6.56513881	7.86114459
n_CO_	0.01015106	0.11447831	2.13013389	4.72228761
G_min_	−5.1233E + 06	−5.4892E + 06	−6.2647E + 06	−7.1193E + 06

	AQSCA	AQSCA	AQSCA
	T = 1300 K	T = 1500 K	T = 2000 K
n_CH4_	0.39252513	0.35565051	0.33542494
n_C2H6_	0.00003262	0.00002225	0.00003675
n_C3H8_	0.00000038	0.00000664	0.00000406
n_O2_	0.00000001	0.00000264	0.00000255
n_N2_	3.99999702	3.99999392	3.99999724
n_CO2_	0.17774038	0.06711626	0.00649503
n_N2O_	0.00000272	0.00000161	0.00000218
n_H2O_	8.21485028	8.28860483	8.32902321
n_CO_	5.42966573	5.57715342	5.65797892
G_min_	−8.0253E + 06	−8.9593E + 06	−1.1382E + 07

**Table 27:** Best fitness values obtained from different algorithms for varying working temperatures.

	AQSCA	AFRICAN	CROW	GRAD
T = 600 K	−5.1233E + 06	−5.1179E + 06	−5.1233E + 06	−5.1196E + 06
T = 700 K	−5.4892E + 06	−5.4870E + 06	−5.4892E + 06	−5.4851E + 06
T = 900 K	−6.2647E + 06	−6.2512E + 06	−6.2647E + 06	−6.2601E + 06
T = 1100 K	−7.1193E + 06	−7.1029E + 06	−7.1179E + 06	−7.1150E + 06
T = 1300 K	−8.0253E + 06	−8.0227E + 06	−8.0248E + 06	−7.9937E + 06
T = 1500 K	−8.9593E + 06	−8.9409E + 06	−8.9429E + 06	−8.9270E + 06
T = 2000 K	−1.1382E + 07	−1.1380E + 07	−1.1294E + 07	−1.1266E + 07

### Combustion of a natural gas mixture

**Fig. 20:**
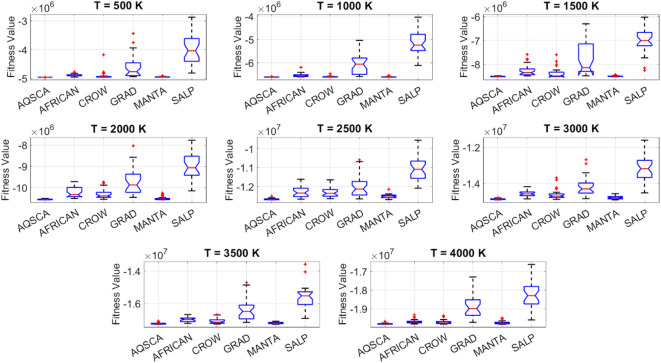
Statistical comparison of the predictive results in terms of box plot representation.

**Fig. 21:**
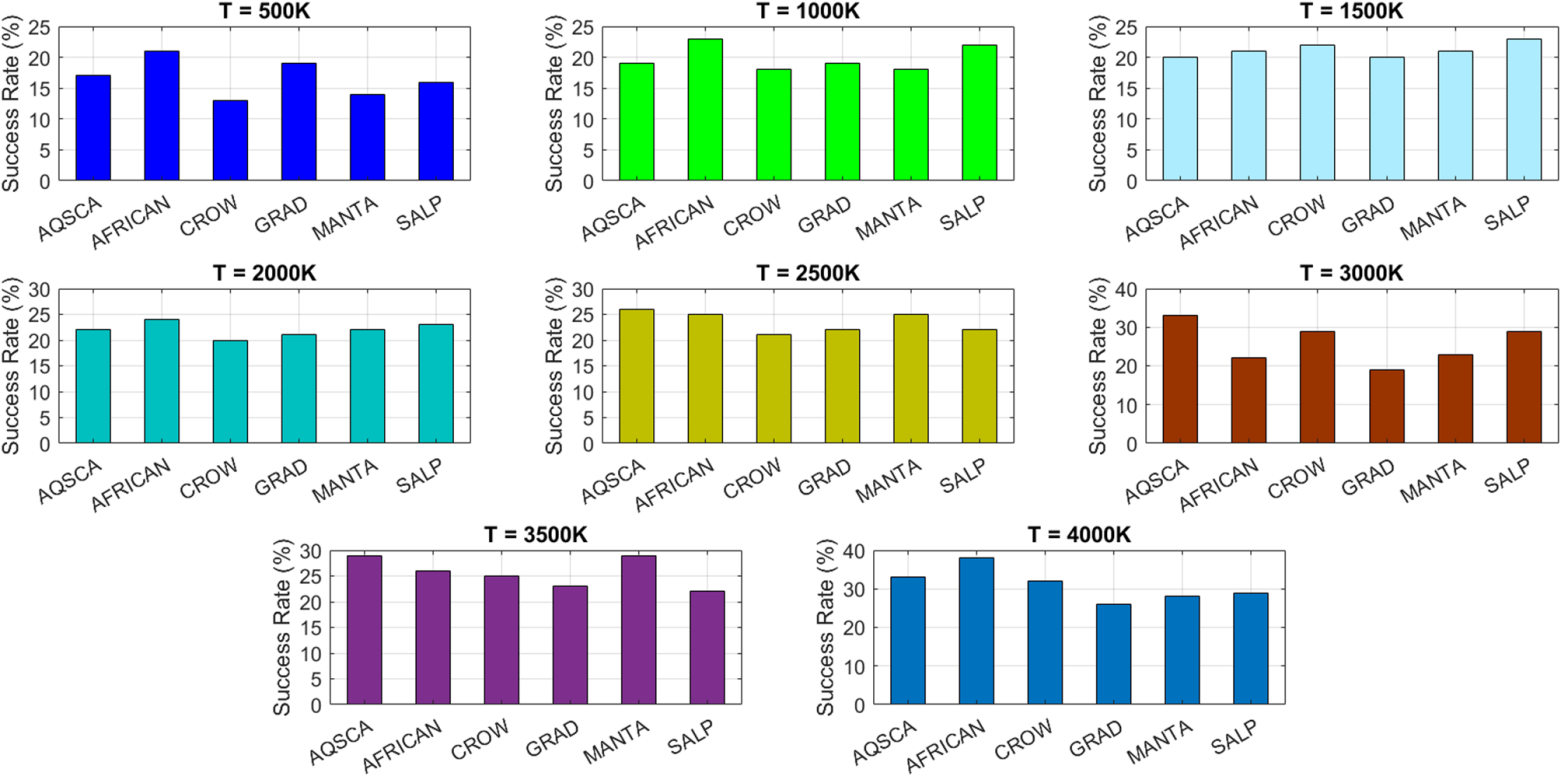
Success rates of the compared algorithms for different reaction temperatures.

**Fig. 22:**
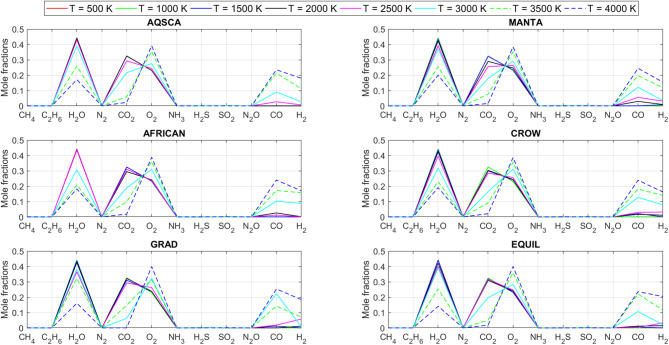
Influences of the system temperatures on the distribution of the mole fractions of the reacting components at the equilibrium point when the system pressure is fixed to 1 bar.

**Fig. 23:**
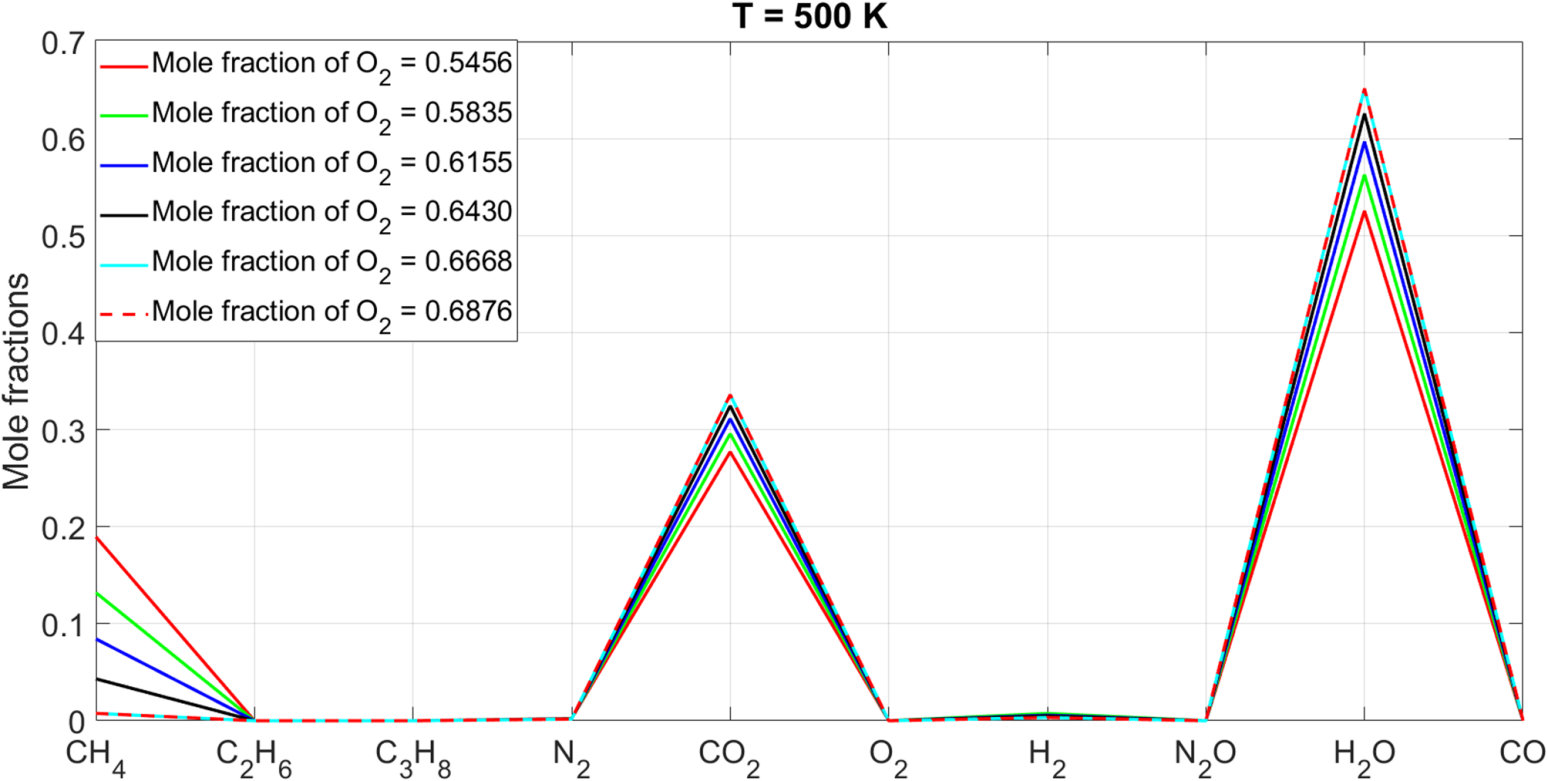
Variation of mole fraction at extra oxygen in the gas mixture.

**Fig. 24:**
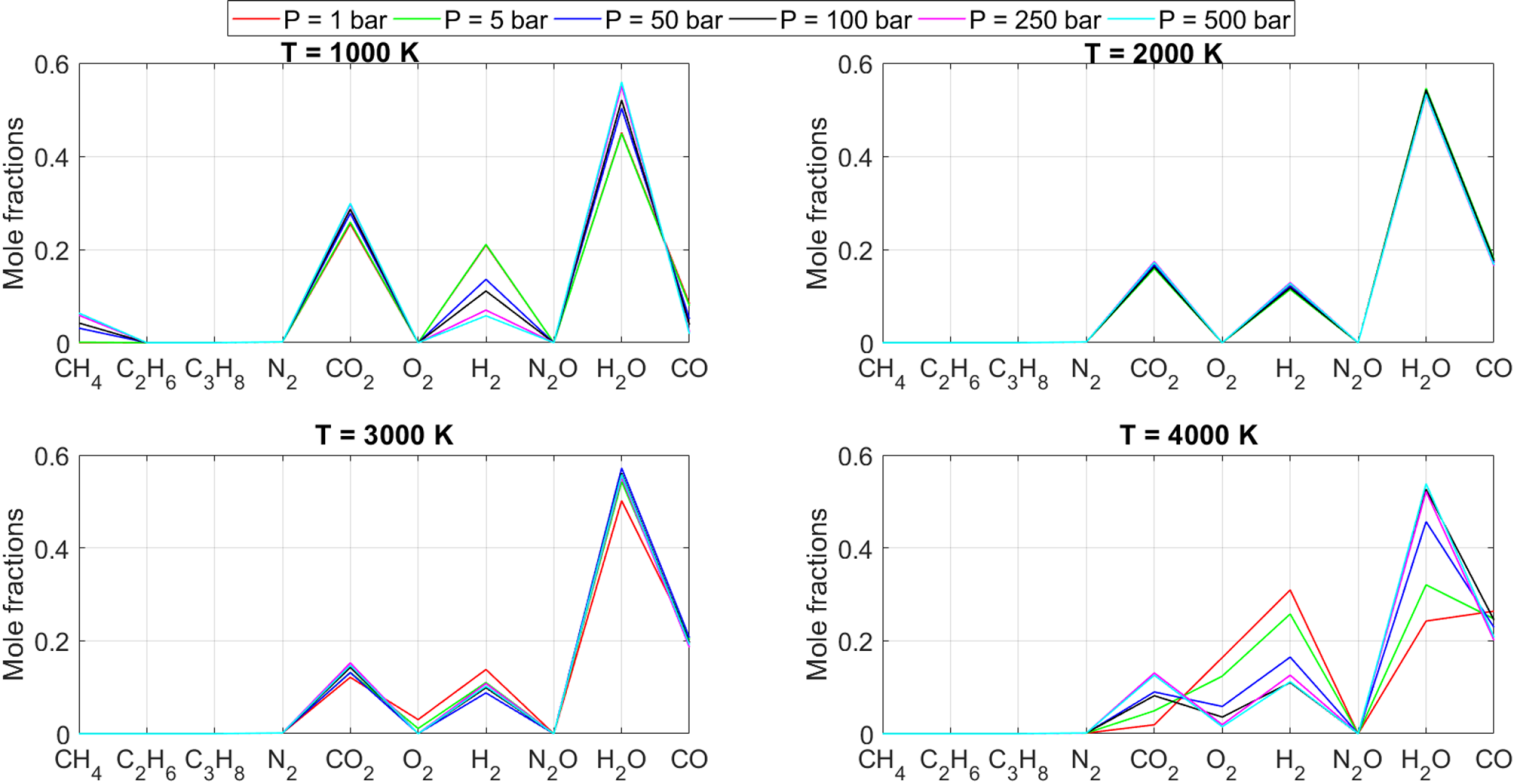
Variations of the equilibrium compositions of the gas mixture components with increasing system pressures.

Consider a mixture whose system components involve the forming species of natural gas. Table 28 reports the initial mole number of the reactants constituting the natural gas mixture before entering the chemical reactions. This section will analyze the chemical behaviors of the reacting components in the heterogeneous gas mixture, considering variations in system temperatures from 500 K to 4000 K and system pressures from 1 bar to 500 bar. The best-performing algorithms for GFE minimization to obtain the equilibrium mole distribution of the interacting species are AQSCA, AFRICAN, CROW, GRAD, MANTA, and SALP optimizers. Table 29 presents the optimal solutions from the compared algorithms for various reaction temperatures, with the system pressure maintained at 1 bar. When the reaction occurs at 500 K, the AQSCA and CROW algorithms converge to the same global minimum, G_min_ = −4.9565E + 06, which yields the most accurate distribution of the equilibrium mole numbers of the reactive species. AQSCA is the best-performing algorithm among the competing algorithms for the remaining operational temperature cases. Table 30 presents the best predictions of the objective function values obtained by the six aforementioned algorithms for varying reaction temperatures. AQSCA achieves the minimum GFE function values for each case, surpassing the remaining algorithms in terms of solution accuracy. Box plots, visualized in Fig. 20, comparatively investigate the statistical performances of the six metaheuristic algorithms employed. AQSCA yields very close estimations in each algorithm run for varying system temperatures and outperforms the competing algorithm in terms of solution persistence. MANTA is the second-best performer among competitors in Table 30, consistently producing similar prediction results across successive runs, which reassures its solution consistency in challenging Gibbs Free energy minimization problems. Figure 21 compares the success rates of the algorithms for different operational reaction temperatures. Like the previous case, the success rates of the algorithms increase with increasing system temperatures. Figure 22 illustrates the variational effects of reaction temperatures on the mixture compositions of the reacting species, with the system pressure held constant at 1 bar. It is worth noting that composition curves are plotted for the optimal solution of the respective algorithm for the corresponding system temperature. Again, similar to the previous case, C_2_H_6_ and C_3_H_8_ are already depleted, while a significant amount of CH_4_ remains in the mixture, even at lower system temperatures. It is observed that the CH_4_ compound in the gas mixture decreases with increasing temperatures, which also results in a decline in mole fraction rates of CO_2_ and H_2_O. The mole fraction of N_2_ remains unchanged with rising temperatures. Therefore, no recognizable formation of N_2_O is identified. Depleting hydrocarbon fuels, including CH_4_, C_2_H_6_, and C_3_H_8_, are transformed into CO and H_2_ as the system temperature is increased from 500 K to 400 K. This type of conversion facilitates the production of synthetic gases, which are combustible and can be reutilized as a fuel. Incomplete combustion is also evident in this case, which can be mitigated by adding extra oxygen to the gas mixture, as shown in Fig. 23. Figure 24 illustrates the effects of increasing system pressures on the mole fraction distribution of the reacting molecules. When the system temperature is 1000 K, increasing system pressures from 1 to 100 bars significantly impacts hydrogen production, as lower system pressures lead to better H_2_ formation. However, increasing reaction pressures gives rise to the production of CO_2_ and H_2_O. There is no significant effect of increasing pressure on the change in mole fractions when the system pressure is T = 2000 K. A completely different reaction scenario is encountered when the system temperature is equal to or above T = 3000 K, as hydrogen production is more efficient at lower system pressures. This tendency is more pronounced at T = 4000 K, as the formation of CO_2_ and H_2_O increases significantly at higher system pressures; furthermore, a minor amount of O_2_ formation is also observed at elevated pressures.

**Table 28:** Initial mole numbers of the reactants.

Component	Feed (mole)
CH_4_	4.735
C_2_H_6_	0.210
C_3_H_8_	0.010
N_2_	0.025
CO_2_	0.015
O_2_	8.000
H_2_	0.001
N_2_O	0.000
H_2_O	0.000
CO	0.000

**Table 29:** Variations of the equilibrium mixture components with increasing system temperatures.

	AQSCA-CROW	AQSCA	AQSCA	AQSCA
	T = 500 K	T = 1000 K	T = 1500 K	T = 2000 K
*n*_*CH4*_	1.10968134	0.00178466	0.00001973	0.00006142
*n*_*C2H6*_	0.00012757	0.00001303	0.00001652	0.00002849
*n*_*C3H8*_	0.00000671	0.00000943	0.00000216	0.00006102
*n*_*N2*_	0.02499564	0.02497578	0.02499988	0.02433953
*n*_*CO2*_	4.08919421	3.91204439	2.97075622	2.56398099
*n*_*O2*_	0.00000183	0.00000657	0.00001558	0.00068855
*n*_*H2*_	0.07015677	3.21758743	2.28159456	1.87612823
*n*_*N2O*_	0.00000106	0.00001861	0.00000000	0.00050318
*n*_*H2O*_	7.85105225	6.91976445	7.85927639	8.26439201
*n*_*CO*_	0.00055333	1.28610663	2.22913542	2.63557389
*G*_*min*_	−4.9565E + 06	−6.6162E + 06	−8.5288E + 06	−1.0566E + 07

	AQSCA	AQSCA	AQSCA	AQSCA
	T = 2500 K	T = 3000 K	T = 3500 K	T = 4000 K
*n*_*CH4*_	0.00178072	0.00263519	0.00008497	0.00003847
*n*_*C2H6*_	0.00046443	0.00000383	0.00005260	0.00002173
*n*_*C3H8*_	0.00009455	0.00005816	0.00019412	0.00001356
*n*_*N2*_	0.02327018	0.02481673	0.02031808	0.02477145
*n*_*CO2*_	2.32155717	1.92318371	0.89044671	0.35255832
*n*_*O2*_	0.00135576	0.48129687	1.83129349	3.01082356
*n*_*H2*_	1.62804145	2.18862364	3.86580677	5.68496571
*n*_*N2O*_	0.00157934	0.00016998	0.00456709	0.00007540
*n*_*H2O*_	8.50733052	7.94681942	6.27399705	4.45582100
*n*_*CO*_	2.87524321	3.27399014	4.30792064	4.84722671
*G*_*min*_	1.2698E + 07	−1.4926E + 07	−1.7299E + 07	−1.9832E + 07

**Table 30:** Comparison of the respective prediction accuracies of the algorithms for varying reaction temperatures.

	AQSCA	AFRICAN	CROW	GRAD	MANTA	SALP
T = 500 K	−4.9565E + 06	−4.9537E + 06	−4.9565E + 06	−4.9410E + 06	−4.9563E + 06	−4.8119E + 06
T = 1000 K	−6.6162E + 06	−6.6024E + 06	−6.6153E + 06	−6.5952E + 06	−6.6160E + 06	−6.1138E + 06
T = 1500 K	−8.5288E + 06	−8.4930E + 06	−8.5262E + 06	−8.4860E + 06	−8.5279E + 06	−8.2591E + 06
T = 2000 K	−1.0566E + 07	−1.0502E + 06	−1.0543E + 06	−1.0499E + 06	−1.0564E + 07	−1.0227E + 06
T = 2500 K	−1.2698E + 07	−1.2665E + 07	−1.2664E + 07	−1.2671E + 07	−1.2692E + 07	−1.2234E + 07
T = 3000 K	−1.4926E + 07	−1.4867E + 07	−1.4900E + 07	−1.4857E + 07	−1.4924E + 07	−1.4537E + 07
T = 3500 K	−1.7299E + 07	−1.7252E + 07	−1.7295E + 07	−1.7229E + 07	−1.7292E + 07	−1.6926E + 07
T = 4000 K	−1.9832E + 07	−1.9811E + 07	−1.9819E + 07	−1.9779E + 07	−1.9829E + 07	−1.9595E + 07

### Combustion of a fuel gas mixture

**Fig. 25:**
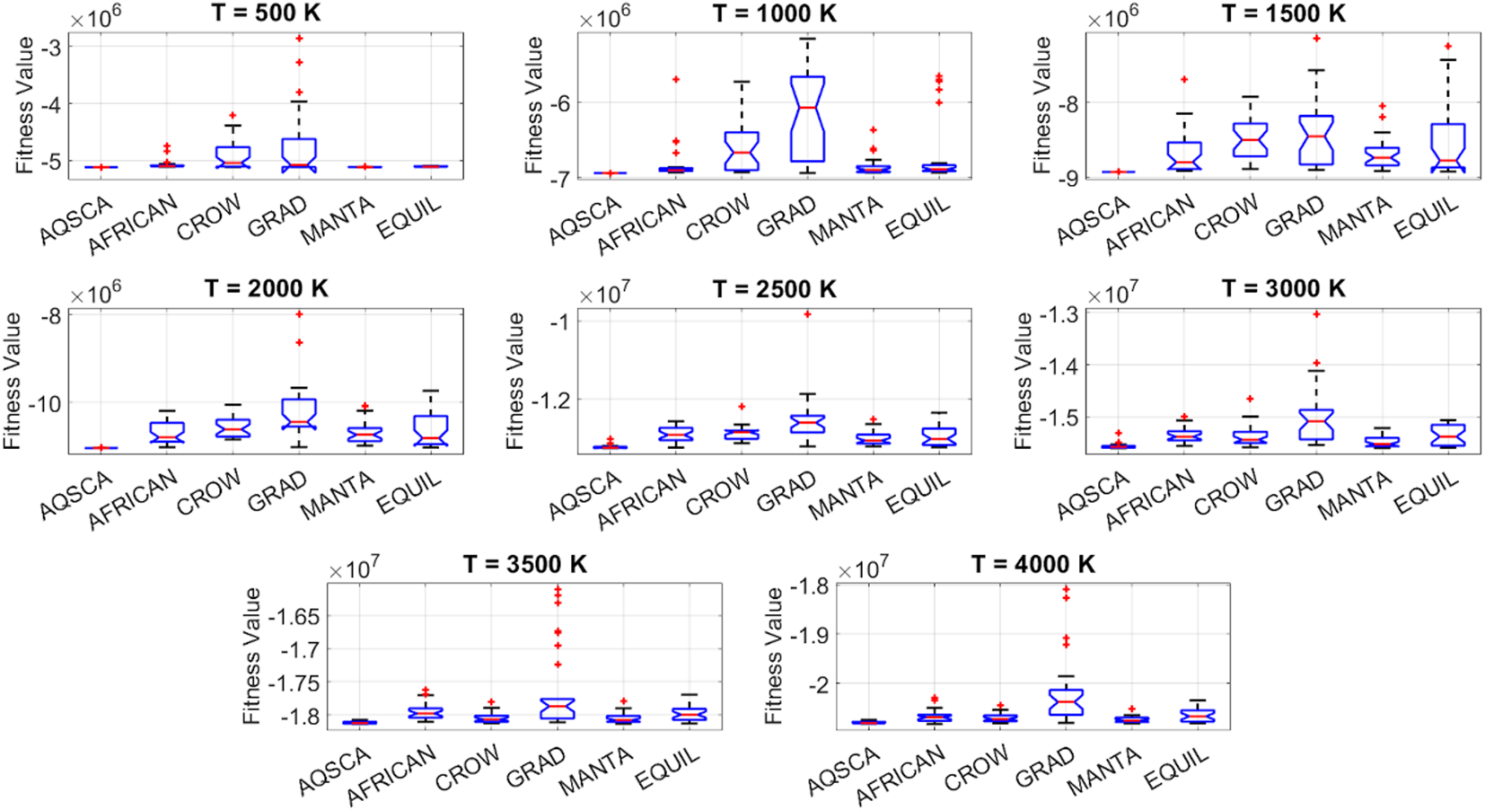
Statistical comparison between the competing algorithms for the third case study.

**Fig. 26:**
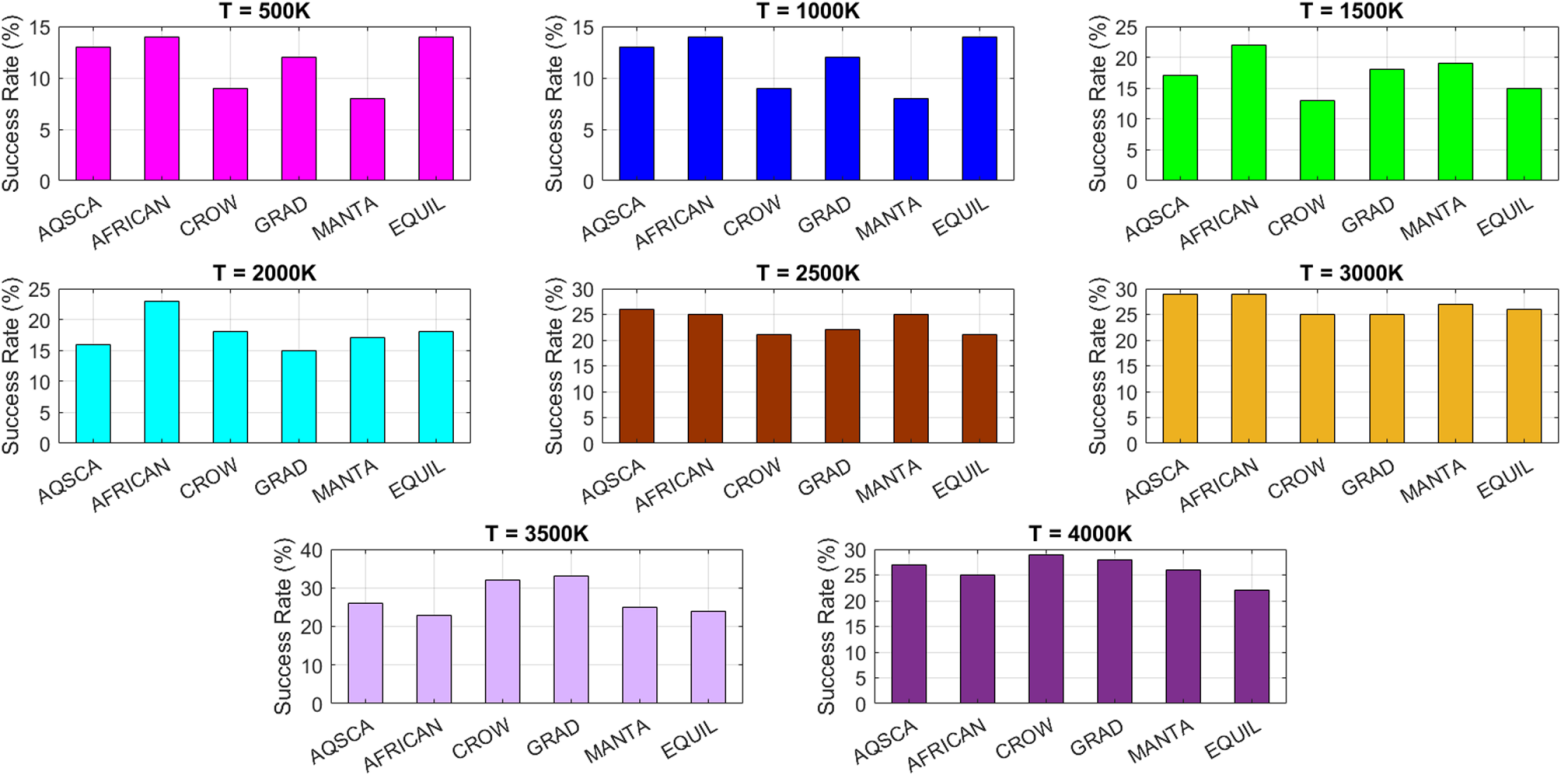
Respective success rates of the algorithms for different operation temperatures.

**Fig. 27:**
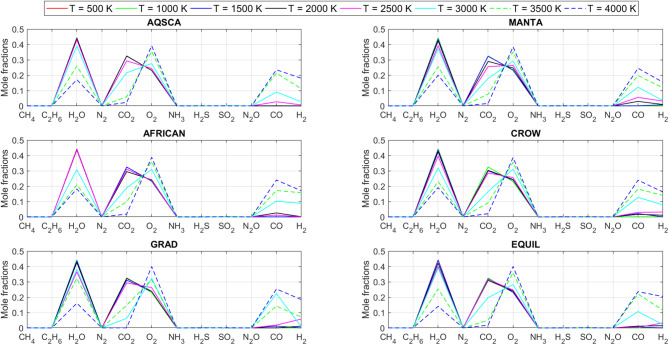
Variation of the equilibrium mole fractions with increasing system temperature for the compared algorithms.

**Fig. 28:**
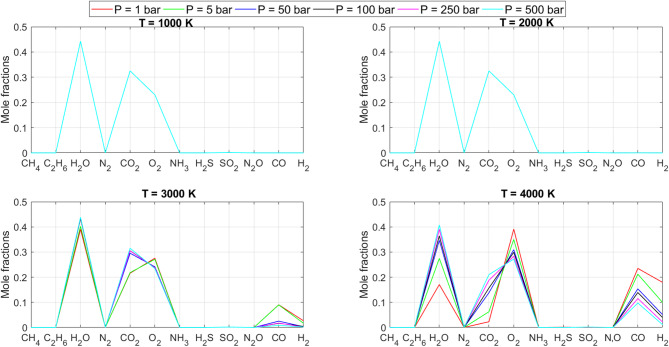
Influences of the operation pressures on the mole distribution of the reacting components at the equilibrium point in the gas temperature.

Assume a gas mixture whose initial mole composition is given in Table 31, involving chemical species of CH_4_, C_2_H_6_, H_2_O, N_2_, CO_2_, O_2_, NH_3_, H_2_S, SO_2_, N_2_O, CO, and H_2_ to be reacted at varying system temperatures and pressures. Table 32 presents the equilibrium mole compositions of the reacting mixture components at various reaction temperatures, with the system pressure maintained at 1 bar. The best-performing optimizers among AQSCA, AFRICAN, CROW, GRAD, MANTA, and EQUIL determined these values. AQSCA and MANTA find, respectively, the same minimum GFE value of G_min_ = −5.1179E + 06, G_min_ = −1.5603E + 07, and G_min_ = −1.8134E + 07 when reaction temperatures are T = 500 K, T = 3000 K, and T = 3500 K. AFRICAN obtains the lowest objective function value of G_min_ = −2.0825E + 07 for the reaction temperature of T = 4000 K. Table 33 gives a more detailed comparative analysis between the competing algorithms in terms of best obtained prediction results for varying system temperatures. Statistical comparison of the fitness values retained after successive algorithm runs are evaluated and schematically analyzed in box plots in Fig. 25. AQSCA reaches the exact optimal solution with each algorithm run when reaction temperatures are T = 500 K, T = 1000 K, T = 1500 K, and T = 2000 K, and slight deviations from the best solution are observed for the remaining cases, which assures the effectivity of the proposed hybrid AQSCA algorithm over solving highly nonlinear chemical equilibrium problems, thanks to its superior solution quality and consistency. However, as can be seen in Fig. 26, the contestant optimizers outperform the proposed method in most cases in terms of success rates. Figure 27 shows the distribution of mole compositions of the reacting species with increasing reaction temperatures, where the system pressure is constant at 1 bar. At lower system temperatures, vapor deposition of H_2_O and CO_2_ is evident in the mixture, while the available O_2_ in the mix increases at higher temperatures. Syngas production is significantly higher at higher working temperatures due to the decomposition of H_2_O and CO_2_ into CO and H_2_, the system components. The system’s hydrogen sulfide (H_2_S) reacts with the excess O_2_. It forms SO_2_, one of the main reasons for the increase in H_2_ production in the system, along with the contribution of methane and ethane oxidation, which yields excessive hydrogen, particularly at elevated reaction temperatures. In addition, no significant conversion between NH_3_ and N_2_O is observed in the gas mixture, particularly at lower system temperatures. It can also be concluded from the distribution of the reacting species’ composition that the final state of the products can be evaluated as a “lean mixture” since there is still burnable oxygen. In contrast, available fuel compounds in the gas mixture are depleted. Figure 28 illustrates the effects of operating pressures on the mole distributions of the reacting components at equilibrium points for varying system temperatures. It is observed that the variations in system pressures significantly impact the mole distribution of the mixture components at higher reaction temperatures.

**Table 31:** Initial composition of the gas mixture.

Component	Feed (mole)
CH_4_	3.250
C_2_H_6_	0.005
H_2_O	0.075
N_2_	0.015
CO_2_	1.600
O_2_	10.000
NH_3_	0.0025
H_2_S	0.025
SO_2_	0.000
N_2_O	0.000
CO	0.000
H_2_	0.000

**Table 32:** Equilibrium mole compositions in the products for varying reaction temperatures.

	AQSCA-MANTA	AQSCA	AQSCA	AQSCA
	T = 500 K	T = 1000 K	T = 1500 K	T = 2000 K
*n*_*CH4*_	0.00000000	0.00000000	0.00000061	0.00000189
*n*_*C2H6*_	0.00000000	0.00000000	0.00000042	0.00001066
*n*_*H2O*_	6.61874997	6.61874963	6.61853011	6.61520866
*n*_*N2*_	0.00374992	0.00374992	0.00372259	0.00369597
*n*_*CO2*_	4.85999999	4.85999999	4.85987229	4.84858869
*n*_*O2*_	3.44312498	3.44312522	3.44328736	3.45085691
*n*_*NH3*_	0.00000000	0.00000000	0.00000173	0.00001424
*n*_*H2S*_	0.00000000	0.00000000	0.00000080	0.00027576
*n*_*SO2*_	0.02499999	0.02499999	0.02499876	0.02472278
*n*_*N2O*_	0.00000000	0.00000000	0.00002616	0.00004584
*n*_*CO*_	0.00000000	0.00000000	0.00012606	0.01138386
*n*_*H2*_	0.00000000	0.00000000	0.00021195	0.00320001
*G*_*min*_	−5.1179E + 06	−6.9371E + 06	−8.9260E + 06	−1.1037E + 07

	AQSCA	AQSCA-MANTA	AQSCA-MANTA	AFRICAN
	T = 2500 K	T = 3000 K	T = 3500 K	T = 4000 K
*n*_*CH4*_	0.00015659	0.00046617	0.00007194	0.00165839
*n*_*C2H6*_	0.00006508	0.00003022	0.00002192	0.00001386
*n*_*H2O*_	6.52159311	6.19355088	4.62168154	3.42358950
*n*_*N2*_	0.00353752	0.00132475	0.00018175	0.00109381
*n*_*CO2*_	4.45015881	3.42602217	1.05720488	0.35006298
*n*_*O2*_	3.69715605	4.37277655	6.34917939	7.29590043
*n*_*NH3*_	0.00008642	0.00291065	0.00002055	0.00031721
*n*_*H2S*_	0.00034741	0.00021643	0.00769017	0.00002127
*n*_*SO2*_	0.02460346	0.02468613	0.01720961	0.02440928
*n*_*N2O*_	0.00008981	0.00090142	0.00345101	0.00241481
*n*_*CO*_	0.40943096	1.43338807	3.80267299	4.50825014
*n*_*H2*_	0.09584121	0.41958514	1.98908637	3.19130501
*G*_*min*_	−1.3253E + 07	−1.5603E + 07	−1.8134E + 07	−2.0825E + 07

**Table 33:** Best fitness values for different system temperatures.

	AQSCA	AFRICAN	CROW	GRAD	MANTA	EQUIL
T = 500 K	−5.1179E + 06	−5.1139E + 06	−5.1176E + 06	−5.1176E + 06	−5.1179E + 06	−5.1175E + 06
T = 1000 K	−6.9371E + 06	−6.9289E + 06	−6.9323E + 06	−6.9364E + 06	−6.9333E + 06	−6.9328E + 06
T = 1500 K	−8.9260E + 06	−8.9090E + 06	−8.8835E + 06	−8.8983E + 06	−8.9132E + 06	−8.9175E + 06
T = 2000 K	−1.1037E + 07	−1.1016E + 07	−1.1012E + 07	−1.1015E + 07	−1.1005E + 07	−1.1019E + 07
T = 2500 K	−1.3253E + 07	−1.3237E + 07	−1.3236E + 07	−1.3211E + 07	−1.3235E + 07	−1.3231E + 07
T = 3000 K	−1.5603E + 07	−1.5562E + 07	−1.5587E + 07	−1.5540E + 07	−1.5603E + 07	−1.5596E + 07
T = 3500 K	−1.8134E + 07	−1.8102E + 07	−1.8123E + 07	−1.8109E + 07	−1.8134E + 07	−1.8129E + 07
T = 4000 K	−2.0823E + 07	−2.0825E + 07	−2.0824E + 07	−2.0805E + 07	−2.0822E + 07	−2.0815E + 07

## Discussion over the algorithmic structure of the proposed method and optimization results

This research study proposes contributive novelties to the current literature regarding the design and application of metaheuristic algorithms for solving chemical equilibrium problems. A novel learning scheme is developed and integrated into the standard AQUILA algorithm, significantly enhancing the probing performance of the responsible agents roaming within the search domain. This improvement occurred in the synergy between the random numbers generated by the Levy flight distribution, pseudo-random numbers generated by the Ikeda chaotic map, and iteratively adjusted weight parameters developed from different mathematical foundations. Random numbers generated by the Levy flight mechanism enable the algorithm to make both small and long jumps across the solution domain, which helps the algorithm discover previously unexplored regions and improve solution diversity. This behavior also entails reaching faster convergence with higher accuracy in some cases. In addition, Ikeda chaotic map-based random numbers elevate the algorithm to higher degrees, achieving a superior quality of randomness thanks to the unpredictable and non-repetitive characteristics of chaotic random numbers. The controlled randomness of the Ikeda map balances the exploration and exploitation mechanisms, whose favorable interaction yields a diverse set of solutions and avoids premature convergence to sub-optimal solutions. Using different characteristics of dynamic inertia weight parameters is another factor that improves diversity in the evolving population, as its proper utilization controls the trade-off between the exploration and exploitation mechanisms of the running algorithm. In this context, higher inertia weight parameter values promote global exploration, and lower inertia weight activates the local exploitation mechanism of the algorithm. It is also observed that iteratively adjusted (decreased) weight values appropriately administer the search direction and maintain efficient probing over the search space. In addition, using sine and cosine operators, augmented by the contributions of chaotic random numbers in metaheuristics, improves the inherent randomness. This is due to the periodic and nonlinear behaviors of these trigonometric functions, which oscillate between − 1 and 1 during the iterative process. Large oscillations promote the activation of the global search mechanism, while small oscillations favor the activation of local search. Random numbers generated by sine and cosine functions produce a wave-like search pattern, another beneficial characteristic that enhances the overall diversity of the population. This is why the proposed AQSCA can effectively eliminate the local pitfalls over the high-dimensional solution domain and progressively approach the global optimum solution. The proposed search scheme possesses an intrinsic capability to rectify the imbalance between exploration and exploitation mechanisms, resulting in a swift yet accurate progression toward the optimal solution of the problem. Although this may not be the primary concern of this research study, comparative analysis also reveals that the constraint-handling method proposed by Kim et al.^100^ outperforms existing penalty-based-handling strategies in the current literature. This success in finding feasible solutions for constrained problems with varying functional complexities stems from the structural formation of the developed model, which does not rely on trial-and-error-based adjustable penalty parameters to eliminate infeasible regions over the solution domain.

Applying metaheuristic algorithms to solving chemical equilibrium problems is another innovative novelty proposed in this study. Retaining the accurate number of products in the chemical reaction for specified operational conditions through metaheuristic algorithms has many advantages over traditional analytical-based optimization algorithms. Characteristically, chemical equilibrium problems are highly nonlinear constrained engineering cases for which many conventional derivative-based and deterministic algorithms often fail due to their inherent limitations in handling these complex challenges. First and foremost, metaheuristic algorithms do not require derivative information from the search domain, rendering them adept and favorable approaches for discontinuous and non-differentiable functions, such as activity functions and fugacity models, which are the main components of chemical reaction models. Chemical equilibrium models require dealing with restrictive model constraints such as mass balance, reaction equilibrium constants, and other thermodynamic constraints, which stochastic metaheuristic algorithms can handle reliably and accurately. When the number of reacting species increases in the chemical mixture, the governing multimodality of the objective function increases dramatically, which in turn increases the number of local points in the evolving solution domain. Metaheuristic algorithms can effectively cope with these nonlinearities imposed by the increased problem dimensionalities. The scalability of the metaheuristic algorithm puts it one step ahead of other alternatives for large-scale equilibrium problems, particularly for reactive multiphase systems. Chemical equilibrium systems may involve uncertain model parameters, such as experimental errors or variations in operation temperatures or pressures. Metaheuristic algorithms can find robust and consistent solutions for equilibrium problems with relatively higher experimental noise and uncertainties. Finally, they can be easily integrated into the governing thermodynamic model of the chemical equilibrium problem without modifying the base optimization algorithm. The advantages of metaheuristic algorithms render them superior candidates for solving chemical equilibrium problems compared to traditional derivative-based optimization methods, including Newton-based optimizers and Conjugate Gradient methods.

According to the optimal results for different chemical reaction cases previously discussed in Sect. 6, the following conclusion can be drawn: working operational temperatures have a significantly greater effect on the formation and distribution of new components in the reacting mixture than operational pressures. This behavior can be attributed to the fact that operational temperatures directly affect reaction rates, providing the necessary energy input to the reacting system components, which facilitates frequent collisions of active molecules and thereby overcomes the energy barrier. The Arrhenius equation, defined below, can also verify this behavior of energized molecules^115^.

41 $$\:k=A{e}^{\raisebox{1ex}{$-{E}_{a}$}\!\left/\:\!\raisebox{-1ex}{$RT$}\right.}$$

Where k is the reaction rate constant, *E*_*a*_ is the activation energy, *R* is the universal gas constant, and *T* is the temperature in Kelvin scale. Even a slight change in temperature values can lead to exponential variations in reaction rates, influencing the distribution of reacting molecules in the mixture. The pressure does not affect the Arrhenius equation. According to the fundamentals of Le Chatelier’s principles, variations in working temperatures in the chemical mixture can cause equilibrium shifts, which may occur in either a forward or reverse direction. Endothermic reactions occur at higher temperatures, while exothermic reactions are favored at lower operational temperatures, which can substantially alter the formation of new components in the chemical mixture. Higher working temperatures may increase the likelihood of thermal decomposition, resulting in the formation of various chemical products.

It is also observed that working pressures have a lower influence on the distribution of new components in the chemical mixture. This is mainly because molecular collision frequently occurs at higher temperatures due to increased gas expansion and reduced molecular density. Higher working pressures compensate for this expansion by increasing the frequency of molecular collisions, thereby enhancing the reaction rate and product formation. Similar to the influence of temperature on product formation, higher pressures can also lead to shifts in reaction, significantly affecting the distribution of the products that form. It is also evident that new reaction pathways are accessible at higher temperatures, which are pressure-sensitive and considerably influenced by variations in operational pressure rates.

Although the hybrid algorithm’s time complexity is comparable to that of the original Aquila Optimizer, it may require a relatively longer computational time to calculate random numbers generated by the Levy flight distribution and the Ikeda chaotic map, posing the main challenge to the algorithm’s design. The proposed method encounters difficulties in capturing accurate estimations for optimal control problems, which this research study has not yet addressed. These problems have been incorporated into the algorithm development phase to test the efficiency and accuracy of the proposed method’s predictions. Despite hybridization, the AQSCA algorithm may still stagnate in deceptive landscapes, due to SCA’s tendency to be trapped in a local solution for high-dimensional spaces. AQSCA requires numerous function evaluations, resulting in a high computational cost that limits scalability for high-dimensional optimization problems. AQSCA relies on several adjustable model parameters, making it sensitive to the initial parameter settings. Population initialization of AQUILA may lack solution diversity. Although SCA’s oscillatory movements improve the general solution quality through this adaptive refinement process, the hybrid AQSCA may still suffer from low solution diversity across the population individuals, leading to being trapped in a stagnant phase in shifted or noisy problems.

### Conclusive remarks and future projections

This study proposes a novel hybrid metaheuristic algorithm, AQSCA, by combining the original Aquila Optimization with two different literature variants of SCA to solve chemical equilibrium problems, leveraging the merits of the GFE Minimization method. The primary objective of this integration is to create a synergistic interaction between these two complementary metaheuristic algorithms, thereby enhancing the balance between exploration and exploitation mechanisms, which leads to a significant improvement in the quality of the predictive results. A hundred multidimensionally artificially generated benchmark functions have been solved to assess the accuracy of the proposed methods’ estimation and optimization performance. The results have been compared to those obtained for some of the state-of-the-art cutting-edge metaheuristic methods. The developed AQSCA proves to be the best-performing optimizer in terms of solution accuracy and consistency in most cases. To further evaluate the solution efficiency of the hybrid AQSCA optimizer, 28 challenging test problems from the CEC competitions have been solved, and the retained solutions have been compared to those found by some recent optimization methods. Finally, three multi-reaction chemical equilibrium problems with varying degrees of difficulty have been applied to the AQSCA algorithm to benchmark its effectiveness on real-world constrained, complex optimization problems. The Gibbs Free Energy minimization method is used to determine the equilibrium compositions of the reacting molecules in each case, and the corresponding solutions obtained by AQSCA are compared with those obtained by some newly emerging literature optimizers. AQSCA outperforms the algorithm employed in most optimization cases and effectively challenges multi-dimensional chemical equilibrium problems.

Overall, favorable hybridization between these two optimizers considerably enhances the search efficiency and convergence behavior of the standard Aquila Optimizer, which suffers from excessive exploration of the solution domain and neglects the exploitation of promising regions. Future projections for this proposed algorithm can be directed toward solving phase stability problems that involve more than one phase in the heterogeneous mixture. Multi-objective design optimization of thermodynamic cycles can be another alternative application that leverages the superior optimization capabilities of the proposed AQSCA. AQSCA’s versatility can be extended to multi-strategy enhancement and adaptive variants that integrate several novel search schemes guided by an intelligent adaptive mechanism. In addition, machine learning algorithms can be alternatively employed to AQSCA for identifying tunable model parameters that improve robustness in high-dimensional problems with varying function landscapes. Instead of using Levy flights to improve exploration, quantum-inspired enhancements can also be integrated into the proposed search scheme. Dynamic population sizing can also be another favorable integration into the algorithm, which aims to balance computational efficiency, exploration, and exploitation concurrently by scaling the population based on algorithmic factors such as run-time and solution diversity.

## Data Availability

The datasets used during the current study are available from the corresponding author upon reasonable request.
